# Discovery of IRAK4 Inhibitors **BAY1834845** (Zabedosertib) and **BAY1830839**

**DOI:** 10.1021/acs.jmedchem.3c01714

**Published:** 2024-01-17

**Authors:** Ulrich Bothe, Judith Günther, Reinhard Nubbemeyer, Holger Siebeneicher, Sven Ring, Ulf Bömer, Michaele Peters, Alexandra Rausch, Karsten Denner, Herbert Himmel, Andreas Sutter, Ildiko Terebesi, Martin Lange, Antje M. Wengner, Nicolas Guimond, Tobias Thaler, Johannes Platzek, Uwe Eberspächer, Martina Schäfer, Holger Steuber, Thomas M. Zollner, Andreas Steinmeyer, Nicole Schmidt

**Affiliations:** Bayer AG, Research & Development, Pharmaceuticals, 13353 Berlin, Germany

## Abstract

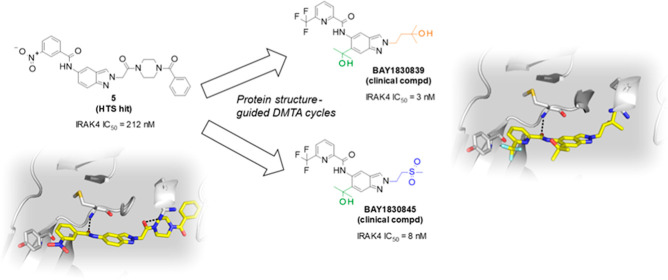

Interleukin-1 receptor-associated kinase 4 (IRAK4) plays a critical role in innate inflammatory processes. Here, we describe the discovery of two clinical candidate IRAK4 inhibitors, **BAY1834845** (zabedosertib) and **BAY1830839**, starting from a high-throughput screening hit derived from Bayer’s compound library. By exploiting binding site features distinct to IRAK4 using an in-house docking model, liabilities of the original hit could surprisingly be overcome to confer both candidates with a unique combination of good potency and selectivity. Favorable DMPK profiles and activity in animal inflammation models led to the selection of these two compounds for clinical development in patients.

## Introduction

Interleukin-1 receptor-associated kinase 4 (IRAK4) is ubiquitously expressed and is a central regulator of the innate immune system.^1^ Innate immunity is based on the recognition of either the inherent characteristics of microorganisms, called pathogen-associated molecular patterns (PAMPs), or endogenous cell-derived molecules released due to trauma, ischemia, or other tissue-destroying processes, known as damage-associated molecular patterns (DAMPs), which can be recognized by Toll-like receptors (TLRs). In humans, the TLR family comprises 10 different family members (TLR1–TLR10), each recognizing different ligands.^2^ In addition to TLRs, soluble mediators such as cytokines play an important role in innate and adaptive immunity; in particular, infection or tissue and cell stress prompts different immune cells to produce cytokines of the interleukin (IL)-1 family, which includes IL-1, IL-18, IL-33, and IL-36. Binding of these interleukins to their respective receptor leads to a potent modulation of inflammation.^3^

**Figure 1:**
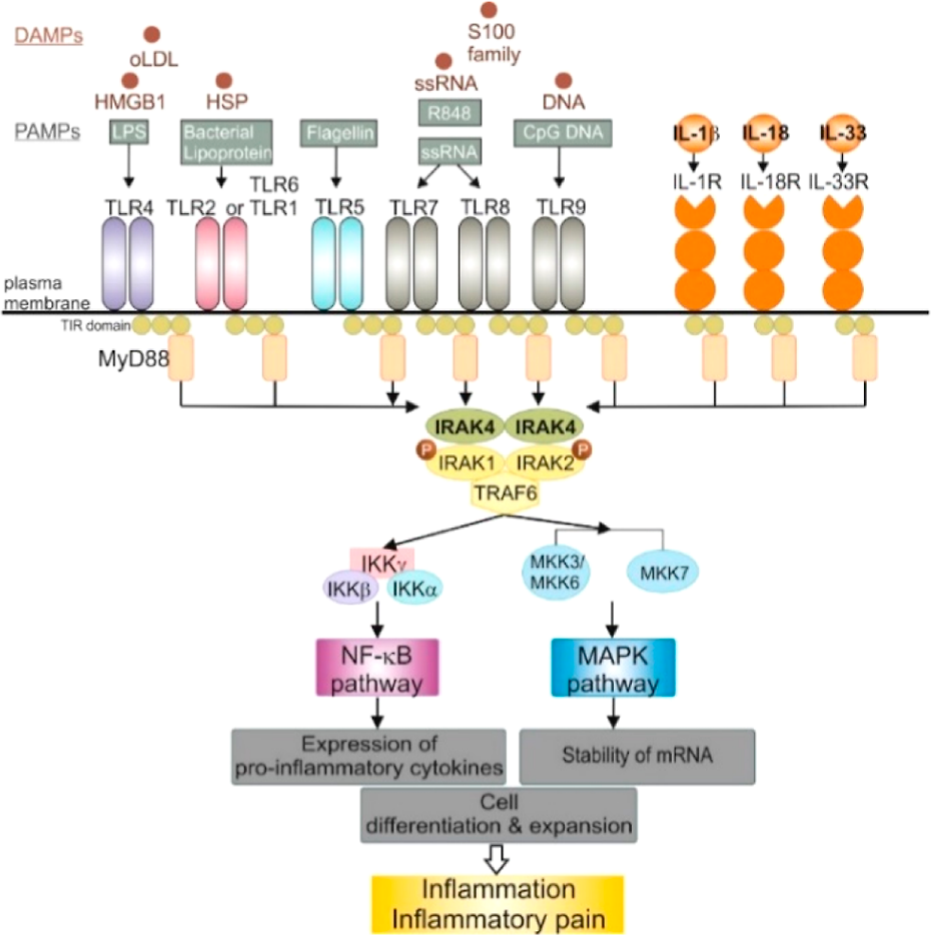
IRAK4 plays a central role in TLR/IL-1R signaling pathways. Recognition of different TLR ligands, such as oxidized low-density lipoproteins (oLDL), high mobility group box 1 (HMGB1) protein, heat shock protein (HSP), S100 family member proteins, and single stranded ribonucleic acid (ssRNA) by TLRs or recognition of the IL-1R ligands initiates the recruitment of myeloid differentiation primary response protein 88 (MyD88) to the respective receptor and subsequent activation of IRAK4. This facilitates the activation of further downstream processes like the NF-κB pathway or the MAPK pathway via IRAK1, IRAK2, and tumor necrosis factor receptor associated factor 6 (TRAF6). The activation of these pathways results in the expression of pro-inflammatory cytokines, the enhanced stability of mRNA, cell differentiation and expansion, driving inflammation and inflammatory pain. IKK, inhibitor of NF-κB kinase; IRAK, interleukin-1 receptor-associated kinase; MKK, MAPK kinase.

Upon binding of the respective ligand, TLRs (except TLR3) and receptors of the IL-1 (IL-1R) family signal via IRAK4 (Figure 1). In this process, ligand–receptor binding is associated with recruitment of myeloid differentiation primary response 88 (MyD88) protein to the receptor, where it interacts with IRAK4 forming the myddosome.^4^ Interaction of this complex with IRAK1 or IRAK2 leads to the autophosphorylation of IRAK4, followed by the phosphorylation of IRAK1 or IRAK2.^5^ Via further steps, the nuclear factor kappa B (NF-κB) and mitogen-activated protein kinase (MAPK) pathways are activated.^6,7^ Finally, activation of this MyD88–IRAK4 pathway promotes other processes associated with inflammation and inflammatory pain, including the increased expression of different inflammatory signaling molecules and enzymes (cytokines, chemokines, and cyclooxygenase-2 [COX-2]) and the enhancement of mRNA stability for various inflammation-associated genes.^8,9^ In addition, processes associated with the proliferation and differentiation of certain cell types, especially immune cells, are affected.^10,11^

In summary, IRAK4 appears to be indispensable in TLR/IL-1R signaling, and activation of the IRAK4 pathway is associated with inflammatory and autoimmune disorders such as atopic dermatitis, lupus erythematosus, hidradenitis suppurativa, and rheumatoid arthritis. Modulation of IRAK4 activity thus presents an attractive therapeutic approach for the treatment of several immune-inflammatory diseases.

Numerous IRAK4 inhibitors have been identified and several structural classes have been discussed in the literature;^12−14^ all target the ATP binding site. The druggability of this target site is evidenced by a very high *K*_m_ of ∼1 mM (according to in-house assessment—see Experimental Section), making it comparatively easy for an IRAK4 inhibitor to compete with cellular ATP levels. Collectively, the inhibitor compounds and their binding modes reflect a relatively small pocket with helix αC adopting an “αC-in” conformation.^15^ A limited number of type II inhibitors have been reported for IRAK4, and to date, the KLIFS database^16^ only lists two IRAK4 structures in which inhibitors target a deep pocket in DFG-out conformation (PDB entries 6EGS and 6EGA).^17^

**Figure 2:**
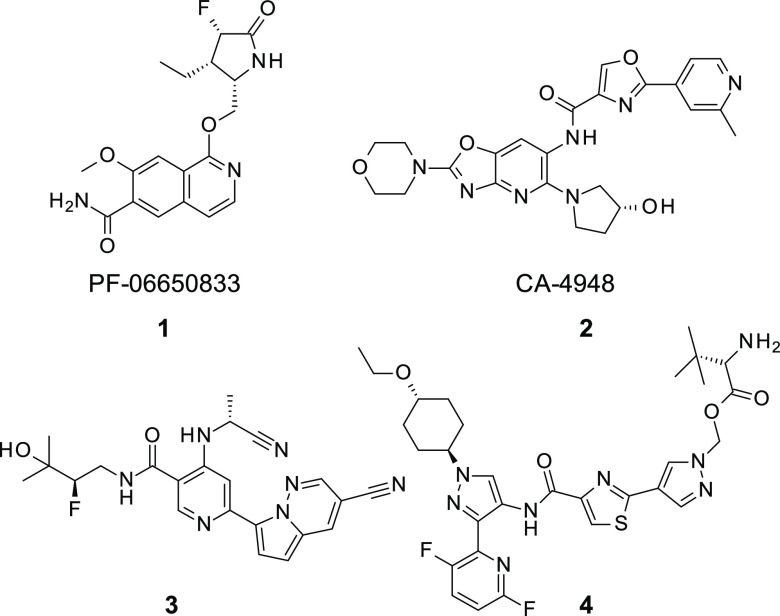
Clinical IRAK4 inhibitors from Pfizer, Inc. **1** and Curis, Inc.—Aurigene Discovery Technologies Ltd. **2** and representative examples taken from patent applications filed by Gilead Sciences, Inc. **3** and Rigel Pharmaceuticals **4**.

So far, only a few IRAK4 inhibitors have progressed to clinical development, despite the substantial efforts by several research groups (Figure 2). The highly potent and selective IRAK4 inhibitor PF-06650833 **1** (Pfizer, Inc.), generated by a fragment-based drug design approach, is currently under clinical investigation for the treatment of inflammatory diseases. According to the published preclinical data, PF-06650833 was absorbed with low-to-moderate oral bioavailability in rats, dogs, and monkeys.^18^ Clinical trials studying the IRAK4 inhibitor CA-4948 **2** (Curis Inc.—Aurigene Discovery Technologies Ltd.; IRAK4 IC_50_ = 0.03 μM) for the treatment of relapsed or refractory hematologic malignancies, acute myelogenous leukemia, and myelodysplastic syndrome are ongoing.^19^ IRAK4 inhibitor GS-5718 (Gilead Sciences, Inc., structure not disclosed) has been investigated in a phase 1b clinical study for the treatment of cutaneous lupus erythematosus (ClinicalTrials.gov Identifier: NCT04809623, study suspended). Pyrimidine carboxamides such as compound **3** have been described in a patent application filed by researchers at Gilead Sciences. The undisclosed IRAK4 inhibitor, R835 (Rigel Pharmaceuticals), established proof-of-mechanism in a first-in-human study by demonstrating the inhibition of inflammatory cytokine production in response to TLR4 signaling in a lipopolysaccharide-challenge test.^20^ A patent application describing prodrugs such as compound **4** has been filed by Rigel Pharmaceuticals.^21^ A clinical study of IRAK4 inhibitor TQH3821 (undisclosed structure) in a tablet formulation for rheumatoid arthritis has been started by the Chia Tai Tianquing Pharmaceutical Group,^22^ and Evommune/Dermira is currently investigating undisclosed compound EVO101 in topical administration for atopic dermatitis.^23^ In addition, AstraZeneca has started a safety and tolerability study of AZD6793 (ClinicalTrials.gov Identifier: NCT 05662033). Indazole-based IRAK4 inhibitors have also recently been described by Zhejiang Hisun Pharmceutical Co. Ltd.^24^ Finally, Kymera advanced IRAK4 degrader KT-474 to Phase 2 clinical trials for the treatment of atopic dermatitis and hidradenitis suppurativa (ClinicalTrials.gov Identifiers: NCT06058156 and NCT06028230, studies initiated by collaborator Sanofi). Results from the Phase 1 clinical trial (ClinicalTrials.gov Identifier: NCT04772885) were recently published.^25^

## Results and Discussion

Our approach to generating an orally active IRAK4 inhibitor started with high throughput screening (HTS) of 3 million compounds in Bayer’s proprietary compound library. Careful analysis of the resulting hits included the assessment of both their profile in Bayer’s in silico absorption, distribution, metabolism, and excretion (ADMET) platform^26^ and their experimental profile resulting from in-house bioactivity data.^27,28^ For the latter, the cross-reactivity of each compound with other protein kinases was assessed using a representative kinase panel. Structure–activity relationship (SAR) trends within a given cluster series were considered particularly relevant, and given the objective of developing a highly selective IRAK4 inhibitor, all promiscuous kinase inhibitor series were discarded early in the screening cascade. To assess the potential of structural clusters for obtaining kinase selectivity beyond the level that was already given in the initial HTS hits, we employed docking^29^ to derive hypothetical binding modes. Based on these poses, we checked how well the estimated exit vectors matched the known selectivity handles of IRAK4.

From the HTS hit-list review, compound **5** was considered a potential starting point despite its unfavorably high molecular weight and the presence of a nitro group, which is often associated with toxicity.^30^**5** exhibited reasonable target activity (IRAK4 inhibition assay based on 1 mM ATP concentration: IC_50_ = 212 nM; inhibition of tumor necrosis factor [TNF]-alpha release in THP-1 cells challenged by lipopolysaccharides [LPS]: IC_50_ = 2.3 μM) as well as a very promising kinase selectivity profile in the in-house panel and in a KINOMEscan (DiscoveRx Corp., Fremont, USA, see Supporting Information). The compound was stable in hepatocytes (rat) and showed no evidence of CYP3A4, CYP1A2, CYP2C8, CYP2C9, or CYP2D6 inhibition at a 10 μM concentration. It did, however, exhibit strong efflux in a Caco-2 assay (Table 1).

**Table 1:**
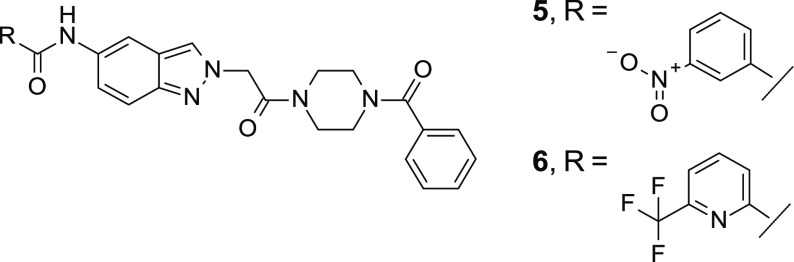
In Vitro Data for Original Hit Compound **5** and Compound **6** Displaying a Trifluoromethylpyridine Moiety

compd	IRAK4 IC_50_ (nM)^a^	TNF-alpha IC_50_ THP1 cells (μM)^b^	heps(r) CL_blood_ (L/h/kg), *F*_max_ (%)^c^	Caco-2 *P*_app_ A–B (nm/s), efflux ratio	CYP inhibition IC_50_ (μM)
**5**	212	2.3	0.42, 90	2, 101	3A4, 1A2, 2C8, 2C9, 2D6 > 10
**6**	229	2.7	0.49, 88	107, 1.65	3A4, 1A2, 2C8, 2C9, 2D6 > 20

a Biochemical potency of IRAK4 inhibition (1 mM ATP).

b Inhibition of TNF-alpha release in THP1 cells challenged by LPS.

c In vitro stability in rat hepatocytes (heps(r)). *F*_max_: calculated maximal oral availability.

We hypothesized after careful assessment of the residues lining the binding-site of compound **5** that we could simultaneously replace the nitrophenyl group, reduce molecular weight, and expand the observed kinase selectivity to more kinome family members (see Figure S4). A docking pose based on the IRAK4 cocrystal structure^15^ led to the hypothesis that the positioning of the nitrophenyl group in direct proximity to the Tyr gatekeeper provides an opportunity to modulate aromatic–aromatic interactions by varying the aromatic system. This would allow for exploitation of this binding-site feature, which is unique to IRAKs in the human kinome. The proposed binding mode employs the *trans*-amide as a hinge binder. As such, it features a single hydrogen bond at the hinge region, a feature not found in many of our various HTS series when docked to the pocket. Because the moderate strength of the hinge binder was considered an advantage in the context of the desired selectivity profile, we sought to achieve maximal augmentation of binding affinity using binding interactions that are less conserved within the protein-kinase family. Docking experiments showed that the N2-position of the indazole provides a convenient exit vector to address the IRAK4 front pocket. This subpocket in IRAK4 can empirically be used to augment selectivity over many other kinases. These findings were supported by matched pair analyses^31−33^ that showed a lower IC_50_ for N2-substituted structures versus their corresponding N1-substituted matches (data not shown). Although from the perspective of available crystal structure data, the front pocket marks the transition of the kinase domain to solvent space, the pronounced SAR observed in this area hinted at contributions to binding related to dynamic effects.

Focusing on the back pocket part of the molecule, we first set out to replace the nitrophenyl moiety. To generate a small tailored set of amides that allowed exploration of the IRAK4 back pocket, we resorted to protein structure-based design methods. A virtual library of amides was enumerated based on available benzoic acid and picolinic acid building blocks that provide drug-like molecules devoid of unwanted moieties. The aminoindazole part of each molecule was kept identical to that of compound **5** to allow for matched pair analyses. Each entry in the enumerated library was assessed using docking experiments based on the publicly available crystallographic structure.^15^ The compound set was prefiltered based on docking scores in the IRAK4 pocket and then compounds were selected for synthesis based on visual inspection of their poses, thus ensuring a fine granular sampling of the electronic properties of the aromatic ring system interacting with Tyr262. The most promising derivative originating from this set of synthesized compounds was compound **6**, which contains a trifluoromethylpyridine moiety. **6** showed comparable in vitro potency to **5**, but with a greatly improved permeability profile according to the data derived from the Caco-2 assay (Table 1).

Next, to check for a potential influence on the compound’s metabolism, we focused on the introduction of substituents at the 6-position of the indazole core (substituent R^1^ in Table 2). Surprisingly, we found a marked increase in potency by introducing a methyl group, a fluorine atom, or a chlorine atom (Table 2; compounds **7**–**9**). Further potency increases reaching single-digit nanomolar potencies were accomplished using alkoxy groups (Table 2; compounds **10** and **11**).

**Table 2:**
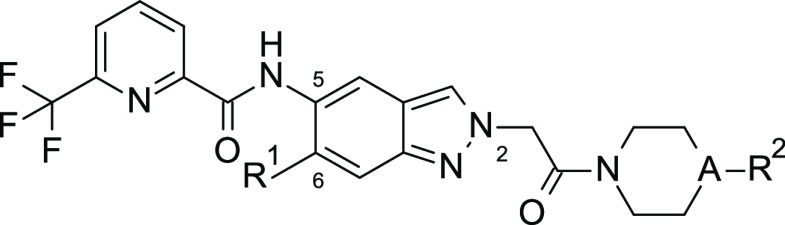
SAR: Introduction of R^1^ Substituents Underneath the Nucleotide Binding Loop and Combination with a *N*-Methylpiperazine Moiety

compd	IRAK4 IC_50_ (nM)^a^	A	R^1^	R^2^
**7**	111	N	Me	benzoyl
**8**	83	N	F	benzoyl
**9**	51	N	Cl	benzoyl
**10**	6	N	OEt	benzoyl
**11**	13	N	O*i*Pr	benzoyl
**12**	961	N	F	Me
**13**	126	N	Cyano	Me
**14**	57	N	OMe	Me
**15**	38	N	OCF_3_	Me
**16**	5	N	OEt	Me
**17**	4	N	O*i*Pr	Me
**18**	3	N	cyclopropylmethoxy	Me
**19**	12	O	OMe	
**20**	12	O	OEt	

a Biochemical potency of IRAK4 inhibition (1 mM ATP).

Importantly, we were able to reduce the molecular weight by replacing the benzoyl group with a methyl substituent (substituent R^2^ in Table 2); this resulted in compounds containing a basic *N*-methylpiperazine moiety, which facilitated ammonium salt formation, thus increasing aqueous solubility. Again, combinations with several substituents at the 6-positon were evaluated (compounds **12**–**18**); this process produced a set of highly potent compounds containing an alkoxy substituent. In addition, a morpholine moiety was found to be a good replacement for the benzoylpiperazine moiety of the original hit (compounds **19** and **20**).

Upon further profiling, we found that compound **14** was moderately stable in rat hepatocytes and exhibited a high permeability and low efflux in the Caco-2 assay. Compound **14** exhibited no relevant CYP inhibition, moderate CYP3A4 induction, and strong CYP1A2 induction (Table 3). The latter property, which discouraged further characterization of this compound, could be a result of the combination of molecule planarity and the presence of a basic nitrogen atom in the piperazine moiety.^34^ Interestingly, compound **14** showed low clearance following intravenous (iv) administration and moderate oral availability in rats (Table 4).

**Table 3:** In Vitro Data for Compound **14** (Containing a *N*-Methylpiperazine Moiety) and Compound **20** (Containing a Morpholine Ring)

compd	heps(r) CL_blood_ (L/h/kg), *F*_max_ (%)^a^	Caco-2 *P*_app_ A–B (nm/s), efflux ratio	CYP inhibition IC_50_ (μM)	CYP induction NOEL (μg/L)
**14**	2.62, 38	102, 1.0	3A4, 1A2, 2C8, 2C9, 2D6 > 20	1A2 = 7; 3A4 = 1667
**20**	0.26, 94	190, 0.5	3A4, 1A2, 2C8, 2C9, 2D6 > 10	1A2 = 3333; 3A4 = 3333

a In vitro stability in rat hepatocytes (heps(r)). *F*_max_: calculated maximal oral availability.

**Table 4:** In Vivo PK Data in Rats: Compound **14** and **20**

compd	In vivo PK rat
**14**	CL_blood_ = 0.50 L/h/kg, *t*_1/2_ = 2.9 h, *V*_ss_ = 1.6 L/kg, *F* = 41%
**20**	CL_blood_ = 0.31 L/h/kg, *t*_1/2_ = 2.9 h, *V*_ss_ = 1.3 L/kg, *F* = 46%

Compound **20**, in which the *N*-methylpiperazine moiety was replaced by morpholine, exhibited no relevant CYP inhibition or induction, significantly higher metabolic stability in rat hepatocytes than the parent molecule, and high permeability (Table 3). Oral availability of the compound was moderate, which we hypothesized to be related to low aqueous solubility (2 mg/L in phosphate-buffered saline [DMSO solution] at pH 7.4) (Table 4).

Improvement of the selectivity profile across the kinome remained a key challenge throughout the project. Therefore, we followed a strategy that was built on two pillars: (1) we systematically exploited sequential differences between pairs of kinases in the ATP-binding site and worked with the differences in size and polarity of the residues in direct interaction with the inhibitor. Where possible, 3D protein structural information and protein structure-based design techniques were used. (2) We took a more empirical and data-driven approach toward protein kinases that inherently feature a high level of promiscuity in their ability to bind to ATP-competitive inhibitors. Relatively recently, the molecular mechanism underlying this promiscuity was nicely characterized by a Markov state model (MSM) built for discoidin domain receptor tyrosine kinase 1 (DDR1).^35^ Based on the IC_50_-data collected over many years from our in-house kinase projects, we identified fms-like receptor tyrosine kinase 3 (FLT3) as the most promiscuous kinase. Moreover, we empirically found that across a number of kinase projects the level of FLT3 selectivity of a compound is often indicative of its kinase selectivity (for an overview of kinase selectivity metrics, see Bosc et al.^36^). Therefore, we used FLT3 as the benchmark for monitoring the kinase selectivity in the IRAK4 program.

Having identified that the morpholine and methoxy groups were substituents that granted high potency, we focused on further variation of the trifluoromethylpyridine moiety and monitored selectivity against FLT3 (Table 5). Exchanging the trifluoromethyl substituent with methyl (compound **21**) led to a decrease in potency. Introduction of a difluoromethyl substituent (compound **22**) conferred slightly enhanced potency, but a shorter *t*_1/2_ in rats following iv administration (in vivo PK rat: CL_blood_ = 0.14 L/h/kg, *t*_1/2_ = 1.6 h, *V*_ss_ = 0.2 L/kg, *F* = 55%). Substituents with a hydroxyl substituent were then introduced to improve the aqueous solubility (compounds **23**–**26**). Only *R*-enantiomer **23** exhibited acceptable biochemical potency, but the compound also exhibited high efflux in the Caco-2 assay (*P*_app_ A–B = 20 nm/s, efflux ratio = 12). Its cocrystal structure with IRAK4, which also served to determine the absolute stereochemistry of the enantiomer, showed that it formed an additional hydrogen bond to the conserved Asp329 via its hydroxyl group (see crystal structure PDB 8ATL, Supporting Information, Figure S3), in line with its largely unchanged kinase selectivity profile based on kinase panel results.

**Table 5:**
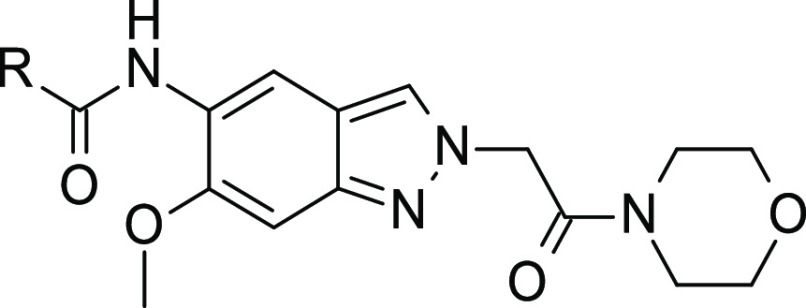

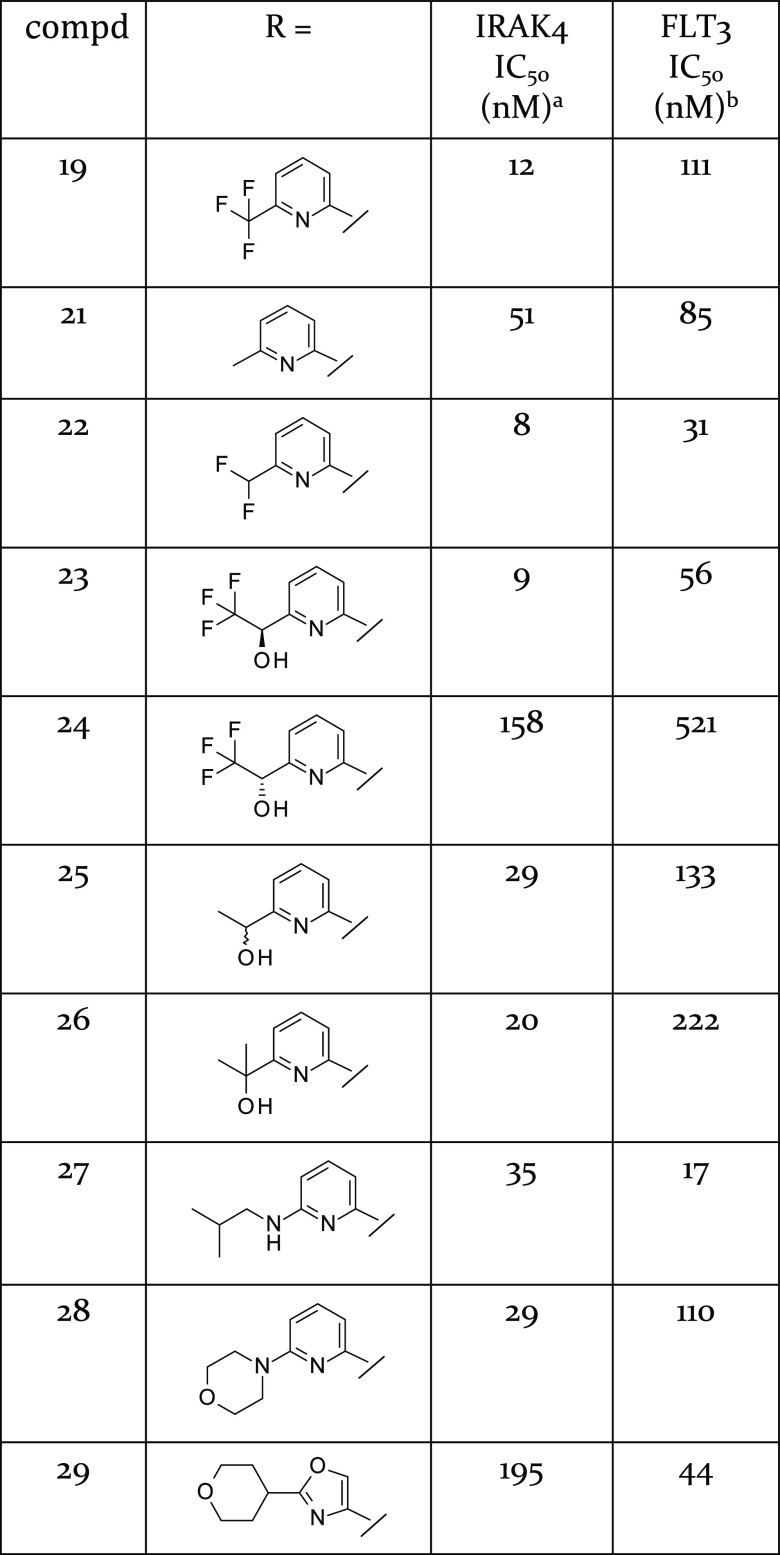
SAR: Introduction of Substituents R Pointing to the Backwall of the ATP-Pocket

a Biochemical potency of IRAK4 inhibition (1 mM ATP).

b Biochemical potency of FLT3 inhibition (10 μM ATP).

Notably, efflux was lower for compound **26** (*P*_app_ A–B = 53 nm/s, efflux ratio = 4). Introduction of an alkylamine-, a morpholine-, or an oxazole-based substituent led to insufficient potency (compounds **27**–**29**). Compound **29** exhibited reduced selectivity relative to the benchmark kinase FLT3, and its back pocket substituent appeared to be too large to fit into the IRAK4 pocket comfortably.

**Figure 3:**
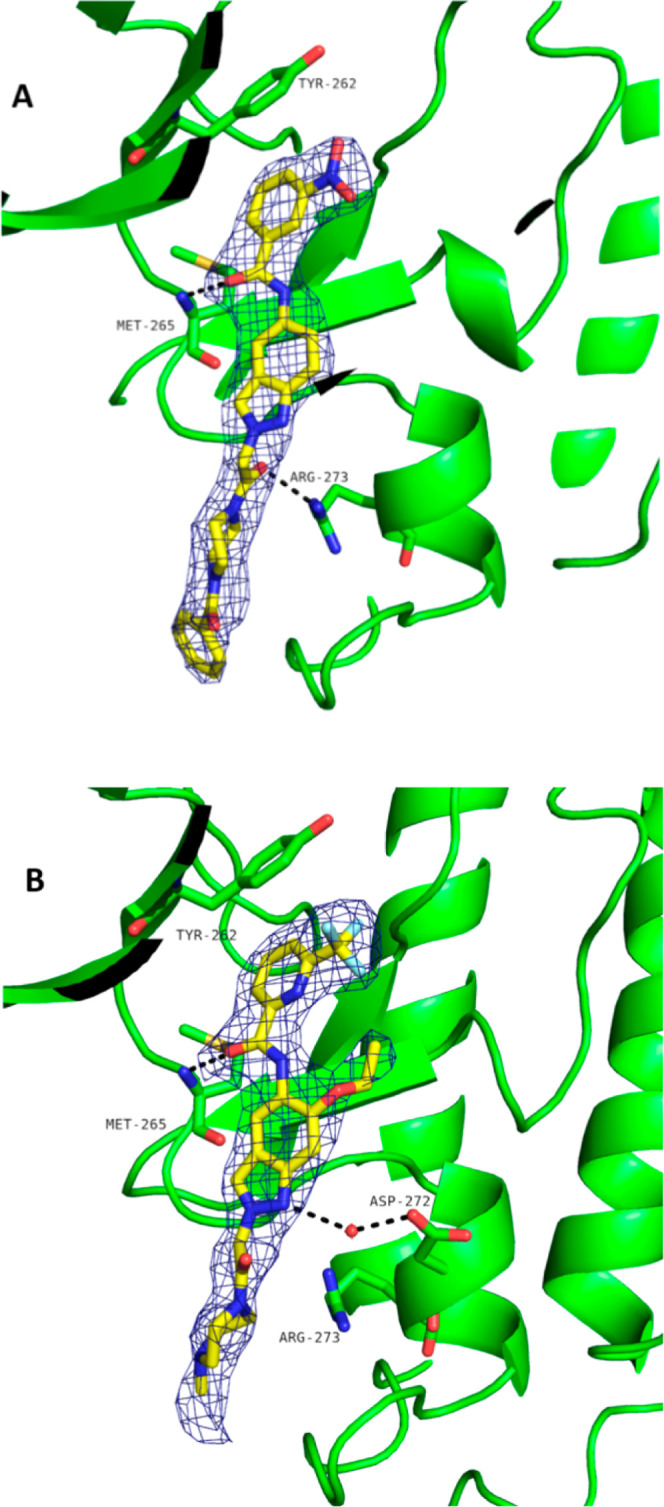
Cocrystal structures of compound **5** (A, PDB 8BR7) and compound **16** (B, PDB 8ATB) with IRAK4.

Generation of a cocrystal structure was first achieved with compound **5** (see Figure 3A). The binding mode was in line with PDB 2NRU, but the orientation of the N2-substituent was different in the details. Although the protein crystallized in a different space group, its structure largely resembled that published by Wang et al.^15^ with some recognizable differences in the conformation of the nucleotide binding loop (NBL). The piperazine carboxamide formed a distorted hydrogen bond with Arg273, similar to the interaction pattern of the ligand in the structure 2NRU as reported by Wang et al. In molecular dynamics simulation, this hydrogen bond to Arg273 was observed only half of the time, supporting SAR data that suggested this was not a key interaction in target binding. Later, cocrystallization of compound **16** was accomplished (see Figure 3B), allowing an in-depth analysis of the influence of the substituent at the 6-position binding under the NBL (see formula of Table 2 for atom numbering). No strong local interactions with the protein were observed, leaving open the question of the molecular basis of the pronounced effect on potency that this substituent had. Later, when we characterized the binding kinetics of the series, the 6-substituent was identified as a major modulator of the off-rates (data not shown). The aforementioned hydrogen bond to Arg273 was not observed in chain A of PDB 8ATB. Instead, a water-mediated H-bond between the indazole core and Asp272 was formed.

**Figure 4:**
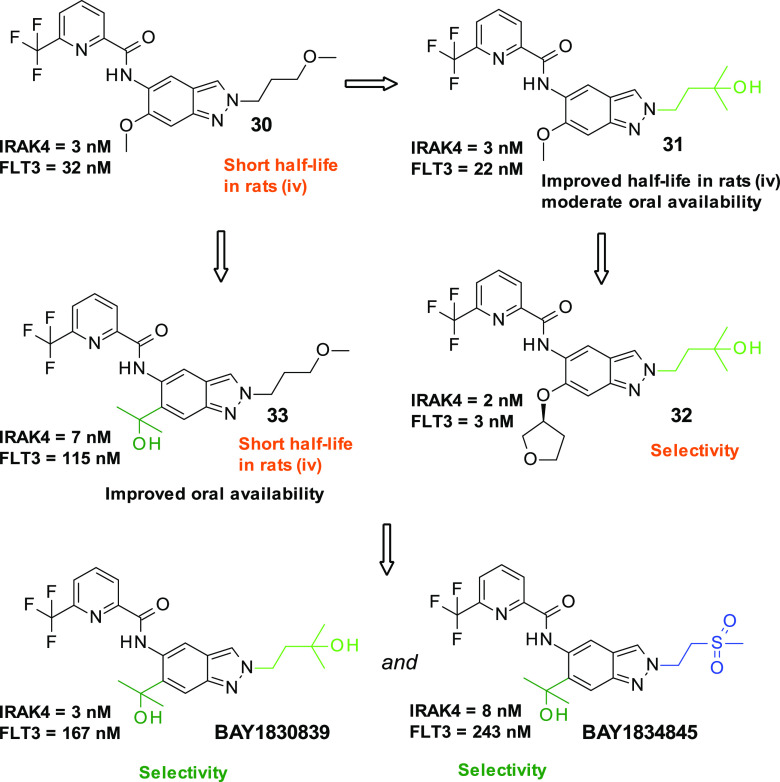
Compounds **30–33** and identification of clinical compounds **BAY1830839** and **BAY1834845**, starting from compound **30** based on key selectivity data and PK data from in vivo studies in rats. Biochemical potency data for IRAK4 inhibition (1 mM ATP) and FLT3 inhibition (10 μM ATP) are also shown.

We then investigated the derivatization of position 2 of the indazole (Figure 4 and Table 6). We found that compound **30**, which contained a 3-methoxypropyl substituent, exhibited stronger potency relative to compound **19**. This compound also exhibited high selectivity for the benchmark kinase FLT3, but a short *t*_1/2_ in rats following iv administration (*t*_1/2_ = 0.8 h). The introduction of a side chain containing a hydroxy group protected from oxidative metabolism led to equally potent compound **31** with a much more promising PK profile but still moderate oral availability. We then focused on the derivatization of the 6-position of the indazole core and found compound **32** containing a tetrahydrofuranylether moiety with comparable potency but significantly lower selectivity for the benchmark kinase FLT3.

**Table 6:** In Vivo PK Data from Rats for Compounds **30**, **31**, and **33**

compd	in vivo PK rat
30	CL_blood_ = 1.99 L/h/kg, *t*_1/2_ = 0.8 h, *V*_ss_ = 1.4 L/kg, *F* = 40%
31	CL_blood_ = 0.43 L/h/kg, *t*_1/2_ = 3.6 h, *V*_ss_ = 1.8 L/kg, *F* = 58%
33	CL_blood_ = 2.3 L/h/kg, *t*_1/2_ = 0.9 h, *V*_ss_ = 1.8 L/kg, *F* = 76%

Replacing the methoxy group at the 6-position of the indazole core with a 2-hydroxy-propan-2-yl substituent protected from oxidative metabolism resulted in compound **33**, which was found to be slightly less potent than compound **30** but showed promising selectivity for FLT3. The assessment of compound **33** in an in vivo PK study in rats found it to have acceptable oral bioavailability; however, the relatively high clearance and short *t*_1/2_ led to discontinuation of the compound. Based on the available information from compounds **31** and **33**, introducing both hydroxyl-based substituents at the 2- and 6-positions led to the generation of compound **BAY1830839**, which could be progressed as a clinical compound. To generate a second compound with clinical potential, we pursued further derivatization of the 2-position and, by introducing a sulfone moiety, generated compound **BAY1834845** (zabedosertib).

While the described SAR exploration employed the systematic modification of all substituents around the indazole–amide core motif, replacing the indazole using other heterocyclic systems that retained the exit vectors was also a viable approach to extending the SAR. Available crystal structure data showed that the indazole faced the kinase hinge region, with the C–H of the 3-position pointing toward the carbonyl oxygen atom of Met265. Notably, the indazole was not in a coplanar arrangement with the said carbonyl group; instead, its aromatic plane was significantly shifted upward toward the Tyr264 side chain. Although considerable variability in the geometry of nonclassical hydrogen bonds formed between C–H groups in kinase inhibitors with carbonyl groups has been reported before,^37^ the interaction geometries observed in our crystal structures appeared remarkable (the elevation angle ϕ, which measures the extent to which the donated hydrogen is elevated out of the plane of the acceptor lone pairs, was 46° in PDB 8ATL [compound **23**], and thus was approximately double the angle noted in PDB entry 2NRU [25° chain B, 15° chain A]).

Factoring in the aforementioned binding mode, we assessed possible core hops using docking,^29,38^ which led to the hypothesis that the C–H group of the 3-position could be replaced by an acceptor or donor atom to favorably engage in a hydrogen bond with the hydroxyl group of the Tyr264 side chain. We considered this possibility in our synthetic plans but also explored a broader range of options than scoring functions suggested. Notably, at the time of discovery, free energy perturbation (FEP) methods^39^ were not available in an implementation suitable for industrial drug discovery. Based on our previous experience with selectivity mediating hinge-binding motifs in other kinase projects,^40^ we also included alternative cores in our synthetic efforts that resulted in seemingly unfavorable contacts with the IRAK4 hinge region in our synthetic efforts.

A variety of compound classes based on a 5–6-bicyclic-heteroaromatic core have been described in patent applications for IRAK4 inhibitors, showing that the replacements of the 3-CH group in the indazole core described above are possible.^41−47^ We complement this data with a matched pair analysis^31−33^ for selected cores here, allowing for a quantification of the associated changes in biochemical IC_50_ values and by X-ray data that support interpretation of possible core hops in light of the recognition properties of the IRAK4 pocket.

As exemplified by the comparison of indazole **34**, which displayed a cyclic substituent containing a sulfone moiety (1,1-dioxidotetrahydro-2*H*-thiopyran-3-yl) at the 2-position, and the analogous benzimidazole-based compound **35**, the replacement of the C–H group present at the 3-position of the indazole by a classical H-bond donor led to a reduction in target potency (Table 7). This was in line with the view that formation of an H-bond to Tyr264 would involve either suboptimal geometry or a slight rearrangement of the core in the pocket. Interestingly, the methylated benzimidazole, **36**, was also found to be a potent IRAK4 inhibitor (both enantiomers were tested).^48^

**Table 7:**
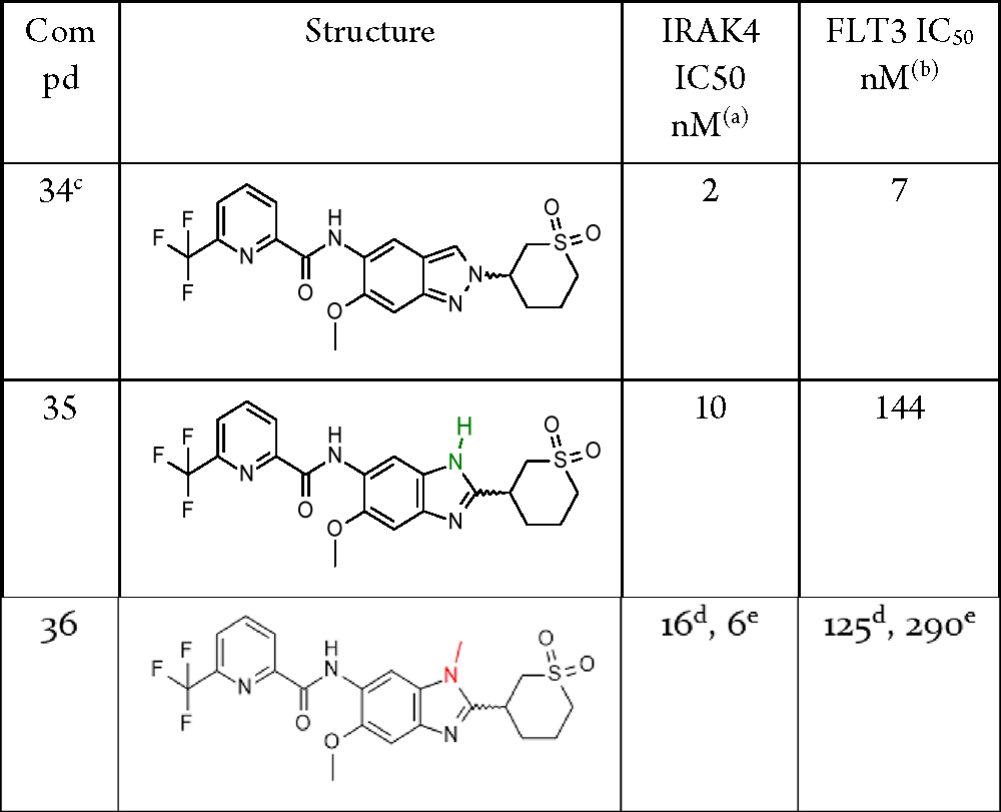
Comparison of Matched Pairs Based on Indazole and Benzimidazole Core

a Biochemical potency of IRAK4 inhibition (1 mM ATP).

b Biochemical potency of FLT3 inhibition (10 μM ATP).

c Compound purity ap. 90% according to ^1^H NMR.

d ^,*e*^Results for the individual enantiomers with unkown absolute stereochemistry.

Similarly, the steric demand of the added methyl group was also well-tolerated in the original indazole series. 3-Methyl-substituted indazoles exhibited slightly reduced or comparable potency relative to the original indazoles, as exemplified by the matched pairs **31** and **37** (Table 8). In contrast to other kinases, an *N*-ethyl substituent facing the hinge region^40^ was not well-tolerated by IRAK4 leading to a significant drop in potency (compare benzimidazole-based compounds **38** and **39**, Table 8).

**Table 8:**
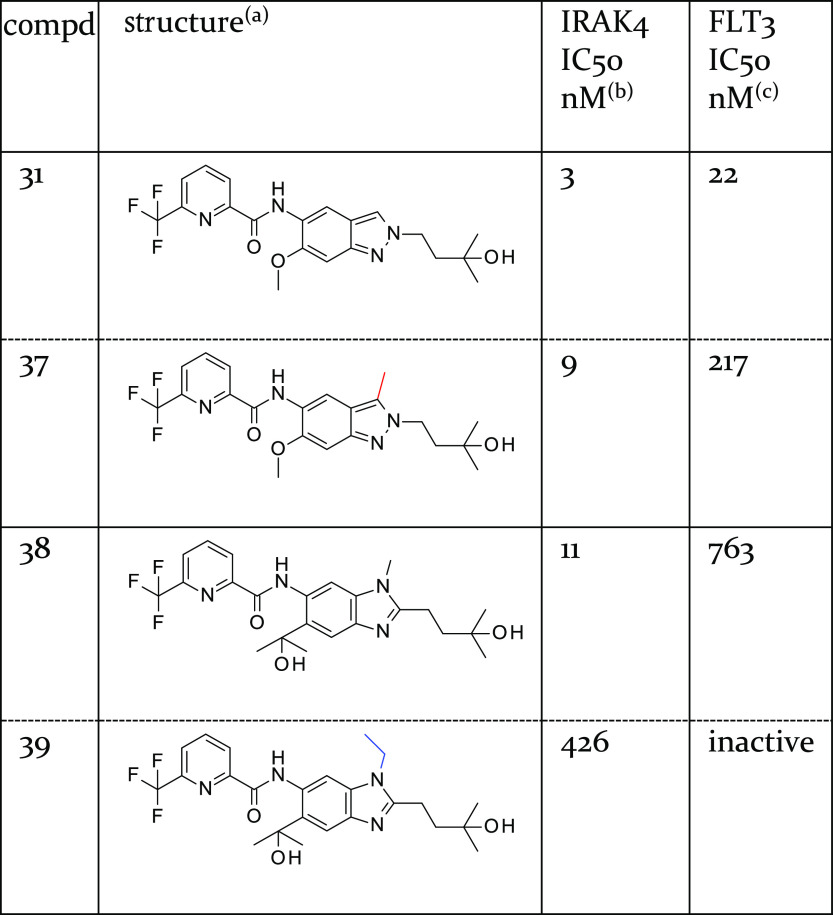
Influence of Substitution with Alkyl at the 3-Position of the Indazole (Compounds **31** and **37**) and Benzimidazole Core (Compounds **38** and **39**)

a Matched pairs are shown in adjacent rows separated by a dashed horizontal line.

b Biochemical potency of IRAK4 inhibition (1 mM ATP).

c Biochemical potency of FLT3 inhibition (10 μM ATP).

**Figure 5:**
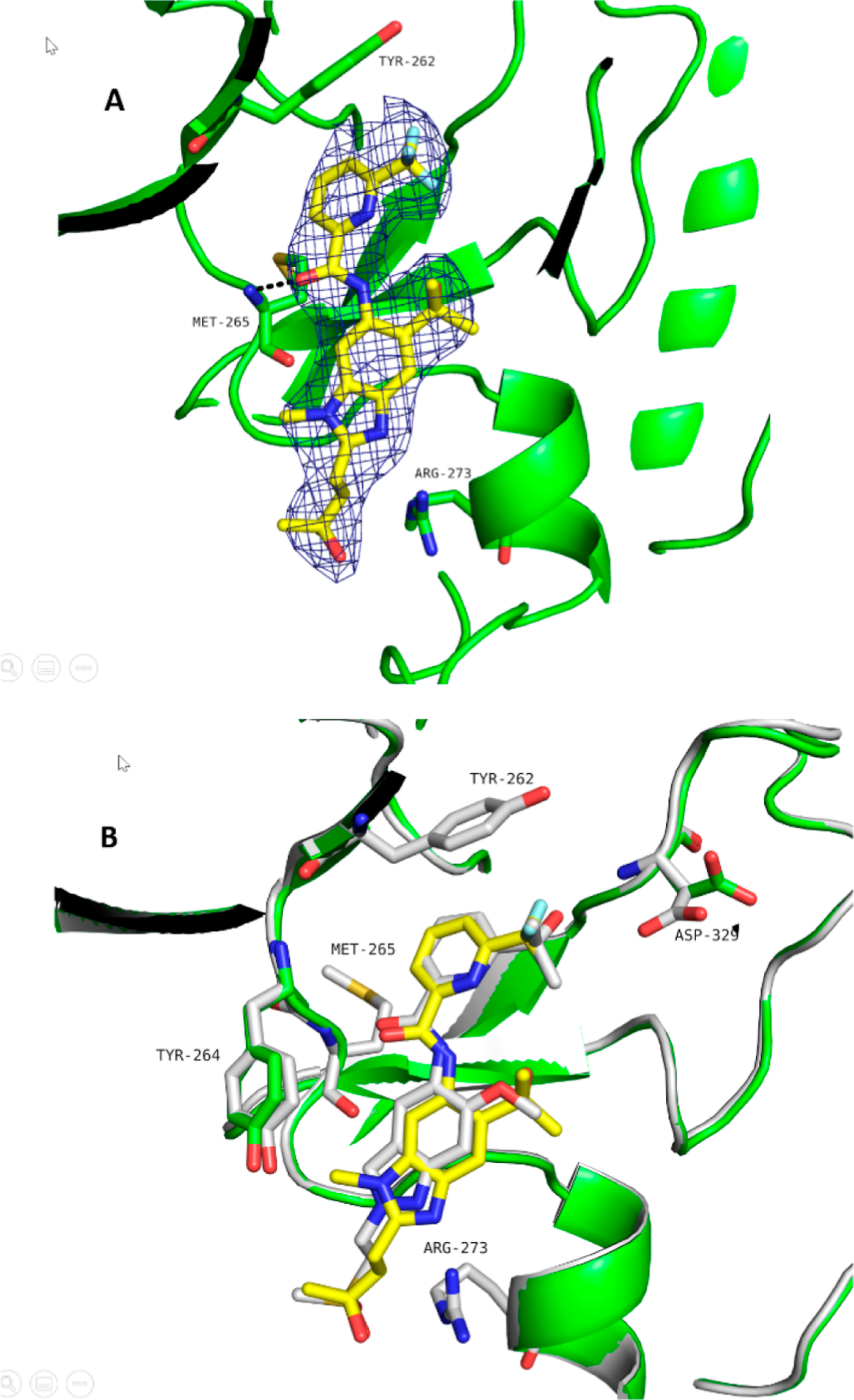
IRAK4 cocrystal structures of the methylbenzimidazole-based 38 (A, PDB 8ATN) (Table 8) and superposition with the cocrystal structure of the indazole-based compound 40 (B, PDB 8BR6) (Table 9). The chemical variation in the core induced no positional shift. Moreover, the methylsulfonylethyl and the 2-hydroxy-propan-2-yl substituent take equivalent positions when used as a front pocket substituent. A remarkable difference between the two X-ray protein conformations is the reorientation of the Asp329 side chain, enabling a hydrogen bond to be formed with the hydroxyl group of the pyridyl substituent of 40. Moreover, the aromatic plane of the Tyr264 side chain is rotated in response to the binding of the methylated core.

Our interest in the methylbenzimidazole series was prompted by the observation that the series showed higher selectivity relative to the original indazole series when profiled in our in-house kinase inhibition panel (see compound **38**). This higher selectivity was also reflected in the higher IC_50_ values observed in the methylbenzimidazole series using the benchmark kinase FLT3 (see Table 8). A cocrystal structure of **38**, the methylbenzimidazole-based matched companion to clinical compound **BAY1830839**, was solved at 2.2 Å resolution (Figure 5), showing almost no shift of the core relative to the original indazole series (the cocrystal structure of indazole-based compound **40**, which contains the same methylsulfonylethyl substituent present in clinical compound **BAY1834845**, was used for comparison).

Further SAR exploration (details not shown) led to the identification of compound **41**, which exhibited reasonable potency and good selectivity against FLT3 (see Table 9) despite lacking a larger front pocket substituent. Compound **41** inhibited CYP3A4 (IC_50_ = 2 μM) to some extent and was therefore deprioritized for further optimization. Its cocrystal structure (see Figure S3) shows the pyrazole ring in a tilted orientation relative to the methylbenzimidazole core.

**Table 9:**
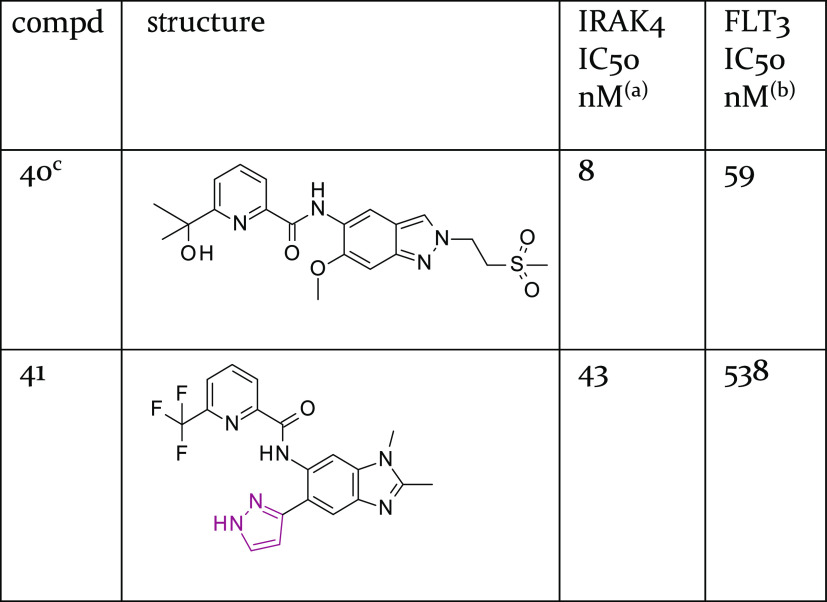
Biochemical Potency Data for Indazole-Based Compound **40** and Methylbenzimidazole-Based Compound **41**, Each of Which had a Cocrystal Structure Available

a Biochemical potency of IRAK4 inhibition (1 mM ATP).

b Biochemical potency of FLT3 inhibition (10 μM ATP).

c Compound purity 92% HPLC UV.

Overall, the use of a methylbenzimidazole core created compounds of similar quality to the original indazole series compounds with respect to several parameters, including selectivity and in vitro metabolic stability. As such, compounds with a methylbenzimidazole core were intensively pursued as additional lead series. However, none of the resulting compounds possessed a more promising overall profile than those of the clinical compounds (data not shown).

Thus, our exploration of alternative cores eventually confirmed the original indazole core as the most suitable scaffold.

**Scheme 1:**
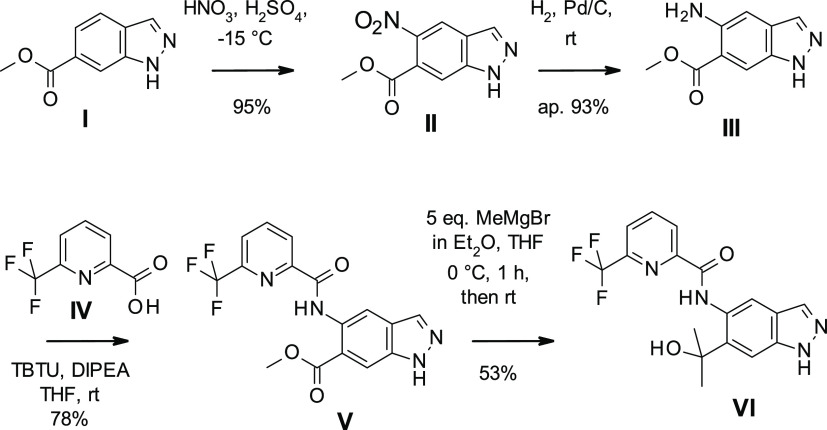
Preparation of **BAY1830839** and **BAY1834845**

For the preparation of **BAY1830839** and **BAY1834845**, we started with commercially available indazole **I** (Scheme 1). Regioselective nitration resulted in **II**, which was reduced to **III** by hydrogen ionization using palladium on charcoal. Amide coupling with **IV** led to **V**, which was then treated with excess Grignard reagent to produce **VI**.

**Scheme 2:**
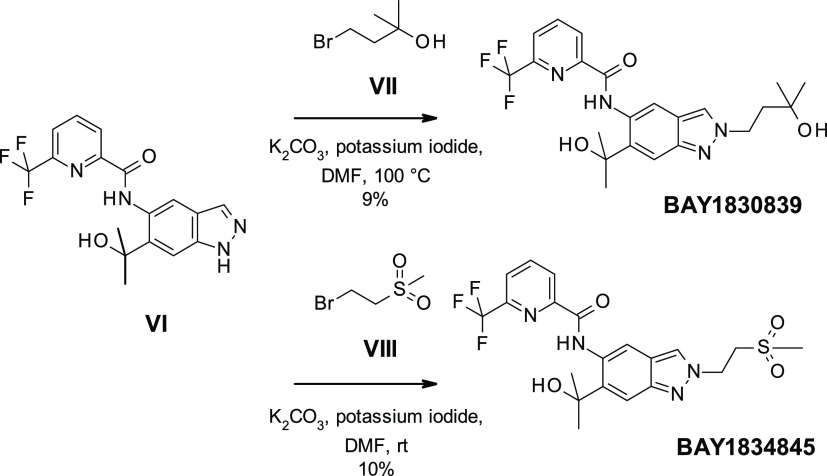
Preparation of **BAY1830839** and **BAY1834845** (Initial Route)

Scheme 2 shows that **VI** was then transformed into **BAY1830839** by alkylation with bromoalkylalcohol **VII** using potassium carbonate and potassium iodide at 100 °C. Reaction between **VI** and 1-bromo-2-(methylsulfonyl)ethane **VIII** resulted in **BAY1834845**. In both cases, yields were low due to the formation of both the undesired N1 (major isomer) and the desired N2 indazole isomer; considerable separation work was thus required to separate the products.

**Scheme 3:**
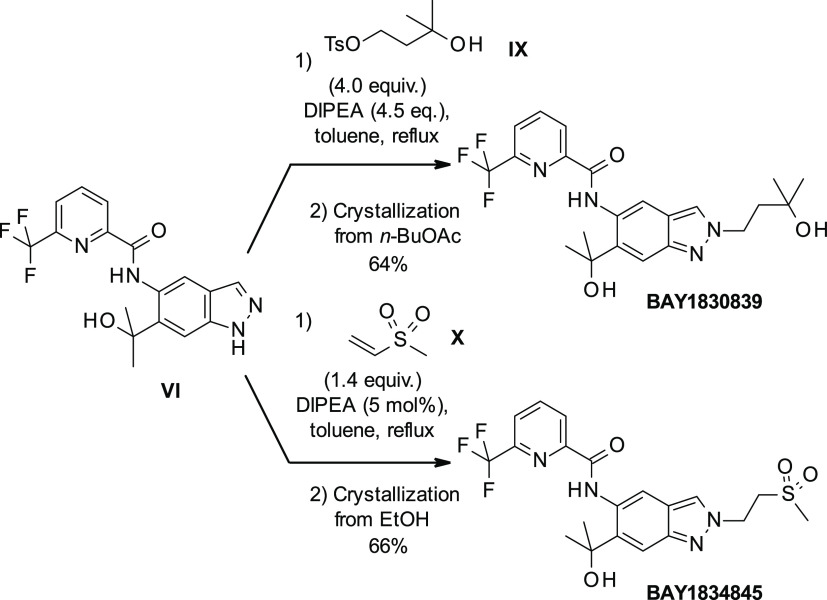
Improved Preparation of **BAY1830839** and **BAY1834845**

Further elaboration of the reaction conditions for the alkylation of the indazole N2 atom led to an improved, highly regioselective method for the synthesis of both clinical compounds (Scheme 3). Using the tosylate **IX** in the presence of a weaker base such as *N*,*N*-diisopropylethylamine (DIPEA) in the nonpolar solvent toluene at reflux temperature provided almost exclusively the desired N2 regioisomer. This strategy worked best for **BAY1830839** when a more reactive electrophile such as a tosylate was used and after workup, and crystallization resulted in isolated yields of up to 64%. For **BAY1834845**, it was found to be advantageous to use methyl vinyl sulfone **X** to serve as the Michael acceptor. In this case, only a catalytic amount of DIPEA sufficed to reach full conversion. Yields of up to 66% were reached after crystallization.

Assessment of the kinase selectivity of **BAY1830839** and **BAY1834845** in a Bayer in-house kinase panel revealed high selectivity profiles, as illustrated by the IC_50_ values of IRAK4, FLT3, and TrkA (Table 10). Both compounds exhibited high selectivity for the benchmark kinase FLT3, and they also somewhat inhibited TrkA.

**Table 10:** Biochemical Potency Data for Clinical Compounds **BAY1830839** and **BAY1834845**

compd	IRAK4 IC_50_ (nM)^a^	FLT3 IC_50_ (nM)^a^	TrkA IC_50_ (nM)^a^
**BAY1830839**	3	167	636
**BAY1834845**	8	243	600

a Biochemical potency of IRAK4 (1 mM ATP), FLT3 (10 μM ATP), and TrkA (10 μM ATP) inhibition.

**Figure 6:**
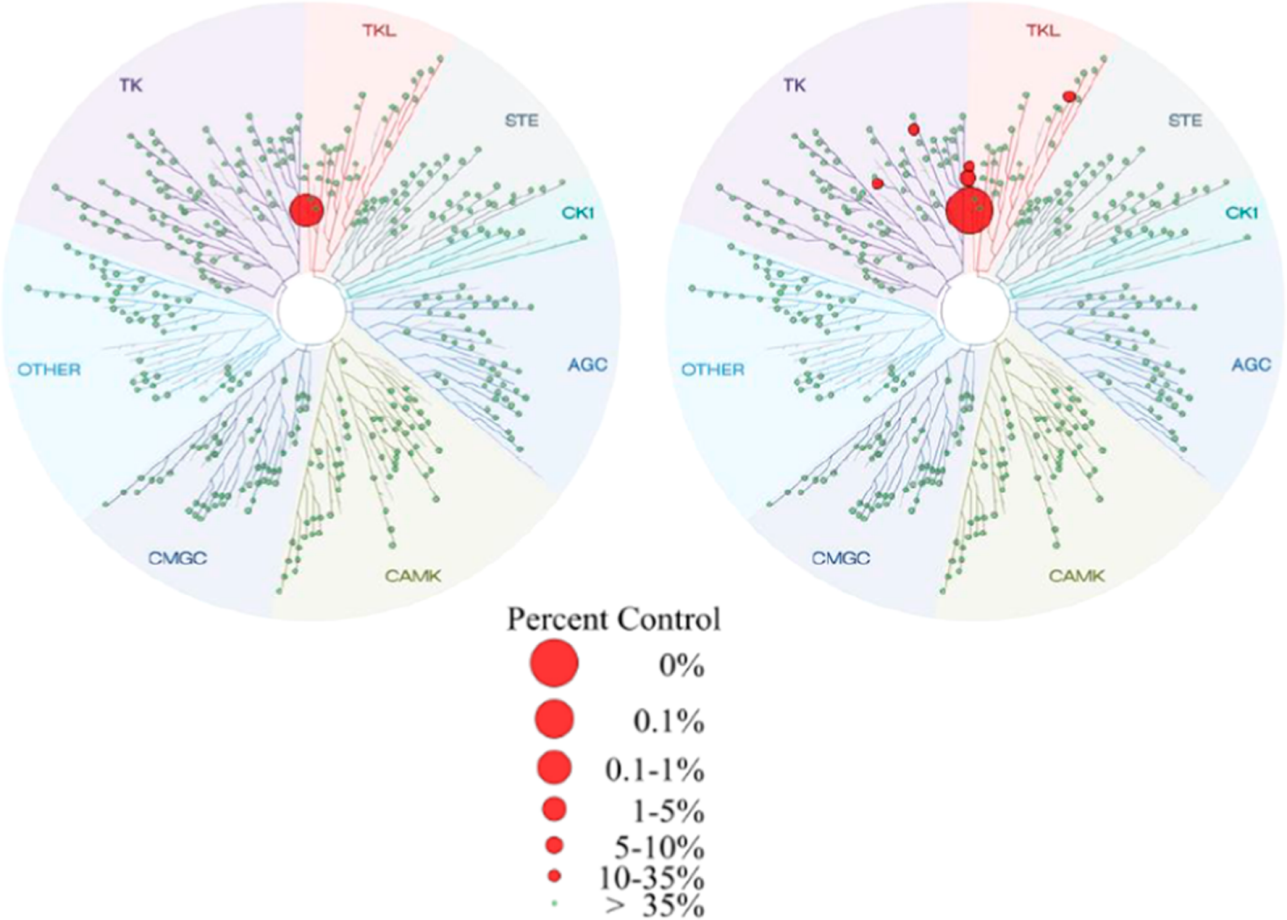
TREEspot interaction maps for **BAY1830839** at 0.1 μM (left) and 1 μM (right) compound concentration. Image generated using TREEspot Software Tool and reprinted with permission from KINOMEscan (DiscoveRx Corp., Fremont, USA).

**Figure 7:**
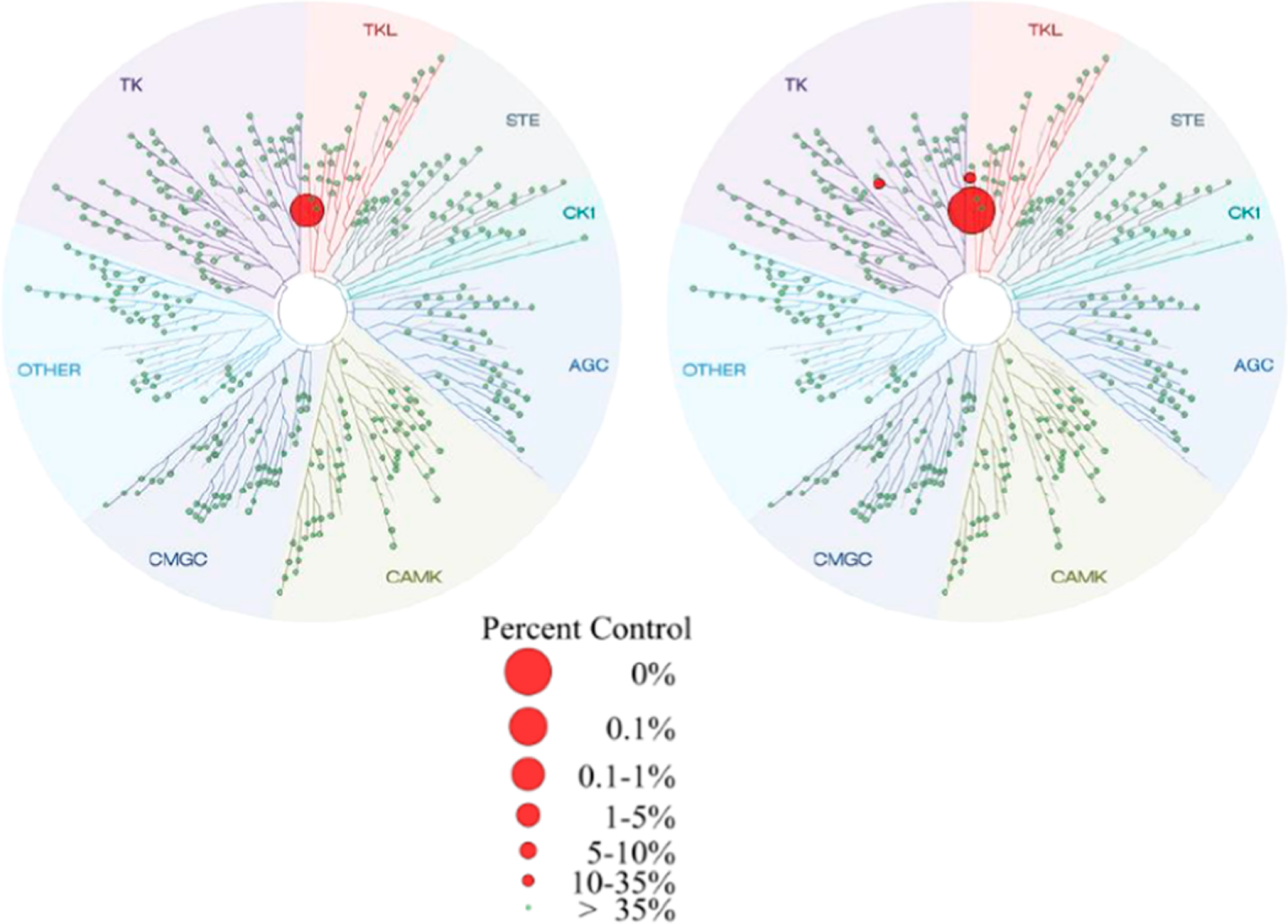
TREEspot interaction maps for **BAY1834845** at 0.1 μM (left) and 1 μM (right) compound concentration. Image generated using TREEspot Software Tool and reprinted with permission from KINOMEscan (DiscoveRx Corp., Fremont, USA).

A KINOMEscan (DiscoveRx Corp., Fremont, USA) kinase assay panel (456 kinases) confirmed the excellent selectivity profiles of **BAY1830839** (Figure 6) and **BAY1834845** (Figure 7). At 1 μM concentration, **BAY1830839** showed limited competitive binding to IRAK1/IRAK3/TrkB in addition to the interactions with kinases already identified in the internal kinase panel.

**BAY1834845** also exhibited very limited competitive binding to kinases at 1 μM concentration, demonstrating the promising kinase selectivity profile for the compound (see Supporting Information).

**BAY1830839** and **BAY1834845** are metabolically stable in vitro, as demonstrated in assays in human, primate, rat, and dog hepatocytes, with **BAY1834845** showing a slightly higher stability than **BAY1830839** (Table 11). In addition, both compounds are highly permeable based on Caco-2 cell assay data (Table 12). Following the in vitro analyses, the PK of the two compounds was then investigated in vivo. Upon iv administration in rats and dogs, **BAY1830839** showed a low-to-moderate CL_blood_; moderate-to-high *V*_ss_; intermediate-to-long *t*_1/2_, dependent on the test species; and high oral bioavailability (Table 13). In comparison, **BAY1834845** exhibited low clearance and excellent oral availability in all species (Table 14).

**Table 11:** Metabolic Stability of **BAY1830839** and **BAY1834845** in Hepatocytes

species/gender	**BAY1830839**		**BAY1834845**	
	CL_blood_ [L/h/kg]	*F*_max_ [%]^a^	CL_blood_ [L/h/kg]	*F*_max_ [%]^a^
human/male	0.17	87	stable	100
primate/female	0.63	75	0.62	75
rat/male	0.47	89	0.051	99
dog/female	0.43	80	stable	100

a *F*_max_: calculated maximal oral availability.

**Table 12:** Permeability and Transport of **BAY1830839** and **BAY1834845** in Caco-2 Cells^a^

	**BAY1830839**	**BAY1834845**
ap-bas (nm/s)	160	160
bas-ap (nm/s)	180	240
efflux ratio	1.2	1.5

a ap, apical; bas, basolateral.

**Table 13:** In Vivo PK Data for **BAY1830839**

	mouse	rat	dog
dose_iv_ (mg/kg)	0.5	0.5	0.5
AUC_norm,iv_ (kg h/L)	2.3	5.9	1.4
CL_blood_ (L/h/kg)	0.36	0.20	0.81
*V*_ss_ (L/kg)	1.6	4.3	2.6
*t*_1/2,iv_ (h)	3.0	4.3	2.6
dose_po_ (mg/kg)		2.0	1.0
AUC_norm po_ (kg h/L)		4.8	2.6
*C*_max,norm_ (kg/L)		0.49	0.37
*t*_max_ (h)		4.0	0.25
*F* (%)		82	90

**Table 14:** In Vivo PK Data for **BAY1834845**

	mouse	rat	dog
dose_iv_ (mg/kg)	0.5	0.5	0.5
AUC_norm,iv_ (kg h/L)	3.3	5.6	15
CL_blood_ (L/h/kg)	0.38	0.24	0.088
*V*_ss_ (L/kg)	1.1	0.92	1.6
*t*_1/2,iv_ (h)	2.6	4.2	17
dose_po_ (mg/kg)		2.0	1.0
AUC_norm po_ (kg h/L)		5.3	15
*C*_max,norm_ (kg/L)		0.55	0.57
*t*_max_ (h)		4.0	2.0
*F* (%)		94	104

**Figure 8:**
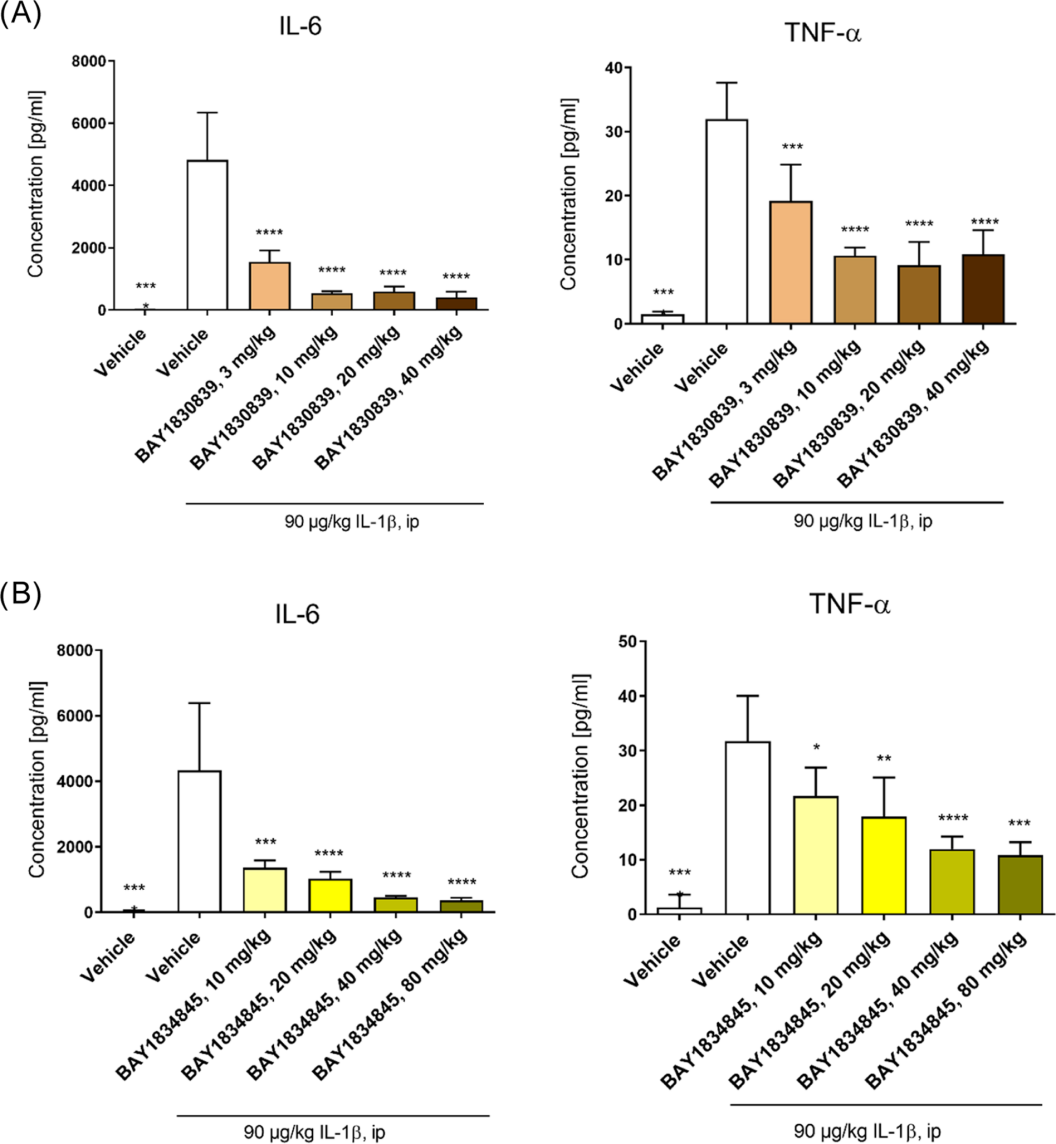
**BAY1830389** (A) and **BAY1834845** (B) dose-dependently blocked IL-1β-induced inflammation in mice. Female BALB/c mice were treated with vehicle, **BAY1830839**, or **BAY1834845**, followed by intraperitoneal injection of 90 μg/kg of IL-1β 6 h after treatment. After an additional 2 h, the animals were euthanized, and blood was collected to obtain plasma samples. IL-6 and TNF-alpha plasma levels were then determined by multiplex protein assay. Data are shown as mean ± SD for each treatment group (*n* = 5 per treatment group, except for vehicle alone [*n* = 4]; results for IL-6 levels in the 80 mg/kg **BAY1834845** group [*n* = 3]). One-way ANOVA vs IL-1β vehicle: *p < 0.05, **p < 0.01, ***p < 0.005, and ****p < 0.001.

The anti-inflammatory effects of **BAY1830839** and **BAY1834845** were investigated by using three different pharmacodynamic (PD) models. Intraperitoneal injection of IL-1β induces systemic inflammation in mice characterized by elevated plasma levels of IL-6 and TNF-alpha 2 h after administration. **BAY1830839** and **BAY1834845** significantly and dose-dependently reduced the concentration of both of these cytokines when compared with vehicle-treated mice (Figure 8; for the corresponding unbound compound exposure data, see Supporting Information, Figure S1).

Furthermore, this mouse model has been used to investigate the PK–PD relationships of **BAY1830839** and **BAY1834845** and to support human dose selection.^51^

**Figure 9:**
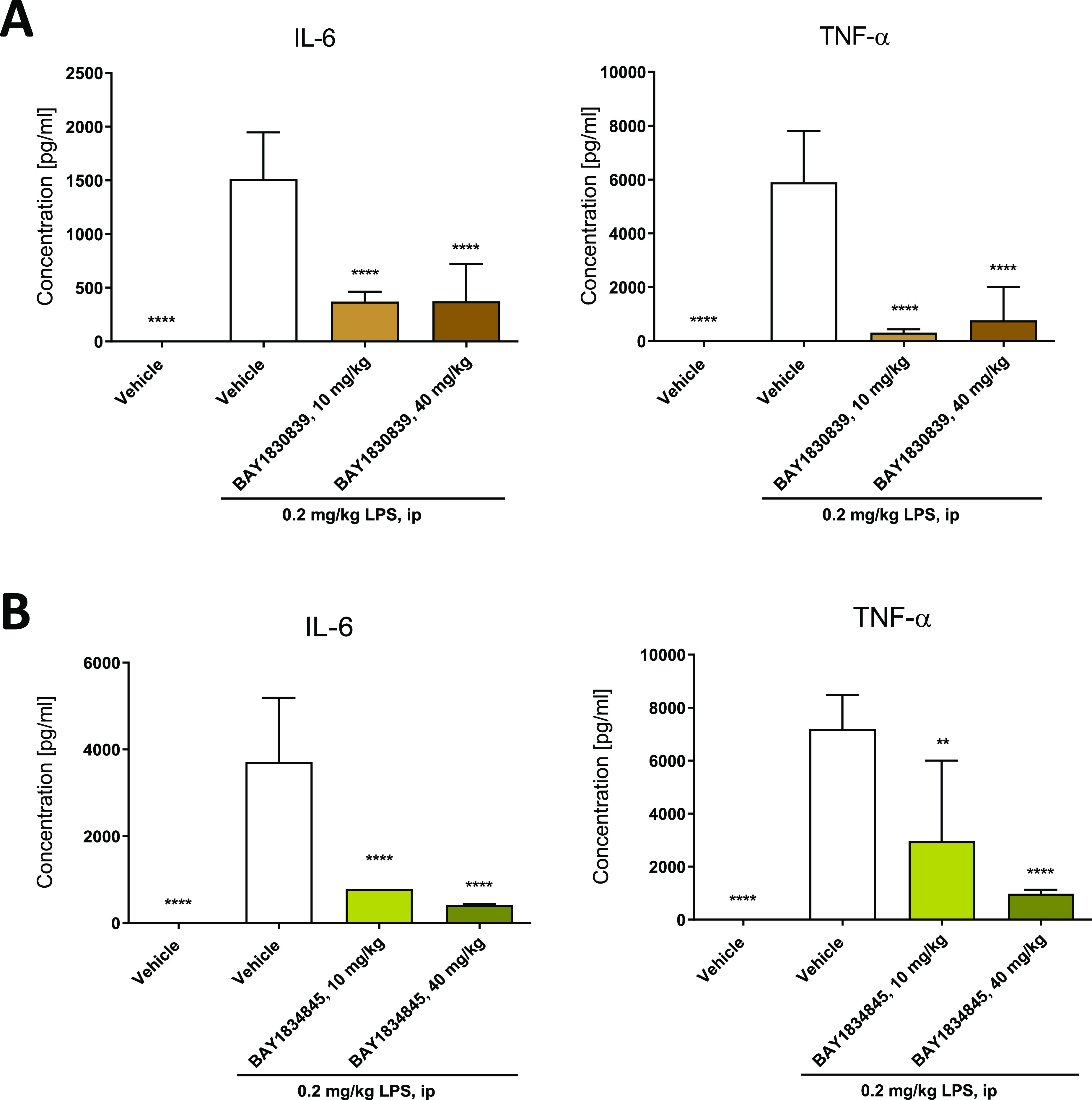
**BAY1830839** (A) and **BAY1834845** (B) inhibited LPS-induced inflammation in mice in vivo. Plasma levels of TNF-alpha and IL-6 were determined using multiplex protein assays 1.5 h after intraperitoneal injection of LPS. Mice were treated orally with either vehicle, **BAY1830839** (10 and 40 mg/kg) or **BAY1834845** (10 and 40 mg/kg) 4 h before the induction of inflammation. Healthy controls were treated with vehicle. Data are shown as mean ± SD for each treatment group (*n* = 5); one-way analysis of variance (ANOVA) versus LPS vehicle: *p < 0.05, **p < 0.01, ***p < 0.001, and ****p < 0.0001.

The efficacy of **BAY1830839** and **BAY1834845** was also investigated by using TLR-driven PD models. Plasma IL-6 and TNF-alpha were elevated within 1.5 h after intraperitoneal injection of the TLR4 ligand LPS in mice. The elevation of both IL-6 and TNF-alpha levels was significantly attenuated by the administration of **BAY1830839** and **BAY1834845** (Figure 9; for the corresponding unbound compound exposure data; see the Supporting Information, Figure S2).

**Figure 10:**
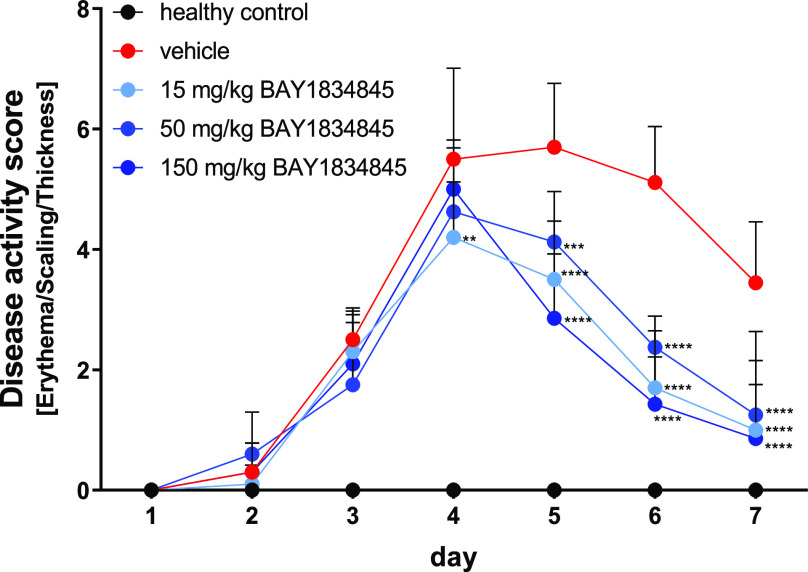
**BAY1834845** reduced imiquimod-induced psoriasis-like inflammation in mice measured using a modified disease activity score based on the severity of erythema, scaling, and skin thickening (Table S5). Disease scores were measured daily after topical administration of imiquimod to mice receiving twice daily oral treatment with **BAY1834845** (15, 50, or 150 mg/kg). The healthy control group (healthy control) and imiquimod disease control group (vehicle) were treated with vehicle only. Data are shown as mean ± SD for each treatment group (*n* = 10). One-way ANOVA with Dunnett’s test, **p < 0.01; ***p < 0.005; and ****p < 0.001 compared with imiquimod-induced disease control group.

Repeated topical administration of imiquimod, a TLR7/8 ligand, for 7 days induced a psoriasis-like phenotype in mice characterized by epidermal thickening, altered keratinocyte differentiation, neoangiogenesis, skin infiltration by immune cells, and the development of skin erythema.^49^ The severity of the psoriasis-like phenotype was assessed daily after disease induction using a modified disease scoring system developed by van der Fits et al.^50^ which encompasses erythema, scaling, and skin thickening (Figure 10). Relative to the vehicle-treated disease group, mice treated once daily with 100 mg/kg **BAY1830839** for 7 days showed significant reductions in disease score accompanied by significant reductions in the levels of inflammatory cytokines (such as TNF-alpha, IL-1β, and IL-23) in the imiquimod-challenged skin on day 7 (data not shown). Twice-daily oral administration of **BAY1834845** resulted in significant reductions in disease score when compared with imiquimod-challenged mice treated with the vehicle (Figure 10). In summary, **BAY1830839** and **BAY1834845** showed significant inhibition of IL-1β-induced and TLR-induced inflammation in vivo.

## Conclusions

In summary, our study identified two IRAK4 inhibitors, **BAY1830839** and **BAY1834845**, exhibiting high potency and a unique combination of high selectivity and excellent oral PK profiles across preclinical species. Key selectivity factors comprised a single interaction at the hinge; steric bulk under the nucleotide binding loop; and not deducible from kinase domain crystal structures, substituents pointing to the front pocket. Moreover, a markedly improved method for the selective *N*-2-alkylation of an indazole-based starting material has been developed.

Both clinical compounds showed significant anti-inflammatory in vivo efficacy in relevant pharmacodynamic inflammation models. Both **BAY1834845** (zabedosertib) and **BAY1830839** have advanced to clinical trials, the results of which will be reported in due course.

## Experimental Section

### Chemistry

#### General Methods and Materials

Commercially available reagents and anhydrous solvents were used as supplied without further purification. A Biotage Initiator Classic microwave reactor (Uppsala, Sweden) was used for reactions conducted in a microwave oven. Reactions were monitored by thin-layer chromatography (TLC) and ultra performance liquid chromatography (UPLC) using either analytical method A (Instrument: Waters Acquity UPLCMS SingleQuad; Column: Acquity UPLC BEH C18 1.7 μm, 50 mm × 2.1 mm; eluent A: water + 0.1 vol % formic acid (99%), eluent B: acetonitrile; gradient: 0–1.6 min 1–99% B, 1.6–2.0 min 99% B; flow 0.8 mL/min; temperature: 60 °C; DAD scan: 210–400 nm), analytical method B (Instrument: Waters Acquity UPLCMS SingleQuad; Column: Acquity UPLC BEH C18 1.7 μm, 50 × 2.1 mm; eluent A: water + 0.2 vol % aqueous ammonia (32%), eluent B: acetonitrile; gradient: 0–1.6 min 1–99% B, 1.6–2.0 min 99% B; flow 0.8 mL/min; temperature: 60 °C; DAD scan: 210–400 nm), analytical method C (Instrument: Waters Acquity UPLCMS SingleQuad; Column: Acquity UPLC BEH C18 1.7 50 mm × 2.1 mm; eluent A: water + 0.1 vol % formic acid (99%), eluent B: acetonitrile; gradient: 0–1.6 min 1–99% B, 1.6–2.0 min 99% B; flow 0.8 mL/min; temperature: 60 °C; DAD scan: 210–400 nm), or analytical method D (Instrument: Waters Acquity UPLCMS SingleQuad; Column: Acquity UPLC BEH C18 1.7 50 mm × 2.1 mm; eluent A: water + 0.2 vol % aqueous ammonia (32%), eluent B: acetonitrile; gradient: 0–1.6 min 1–99% B, 1.6–2.0 min 99% B; flow 0.8 mL/min; temperature: 60 °C; DAD scan: 210–400 nm) analytical TLC was carried out on aluminum-backed plates coated with Merck Kieselgel 60 F254, with visualization under UV light at 254 nm. Flash chromatography was carried out using a Biotage Isolera One system with a 200–400 nm variable detector. Preparative high performance liquid chromatography (HPLC) was carried out with a Waters AutoPurification MS Single Quad system; column: Waters XBridge C18 5 μm, 100 mm × 30 mm; basic conditions: eluent A, water + 0.2 vol % aq ammonia (32%); eluent B, MeCN; acidic conditions: eluent A: water + 0.1 vol % formic acid, eluent B: MeCN; DAD scan, 210–400 nm. Nuclear magnetic resonance (NMR) spectra were recorded at rt (22 ± 1 °C), unless otherwise noted, on Bruker Avance III HD spectrometers. ^1^H NMR spectra were obtained at 300, 400, 500, or 600 MHz. ^1^H NMR data are reported as follows: chemical shift (δ) in ppm, multiplicity (s = singlet, d = doublet, t = triplet, q = quartet, br = broad, m = multiplet), integration, and assignment. Low-resolution mass spectra (electrospray ionization, ESI) were obtained via HPLC-MS (ESI) using a Waters Acquity UPLC system equipped with an SQ 3100 mass detector. The purity of all target compounds, if not otherwise mentioned, was at least 95%, as determined by UPLC-MS. Compound names were generated using ICS software.

#### Synthesis of Clinical Compounds **BAY1830839** and **BAY1834845**

(a) Synthesis of intermediate *N*-[6-(2-hydroxypropan-2-yl)-1*H*-indazol-5-yl]-6-(trifluoromethyl)pyridine-2-carboxamide **VI** according to Scheme 1.

##### Methyl 5-Nitro-1*H*-indazole-6-carboxylate **II**

4.60 g (26.1 mmol) portion of methyl 1*H*-indazole-6-carboxylate **I** (CAS number 170487-40-8) was dissolved in 120 mL of sulfuric acid (96%) and cooled to −15 °C in a three-neck flask having a CPG stirrer, dropping funnel, and internal thermometer. Over a period of 15 min, the nitrating acid (10 mL of 96% sulfuric acid in 5 mL of 65% nitric acid), which had been prepared and cooled beforehand, was added dropwise to this solution. After the dropwise addition had ended, the mixture was stirred for a further 1 h (internal temperature at −13 °C). The reaction mixture was added to ice, and the precipitate was filtered off with suction, washed with water and dried in a drying cabinet at 50 °C under reduced pressure. 5.49 g (91% yield) of the title compound was obtained.

MS (ESIpos): *m*/*z* 222(M + H)^+^

1H NMR (400 MHz, DMSO-*d*_6_): δ (ppm) 3.87 (s, 3H), 7.96 (s, 1H), 8.44 (s, 1H), 8.70 (s, 1H), 13.98 (br s, 1H).

##### Methyl 5-Amino-1*H*-indazole-6-carboxylate **III**

4.40 g (19.8 mmol) portion of methyl 5-nitro-1*H*-indazole-6-carboxylate was dissolved in 236 mL of methanol and hydrogenated with 1.06 g (0.99 mmol) of palladium on activated carbon under hydrogen atmosphere at 25 °C for 3 h. The reaction mixture was filtered through Celite, the filter was washed with methanol, and the filtrate was concentrated. 3.53 g (88% yield) of the title compound was obtained.

1H NMR (300 MHz, DMSO-*d*_6_): δ (ppm) 3.85 (s, 3H) 6.01 (s, 2H) 6.98 (s, 1H) 7.79–7.91 (m, 1H) 7.99 (s, 1H) 12.84 (br s., 1H).

##### Methyl 5-({[6-(Trifluoromethyl)pyridin-2-yl]carbonyl}amino)-1*H*-indazole-6-carboxylate **V**

4.95 g portion (25.9 mmol) of 6-(trifluoromethyl)pyridine-2-carboxylic acid **IV** was initially charged in 45 mL of tetrahydrofuran (THF). 9.07 g (28.2 mmol) of *O*-(benzotriazol-1-yl)-*N*,*N*,*N*′,*N*′-tetramethyluronium tetrafluoroborate (TBTU) and 4.92 mL (28.2 mmol) of *N*-ethyl-*N*-isopropylpropan-2-amine (DIPEA) was added, and the mixture was stirred at 25 °C for 30 min. Subsequently, 4.50 g (23.5 mmol) of methyl 5-amino-1*H*-indazole-6-carboxylate was added and the mixture was stirred at 25 °C for 24 h. The reaction mixture was filtered with suction through a membrane filter, and the solids were washed with THF and with water and dried in a drying cabinet overnight. 7.60 g (78% yield) of the title compound was obtained.

MS (ESIpos): *m*/*z* 365 (M + H)^+^

1H NMR (400 MHz, DMSO-*d*_6_): δ (ppm) 3.97 (s, 3H), 8.13–8.27 (m, 2H), 8.30 (s, 1H), 8.33–8.45 (m, 1H), 8.45–8.51 (m, 1H), 9.15 (s, 1H), 12.57 (s, 1H), 13.44 (s, 1H).

##### *N*-[6-(2-Hydroxypropan-2-yl)-1*H*-indazol-5-yl]-6-(trifluoromethyl)pyridine-2-carboxamide **VI**

To a solution, cooled in an ice–water cooling bath, of 1.50 g (4.12 mmol) of methyl 5-({[6-(trifluoromethyl)pyridin-2-yl]carbonyl}amino)-1*H*-indazole-6-carboxylate **V** in 20 mL of THF was cautiously added 6.9 mL (5 equiv) of a 3 M methylmagnesium bromide solution in diethyl ether. The mixture was stirred while cooling with an ice bath for 1 h and at room temperature for 19.5 h. Another 2 equiv of methylmagnesium bromide solution was added and the mixture was stirred at room temperature for a further 24 h. Saturated aqueous ammonium chloride solution was added, and the mixture was stirred and extracted three times with ethyl acetate. The combined organic phases were washed with sodium chloride solution, filtered through a hydrophobic filter and concentrated. The residue was purified by column chromatography on silica gel (hexane/ethyl acetate). 763 mg (45% yield) of the title compound was obtained.

1H NMR (400 MHz, DMSO-*d*_6_): δ [ppm] 1.63 (s, 6H), 5.99 (s, 1H), 7.49 (s, 1H), 8.06 (s, 1H), 8.14–8.19 (m, 1H), 8.37 (t, *J* = 7.9 Hz, 1H), 8.46 (d, *J* = 7.8 Hz, 1H), 8.78 (s, 1H), 12.32 (s, 1H), 12.97 (s, 1H).

(b) Synthesis of **BAY1830839** and **BAY1834845** by improved alkylation procedures according to Scheme 2.

##### *N*-[2-(3-Hydroxy-3-methylbutyl)-6-(2-hydroxypropan-2-yl)-2*H*-indazol-5-yl]-6-(trifluoromethyl)pyridine-2-carboxamide **BAY1830839**

Step a: 3-Methylbutane-1,3-diol (63 g, 0.604 mol) was mixed with toluene (166 g), and the mixture was cooled down to 0 °C. Triethylamine (67 g, 0.66 mol) was then added to the mixture, followed by *N*,*N*-dimethylaminopyridine (3.7 g, 0.030 mol). In a separate vessel, *para*-toluenesulfonyl chloride (109 g, 0.571 mol) was dissolved in toluene (175 g) at 25 °C (endothermic). The resulting solution with fine particles was filtered and added to the solution containing the 3-methylbutane-1,3-diol at 0 °C over the course of 5 h. The resulting suspension was stirred at 0 °C for 15 h before filtering off the triethylammonium chloride salts. The filter cake was washed three times with 75 g toluene. The resulting solution was used as is for the alkylation step.

Step b: Alkylation Reaction: *N*-[6-(2-Hydroxypropan-2-yl)-1*H*-indazol-5-yl]-6-(trifluoromethyl)pyridine-2-carboxamide (50 g, 0.137 mol) **VI** was dissolved in toluene (288 g). *N*,*N*-Diisopropylethylamine (85 g, 0.659 mol) was then added to the mixture. Within 30 min, the jacket temperature was increased to 130 °C. The 3-hydroxy-3-methylbutyl 4-methylbenzenesulfonate **IX** solution prepared above was then completely added to the mixture regularly over the course of 10 h. The resulting mixture was then further stirred at reflux for an additional 16 h before being cooled down to 20 °C. The volatiles were then distilled off at a pressure of 70 mbar and a jacket temperature of 60 °C down to about 380 mL. The vacuum was then released while keeping the jacket temperature at 60 °C. Butyl acetate (500 mL) was then added to the mixture, followed by a mixture of acetic acid (55 g, 0.919 mol) in water (500 mL). The biphasic mixture was then stirred for 1 h before separating the phases. The aqueous phase was then extracted with 500 mL of *n*-butyl acetate. The organic phases were then combined, and 500 mL of a half-saturated sodium bicarbonate solution was added to the mixture. It was then allowed to stir at 60 °C for 1 h. The phases were separated, and the organic phase was washed with water (300 mL). The aqueous phase was discarded, and the organic phase was distilled down to 300 mL with a jacket temperature of 70 °C and a pressure of 200 mbar. 200 mL of butyl acetate was added, and the mixture was distilled back to 300 mL. *n*-Butyl acetate (200 mL) was added once more, and the mixture was distilled down to 300 mL again. The mixture was placed under nitrogen at ambient pressure and heated to 90 °C. It was then cooled to 83 °C within 1 h. The mixture was stirred at that temperature for a further hour and seeded with 220 mg product. It was then cooled to 60 °C within 2 h and stirred at that temperature for 30 min. It was then heated back to 78 °C and stirred for 30 min at this temperature. Finally, it was cooled down to 20 °C within 6 h and stirred overnight at that temperature. The crystals obtained were filtered and washed with 40 mL of *n*-butyl acetate twice. After 16 h drying at 50 °C under 20 mbar pressure, 40 g **BAY1830839** was isolated (64% yield).

1H NMR (DMSO-*d*_6_, 600 MHz): δ 12.40 (s, 1H), 8.76 (s, 1H), 8.48 (d, *J* = 7.8 Hz), 8.4–8.3 (m, 2H), 8.16 (d, 1H, *J* = 7.8 Hz), 7.61 (s, 1H), 5.98 (s, 1H), 4.56 (s, 1H), 4.5–4.5 (m, 2H), 2.0–2.1 (m, 2H), 1.66 (s, 6H), 1.18 (s, 6H).

##### *N*-{6-(2-Hydroxypropan-2-yl)-2-[2-(methylsulfonyl)ethyl]-2*H*-indazol-5-yl}-6-(trifluoromethyl)pyridine-2-carboxamide **BAY1834845**

*N*-[6-(2-Hydroxypropan-2-yl)-1*H*-indazol-5-yl]-6-(trifluoromethyl)pyridine-2-carboxamide **VI** (15 g, 41.2 mmol) was suspended in 225 mL toluene. The mixture was brought to reflux, and 75 mL toluene was distilled off in order to dry the reaction mixture. Diisopropylethylamine (259 μL, 2.06 mmol) was added, and the mixture was stirred for 15 min. Methyl vinyl sulfone **X** (7.0 g, 65.9 mmol) was added, and the mixture was refluxed for 96 h. Some toluene (45 mL) was then distilled off and a suspension was obtained. The mixture was then cooled down to 60 °C within 30 min. Methyl *tert*-butyl ether (100 mL) was added at this temperature within 30 min. The mixture was then cooled down to 20 °C within 2 h and stirred for a further 2 h. The mixture was then cooled to 3 °C within 2 h and stirred for 2 more hours. The solid was filtered off and washed twice with 15 mL cold methyl *tert*-butyl ether resulting in 14.7 g of wet material. This was then dissolved in acetone (294 g) to obtain a slightly turbid solution, which was immediately filtered. The clear filtrate was then added within 30 min to boiling ethanol (236 g). A total of 330 g of solvent was distilled off during this operation. Ethanol (118 g) was then added and distilled off twice. The temperature at the end of this process should be the boiling point of ethanol (79 °C). The mixture was then cooled down to 22 °C within 3 h and stirred for 2 h at 22 °C. It was then cooled further down to 2 °C within 1 h and stirred for another 2 h. The suspension was filtered, and the solid was washed twice with 12 g ethanol. It was then dried for 12 h at 30 mbar and 70 °C under a stream of nitrogen. 12.5 g (66% yield) of **BAY1834845** was isolated.

^1^H NMR (DMSO-*d*_6_, 600 MHz): δ 12.37 (s, 1H), 8.74 (s, 1H), 8.5–8.4 (m, 2H), 8.37 (t, 1H, *J* = 7.8 Hz), 8.16 (d, 1H, *J* = 7.7 Hz), 7.59 (s, 1H), 5.98 (s, 1H), 4.86 (t, 2H, *J* = 6.8 Hz), 3.86 (t, 2H, *J* = 6.8 Hz), 2.91 (s, 3H), 1.63 (s, 6H).

## Supporting Information Available

Molecular formula strings (CSV)
Drug metabolism and pharmacokinetics (DMPK); in vitro pharmacology; X-ray crystallography; computational studies; experimental section synthetic chemistry; original analytical data of test compounds; physicochemical assays (PDF)

## Accession Codes

The coordinates and structure factors for the described crystal structures have been deposited with the Protein Data Bank (PDB). The PDB accession codes are 8BR7 (compound **5**), 8ATB (compound **16**), 8ATL (compound **23**), 8ATN (compound **38**), 8BR6 (compound **40**), and 8BR5 (compound **41**). Authors will release the atomic coordinates upon article publication.

## Abbreviations

ADMET: absorption, distribution, metabolism, excretion, and toxicity
AUC: area under the curve
CL_blood_: hepatic in vivo blood clearance
*C*_max_: peak serum concentration
COX: cyclooxygenase
DAMPs: damage-associated molecular patterns
DDR1: discoidin domain receptor tyrosine kinase 1
DIPEA: *N*,*N*-diisopropylethylamine
FEP: free energy perturbation
FLT3: fms-like receptor tyrosine kinase 3
*F*_max_: maximal oral bioavailability
HMGB1: high-mobility group box 1 protein
^1^H NMR: proton nuclear magnetic resonance
HPLC: high performance liquid chromatography
HSP: heat shock protein
HTS: high throughput screening
IC_50_: half-maximum inhibitory concentration
IKK: inhibitor of NF-κB kinase
IL: interleukin
IL-1R: interleukin-1 receptor
IRAK4: interleukin-1 receptor-associated kinase 4
LPS: lipopolysaccharides
MAPK: mitogen-activated protein kinase
MKK: MAPK kinase
mRNA: messenger ribonucleic acid
MSM: Markov state model
MyD88: myeloid differentiation primary response protein 88
NBL: nucleotide binding loop
NF-κB: nuclear factor kappa-light-chain-enhancer of activated B cells
oLDL: oxidized low-density lipoprotein
PAMPs: pathogen-associated molecular patterns
*P*_app_: apparent permeability coefficient
PD: pharmacodynamic
PK: pharmacokinetic
SAR: structure–activity relationship
ssRNA: single-strand ribonucleic acid
*t*_1/2_: half-life
TLC: thin-layer chromatography
TLR: toll-like receptor
*t*_max_: time to peak drug concentration
TNF: tumor necrosis factor
TRAF: tumor necrosis factor receptor associated factor
Trk: tropomyosin receptor kinase
UPLC: ultra performance liquid chromatography
UV: ultraviolet
*V*_ss_: steady-state volume of distribution
