# Supplementary material for: Discovery of IRAK4 Inhibitors BAY1834845 (Zabedosertib) and BAY1830839
Source: J Med Chem. 2024 Jan 17;67(2):1225–42. doi: 10.1021/acs.jmedchem.3c01714 (PMC10823478; doi:10.1021/acs.jmedchem.3c01714)
Supplement: Supplementary file 2 — jm3c01714_si_002.pdf [file jm3c01714_si_002.pdf]

Supporting Information

Discovery of IRAK4 Inhibitors BAY1834845 (zabedosertib) and BAY1830839

Ulrich Bothe[a*], Judith Günther[a], Reinhard Nubbemeyer[a#], Holger Siebeneicher[a], Sven Ring[a], Ulf Bömer[a#], Michaele Peters[a], Alexandra Rausch[a#], Karsten Denner[a], Herbert Himmel[a], Andreas Sutter[a], Ildiko Terebesi[a], Martin Lange[a#], Antje M. Wengner[a], Nicolas Guimond[a], Tobias Thaler[a], Johannes Platzek[a], Uwe Eberspächer[a], Martina Schäfer[a#], Holger Steuber[a#], Thomas M. Zollner[a], Andreas Steinmeyer[a], and Nicole Schmidt[a]

[a]Bayer AG, Research & Development, Pharmaceuticals, 13353 Berlin, Germany

Corresponding author:
*Email: Ulrich.bothe@bayer.com

Present addresses
[#]R.N., U.Bö., A.R., M.L., M.S., H.St.: Nuvisan ICB GmbH, 13353 Berlin, Germany.

Contents

Drug metabolism and pharmacokinetics (DMPK) 10
Caco-2 cell permeability assay 10
In vitro metabolic stability in rat hepatocytes 10
In vitro metabolic stability in human, dog, and primate hepatocytes 11
Inhibition of CYP450 metabolism 12
CYP induction 12
In vivo studies general information 12
Pharmacokinetics in rats 12
Pharmacokinetics in mice 13
Pharmacokinetics in beagle dogs 13
In vitro pharmacology 14
Kinase assays 14
Table S1. Summary of kinase assays. 15
DiscoveRx KINOMEscan™ data 17
Table S2. Matrix of compound screen for BAY1830839. 18
Table S3. Matrix of Compound Screen for BAY1834845. 23
Table S4. Matrix of compound screen for compound 5 28
Murine cellular potency 31
In vivo pharmacology 32
IL-1-beta induced systemic inflammation in mice 32
Figure S1 32
LPS (Lipopolysaccharides) induced systemic inflammation in mice 33
Figure S2: 33
Imiquimod-induced topical inflammation in mice 33
Table S5. Disease scoring method for assessing back skin in mice. 34
X-ray crystallography 34
Expression and purification of IRAK4 for X-ray crystallography 34
Crystallization and complex formation 35
Data collection and refinement for IRAK4 co-complex structures: 35

Table S6. Data collection and refinement statistics. 37
Figure S3: 38
Computational studies 39
Figure S4: 39
Experimental section: synthetic chemistry 40
General Methods and Materials 40
Abbreviations 41
Synthesis of compound 6 42
Intermediate 6-a 42
Intermediate 6-b 42
Intermediate 6-c 42
Intermediate 6-d 43
Intermediate 6-e 43
Compound 6 (step 6-f) 44
Synthesis of compound 7 44
Intermediate 7-a 44
Intermediate 7-b 45
Intermediate 7-c 46
Intermediate 7-d 46
Intermediate 7-e 47
Compound 7 (step 7-f) 47
Synthesis of compound 8 48
Intermediate 8-a 48
Intermediate 8-b 48
Intermediate 8-c 49
Intermediate 8-d 49
Intermediate 8-e 50
Compound 8 (step 8-f) 50
Synthesis of compound 9 51

Intermediate 9-a 51
Intermediate 9-b 51
Intermediate 9-c 52
Intermediate 9-d 53
Intermediate 9-e 53
Compound 9 (step 9-f) 54
Synthesis of compound 10 54
Intermediate 10-a 54
Intermediate 10-b 55
Intermediate 10-c 56
Compound 10 (step 10-d) 56
Synthesis of compound 11 57
Intermediate 11-a 57
Intermediate 11-b 58
Intermediate 11-c 58
Intermediate 11-d 59
Intermediate 11-e 59
Intermediate 11-f 60
Compound 11 (step 11-g) 60
Synthesis of compound 12 61
Compound 12 (step 12-a) 61
Synthesis of compound 13 62
Intermediate 13-a 62
Intermediate 13-b 62
Intermediate 13-c 63
Intermediate 13-d 63
Intermediate 13-e 64
Intermediate 13-f 64
Intermediate 13-g 64

Compound 13 (step 13-h)....65
Synthesis of compound 14....66
Intermediate 14-a....66
Intermediate 14-b....66
Intermediate 14-c....67
Intermediate 14-d....68
Synthesis of compound 15....68
Intermediate 15-a....68
Intermediate 15-b....69
Intermediate 15-c....69
Intermediate 15-d....70
Intermediate 15-e....71
Intermediate 15-f....71
Intermediate 15-g....72
Compound 15 (step 15-h)....72
Synthesis of compound 16....73
Compound 16 (step 16-a)....73
Synthesis of compound 17....73
Compound 17 (step 17-a)....73
Synthesis of compound 18....74
Intermediate 18-a....74
Intermediate 18-b....74
Intermediate 18-c....75
Intermediate 18-d....76
Intermediate 18-e....76
Intermediate 18-f....77
Intermediate 18-g....78
Intermediate 18-h....78
Intermediate 18-i....79

Compound 18 (step 18-j) 79
Synthesis of compound 19 80
Compound 19 (step 19-a) 80
Synthesis of compound 20 81
Compound 20 (step 20-a) 81
Synthesis of compound 21 81
Intermediate 21-a 81
Intermediate 21-b 82
Intermediate 21-c 83
Compound 21 (step 21-d) 83
Synthesis of compound 22 84
Intermediate 22-a 84
Intermediate 22-b 84
Intermediate 22-c 85
Compound 22 (step 22-d) 85
Synthesis of compound 23 and compound 24 86
Intermediate 23-a 86
Intermediate 23-b 86
Intermediate 23-c 87
Intermediate 23-d 87
Intermediate 23-e 88
Table S7. HPLC methodology. 89
Compound 23 and Compound 24 (step 23/24-f) 89
Table S8. Chiral HPLC methodology. 90
Table S9. Chiral HPLC methodology (Preparation). 91
Synthesis of compound 25 91
Compound 25 (step 25-a) 91
Synthesis of compound 26 92
Intermediate 26-a 92

Intermediate 26-b....93
Compound 26 (step 26-c)....93
Synthesis of compound 27....94
Compound 27 (step 27-a)....94
Synthesis of compound 28....95
Compound 28 (step 28-a)....95
Synthesis of compound 29....95
Intermediate 29-a....95
Intermediate 29-b....96
Intermediate 29-c....97
Compound 29 (step 29-d)....97
Synthesis of compound 30....98
Compound 30 (step 30-a)....98
Synthesis of compound 31....98
Compound 31 (step 31-a)....98
Synthesis of compound 32....99
Intermediate 32-a....99
Intermediate 32-b....100
Intermediate 32-c....100
Compound 32 (step 32-d)....101
Synthesis of compound 33....101
Intermediate 33-a....101
Compound 33 (step 33-b)....102
Synthesis of compound 34....103
Compound 34 (step 34-a)....103
Synthesis of compound 35....104
Intermediate 35-a....104
Intermediate 35-b....104
Intermediate 35-c....104

Intermediate 35-d 105
Intermediate 35-e 106
Intermediate 35-f 106
Compound 35 (step 35-g) 107
Synthesis of compound 36 108
Intermediate 36-a 108
Intermediate 36-b 109
Intermediate 36-c 109
Intermediate 36-d 110
Intermediate 36-e 110
Intermediate 36-f 111
Intermediate 36-g 112
Compound 36 (step 36-h) 112
Synthesis of compound 37 113
Intermediate 37-a 113
Intermediate 37-b 114
Intermediate 37-c 114
Intermediate 37-d 115
Intermediate 37-e 115
Compound 37 (step 37-f) 116
Synthesis of compound 38 116
Intermediate 38-a 116
Intermediate 38-b 117
Intermediate 38-c 117
Intermediate 38-d 118
Intermediate 38-e 119
Compound 38 (step 38-f) 119
Synthesis of compound 39 121
Intermediate 39-a 121

Intermediate 39-b 121
Intermediate 39-c 122
Intermediate 39-d 122
Intermediate 39-e 123
Compound 39 (step 39-f) 124
Synthesis of compound 40 125
Intermediate 40-a 125
Intermediate 40-b 125
Compound 40 (step 40-c) 126
Synthesis of compound 41 127
Intermediate 41-a 127
Intermediate 41-b 127
Intermediate 41-c 128
Compound 41 (step 41-d) 128
Figure S5. Original analytical data of test compounds 129
Experimental section: physicochemical assays 143
Aqueous Solubility of Compound from DMSO solutions 143
References 143

Drug metabolism and pharmacokinetics (DMPK)

Caco-2 cell permeability assay

Caco-2 cells (DSMZ, Braunschweig, Germany) were seeded at a density of 4.5 × 10^4 cells/well on 24-well microtiter plates with a 0.4 μm pore size and grown for 15 d in Dulbecco's Modified Eagle Medium (DMEM) supplemented with 10% FCS (Gibco, Thermo Fisher Scientific, Waltham, MA USA), 1% GlutaMAX (100 ×, Gibco), 100 U/mL penicillin, 100 μg/mL streptomycin (Gibco), and 1% non-essential amino acids (100 ×). Cells were maintained at 37 °C in a humidified 5% CO_2 atmosphere. Medium was changed every 2–3 d. Before the permeation assay was run, the culture medium was replaced by FCS-free *N*-2-hydroxyethylpiperazine-*N'*-2-ethanesulfonic acid (HEPES) carbonate transport buffer (pH 7.2). The transepithelial electrical resistance was measured to assess monolayer integrity. Test compounds were predissolved in DMSO and added to either the apical or basolateral compartment at a final concentration of 2 μM. Samples were taken from both compartments before and after incubation for 2 h at 37 °C and analyzed by LC-MS/MS after precipitation with MeOH. Permeability (Papp) was calculated in the apical to basolateral (A→B) and basolateral to apical (B→A) directions. Papp was calculated using the equation Papp = (Vr/P0)(1/S)(P2/t), where Vr is the volume of medium in the receiver chamber, P0 is the measured peak area of the test drug in the donor chamber at t = 0, S is the surface area of the monolayer, P2 is the measured peak area of the test drug in the acceptor chamber after a two-hour incubation, and t is the incubation time. The efflux ratio (ER) of basolateral (B) to apical (A) was calculated as Papp B→A/Papp A→B. Compound recovery was also calculated. As an assay control, reference compounds were analyzed in parallel. Permeability ratings were based on the subsequent ranges: low permeability: < 10 nm/s, moderate permeability: 10–70 nm/s, and high permeability: > 70 nm/s (a-b). ER values < 2.0 were interpreted as no significant efflux.

***In vitro* metabolic stability in rat hepatocytes**

Hepatocytes from Han–Wistar rats (Harlan, Laboratories, Horst, The Netherlands) were isolated via a two-intermediate perfusion method. After perfusion, the liver was carefully removed from the rat, the liver capsule was opened, and the hepatocytes were gently shaken out into a Petri dish with ice-cold Williams' medium E (WME, Gibco). The resulting cell suspension was then filtered through sterile gauze into 50 mL Falcon tubes (Corning, US) and centrifuged at 50 × g for 3 min at rt. The cell pellet was resuspended in 30 mL of WME and centrifuged through a Percoll gradient twice at 100 × g. The hepatocytes were washed

again with WME and resuspended in medium containing 5% FCS. Cell viability was determined by trypan blue exclusion. For the metabolic stability assay, liver cells were distributed in WME containing 5% FCS and transferred to glass vials at a density of 1.0 × 10^6 vital cells/mL. The test compound was added at a final concentration of 1 μM. During incubation, the hepatocyte suspensions were continuously shaken at 580 rpm and aliquots were taken at 2, 8, 16, 30, 45, and 90 min, to which an equal volume of cold MeOH was immediately added. Samples were frozen at –20 °C overnight and subsequently centrifuged for 15 min at 3000 rpm, after which the supernatant was analyzed with an Agilent 1200 HPLC system with LC-MS/MS detection (Agilent Technologies, Inc. Santa Clara, US). The $t_{1/2}$ of a test compound was determined from the concentration–time plot. The intrinsic clearance was calculated from the $t_{1/2}$ using the 'well-stirred' liver model together with the additional parameters of liver blood flow and number of liver cells *in vivo* and *in vitro*. The hepatic *in vivo* blood clearance (CL) and the maximal oral bioavailability (F_{max}) were calculated. The following parameter values were used: liver blood flow 4.2 L/h/kg rat; specific liver weight 32 g/kg rat body weight; liver cells *in vivo* 1.1 × 10^8 cells/g liver; and liver cells *in vitro* 1.0 × 10^6/mL.

***In vitro* metabolic stability in human, dog, and primate hepatocytes**

Human cryopreserved hepatocytes were purchased from Ka-Ly-Cell (Plobsheim, France, batches EFF, FME, GGJ, XPD) and freshly prepared primate (cynomolgus macaque) and dog hepatocytes from Primacyt (Schwerin, Germany). For the metabolic stability assay, liver cells were distributed in WME containing 5% FCS and transferred to glass vials at a density of 1.0 × 10^6 vital cells/mL. The test compound was added at a final concentration of 1 μM. During incubation, the hepatocyte suspensions were continuously shaken at 580 rpm and aliquots were taken at 2, 8, 16, 30, 45, and 90 min, to which an equal volume of cold MeOH was immediately added. Samples were frozen at –20 °C overnight and subsequently centrifuged for 15 min at 3000 rpm, after which the supernatant was analyzed with an Agilent 1200 HPLC system with LC-MS/MS detection (Agilent Technologies, Inc., Santa Clara, US). The $t_{1/2}$ of a test compound was determined from the concentration–time plot. The intrinsic clearance was calculated from the $t_{1/2}$ using the 'well-stirred' liver model together with the additional parameters of liver blood flow and number of liver cells *in vivo* and *in vitro*. CL_{blood} and Fmax were calculated. The following parameter values were used: liver blood flow 4.2/2.6/2.1 L/h/kg (human/primate/dog); specific liver weight 21/30/39 g/kg body weight

(human/primate/dog); liver cells *in vivo* 1.1 × 10^8 cells/g liver (except in the dog 2.2 × 10^8 cells/g liver); liver cells *in vitro* 1.0 × 10^6/mL.

Inhibition of CYP450 metabolism

The inhibitory potency of test compounds towards CyP450-dependent metabolic pathways was determined in human liver microsomes (purchased from Xenotech, USA) by applying individual CYP isoform selective standard probes (CYP1A2: phenacetin; CYP2C8: amodiaquine; CYP2C9: diclofenac; CYP2D6: dextromethorphan; CYP3A4: midazolam). Reference inhibitors were included as positive controls. Incubation conditions (protein and substrate concentration, incubation time) were optimized according to the linearity of metabolite formation. Assays were processed in 96-well microtiter plates at 37 °C using a Genesis Workstation (Tecan, Crailsheim, Germany). After protein precipitation, metabolite formation was quantified by LC-MS/MS analysis, followed by inhibition evaluation and IC_{50} calculation.

CYP induction

To evaluate the CYP induction potential *in vitro*, hepatocytes in sandwich culture from three different liver donors were tested once daily for three consecutive days with vehicle control, eight different concentrations of a test substance, and known positive controls (e.g., omeprazole, phenobarbital, rifampicin). After treatment, the cells were incubated *in situ* with appropriate standard substrates for CYP3A4 and CYP1A2 and their activity was quantified using LC-MS/MS via the metabolites formed. Following *in situ* incubation with standard substrates, the same hepatocytes were harvested from the different treatment groups, the RNA was isolated, and the effect of the test substances on the CYP1A2, CYP3A4, and CYP2B6 mRNA expression levels was determined using qRT-PCR.

***In vivo* studies general information**

All animal studies were conducted in accordance with the German Animal Welfare Act and the French directives and the ethical guidelines of Bayer AG and were approved by the local ethics committee.

Pharmacokinetics in rats

Male Wistar rats were obtained from Harlan Laboratories (Horst, The Netherlands) and had access to food and water ad libitum. All animals were housed according to institutional guidelines under a 12 h/12 h light/dark cycle and maintained under standard conditions (20–22 °C, 50–70% humidity). Rats were housed in Makrolon cages type IV (4 animals/cage), fed

a pelleted standard maintenance diet (Ssniff, Spezialdiäten GmbH, Soest, Germany), and used for *in vivo* studies at a weight of 200–300 g.

For *in vivo* PK experiments, test compounds were administered to male Wistar rats iv at a dose of 0.5 mg/kg and po at a dose of 2.0 mg/kg, formulated as solutions using solubilizers such as PEG400, Solutol, and EtOH in well-tolerated amounts. Blood samples were collected from the vena jugularis at 2 min (iv only), 8 min, 15 min, 30 min, 45 min, 1 h, 2 h, 4 h, 7 h, 24 h, and 48 h (if needed) after dosing, saved in lithium heparin tubes (Monovette®, Sarstedt, Germany), and centrifuged for 15 min at 3000 rpm. An aliquot of 100 μL of the supernatant (plasma) was taken and precipitated by the addition of 400 μL cold acetonitrile. Samples were frozen at –20 °C overnight, and subsequently thawed and centrifuged at 3000 rpm and 4 °C for 20 min. Aliquots of the supernatant were analyzed with an Agilent HPLC system with LC-MS/MS detection (Agilent Technologies, Inc., Santa Clara, US). Pharmacokinetic parameters were calculated by non-compartmental analysis using pharmacokinetics calculation software (e.g., Phoenix WinNonlin 6.3, Certara USA, Inc.).

Pharmacokinetics in mice

Female NMRI mice were obtained from Charles River Laboratories (Sulzfeld, Germany) and had access to food and water ad libitum. All animals were housed according to institutional guidelines under a 12 h/12 h light/dark cycle and maintained under standard conditions (20–22 °C, 50–70% humidity). Mice were housed in Makrolon cages type IV (10 animals/cage), fed a pelleted diet (see above, Ssniff, Spezialdiäten GmbH, Soest, Germany), and used for in vivo studies at a weight of 20–30 g.

For *in vivo* PK experiments, test compounds were administered to female NMRI mice iv at a dose of 0.5 mg/kg, formulated as solutions using solubilizers such as PEG400 and EtOH in well-tolerated amounts. Blood samples were collected from the vena jugularis at 2 min, 8 min, 15 min, 30 min, 1 h, 2 h, 4 h, 7 h, and 24 h after dosing, saved in lithium heparin tubes (Monovette®, Sarstedt, Germany), and centrifuged for 15 min at 3000 rpm. An aliquot of 100 μL from the supernatant (plasma) was taken and precipitated by the addition of 400 μL cold acetonitrile. The next intermediates in processing the samples as well as the data evaluation are described above (see section 'Pharmacokinetics in Rats').

Pharmacokinetics in beagle dogs

All animals were housed according to institutional guidelines under a 12 h/12 h light/dark cycle and maintained under standard conditions. For *in vivo* pharmacokinetic experiments, test compounds were administered to female or male dogs iv for 15 min at a dose of 0.5

mg/kg and po at a dose of 2.0 mg/kg, formulated as solutions using solubilizers such as PEG400 and EtOH in well-tolerated amounts. Blood samples were collected from the Vena cephalica antebrachii at 5 min, 10 min, 15 min (prior end of infusion), 20 min, 30 min, 1 h, 2 h, 4 h, 7 h, 24 h, and 48 h (if needed) after iv infusion dosing and at 5 min, 15 min, 30 min, 1 h, 2 h, 4 h, 7 h, 24 h, and 48 h after po dosing (if needed). Samples were stored in lithium heparin tubes (Monovette, Sarstedt) and centrifuged for 15 min at 3000 rpm. An aliquot of 100 μL from the supernatant (plasma) was taken and precipitated by the addition of 400 μL cold acetonitrile. The next intermediates in processing the samples as well as the data evaluation are described above (see section 'Pharmacokinetics in Rats').

In vitro pharmacology

Kinase assays

The inhibitory activities of the compounds against IRAK4, FLT3, and TrkA were measured in TR-FRET-based kinase activity inhibition assays using purified recombinant proteins as enzymes and biotinylated peptides or a biotinylated poly-Glu,Tyr(4:1)-copolymer as substrate (Table S1). For the assays, 50 nL of a 100-fold concentrated solution of the test compound in DMSO was pipetted into a black, low-volume, 1536-well microtiter plate (Greiner Bio-One, Frickenhausen, Germany); 2 μL of enzyme solution in aqueous assay buffer (see below) was added; and the mixture was incubated for 15 min at 22 °C to allow prebinding of the test compound to the enzyme before the start of the kinase reaction. The kinase reaction was then started by the addition of 3 μL of a solution of ATP (final conc.: 10 μM [FLT3, Trk-A] or 1 mM [IRAK4]) and substrate to the assay buffer; the resulting mixture was incubated for a reaction time of 45 min (IRAK4, FLT3) or 60 min (TrkA) at 22 °C. The concentrations of the enzymes were adjusted depending on the activity of the enzyme lot; the choice of enzyme was based on which enzyme lot kept the assay in the linear range. Typical concentrations were in the range of 0.1–0.3 nM. The reaction was stopped by the addition of 5 μL of a solution of TR-FRET detection reagents in an aqueous EDTA (see below). The resulting mixture was incubated for 1 h at 22 °C to allow the formation of a complex between the phosphorylated biotinylated peptide and the detection reagents. Subsequently, the amount of phosphorylated substrate was evaluated by measuring the resonance energy transfer from the europium chelate to streptavidin-XL665. To do this, fluorescence emissions at 620 nm and 665 nm after excitation at 350 nm were measured with a PHERAstar FS reader (BMG Labtech, Offenburg, Germany). The ratio of the emissions at 665 nm and at 620 nm was considered the amount of phosphorylated substrate. The compounds were tested on the

same microtiter plate at 11 different concentrations in the range 20 µM to 0.07 nM (the dilution series was prepared separately, before the assay, using 100-fold concentrated solutions in DMSO by serial dilutions) in duplicate for each concentration. IC_{50} values were calculated using Genedata Screener™ software.

Table S1. Summary of kinase assays.

	IRAK4	FLT3	TrkA
Enzyme	Human: Recombinant fusion protein from N-terminal GST (glutathione S-transferase) and human IRAK4, expressed in baculovirus-infected insect cells (Hi5)<br>Rat, mouse, monkey, and dog: N-terminally His6-tagged recombinant IRAK4 (respective species), expressed in baculovirus-infected insect cells (Hi5)	Recombinant fusion protein from N-terminal GST and human FLT3 (aa 564-end (Merck Millipore # 14-500)	Recombinant fusion protein of N-terminally His6-tagged GST and a C-terminal fragment of human TrkA (aa 443-796, ProQinase # 0311-0000-2)
Assay buffer	50 mM HEPES pH 7.5, 5 mM $MgCl_2$, 1 mM DTT, 30 μM activated sodium *ortho*-vanadate, 0.1% (w/v) BGG,	25 mM HEPES pH 7.5, 10 mM $MgCl_2$, 5 mM glycerol-2-phosphate, 2 mM DTT, 0.5 mM	8 mM MOPS/HCl pH 7.0, 10 mM $MgCl_2$, 1 mM DTT, 0.2 mM EDTA, 0.01% (v/v) Nonidet-P40

	0.04% (v/v) Nonidet-P40 (Sigma-Aldrich, St. Louis, USA)	EDTA, 0.01% (v/v) Triton X-100 (Sigma-Alrich, St. Louis, USA)	
Substrate	Biotin-Ahx-KKARFSRFAGSSPSQASFAEPG (C-terminus in amide form), final conc. in enzyme reaction: 0.5 *μ*M	Biotin-Ahx-GGEEEEYFELVKKKK (C-terminus in amide form), final conc. in enzyme reaction: 1 *μ*M	Biotinylated poly-Glu,Tyr (4:1)-copolymer (CisBio # 61GT0BLA), final conc. in enzyme reaction: 1.36 *μ*g/mL
TR-FRET detection reagent solution	25 mM HEPES pH 7.5, 100 mM EDTA, 0.1 *μ*M streptavidin-XL665 (Cisbio Bioassays; # 610SAXLG), 1.5 nM anti-phosphoserine antibody (Merck Millipore, (Darmstadt, Germany), # 35-002), 0.6 nM LANCE EU-W1024-labelled anti-mouse-IgG antibody (Perkin-Elmer (Waltham, USA), # AD0077), 0.4 % (w/v) BSA	50 mM HEPES pH 7.5, 50 mM EDTA, 0.2 *μ*M streptavidin-XL665, 3 nM PT66-Eu-chelate (PerkinElmer, (Waltham, USA), # AD0069), 0.1 % (w/v) BSA	50 mM HEPES/HCl pH 7.0, 100 mM EDTA, 30 nM streptavidin-XL665, 1.4 nM PT66-Eu-chelate, 0.2 % (w/v) BSA

BGG, bovine gamma-globulin; BSA, bovine serum albumine; DTT, dithiothreitol; EDTA, ethylenediaminetetraacetic acid; GST, glutathione *S*-transferase; HEPES, 4-(2-

hydroxyethyl)-1-piperazineethanesulfonic acid; MOPS, 3-(*N*-morpholino)propanesulfonic acid.

DiscoveRx KINOMEscan^{TM} data

Kinase selectivity data was assessed using KINOMEscan^{TM} provided by DiscoveRx Corporation, 42501 Albrae Street, Fremont, CA 94538-3142:[1] BAY1830839 and BAY1834845 were tested using two concentrations (100 nM and 1000 nM)

Table S2. Matrix of compound screen for BAY1830839.

Target	BAY1830839	
Gene Symbol	%Ctrl @ 100nM	%Ctrl @ 1000nM
AAK1	74	51
ABL1(E255K)-phosphorylated	100	96
ABL1(F317I)-nonphosphorylated	100	100
ABL1(F317I)-phosphorylated	100	98
ABL1(F317L)-nonphosphorylated	100	95
ABL1(F317L)-phosphorylated	90	92
ABL1(H396P)-nonphosphorylated	100	100
ABL1(H396P)-phosphorylated	94	94
ABL1(M351T)-phosphorylated	92	94
ABL1(Q252H)-nonphosphorylated	100	94
ABL1(Q252H)-phosphorylated	99	100
ABL1(T315I)-nonphosphorylated	100	95
ABL1(T315I)-phosphorylated	92	84
ABL1(Y253F)-phosphorylated	100	100
ABL1-nonphosphorylated	100	95
ABL1-phosphorylated	100	100
ABL2	88	100
ACVR1	96	100
ACVR1B	94	97
ACVR2A	92	90
ACVR2B	98	83
ACVRL1	100	100
ADCK3	97	83
ADCK4	99	100
AKT1	87	100
AKT2	90	100
AKT3	92	98
ALK	100	91
ALK(C1156Y)	94	81
ALK(L1196M)	86	96
AMPK-alpha1	100	93
AMPK-alpha2	100	97
ANKK1	96	84
ARK5	100	79
ASK1	86	96
ASK2	100	100
AURKA	77	76
AURKB	91	89
AURKC	98	91
AXL	100	91
BIKE	93	38
BLK	82	66
BMPR1A	89	95
BMPR1B	82	85
BMPR2	92	83
BMX	91	76
BRAF	92	81
BRAF(V600E)	91	86

Target	BAY1830839	
Gene Symbol	%Ctrl @ 100nM	%Ctrl @ 1000nM
BRK	97	92
BRSK1	82	100
BRSK2	95	92
BTK	100	97
BUB1	97	96
CAMK1	83	100
CAMK1D	96	100
CAMK1G	80	65
CAMK2A	98	89
CAMK2B	100	83
CAMK2D	87	83
CAMK2G	95	79
CAMK4	100	100
CAMKK1	84	50
CAMKK2	80	39
CASK	100	100
CDC2L1	97	93
CDC2L2	98	98
CDC2L5	100	97
CDK11	88	100
CDK2	100	100
CDK3	94	100
CDK4-cyclinD1	98	88
CDK4-cyclinD3	100	100
CDK5	91	99
CDK7	96	91
CDK8	89	100
CDK9	86	82
CDKL1	90	77
CDKL2	94	100
CDKL3	89	96
CDKL5	99	81
CHEK1	95	100
CHEK2	83	77
CIT	100	95
CLK1	92	89
CLK2	100	54
CLK3	100	94
CLK4	100	86
CSF1R	88	87
CSF1R-autoinhibited	100	89
CSK	100	100
CSNK1A1	89	93
CSNK1A1L	95	100
CSNK1D	99	100
CSNK1E	99	66
CSNK1G1	92	77
CSNK1G2	100	100
CSNK1G3	96	98

Target	BAY1830839	
Gene Symbol	%Ctrl @ 100nM	%Ctrl @ 1000nM
CSNK2A1	90	76
CSNK2A2	98	96
CTK	100	100
DAPK1	95	90
DAPK2	100	89
DAPK3	100	87
DCAMKL1	88	80
DCAMKL2	97	92
DCAMKL3	100	100
DDR1	100	100
DDR2	92	94
DLK	98	98
DMPK	88	100
DMPK2	94	100
DRAK1	97	100
DRAK2	96	95
DYRK1A	97	78
DYRK1B	67	61
DYRK2	87	100
EGFR	78	84
EGFR(E746-A750del)	90	100
EGFR(G719C)	98	93
EGFR(G719S)	87	86
EGFR(L747-E749del, A750P)	75	83
EGFR(L747-S752del, P753S)	100	100
EGFR(L747-T751del,Sins)	90	88
EGFR(L858R)	84	100
EGFR(L858R,T790M)	91	76
EGFR(L861Q)	88	89
EGFR(S752-I759del)	97	92
EGFR(T790M)	94	95
EIF2AK1	98	80
EPHA1	100	100
EPHA2	97	78
EPHA3	75	81
EPHA4	100	91
EPHA5	92	95
EPHA6	100	90
EPHA7	91	100
EPHA8	84	100
EPHB1	85	100
EPHB2	62	64
EPHB3	100	94
EPHB4	100	98
EPHB6	93	91
ERBB2	81	83
ERBB3	100	100
ERBB4	94	100
ERK1	100	100

Target	BAY1830839	
Gene Symbol	%Ctrl @ 100nM	%Ctrl @ 1000nM
ERK2	91	97
ERK3	89	100
ERK4	90	76
ERK5	96	96
ERK8	97	88
ERN1	91	92
FAK	95	100
FER	91	100
FES	100	92
FGFR1	95	74
FGFR2	90	71
FGFR3	96	68
FGFR3(G697C)	91	88
FGFR4	94	99
FGR	100	96
FLT1	100	96
FLT3	100	77
FLT3(D835H)	76	33
FLT3(D835Y)	79	21
FLT3(ITD)	87	27
FLT3(K663Q)	99	73
FLT3(N841I)	81	30
FLT3(R834Q)	100	73
FLT3-autoinhibited	100	97
FLT4	100	100
FRK	95	95
FYN	100	99
GAK	90	68
GCN2(Kin.Dom.2,S808G)	99	88
GRK1	100	100
GRK4	95	95
GRK7	77	65
GSK3A	100	100
GSK3B	86	85
HASPIN	87	79
HCK	93	70
HIPK1	79	64
HIPK2	96	97
HIPK3	97	86
HIPK4	88	96
HPK1	81	65
HUNK	100	100
ICK	92	100
IGF1R	96	100
IKK-alpha	95	100
IKK-beta	88	84
IKK-epsilon	96	99
INSR	91	86
INSRR	87	100

Target	BAY1830839	
Gene Symbol	%Ctrl @ 100nM	%Ctrl @ 1000nM
IRAK1	50	7
IRAK3	53	28
IRAK4	0.15	0
ITK	85	100
JAK1(JH1domain-catalytic)	100	97
JAK1(JH2domain-pseudokinase)	97	34
JAK2(JH1domain-catalytic)	68	40
JAK3(JH1domain-catalytic)	93	72
JNK1	90	90
JNK2	84	77
JNK3	90	80
KIT	88	72
KIT(A829P)	79	74
KIT(D816H)	97	80
KIT(D816V)	86	58
KIT(L576P)	81	50
KIT(V559D)	84	57
KIT(V559D,T670I)	83	80
KIT(V559D,V654A)	96	84
KIT-autoinhibited	96	95
LATS1	91	97
LATS2	84	100
LCK	100	100
LIMK1	99	100
LIMK2	98	100
LKB1	61	91
LOK	100	94
LRRK2	91	38
LRRK2(G2019S)	84	21
LTK	100	93
LYN	72	75
LZK	93	79
MAK	91	93
MAP3K1	78	73
MAP3K15	100	87
MAP3K2	100	53
MAP3K3	100	52
MAP3K4	97	100
MAP4K2	93	59
MAP4K3	97	93
MAP4K4	94	75
MAP4K5	100	84
MAPKAPK2	99	100
MAPKAPK5	95	95
MARK1	84	48
MARK2	87	80
MARK3	95	70
MARK4	87	64
MAST1	97	94

Target	BAY1830839	
Gene Symbol	%Ctrl @ 100nM	%Ctrl @ 1000nM
MEK1	97	69
MEK2	89	55
MEK3	98	100
MEK4	100	100
MEK5	100	98
MEK6	83	81
MELK	85	54
MERTK	56	78
MET	92	100
MET(M1250T)	98	99
MET(Y1235D)	75	74
MINK	75	48
MKK7	95	88
MKNK1	86	97
MKNK2	91	92
MLCK	94	97
MLK1	97	92
MLK2	99	73
MLK3	98	98
MRCKA	100	100
MRCKB	89	99
MST1	100	95
MST1R	83	95
MST2	83	51
MST3	100	97
MST4	100	100
MTOR	100	90
MUSK	93	100
MYLK	100	95
MYLK2	97	94
MYLK4	83	89
MYO3A	97	96
MYO3B	88	99
NDR1	99	100
NDR2	98	100
NEK1	84	85
NEK10	89	92
NEK11	99	85
NEK2	89	73
NEK3	95	97
NEK4	70	81
NEK5	89	100
NEK6	89	90
NEK7	98	100
NEK9	98	100
NIK	92	100
NIM1	99	100
NLK	85	100
OSR1	100	100

Target	BAY1830839	
Gene Symbol	%Ctrl @ 100nM	%Ctrl @ 1000nM
p38-alpha	98	100
p38-beta	93	98
p38-delta	93	100
p38-gamma	79	82
PAK1	90	87
PAK2	75	46
PAK3	95	84
PAK4	100	96
PAK6	100	100
PAK7	97	98
PCTK1	91	87
PCTK2	95	90
PCTK3	99	94
PDGFRA	100	100
PDGFRB	100	100
PDPK1	89	95
PFCDPK1(P.falciparum)	70	93
PFPK5(P.falciparum)	98	95
PFTAIRE2	96	99
PFTK1	96	89
PHKG1	77	95
PHKG2	100	77
PIK3C2B	93	93
PIK3C2G	94	76
PIK3CA	100	100
PIK3CA(C420R)	96	85
PIK3CA(E542K)	94	100
PIK3CA(E545A)	97	78
PIK3CA(E545K)	93	99
PIK3CA(H1047L)	89	95
PIK3CA(H1047Y)	100	77
PIK3CA(I800L)	100	95
PIK3CA(M1043I)	92	70
PIK3CA(Q546K)	90	94
PIK3CB	84	100
PIK3CD	92	88
PIK3CG	100	100
PIK4CB	99	100
PIM1	99	98
PIM2	73	96
PIM3	95	100
PIP5K1A	92	56
PIP5K1C	100	100
PIP5K2B	96	84
PIP5K2C	99	100
PKAC-alpha	86	90
PKAC-beta	89	92
PKMYT1	85	100
PKN1	100	100

Target	BAY1830839	
Gene Symbol	%Ctrl @ 100nM	%Ctrl @ 1000nM
PKN2	97	78
PKNB(M.tuberculosis)	87	89
PLK1	97	100
PLK2	86	76
PLK3	94	87
PLK4	92	91
PRKCD	95	80
PRKCE	100	100
PRKCH	100	86
PRKCI	98	91
PRKCQ	90	100
PRKD1	100	100
PRKD2	94	100
PRKD3	88	100
PRKG1	70	84
PRKG2	90	91
PRKR	95	72
PRKX	84	94
PRP4	100	100
PYK2	86	98
QSK	100	100
RAF1	98	100
RET	100	98
RET(M918T)	100	83
RET(V804L)	98	82
RET(V804M)	100	78
RIOK1	92	55
RIOK2	98	82
RIOK3	100	59
RIPK1	89	100
RIPK2	85	96
RIPK4	96	99
RIPK5	100	92
ROCK1	92	64
ROCK2	100	80
ROS1	100	100
RPS6KA4(Kin.Dom.1-N-terminal)	88	98
RPS6KA4(Kin.Dom.2-C-terminal)	85	90
RPS6KA5(Kin.Dom.1-N-terminal)	100	90
RPS6KA5(Kin.Dom.2-C-terminal)	95	100
RSK1(Kin.Dom.1-N-terminal)	69	71
RSK1(Kin.Dom.2-C-terminal)	100	86
RSK2(Kin.Dom.1-N-terminal)	98	95
RSK2(Kin.Dom.2-C-terminal)	89	90
RSK3(Kin.Dom.1-N-terminal)	96	95
RSK3(Kin.Dom.2-C-terminal)	100	89
RSK4(Kin.Dom.1-N-terminal)	100	100
RSK4(Kin.Dom.2-C-terminal)	100	80
S6K1	82	94

Target	BAY1830839	
Gene Symbol	%Ctrl @ 100nM	%Ctrl @ 1000nM
SBK1	72	80
SGK	89	94
SgK110	99	86
SGK2	99	100
SGK3	100	100
SIK	92	90
SIK2	90	94
SLK	100	100
SNARK	100	95
SNRK	100	94
SRC	83	94
SRMS	93	86
SRPK1	98	89
SRPK2	91	100
SRPK3	100	100
STK16	100	83
STK33	79	72
STK35	91	98
STK36	100	99
STK39	100	100
SYK	87	58
TAK1	73	30
TAOK1	97	97
TAOK2	92	88
TAOK3	92	92
TBK1	98	83
TEC	91	100
TESK1	93	98
TGFBR1	96	94
TGFBR2	100	100
TIE1	88	100
TIE2	91	93
TLK1	100	98
TLK2	94	78
TNIK	75	60
TNK1	95	86
TNK2	95	100
TNNI3K	99	100
TRKA	84	39
TRKB	79	26
TRKC	84	43
TRPM6	81	89
TSSK1B	86	93
TTK	71	92
TXK	98	100
TYK2(JH1domain-catalytic)	85	67
TYK2(JH2domain-pseudokinase)	100	100
TYRO3	100	100
ULK1	86	86

Target	BAY1830839	
Gene Symbol	%Ctrl @ 100nM	%Ctrl @ 1000nM
ULK2	93	82
ULK3	97	83
VEGFR2	100	95
VRK2	89	73
WEE1	94	87
WEE2	98	100
WNK1	97	92
WNK3	97	92
YANK1	95	87
YANK2	100	99
YANK3	88	100
YES	98	100
YSK1	93	87
YSK4	97	100
ZAK	90	96
ZAP70	99	97

Table S3. Matrix of Compound Screen for BAY1834845.

Target	BAY1834845	
Gene Symbol	%Ctrl @ 100nM	%Ctrl @ 1000nM
AAK1	100	80
ABL1(E255K)-phosphorylated	75	68
ABL1(F317I)-nonphosphorylated	92	99
ABL1(F317I)-phosphorylated	88	78
ABL1(F317L)-nonphosphorylated	87	93
ABL1(F317L)-phosphorylated	100	100
ABL1(H396P)-nonphosphorylated	89	87
ABL1(H396P)-phosphorylated	89	80
ABL1(M351T)-phosphorylated	83	96
ABL1(Q252H)-nonphosphorylated	71	67
ABL1(Q252H)-phosphorylated	88	96
ABL1(T315I)-nonphosphorylated	100	100
ABL1(T315I)-phosphorylated	94	100
ABL1(Y253F)-phosphorylated	100	97
ABL1-nonphosphorylated	80	83
ABL1-phosphorylated	88	83
ABL2	100	100
ACVR1	100	100
ACVR1B	100	100
ACVR2A	76	78
ACVR2B	99	91
ACVRL1	100	100
ADCK3	96	87
ADCK4	87	88
AKT1	85	91
AKT2	100	100
AKT3	100	100
ALK	100	100
ALK(C1156Y)	100	95
ALK(L1196M)	100	100
AMPK-alpha1	92	99
AMPK-alpha2	100	100
ANKK1	83	68
ARK5	100	100
ASK1	99	85
ASK2	100	98
AURKA	100	100
AURKB	74	82
AURKC	85	98
AXL	100	100
BIKE	69	43
BLK	100	91
BMPR1A	71	63
BMPR1B	100	100
BMPR2	94	85
BMX	98	85
BRAF	89	86
BRAF(V600E)	88	97

Target	BAY1834845	
Gene Symbol	%Ctrl @ 100nM	%Ctrl @ 1000nM
BRK	91	98
BRSK1	100	100
BRSK2	87	100
BTK	98	100
BUB1	100	100
CAMK1	59	91
CAMK1D	100	95
CAMK1G	98	85
CAMK2A	96	80
CAMK2B	100	86
CAMK2D	100	100
CAMK2G	98	94
CAMK4	86	100
CAMKK1	100	78
CAMKK2	91	64
CASK	82	78
CDC2L1	100	100
CDC2L2	100	100
CDC2L5	100	100
CDK11	100	94
CDK2	99	100
CDK3	74	73
CDK4-cyclinD1	95	100
CDK4-cyclinD3	100	99
CDK5	96	100
CDK7	90	90
CDK8	96	77
CDK9	100	100
CDKL1	75	80
CDKL2	96	94
CDKL3	100	92
CDKL5	100	100
CHEK1	100	100
CHEK2	75	93
CIT	100	100
CLK1	89	81
CLK2	99	70
CLK3	77	81
CLK4	100	100
CSF1R	100	94
CSF1R-autoinhibited	98	100
CSK	100	94
CSNK1A1	99	79
CSNK1A1L	100	100
CSNK1D	99	100
CSNK1E	91	85
CSNK1G1	100	100
CSNK1G2	98	89
CSNK1G3	97	86

Target	BAY1834845	
Gene Symbol	%Ctrl @ 100nM	%Ctrl @ 1000nM
CSNK2A1	86	66
CSNK2A2	100	100
CTK	100	100
DAPK1	100	100
DAPK2	91	85
DAPK3	99	82
DCAMKL1	75	58
DCAMKL2	100	100
DCAMKL3	100	100
DDR1	71	100
DDR2	96	96
DLK	96	100
DMPK	100	100
DMPK2	96	88
DRAK1	100	100
DRAK2	100	100
DYRK1A	96	89
DYRK1B	90	96
DYRK2	81	85
EGFR	81	91
EGFR(E746-A750del)	97	100
EGFR(G719C)	100	98
EGFR(G719S)	76	76
EGFR(L747-E749del,A750P)	100	97
EGFR(L747-S752del,P753S)	100	100
EGFR(L747-T751del,Sins)	84	92
EGFR(L858R)	100	100
EGFR(L858R,T790M)	96	99
EGFR(L861Q)	100	100
EGFR(S752-I759del)	91	82
EGFR(T790M)	84	82
EIF2AK1	100	100
EPHA1	91	91
EPHA2	98	96
EPHA3	100	100
EPHA4	97	100
EPHA5	100	98
EPHA6	99	100
EPHA7	100	99
EPHA8	100	100
EPHB1	97	88
EPHB2	98	89
EPHB3	93	97
EPHB4	100	100
EPHB6	82	84
ERBB2	100	100
ERBB3	96	98
ERBB4	88	90
ERK1	100	96

Target	BAY1834845	
Gene Symbol	%Ctrl @ 100nM	%Ctrl @ 1000nM
ERK2	100	79
ERK3	100	94
ERK4	100	100
ERK5	100	98
ERK8	87	75
ERN1	84	100
FAK	100	100
FER	98	100
FES	92	90
FGFR1	95	93
FGFR2	82	84
FGFR3	83	75
FGFR3(G697C)	81	76
FGFR4	94	97
FGR	100	84
FLT1	89	100
FLT3	100	93
FLT3(D835H)	62	30
FLT3(D835Y)	100	36
FLT3(ITD)	84	48
FLT3(K663Q)	100	93
FLT3(N841I)	94	52
FLT3(R834Q)	100	100
FLT3-autoinhibited	100	100
FLT4	81	94
FRK	98	100
FYN	95	93
GAK	100	100
GCN2(Kin.Dom.2,S808G)	100	100
GRK1	81	86
GRK4	77	100
GRK7	92	75
GSK3A	95	100
GSK3B	100	100
HASPIN	92	98
HCK	100	100
HIPK1	79	65
HIPK2	87	92
HIPK3	100	100
HIPK4	80	88
HPK1	74	59
HUNK	96	95
ICK	100	100
IGF1R	100	88
IKK-alpha	100	100
IKK-beta	97	99
IKK-epsilon	87	95
INSR	93	80
INSRR	91	92

Target	BAY1834845	
Gene Symbol	%Ctrl @ 100nM	%Ctrl @ 1000nM
IRAK1	89	20
IRAK3	100	63
IRAK4	0,3	0
ITK	100	95
JAK1(JH1domain-catalytic)	93	100
JAK1(JH2domain-pseudokinase)	90	27
JAK2(JH1domain-catalytic)	87	49
JAK3(JH1domain-catalytic)	94	67
JNK1	84	87
JNK2	88	86
JNK3	100	100
KIT	95	90
KIT(A829P)	95	100
KIT(D816H)	94	100
KIT(D816V)	89	85
KIT(L576P)	100	100
KIT(V559D)	88	79
KIT(V559D,T670I)	100	96
KIT(V559D,V654A)	100	93
KIT-autoinhibited	98	92
LATS1	100	88
LATS2	93	98
LCK	92	100
LIMK1	100	100
LIMK2	100	95
LKB1	79	58
LOK	100	100
LRRK2	69	50
LRRK2(G2019S)	84	35
LTK	100	100
LYN	99	88
LZK	100	100
MAK	84	100
MAP3K1	100	88
MAP3K15	100	100
MAP3K2	97	55
MAP3K3	79	38
MAP3K4	84	88
MAP4K2	100	93
MAP4K3	98	100
MAP4K4	81	42
MAP4K5	100	84
MAPKAPK2	90	100
MAPKAPK5	100	97
MARK1	93	88
MARK2	100	96
MARK3	100	90
MARK4	93	99
MAST1	92	95

Target	BAY1834845	
Gene Symbol	%Ctrl @ 100nM	%Ctrl @ 1000nM
MEK1	100	93
MEK2	91	81
MEK3	82	69
MEK4	100	100
MEK5	100	100
MEK6	100	100
MELK	95	97
MERTK	86	84
MET	100	99
MET(M1250T)	93	100
MET(Y1235D)	83	83
MINK	95	50
MKK7	98	100
MKNK1	100	99
MKNK2	100	100
MLCK	100	98
MLK1	100	86
MLK2	41	61
MLK3	100	100
MRCKA	95	83
MRCKB	100	100
MST1	100	97
MST1R	92	97
MST2	79	97
MST3	92	97
MST4	77	66
MTOR	96	94
MUSK	93	91
MYLK	68	56
MYLK2	100	100
MYLK4	99	90
MYO3A	100	100
MYO3B	93	81
NDR1	88	90
NDR2	100	97
NEK1	100	98
NEK10	100	100
NEK11	98	97
NEK2	100	99
NEK3	57	66
NEK4	96	80
NEK5	77	75
NEK6	100	100
NEK7	100	100
NEK9	90	93
NIK	100	100
NIM1	89	97
NLK	96	80
OSR1	100	100

Target	BAY1834845	
Gene Symbol	%Ctrl @ 100nM	%Ctrl @ 1000nM
p38-alpha	100	100
p38-beta	100	100
p38-delta	81	100
p38-gamma	64	68
PAK1	100	80
PAK2	100	93
PAK3	92	99
PAK4	100	100
PAK6	100	100
PAK7	100	97
PCTK1	88	98
PCTK2	100	100
PCTK3	100	100
PDGFRA	100	100
PDGFRB	89	95
PDPK1	91	97
PFCDPK1(P.falciparum)	99	99
PFPK5(P.falciparum)	92	91
PFTAIRE2	100	93
PFTK1	99	93
PHKG1	99	96
PHKG2	100	86
PIK3C2B	95	90
PIK3C2G	96	100
PIK3CA	100	95
PIK3CA(C420R)	89	100
PIK3CA(E542K)	98	100
PIK3CA(E545A)	62	69
PIK3CA(E545K)	68	82
PIK3CA(H1047L)	100	100
PIK3CA(H1047Y)	74	87
PIK3CA(I800L)	77	89
PIK3CA(M1043I)	96	96
PIK3CA(Q546K)	98	97
PIK3CB	100	100
PIK3CD	100	97
PIK3CG	98	90
PIK4CB	100	100
PIM1	81	86
PIM2	47	83
PIM3	98	82
PIP5K1A	84	100
PIP5K1C	96	100
PIP5K2B	100	93
PIP5K2C	100	100
PKAC-alpha	82	98
PKAC-beta	100	99
PKMYT1	100	100
PKN1	100	100

Target	BAY1834845	
Gene Symbol	%Ctrl @ 100nM	%Ctrl @ 1000nM
PKN2	100	100
PKNB(M.tuberculosis)	100	87
PLK1	80	87
PLK2	74	59
PLK3	71	57
PLK4	71	71
PRKCD	90	100
PRKCE	97	96
PRKCH	93	100
PRKCI	80	97
PRKCQ	100	100
PRKD1	100	98
PRKD2	100	100
PRKD3	100	100
PRKG1	100	100
PRKG2	87	91
PRKR	100	100
PRKX	100	100
PRP4	85	100
PYK2	71	74
QSK	93	71
RAF1	94	98
RET	100	100
RET(M918T)	84	93
RET(V804L)	86	83
RET(V804M)	100	76
RIOK1	100	92
RIOK2	100	98
RIOK3	95	89
RIPK1	83	75
RIPK2	99	90
RIPK4	100	100
RIPK5	90	80
ROCK1	100	99
ROCK2	100	91
ROS1	98	100
RPS6KA4(Kin.Dom.1-N-terminal)	100	100
RPS6KA4(Kin.Dom.2-C-terminal)	100	96
RPS6KA5(Kin.Dom.1-N-terminal)	95	84
RPS6KA5(Kin.Dom.2-C-terminal)	100	100
RSK1(Kin.Dom.1-N-terminal)	100	100
RSK1(Kin.Dom.2-C-terminal)	100	99
RSK2(Kin.Dom.1-N-terminal)	79	89
RSK2(Kin.Dom.2-C-terminal)	100	100
RSK3(Kin.Dom.1-N-terminal)	100	100
RSK3(Kin.Dom.2-C-terminal)	92	93
RSK4(Kin.Dom.1-N-terminal)	80	81
RSK4(Kin.Dom.2-C-terminal)	94	91
S6K1	95	98

Target	BAY1834845	
Gene Symbol	%Ctrl @ 100nM	%Ctrl @ 1000nM
SBK1	100	100
SGK	100	100
SgK110	98	88
SGK2	100	100
SGK3	97	100
SIK	100	100
SIK2	96	88
SLK	98	100
SNARK	95	100
SNRK	93	96
SRC	86	86
SRMS	95	95
SRPK1	90	100
SRPK2	100	100
SRPK3	93	100
STK16	100	82
STK33	92	100
STK35	100	100
STK36	93	73
STK39	84	99
SYK	92	83
TAK1	83	45
TAOK1	100	100
TAOK2	72	73
TAOK3	99	96
TBK1	65	63
TEC	100	100
TESK1	80	76
TGFBR1	100	99
TGFBR2	100	100
TIE1	90	78
TIE2	90	96
TLK1	100	100
TLK2	100	94
TNIK	97	60
TNK1	96	93
TNK2	100	100
TNNI3K	100	97
TRKA	100	48
TRKB	91	40
TRKC	94	67
TRPM6	77	75
TSSK1B	100	100
TTK	100	100
TXK	89	80
TYK2(JH1domain-catalytic)	88	72
TYK2(JH2domain-pseudokinase)	100	100
TYRO3	94	87
ULK1	100	100

Target	BAY1834845	
Gene Symbol	%Ctrl @ 100nM	%Ctrl @ 1000nM
ULK2	90	91
ULK3	99	89
VEGFR2	98	94
VRK2	95	100
WEE1	94	94
WEE2	95	100
WNK1	100	100
WNK3	100	100
YANK1	100	100
YANK2	100	100
YANK3	100	100
YES	100	97
YSK1	100	100
YSK4	100	100
ZAK	87	80
ZAP70	89	96
ULK2	90	91
ULK3	99	89
VEGFR2	98	94
VRK2	95	100
WEE1	94	94
WEE2	95	100
WNK1	100	100
WNK3	100	100
YANK1	100	100
YANK2	100	100
YANK3	100	100
YES	100	97
YSK1	100	100
YSK4	100	100
ZAK	87	80
ZAP70	89	96
ULK2	90	91
ULK3	99	89
VEGFR2	98	94
VRK2	95	100
WEE1	94	94
WEE2	95	100
WNK1	100	100
WNK3	100	100
YANK1	100	100
YANK2	100	100
YANK3	100	100
YES	100	97
YSK1	100	100
YSK4	100	100
ZAK	87	80
ZAP70	89	96

Table S4. Matrix of compound screen for compound 5

Target	Compound 5
Gene Symbol	% Ctrl @ 1 µM compound concentration
AAK1	100
ABL1(E255K)-phosphorylated	73
ABL1(F317I)-nonphosphorylated	78
ABL1(F317I)-phosphorylated	100
ABL1(F317L)-nonphosphorylated	94
ABL1(F317L)-phosphorylated	90
ABL1(H396P)-nonphosphorylated	58
ABL1(H396P)-phosphorylated	59
ABL1(M351T)-phosphorylated	96
ABL1(Q252H)-nonphosphorylated	52
ABL1(Q252H)-phosphorylated	89
ABL1(T315I)-nonphosphorylated	44
ABL1(T315I)-phosphorylated	85
ABL1(Y253F)-phosphorylated	41
ABL1-nonphosphorylated	93
ABL1-phosphorylated	54
ABL2	97
ACVR1	89
ACVR1B	100
ACVR2A	76
ACVR2B	87
ACVRL1	100
ADCK3	99
ADCK4	100
AKT1	100
AKT2	96
AKT3	100
ALK	100
ALK(C1156Y)	97
ALK(L1196M)	100
AMPK-alpha1	100.00
AMPK-alpha2	100.00
ANKK1	100.00
ARK5	68.00
ASK1	100.00
ASK2	100.00
AURKA	100.00
AURKB	83.00
AURKC	100.00
AXL	100.00
BIKE	100
BLK	100
BMPR1A	96
BMPR1B	96
BMPR2	45
BMX	100
BRAF	77
BRAF(V600E)	73
BRK	98
BRSK1	100
BRSK2	100
BTK	100
BUB1	100
CAMK1	100
CAMK1D	100
CAMK1G	100
CAMK2A	100
CAMK2B	100
CAMK2D	100
CAMK2G	100

Target	Compound 5
Gene Symbol	% Ctrl @ 1 µM compound concentration
CAMK4	100
CAMKK1	100
CAMKK2	100
CASK	100
CDC2L1	100
CDC2L2	100
CDC2L5	78
CDK11	100
CDK2	100
CDK3	100
CDK4-cyclinD1	98
CDK4-cyclinD3	41
CDK5	100
CDK7	54
CDK8	100
CDK9	100
CDKL1	100
CDKL2	84
CDKL3	100
CDKL5	95
CHEK1	100
CHEK2	100
CIT	79
CLK1	100
CLK2	100
CLK3	100
CLK4	100
CSF1R	100
CSF1R-autoinhibited	80
CSK	100
CSNK1A1	83
CSNK1A1L	89
CSNK1D	100
CSNK1E	100
CSNK1G1	100
CSNK1G2	100
CSNK1G3	100
CSNK2A1	83
CSNK2A2	100
CTK	100
DAPK1	98
DAPK2	100
DAPK3	100
DCAMKL1	59
DCAMKL2	100
DCAMKL3	100
DDR1	100
DDR2	100
DLK	99
DMPK	100
DMPK2	98
DRAK1	100
DRAK2	100
DYRK1A	73
DYRK1B	100
DYRK2	73
EGFR	100
EGFR(E746-A750del)	100
EGFR(G719C)	100
EGFR(G719S)	100

Target	Compound 5
**Gene Symbol**	**% Ctrl @ 1 µM compound concentration**
EGFR(L747-E749del, A750P)	100
EGFR(L747-S752del, P753S)	100
EGFR(L747-T751del,Sins)	100
EGFR(L858R)	100
EGFR(L858R,T790M)	100
EGFR(L861Q)	100
EGFR(S752-I759del)	100
EGFR(T790M)	64
EIF2AK1	53
EPHA1	100
EPHA2	100
EPHA3	100
EPHA4	100
EPHA5	100
EPHA6	100
EPHA7	100
EPHA8	100
EPHB1	100
EPHB2	100
EPHB3	100
EPHB4	100
EPHB6	43
ERBB2	100
ERBB3	80
ERBB4	100
ERK1	100
ERK2	100
ERK3	100
ERK4	100
ERK5	100
ERK8	100
ERN1	39
FAK	100
FER	100
FES	100
FGFR1	98
FGFR2	100
FGFR3	100
FGFR3(G697C)	95
FGFR4	100
FGR	100
FLT1	35
FLT3	100
FLT3(D835H)	100
FLT3(D835Y)	100
FLT3(ITD)	27
FLT3(K663Q)	100
FLT3(N841I)	98
FLT3(R834Q)	100
FLT3-autoinhibited	100
FLT4	87
FRK	100
FYN	100
GAK	100
GCN2(Kin.Dom.2,S808G)	89
GRK1	81
GRK4	100
GRK7	100
GSK3A	100
GSK3B	74
HASPIN	100
HCK	94

Target	Compound 5
**Gene Symbol**	**% Ctrl @ 1 µM compound concentration**
HIPK1	66
HIPK2	100
HIPK3	96
HIPK4	100
HPK1	92
HUNK	100
ICK	100
IGF1R	100
IKK-alpha	65
IKK-beta	72
IKK-epsilon	100
INSR	100
INSRR	100
IRAK1	56
IRAK3	100
IRAK4	0.95
ITK	100
JAK1(JH1domain-catalytic)	100
JAK1(JH2domain-pseudokinase)	47
JAK2(JH1domain-catalytic)	100
JAK3(JH1domain-catalytic)	92
JNK1	82
JNK2	92
JNK3	89
KIT	100
KIT(A829P)	100
KIT(D816H)	100
KIT(D816V)	100
KIT(L576P)	100
KIT(V559D)	100
KIT(V559D,T670I)	100
KIT(V559D,V654A)	100
KIT-autoinhibited	99
LATS1	88
LATS2	49
LCK	99
LIMK1	100
LIMK2	100
LKB1	100
LOK	90
LRRK2	93
LRRK2(G2019S)	86
LTK	100
LYN	87
LZK	100
MAK	100
MAP3K1	98
MAP3K15	88
MAP3K2	55
MAP3K3	78
MAP3K4	99
MAP4K2	91
MAP4K3	100
MAP4K4	100
MAP4K5	100
MAPKAPK2	100
MAPKAPK5	94
MARK1	100
MARK2	100
MARK3	98
MARK4	100
MAST1	100

**Target**	**Compound 5**
**Gene Symbol**	**% Ctrl @ 1 µM compound concentration**
MEK1	100
MEK2	99
MEK3	100
MEK4	100
MEK5	92
MEK6	100
MELK	100
MERTK	100
MET	100
MET(M1250T)	100
MET(Y1235D)	100
MINK	100
MKK7	100
MKNK1	100
MKNK2	91
MLCK	100
MLK1	100
MLK2	100
MLK3	100
MRCKA	100
MRCKB	100
MST1	100
MST1R	100
MST2	100
MST3	100
MST4	100
MTOR	100
MUSK	100
MYLK	65
MYLK2	100
MYLK4	100
MYO3A	100
MYO3B	100
NDR1	87
NDR2	82
NEK1	100
NEK10	98
NEK11	91
NEK2	100
NEK3	64
NEK4	100
NEK5	89
NEK6	100
NEK7	93
NEK9	97
NIK	100
NIM1	82
NLK	51
OSR1	100
PAK1	100
PAK2	100
PAK3	100
PAK4	94
PAK6	100
PAK7	100
PCTK1	77
PCTK2	100
PCTK3	100
PDGFRA	100
PDGFRB	100
PDPK1	100
PFCDPK1(P.falciparum)	91

**Target**	**Compound 5**
**Gene Symbol**	**% Ctrl @ 1 µM compound concentration**
PFPK5(P.falciparum)	100
PFTAIRE2	89
PFTK1	100
PHKG1	100
PHKG2	100
PIK3C2B	100
PIK3C2G	95
PIK3CA	100
PIK3CA(C420R)	78
PIK3CA(E542K)	100
PIK3CA(E545A)	90
PIK3CA(E545K)	100
PIK3CA(H1047L)	100
PIK3CA(H1047Y)	78
PIK3CA(I800L)	100
PIK3CA(M1043I)	100
PIK3CA(Q546K)	89
PIK3CB	47
PIK3CD	100
PIK3CG	87
PIK4CB	83
PIM1	88
PIM2	100
PIM3	100
PIP5K1A	100
PIP5K1C	100
PIP5K2B	100
PIP5K2C	74
PKAC-alpha	100
PKAC-beta	100
PKMYT1	100
PKN1	100
PKN2	100
PKNB(M.tuberculosis)	86
PLK1	100
PLK2	100
PLK3	80
PLK4	100
PRKCD	100
PRKCE	62
PRKCH	100
PRKCI	82
PRKCQ	100
PRKD1	89
PRKD2	100
PRKD3	63
PRKG1	100
PRKG2	100
PRKR	100
PRKX	100
PRP4	100
PYK2	92
QSK	91
RAF1	100
RET	100
RET(M918T)	94
RET(V804L)	100
RET(V804M)	100
RIOK1	26
RIOK2	80
RIOK3	100
RIPK1	100

Target	Compound 5
Gene Symbol	% Ctrl @ 1 µM compound concentration
RIPK2	100
RIPK4	89
RIPK5	41
ROCK1	75
ROCK2	95
ROS1	100
RPS6KA4(Kin.Dom.1-N-terminal)	82
RPS6KA4(Kin.Dom.2-C-terminal)	89
RPS6KA5(Kin.Dom.1-N-terminal)	100
RPS6KA5(Kin.Dom.2-C-terminal)	100
RSK1(Kin.Dom.1-N-terminal)	100
RSK1(Kin.Dom.2-C-terminal)	87
RSK2(Kin.Dom.1-N-terminal)	81
RSK2(Kin.Dom.2-C-terminal)	100
RSK3(Kin.Dom.1-N-terminal)	100
RSK3(Kin.Dom.2-C-terminal)	98
RSK4(Kin.Dom.1-N-terminal)	92
RSK4(Kin.Dom.2-C-terminal)	100
S6K1	100
SBK1	70
SGK	100
SGK2	100
SGK3	98
SIK	100
SIK2	98
SLK	100
SNARK	100
SNRK	96
SRC	90
SRMS	95
SRPK1	100
SRPK2	100
SRPK3	100
STK16	100
STK33	100
STK35	100
STK36	89
STK39	84
SYK	96
SgK110	100
TAK1	54
TAOK1	100
TAOK2	82
TAOK3	100
TBK1	68
TEC	96
TESK1	100

Target	Compound 5
Gene Symbol	% Ctrl @ 1 µM compound concentration
TGFBR1	100
TGFBR2	100
TIE1	100
TIE2	100
TLK1	96
TLK2	100
TNIK	100
TNK1	87
TNK2	100
TNNI3K	100
TRKA	85
TRKB	88
TRKC	91
TRPM6	95
TSSK1B	100
TTK	100
TXK	100
TYK2(JH1domain-catalytic)	100
TYK2(JH2domain-pseudokinase)	100
TYRO3	100
ULK1	72
ULK2	78
ULK3	86
VEGFR2	63
VRK2	100
WEE1	78
WEE2	91
WNK1	100
WNK3	100
YANK1	75
YANK2	100
YANK3	100
YES	100
YSK1	100
YSK4	100
ZAK	100
ZAP70	100
p38-alpha	100
p38-beta	95
p38-delta	100
p38-gamma	100

Murine cellular potency

Cells were isolated from murine spleen and treated with either BAY1834845 or BAY1830839 in the presence of 1 µg/mL LPS from *Escherichia coli* 0127:B8 (Sigma, L4516-1MG), respectively, for 24 hours. The amount of secreted TNFα was quantified using multiplex protein assay (Mouse ProInflammatory 7-Plex Mesoscale, MSD, N75012B-1) according to the manufacturer's instructions. IC_{50} values were calculated using 4-parameter fit.

BAY1834845 or BAY1830839 inhibited the secretion of TNFα in LPS-stimulated murine splenic cells with an IC_{50} of 385 nM and 47 nM, respectively.

In vivo pharmacology

IL-1-beta induced systemic inflammation in mice

BALB/c mice (8 week old) were obtained from Charles River Laboratories, Germany and had access to food and water ad libitum. All animals were housed according to institutional guidelines under a 12 h/12 h light/dark cycle and maintained under standard conditions (20–22 °C, 50–70% humidity). Mice were divided into 5 animals per group. The test compound or its vehicle was orally administrated 6 hours before a total of 90 µg of IL-1beta/kg body weight (R&D, Cat. No. 401-ML/CF) was administered intraperitoneally. Two hours after administration of the IL-1beta, TNF-alpha and IL-6 were determined in the plasma after final removal of blood using the Mouse ProInflammatory 7-Plex Tissue Culture Kit (MSD, Cat. No. K15012B) in accordance with manufacturer's instructions.

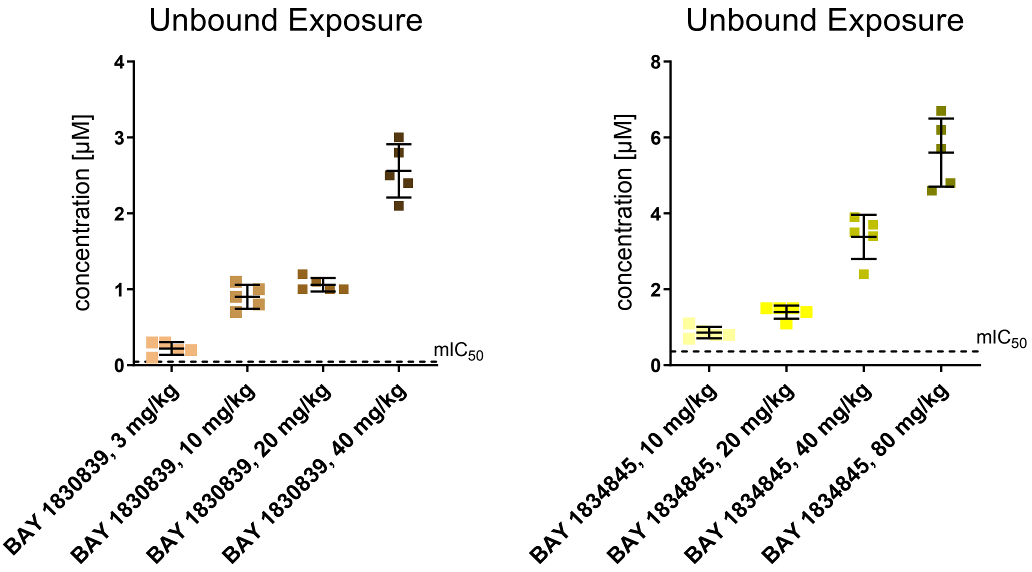

Figure S1: Unbound compound exposure data for **BAY1830839** (left graph) and **BAY1834845** (right graph) in IL-1-beta induced systemic inflammation model in mice. Blood samples were processed as described in the drug metabolism and pharmacokinetics (DMPK) part of the supplement, see section pharmacokinetics in mice. Mouse IC_{50}: *in vitro* murine cellular IC_{50}, which was determined according to the methods described in the section murine cellular potency.

LPS (Lipopolysaccharides) induced systemic inflammation in mice

BALB/c mice (8 week old) were obtained from Janvier Labs, France and had access to food and water ad libitum. All animals were housed according to institutional guidelines under a 12 h/12 h light/dark cycle and maintained under standard conditions (20–22 °C, 50–70% humidity). Mice were divided into 5 animals per group. The test compound or its vehicle was orally administrated 4 hours before a total of 0.2 mg of LPS /kg body weight (Sigma-Aldrich, Cat. No. L4391) was administered intraperitoneally. Animals were sacrificed 1.5 hours after injection of LPS. TNF-alpha and IL-6 were determined in the plasma after final removal of blood using the Mouse ProInflammatory 7-Plex Tissue Culture Kit (MSD, Cat. No. K15012B) in accordance with manufacturer's instructions.

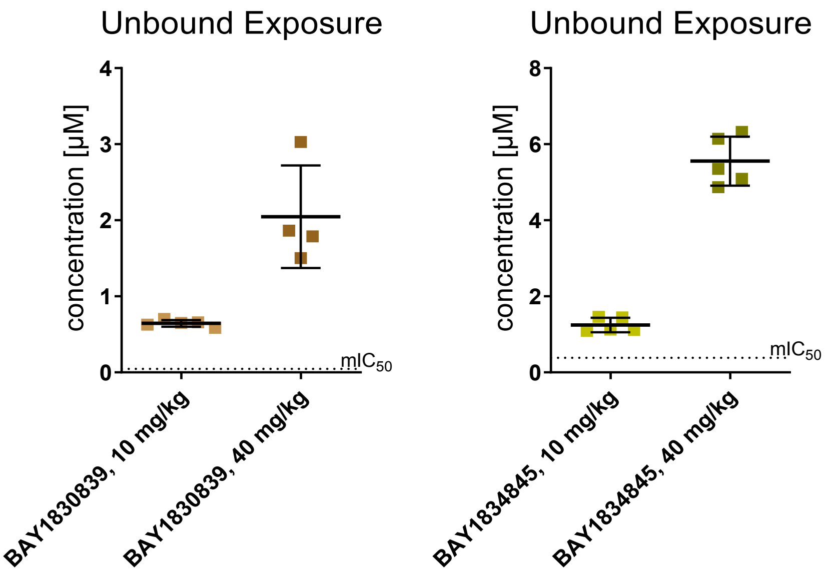

Figure S2: Unbound compound exposure data for **BAY1830839** (left graph) and **BAY1834845** (right graph) in LPS (Lipopolysaccharides) induced systemic inflammation model in mice. Blood samples were processed as described in the drug metabolism and pharmacokinetics (DMPK) part of the supplement, see section pharmacokinetics in mice. Mouse IC_{50}: *in vitro* murine cellular IC_{50}, which was determined according to the methods described in the section murine cellular potency.

Imiquimod-induced topical inflammation in mice

BALB/c mice (9 to 10 weeks old) were obtained from Janvier Labs, France and had access to food and water ad libitum. All animals were housed according to institutional guidelines under a 12 h/12 h light/dark cycle and maintained under standard conditions (target: 22 ± 2

°C, target: 50 ± 15% humidity). Mice were divided into 10 animals per group. For induction of the psoriasis-like inflammatory phenotype fur was removed at the back 1 day before and 3.5 mg of imiquimod (equivalent to 70 mg of Aldara© 5% crème, Meda AB) was daily topically administered for 7 consecutive days. The test compound or its vehicle was orally administered twice daily. Healthy control group was applied with paraffin oil instead of imiquimod. Every day, the disease scores were recorded from back skin (Table S5).

Table S5. Disease scoring method for assessing back skin in mice.

Score	Erythema	Scaling	Skin thickness
0	Normal	Normal	Normal
1	Slight	Slight	Slight
2	Moderate	Moderate	Moderate
3	Important	Important	Important
4	Very important	Very important	Very important

X-ray crystallography

Expression and purification of IRAK4 for X-ray crystallography

A synthetic gene fragment encoding IRAK4 amino acids F165-S460 with three mutants K400A/E401A/E402A was integrated into the baculovirus pVL1393 transfer vector behind a GST-Tag and a Thrombin cleavage site. The GST-fusion protein was expressed in SF9 insect cells infected with moi 1 for 48 h. The overall purification of intermediates was carried out at 4°C or on ice. Cells were resuspended in lysis buffer (50 mM Tris pH 8.0, 120 mM NaCl, 10% glycerol, 0,5%NP-40, 5 mM DTT) supplemented with cOmplete protease inhibitor cocktail (Roche Applied Science). The insoluble fraction was removed by centrifugation (150.000 g, 45 min). GST-tagged IRAK4 was captured on GST-Agarose 4B (GE Healthcare) by batch binding. GST resin was washed with buffer (50mM Tris pH 8.0, 60m M NaCl, 10% glycerol, 1 mM DTT). The IRAK4 protein was eluted by column cleavage over night with thrombin. Elution fractions were applied on a Resource 15Q (GE Healthcare) column and eluted by salt gradient (50mM Tris pH 8.0, 60 mM – 1 M NaCl, 10% glycerol, 1 mM DTT). Elution fractions

from the IEX were pooled and further purified by size-exclusion chromatography using a Superdex 75 26/60 column (GE Healthcare) pre-equilibrated with running buffer (50 mM HEPES pH 7.6, 250 mM NaCl, 10 % glycerol, 2 mM DTT). The monomeric peak fractions were pooled and concentrated with a 10-kDa Amicon Ultra-4 concentrator (Millipore) to 10 mg/mL and freshly used in crystallization experiments.

Crystallization and complex formation

Two different crystallization routes were used to obtain co-complex structures:

a) Co-crystallization: Compound **38** and compound **5** were dissolved giving 100 mM DMSO stock solutions. Prior co-crystallization inhibitors were added to the protein to a final concentration of 2 mM. The complexes were incubated for 2 h on ice and crystallization was performed using vapor diffusion by hanging drop using equal volumes of protein solution and reservoir solution (0.1 M sodium acetate buffer at pH 4.9, 1.5 – 1.7 M ammonium citrate and 0.02 M hexaaminecobalt(III)chloride). Crystals of dimensions of 0.1 – 0.2 mm appeared within 1 – 3 days at 20 °C.

b) Back-soaking: An inhouse IRAK4 inhibitor was used as a tool to obtain suitable crystals for back-soaking experiments. The tool compound was diluted in DMSO to give a 100 mM stock solution. The tool compound was then added to the protein to a final concentration of 5 mM and the complex was incubated for 2 h on ice. Crystallization was performed using vapor diffusion in hanging drops using equal volumes of protein solution and reservoir solution (0.1 M sodium acetate buffer at pH 4.9 and 2.13 – 2.145 M sodium malonate). IRAK4 seeds were added to the final drop. Crystals with a size of ~0.1 – 0.3 mm grew after 1 – 3 days at 20 °C.

IRAK4 crystals were washed three times in reservoir solution overnight to wash out the tool compound. Compound **23**, compound **40**, and compound **16** were dissolved giving 100 mM DMSO stock solutions. DMSO stocks were diluted with reservoir solution to a final concentration of 5 mM of inhibitors. Crystals of IRAK4 were soaked for 3 – 4 days in this solution at 20 °C.

Crystals of IRAK4: compound **41** were grown using Proteros Biostructures.

Data collection and refinement for IRAK4 co-complex structures:

Crystals were flash-frozen in liquid nitrogen in the mother liquor prior to data collection. All data was collected at different synchrotron sources. Data of compound **38** and compound **5** was collected at Hamburg P14, data for compound **23**, compound **40** and compound **16** was

collected at Bessy beamline 14.1. Data of compound **41** was collected at Swiss Light Source beamline PXII by Proteros Biostructures. All data were processed using XDS[2]. Table S6 contains data collection statistics.

The structures were determined via Molecular Replacement using Molrep[3] with protein coordinates from an inhouse structure as the search model. The structure of compound **41** was solved using a previously solved X-ray structure. Refinement was carried out using Refmac[4], model fitting was completed using COOT.[5] The refinement topology parameter file for the inhibitors were generated using ProDrg[6] and were docked into the electron density within COOT. The models contain two IRAK4 molecules in the asymmetric unit, only **41** consists of four IRAK4 molecules in the asymmetric unit. The crystallographic data for the six structures have been deposited within the RCSB Protein Data Bank (PDB) with access codes 8ATB, 8ATL, 8ATN, 8BR5, 8BR6, 8BR7. Refinement statistics are shown in Table S6.

Table S6. Data collection and refinement statistics.

**Compound Number**	**5**	**16**	**23**	**38**	**40**	**41**
PDB Id	8BR7	8ATB	8ATL	8ATN	8BR6	8BR5
**Data collection and processing**						
Wavelength [Å]	0.9763	0.9184	0.9184	0.9763	0.9184	1.000
Space group (no.)	I222	I222	I222	I222	I222	C2
Unit cell parameters, *a*, *b*, *c* [Å], beta [°]	87.258<br>117.97<br>139.86	87.200<br>117.500<br>136.600	87.864<br>118.789<br>138.136	88.117<br>118.798<br>140.363	87.725<br>18.573<br>138.709	143.21<br>141.20<br>87.92<br>124.5
Resolution limit [Å]	19.77 – 2.12<br>(2.25 – 2.12)	48.89 – 2.34<br>(2.48 – 2.34)	46.35 – 2.46<br>(2.60 – 2.46)	49.88 – 2.17<br>(2.30 – 2.17)	46.3 – 2.17<br>(2.30 – 2.17)	90.58 – 2.70<br>(2.95 – 2.70)
No. of reflections	474480 (74103)	178315 (28830)	146185 (22131)	540629 (86294)	228440 (36772)	151292 (34200)
No. of unique reflections	35635 (5751)	30087 (4796)	26561 (4006)	39306 (6285)	38659 (6104)	39488 (9173)
Multiplicity	6.49 (6.20)	5.92 (6.01)	5.50 (5.52)	13.75 (13.73)	5.01 (6.02)	4.42 (4.30)
$I/\sigma(I)$	16.69 (1.66)	16.25 (2.44)	17.30 (3.60)	13.77 (1.11)	10.85 (1.71)	16.80 (3.58)
R_{meas} [%]	11.5 (205.4)	9.5 (83.0)	7.4 (49.1)	11.9 (244.9)	13.8 (114.7)	9.4 (52.3)
CC(1/2)	99.9 (70.1)	99.9 (80.5)	99.9 (92.6)	99.9 (76.7)	99.8 (65.1)	99.8 (88.2)
Completeness [%]	86.2 (87.8)	99.8 (99.6)	99.8 (100.0)	99.9 (100)	99.7 (98.6)	99.3 (99.3)
**Refinement**						
R_{work}/R_{free} [%]	23.87 / 28.79	21.23/ 20.01	19.88 / 24.29	23.84 / 30.59	21.33 / 25.13	21.46 / 25.41
RMSD bond length [Å]	0.0035	0.0021	0.0026	0.0136	0.0028	0.0035
RMSD bond angles [deg]	1.239	1.157	1.274	1.767	1.220	1.253
B factors [$Å^2$]	60.24	55.62	56.28	77.04	48.70	57.76
Ramachandran favored (%)	95.0	95.8	96.9	93.0	96.9	96.2
Ramachandran allowed (%)	4.4	3.8	3.1	6.2	2.6	3.4
Ramachandran outliers (%)	0.6	0.4	0.0	0.7	0.5	0.4

Values in brackets refer to the highest resolution shell.

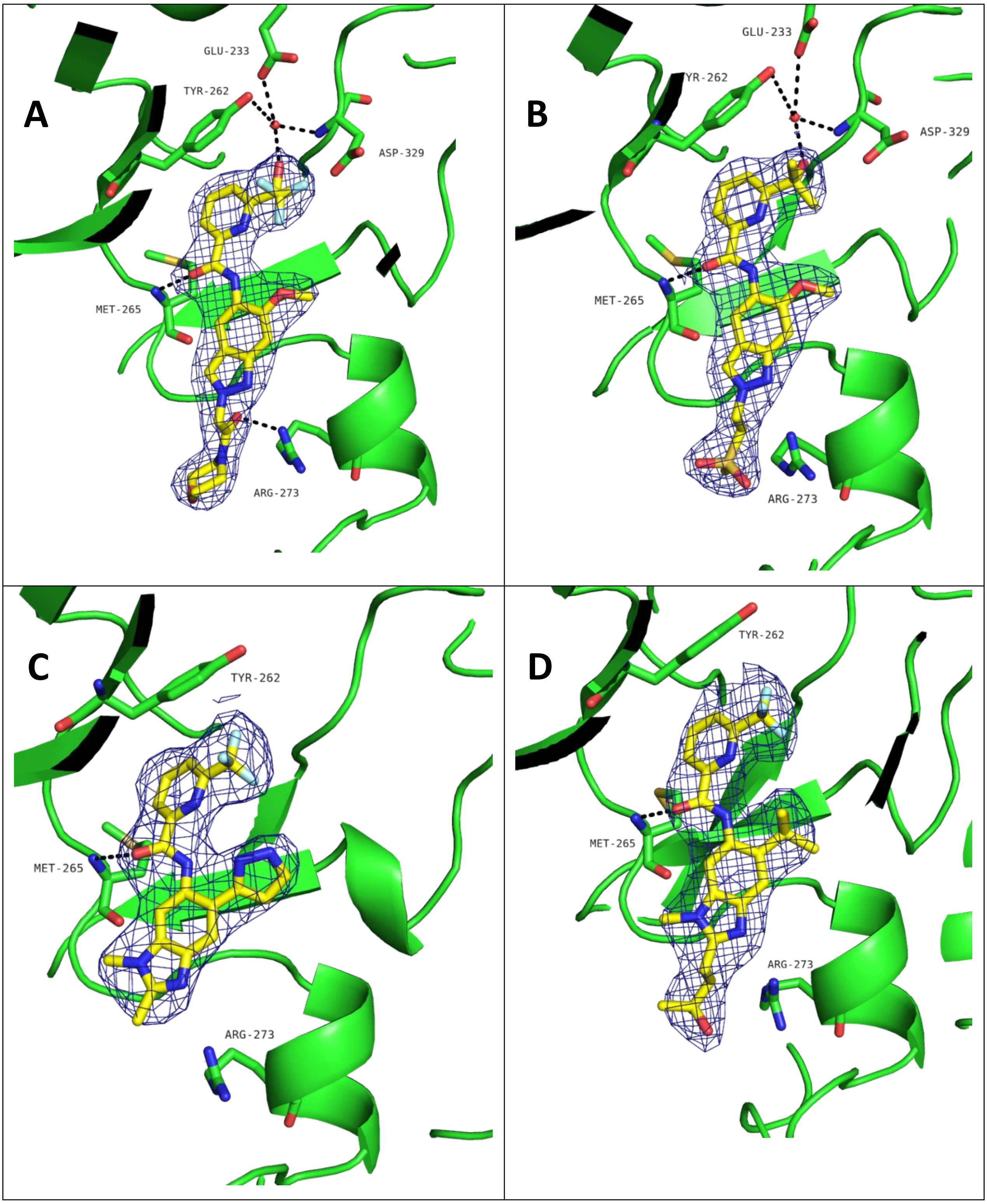

Figure S3: Cocrystal structures of indazole-based compounds **23** (A) and **40** (B), as well as methyl-benzimidazole-based compounds **41** (C) and **38** (D) with the human IRAK4 kinase domain. The general binding mode of the series – the amide carbonyl oxygen atom engaging in a hydrogen bond with the hinge residue Met265 – was stable across the series and throughout the chemistry program (see also Figures 3 and 5). While no experimental binding

modes for the two clinical candidate molecules are available, all substructures contain local binding interactions that have been characterized through X-ray data.

Computational studies

Modelling studies were performed using the Schrodinger Maestro Suite (versions 9.2 to 10.4). For docking, Glide[7, 8] (versions 5711 to 69017) was used, and protein structures were prepared using the protein preparation wizard with default parameters, with ligand protonation states being manually adapted as required. To ensure a comprehensive sampling of the space of docking solutions, GOLD[9] was employed as a second, algorithmically complementary docking tool, using the same protein input structures prepared within Maestro. The preparation of 3D ligand structures for docking employed an inhouse Pipeline Pilot [10] script that utilizes Corina[11] for 3D structure generation and SimPlus ADMET predictor[12] for the assignment of protonation states.

Matched molecular pairs where identified using an inhouse protocol based on Pipeline Pilot[10].

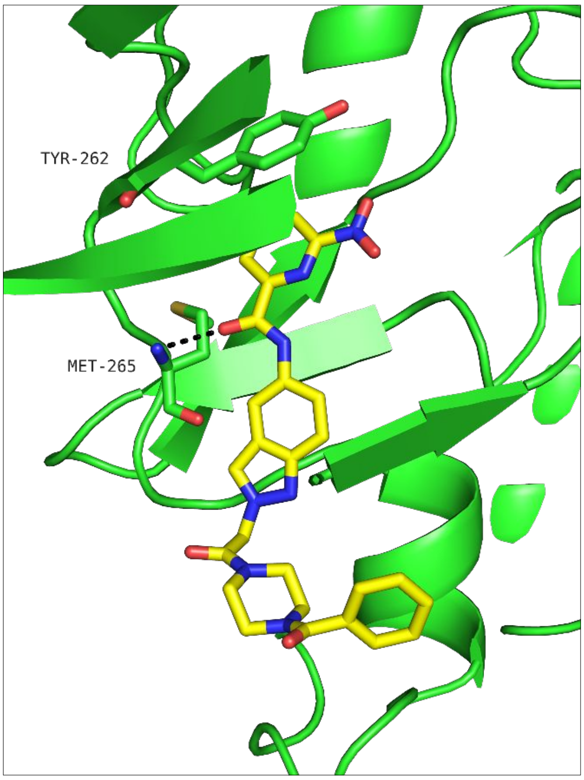

Figure S4: Orientation of the docking pose of compound **5** in the IRAK4 pocket of PDB 2NRU, A chain, qualitatively reproduced with current software (Glide SP as part of maestro version 13.7.125). The original 3D file is no longer available. The hypothesized binding mode involves a single hydrogen bond to the hinge region (Met265) and aromatic-aromatic interactions with the gatekeeper Tyr262.

Experimental section: synthetic chemistry

General Methods and Materials

Commercially available reagents and anhydrous solvents were used as supplied without further purification. A Biotage Initiator Classic microwave reactor (Uppsala, Sweden) was used for reactions conducted in a microwave oven. Reactions were monitored by thin layer chromatography (TLC) and UPLC using either analytical method A (Instrument: Waters Acquity UPLCMS SingleQuad; Column: Acquity UPLC BEH C18 1.7 µm, 50 mm x 2.1 mm; eluent A: water + 0.1 vol % formic acid (99%), eluent B: acetonitrile; gradient: 0–1.6 min 1–99% B, 1.6–2.0 min 99% B; flow 0.8 ml/min; temperature: 60 °C; DAD scan: 210–400 nm), analytical method B (Instrument: Waters Acquity UPLCMS SingleQuad; Column: Acquity UPLC BEH C18 1.7 µm, 50 x 2.1mm; eluent A: water+0.2 vol % aqueous ammonia (32%), eluent B: acetonitrile; gradient: 0–1.6 min 1–99% B, 1.6–2.0 min 99% B; flow 0.8 ml/min; temperature: 60 °C; DAD scan: 210–400 nm), analytical method C (Instrument: Waters Acquity UPLCMS SingleQuad; Column: Acquity UPLC BEH C18 1.7 50mm × 2.1mm; eluent A: water +0.1 vol % formic acid (99%), eluent B: acetonitrile; gradient: 0–1.6 min 1–99% B, 1.6–2.0 min 99% B; flow 0.8 ml/min; temperature: 60 °C; DAD scan: 210–400 nm), or analytical method D (Instrument: Waters Acquity UPLCMS SingleQuad; Colum: Acquity UPLC BEH C18 1.7 50mm × 2.1mm; eluent A: water+0.2 vol % aqueous ammonia (32%), eluent B: acetonitrile; gradient: 0–1.6 min 1–99% B, 1.6–2.0 min 99% B; flow 0.8 ml/min; temperature: 60 °C; DAD scan: 210–400 nm). Analytical thin layer chromatography (TLC) was carried out on aluminum-backed plates coated with Merck Kieselgel 60 F254 (Merck KGaA, Darmstadt, Germany), with visualization under UV light at 254 nm. Flash chromatography was carried out using a Biotage Isolera One system with a 200−400 nm variable detector. Preparative HPLC was carried out with a Waters AutoPurification MS Single Quad system; column: Waters XBridge C18 5µm, 100 mm × 30 mm; basic conditions: eluent A, water+0.2 vol % aq NH_3 (32%); eluent B, MeCN; acidic conditions: eluent A: water+0.1 vol % formic acid, eluent B: MeCN; DAD scan, 210−400 nm. NMR spectra were recorded at rt (22 ± 1 °C), unless otherwise noted, on Bruker Avance III HD spectrometers. ^1H NMR spectra were obtained at 300, 400, 500, or 600 MHz. ^1H NMR data are reported as follows: chemical shift (δ) in ppm, multiplicity (s = singlet, d = doublet, t = triplet, q =quartet, br = broad, m = multiplet), integration, and assignment. Low-resolution mass spectra (electrospray ionization, ESI) were obtained via HPLC-MS (ESI) using a Waters Acquity UPLC system equipped with an SQ 3100 mass detector. The purity of all target compounds

if not otherwise mentioned was at least 95%, as determined by UPLC-MS. Compound names were generated using ACD/name software.

Abbreviations

br, broad; brine, saturated aqueous sodium chloride solution; CAS, Chemical Abstracts Service; DAD, diode array detector; DIPEA, *N*,*N*-diisopropylethylamine (Hünig's base); DMF, *N*,*N*-dimethylformamide; DMSO, dimethyl sulfoxide; EDC, 1-ethyl-3-(3-dimethylaminopropyl)carbodiimide hydrochloride; ELSD, evaporative light scattering detector; eq., equivalent; ESI(neg), electrospray ionization negative ion mode; ESI(pos), electrospray ionization positive ion mode; HATU, 1-[bis(dimethylamino)methylene]-1H-1,2,3-triazolo[4,5-b]pyridinium 3-oxid hexafluorophosphate; HCl, hydrochloric acid; HOBt, 1-hydroxybenzotriazole hydrate; NMR, nuclear magnetic resonance spectroscopy: chemical shifts (δ) are given in ppm. The chemical shifts were corrected by setting the DMSO signal to 2.50 ppm unless otherwise stated; sat., saturated; SQD, single-quadrupole-detector; TEA, triethylamine; UPLC, ultra-performance liquid chromatography.

Synthesis of compound 6

__Intermediate 6-a__

***tert*-Butyl-1H-indazol-5-ylcarbamate**

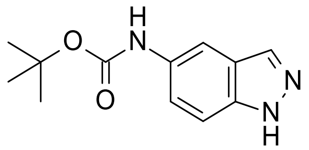

37 mL of DIPEA and 41.8 g (191.5 mmol) of di-*tert*-butyl dicarbonate were added to 25.5 g (192 mmol) of 1H-indazole-5-amine (CAS Number 19335-11-6) in 300 mL of THF and the mixture was stirred at 25°C for 24 h. The mixture was concentrated affording 44.6 g (95% yield) of the title compound.
MS (ESIpos): m/z = 234 $(M+H)^+$
^1H NMR (DMSO-d_6, 300 MHz) δ 1.44 (s, 9H), 7.24–7.46 (m, 2H), 7.84 (s, 1H), 7.92 (s, 1H), 9.24 (br. s., 1H), 12.86 (br. s., 1H).

__Intermediate 6-b__

Ethyl {5-[(*tert*-butoxycarbonyl)amino]-2H-indazol-2-yl}acetate

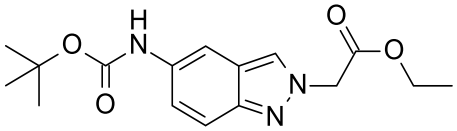

10.5 g (76.3 mmol) of potassium carbonate and 4.67 mL (42.0 mmol) of ethyl bromoacetate were added to 8.90 g (38.1 mmol) of *tert*-butyl 1H-indazol-5-ylcarbamate in 80 mL of DMF and the mixture was stirred at 80 °C for 24 h. The mixture was diluted with water and extracted with ethyl acetate, the organic phase was washed with water and brine, dried, and concentrated and the residue was purified by column chromatography on silica gel (hexane/ethyl acetate). This gave 2.4 g of the title compound as the main component as a mixture with *tert*-butyl 1H-indazol-5-ylcarbamate (starting material).
^1H NMR (CHLOROFORM-d, 500 MHz, selected signals) δ 1.28 (t, 3H, *J*=7.2 Hz), 4.25 (q, 2H, *J*=7.3 Hz), 5.16 (s, 2H), 7.03 (dd, 1H, *J*=1.9, 9.2 Hz), 7.62 (d, 1H, *J*=8.9 Hz).

__Intermediate 6-c__

{5-[(*tert*-Butoxycarbonyl)amino]-2H-indazol-2-yl}acetic acid

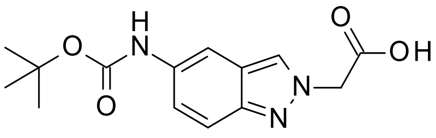

5.00 g (15.6 mmol) of ethyl {5-[(*tert*-butoxycarbonyl)amino]-2H-indazol-2-yl}acetate were dissolved in 50 mL of THF and 5 mL of ethanol. A solution of 6.57 g (15.6 mmol) of lithium hydroxide monohydrate in 20 mL of water was then added and the mixture was stirred at 25 °C for 24 h, diluted with water, and acidified to pH 4 by addition of aqueous hydrochloride solution (1M). The mixture was partly concentrated. The solid was filtered, washed with water and diethylether, and dried *in vacuo* to give 4.1 g (89% yield) of the title compound.

MS (ESIpos): m/z = 292 $(M+H)^+$

<u>Intermediate 6-d</u>

***tert*-Butyl {2-[2-(4-benzoylpiperazin-1-yl)-2-oxoethyl]-2H-indazol-5-yl}carbamate**

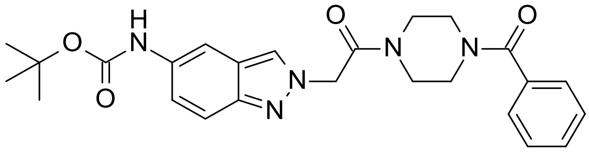

2.53 g (8.69 mmol) of {5-[(*tert*-butoxycarbonyl)amino]-2H-indazol-2-yl}acetic acid were stirred in 50 mL of THF and 1.33 g (8.69 mmol) of HOBt, 3.33 g (17.4 mmol) of EDC, and 3.6 mL (26 mmol) of TEA were added and the suspension was stirred at 25 °C overnight. The mixture was diluted with water and sat. aqueous sodium bicarbonate solution and concentrated until precipitation of a solid. The solid was filtered off, washed with water and ethyl acetate, and dried *in vacuo*.

MS (ESIpos): m/z = 464 $(M+H)^+$

^{1}H NMR (DMSO-d_6, 400 MHz) δ 1.45 (s, 9H), 3.30–3.78 (m, 8H), 5.41 (br. s., 2H), 7.18 (dd, 1H, *J*=1.9, 9.2 Hz), 7.35–7.50 (m, 6H), 7.82 (br. s., 1H), 8.11 (s, 1H), 9.18 (s, 1H).

<u>Intermediate 6-e</u>

2-(5-Amino-2H-indazol-2-yl)-1-(4-benzoylpiperazin-1-yl)ethanone

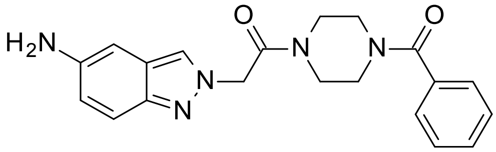

A mixture of 4.20 g (9.06 mmol) of *tert*-butyl {2-[2-(4-benzoylpiperazin-1-yl)-2-oxoethyl]-2H-indazol-5-yl}carbamate and 6.98 mL (90.6 mmol) of TFA in 50 mL DCM was stirred at rt overnight and carefully poured into an aqueous sat. sodium bicarbonate solution. The mixture was stirred and extracted with DCM three times. The combined organic layers were washed with brine, filtered with a water-repellent filter, and concentrated to give 3.27 g (99% yield) of the title compound.

MS (ESIpos): m/z = 364 $(M+H)^+$

^{1}H NMR (DMSO-d_6, 300 MHz) δ 3.36–3.80 (m, 8H), 4.78 (s, 2H), 5.33 (br. s., 2H), 6.55 (d, 1H, *J*=1.3 Hz), 6.74 (dd, 1H, *J*=2.1, 9.0 Hz), 7.30 (d, 1H, *J*=9.0 Hz), 7.38–7.53 (m, 5H), 7.81 (s, 1H).

Compound 6 (step 6-f)

***N*{2-[2-(4-benzoylpiperazin-1-yl)-2-oxoethyl]-2H-indazol-5-yl}-6-(trifluoromethyl)pyridine-2-carboxamide**

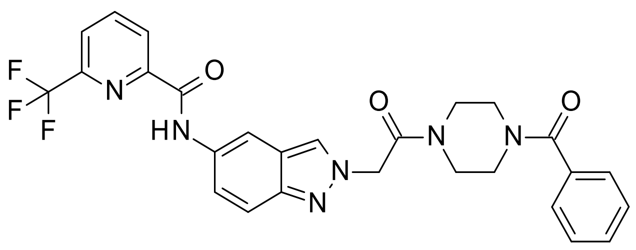

230 mg (1.21 mmol, 1.2 eq.) of 6-(trifluoromethyl)pyridine-2-carboxylic acid, 385 mg (2.01 mmol) of EDC, 154 mg (1.00 mmol) HOBt, and 0.42 mL of TEA were added to a mixture of 365 mg (1.00 mmol) of 2-(5-amino-2H-indazol-2-yl)-1-(4-benzoylpiperazin-1-yl)ethanone in THF (6 mL) and the mixture was stirred at rt overnight. Water was added and the mixture was partly concentrated. The solid was filtered off and washed three times with water and three times with diethylether and dried *in vacuo* to give 488 mg (91%) of the title compound.

LC-MS (method A): Rt = 1.09 min; MS (ESIpos): m/z = 537 $[M+H]^+$

^{1}H NMR (DMSO-d_6, 400 MHz) δ 3.43–3.71 (m, 7H), 5.49 (br s, 2H), 7.42–7.49 (m, 5H), 7.54–7.62 (m, 2H), 8.16 (dd, 1H, *J*=1.3, 7.6 Hz), 8.29–8.41 (m, 4H), 10.36 (s, 1H).

Synthesis of compound 7

Intermediate 7-a

***tert*-Butyl (6-methyl-1H-indazol-5-yl)carbamate**

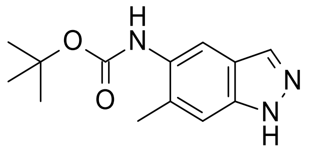

10.3 g (70.0 mmol) of 6-methyl-1H-indazole-5-amine (CAS Number 81115-45-9) was suspended in 150 mL of THF; 13.4 mL (80.0 mmol) of DIPEA was added and the mixture was cooled to 0 °C. After the addition of 5.52 g (25.3 mmol) of di-*tert*-butyl dicarbonate at 0 °C, the mixture was then stirred at 25 °C for 18 h. The mixture was concentrated, giving 17.6 g of a crude product which was used without purification.

MS (ESIpos): m/z = 248 $(M+H)^+$

<u>Intermediate 7-b</u>

Ethyl {5-[(*tert*-butoxycarbonyl)amino]-6-methyl-2H-indazol-2-yl}acetate

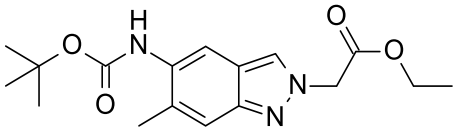

10.0 g (40.4 mmol) of *tert*-butyl (6-methyl-1H-indazol-5-yl)carbamate were stirred with 9.00 mL (80.9 mmol) of ethyl bromoacetate in 75 mL of THF in the presence of 17.1 mL (80.9 mmol) of *N*,*N*-dicyclohexylmethylamine at 70 °C for 24 h. The precipitate was filtered and washed twice with ethyl acetate. Water was added to the filtrate and the organic phase was separated and extracted twice with ethyl acetate. The combined organic phases were washed with 1 M HCl, sat. sodium bicarbonate solution, and brine and concentrated. The residue was purified using the Isolera® flash purification system (Biotage) (mobile phase: hexane/ethyl acetate. The combined product fractions were concentrated and dried. This gave 8.90 g (42% yield) of the title compound.

In a second experiment, 213 mg of the title compound were obtained analogously from 2.00 g of *tert*-butyl (6-methyl-1H-indazol-5-yl)carbamate using 2.24 g of potassium carbonate instead of *N*,*N*-dicyclohexylmethylamine at 80 °C in DMF, with two successive purifications on silica gel (hexane/ethyl acetate).

MS (ESIpos): m/z = 334 $(M+H)^+$

^1H NMR (DMSO-d_6, 600 MHz) δ 1.21 (t, 3H, *J*=7.2 Hz), 1.46 (s, 9H), 2.28 (s, 3H), 4.16 (q, 2H, *J*=7.2 Hz), 5.34 (s, 2H), 7.38 (d, 1H, *J*=0.8 Hz), 7.57 (s, 1H), 8.25 (d, 1H, *J*=0.8 Hz), 8.40 (s, 1H).

<u>Intermediate 7-c</u>

{5-[(*tert*-Butoxycarbonyl)amino]-6-methyl-2H-indazol-2-yl}acetic acid

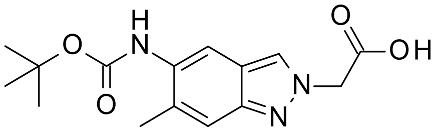

10.7 g (254 mmol) of lithium hydroxide monohydrate dissolved in 50 mL of water were added to 10.6 g (25.4 mmol, 80%) of ethyl {5-[(*tert*-butoxycarbonyl)amino]-6-methyl-2H-indazol-2-yl}acetate in 100 mL of THF and 10 mL of ethanol and the mixture was stirred. This resulted in the precipitation of a solid. After 18 h, the reaction mixture was diluted with water and acidified to pH 4 using aqueous HCl solution (2M) and the solid was filtered off, washed with water and diethylether, and dried. This gave 6.98 g (87% yield) of the title compound.
MS (ESIpos): m/z = 306 $(M+H)^+$
^1H NMR (DMSO-d_6, 300 MHz) δ 1.44 (s, 9H), 2.25 (s, 3H), 4.78 (s, 2H), 7.32 (s, 1H), 7.49 (s, 1H), 8.10 (s, 1H), 8.35 (s, 1H).

<u>Intermediate 7-d</u>

***tert*-Butyl {2-[2-(4-benzoylpiperazin-1-yl)-2-oxoethyl]-6-methyl-2H-indazol-5-yl}carbamate**

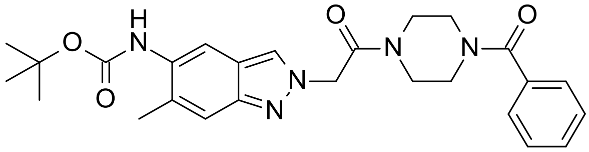

181 mg (0.59 mmol) of {5-[(*tert*-butoxycarbonyl)amino]-6-methyl-2H-indazol-2-yl}acetic acid and 169 mg (0.89 mmol) of phenyl(piperazin-1-yl)methanone were initially charged in 5 mL of THF and 0.5 mL of DMF. 91 mg (0.59 mmol) of HOBt, 227 mg (1.19 mmol) of EDC, and 0.25 mL (1.79 mmol) of TEA were added and the mixture was stirred at 25 °C for 18 h. The mixture was diluted with water and ethyl acetate and the precipitated solid was filtered off, washed with water and diethylether, and dried under reduced pressure. This resulted in 248 mg (85% yield) of the title compound.
MS (ESIpos): m/z = 478 $(M+H)^+$

^1H NMR (DMSO-d_6, 400 MHz) δ 1.42 (s, 9H), 2.24 (s, 3H), 3.32–3.82 (m, 8H), 5.41 (br. s., 2H), 7.33 (s, 1H), 7.38–7.48 (m, 5H), 7.52 (s, 1H), 8.12–8.16 (m, 1H), 8.35 (s, 1H).

<u>**Intermediate 7-e**</u>

2-(5-Amino-6-methyl-2H-indazol-2-yl)-1-(4-benzoylpiperazin-1-yl)ethanone

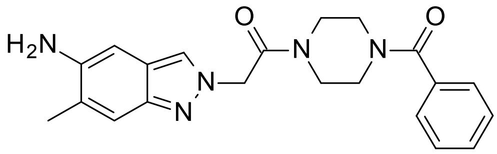

0.3 mL (3.89 mmol) of TFA was added to 247 mg (0.52 mmol) of *tert*-butyl {2-[2-(4-benzoylpiperazin-1-yl)-2-oxoethyl]-6-methyl-2H-indazol-5-yl}carbamate in 5 mL of DCM and the mixture was stirred at 25 °C for 18 h. Another 0.3 mL (3.89 mmol) of TFA was then added and the mixture was stirred for 18 h, poured into sat. sodium bicarbonate solution and extracted three times with DCM. The mixture was concentrated resulting in 223 mg of the title compound as a crude product.
MS (ESIpos): m/z = 378 $(M+H)^+$
^1H NMR (DMSO-d_6, 400 MHz) δ 2.15 (s, 3H), 3.29–3.75 (m, 8H), 4.53 (s, 2H), 5.28 (br. s., 2H), 6.63 (s, 1H), 7.17 (s, 1H), 7.37–7.47 (m, 5H), 7.75–7.79 (m, 1H).

<u>**Compound 7 (step 7-f)**</u>

N-{2-[2-(4-Benzoylpiperazin-1-yl)-2-oxoethyl]-6-methyl-2H-indazol-5-yl}-6-(trifluoromethyl)pyridine-2-carboxamide

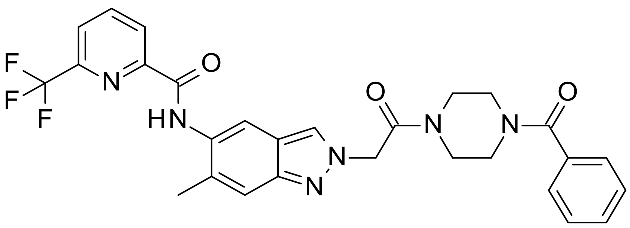

44 mg (0.29 mmol) of HOBt, 11 mg (0.58 mmol, 2 eq.) of EDC and 0.12 mL (3 eq.) of TEA were added to 109 mg (0.29 mmol) 2-(5-amino-6-methyl-2H-indazol-2-yl)-1-(4-benzoylpiperazin-1-yl)ethanone and 83 mg 6-(trifluoromethyl)pyridine-2-carboxylic acid in 2.5 mL THF and 0.5 mL DMF and the mixture was stirred at rt overnight. Water and ethyl acetate were added, and the resulting suspension was filtered; the solid was washed with water and diethylether and dried *in vacuo* affording 108 mg (0.20 mmol, 68% yield) of the title compound.

LC-MS (method A): Rt = 1.16 min; MS (ESIpos): m/z = 551 $[M+H]^+$

^{1}H NMR (DMSO-d_6, 400 MHz) δ 2.38 (s, 3H), 3.32–3.77 (8H), 5.45 (br. s., 2H), 7.39–7.49 (m, 6H), 8.15–8.21 (m, 2H), 8.24 (s, 1H), 8.33–8.43 (m, 2H), 10.11 (s, 1H).

Synthesis of compound 8

Intermediate 8-a

***tert*-Butyl (6-fluoro-1H-indazol-5-yl)carbamate**

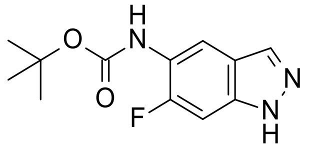

Similar to the synthesis of compound 7 (intermediate 7-a), 4.96 g (32.8 mmol) of 6-fluoro-1H-indazole-5-amine (CAS Number 709046-14-0), 7.16 g (32.8 mmol) of di-*tert*-butyl dicarbonate and 6.28 mL (36 mmol) of DIPEA were dissolved in 51 mL of THF and stirred at 25 °C for 20 h. This gave 5.72 g (69% yield) of the title compound.

MS (ESIpos): m/z = 252 $(M+H)^+$

^{1}H NMR (DMSO-d_6, 300 MHz) δ 1.45 (s, 9H), 7.34 (d, 1H, *J*=10.5 Hz), 7.81 (m, 1H), 8.03 (s, 1H), 8.80 (s, 1H), 13.08 (s, 1H).

Intermediate 8-b

Ethyl {5-[(*tert*-butoxycarbonyl)amino]-6-fluoro-2H-indazol-2-yl}acetate

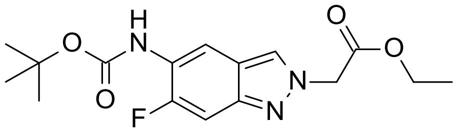

5.44 g (21.6 mmol) of *tert*-butyl (6-fluoro-1H-indazol-5-yl)carbamate, 4.80 mL (43.3 mmol) of ethyl bromoacetate, and 9.18 mL (43.3 mmol) of *N*,*N*-dicyclohexylmethylamine in 30 mL of THF were stirred for 24 h. Then, an additional 0.96 mL (8.6 mmol) of ethyl bromoacetate and 1.84 mL (8.6 mmol) of *N*,*N*-dicyclohexylmethylamine were added twice, once after 24 h and again after 48 h. The mixture was concentrated, taken up in DCM, washed with water, dried, and concentrated. Purification by column chromatography using the Isolera® flash purification system (Biotage) (mobile phase: hexane/DCM/ethyl acetate) gave 3.75 g (47% yield) of the title compound.

MS (ESIpos): m/z = 338 $(M+H)^+$

^1H NMR (DMSO-d_6, 300 MHz) δ 1.20 (t, 3H, *J*=7.2 Hz), 1.46 (s, 9H), 4.17 (q, 2H, *J*=7.2 Hz), 5.36 (s, 2H), 7.37 (d, 1H), 7.84 (d, 1H), 8.36 (s, 1H), 8.80 (s, 1H).

Intermediate 8-c

Ethyl (5-amino-6-fluoro-2H-indazol-2-yl)acetate

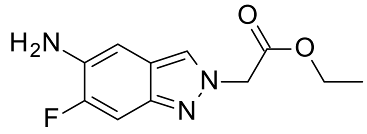

Similar to the preparation of compound 7 (Intermediate 7-e), 1.1 g (3.3 mmol) of ethyl {5-[(*tert*-butoxycarbonyl)amino]-6-fluoro-2H-indazol-2-yl}acetate were reacted with 1.92 mL (24.9 mmol) of TFA in 11 mL of DCM. Work-up gave 790 mg (100% yield) of the title compound.

MS (ESIpos): m/z = 238 $(M+H)^+$

^1H NMR (DMSO-d_6, 300 MHz) δ 7.99 (d, 1H, *J*=0.8 Hz), 7.22 (s, 1H), 7.18 (s, 1H), 6.80 (d, 1H, *J*=8.9 Hz), 5.23 (s, 2H), 4.92 (s, 2H), 4.15 (q, 2H, *J*=7.2 Hz), 1.20 (t, 3H, *J*=7.2 Hz)

Intermediate 8-d

Ethyl [6-fluoro-5-({[6-(trifluoromethyl)pyridine-2-yl]carbonyl}amino)-2H-indazol-2-yl]acetate

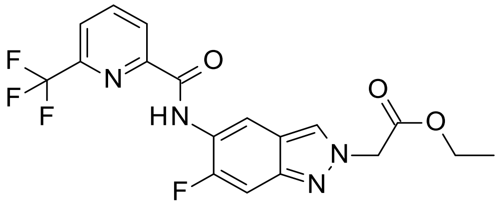

221 mg (1.16 mmol) of 6-(trifluoromethyl)pyridine-2-carboxylic acid, 177 mg (1.16 mmol) of HOBt, and 444 mg (2.32 mmol) of EDC in 5.5 mL of DMF were stirred at 25 °C for 30 min. 250 mg (1.05 mmol) of ethyl (5-amino-6-fluoro-2H-indazol-2-yl)acetate were added and the mixture was stirred at 25 °C for 30 min. The mixture was poured into 150 mL of water, filtered off with suction, washed with water, and dried, producing 366 mg (84% yield) of the title compound.

MS (ESIpos): m/z = 411 $(M+H)^+$

^1H NMR (DMSO-d_6, 300 MHz) δ 1.22 (t, 3H, *J*=7.1 Hz), 4.18 (q, 2H, *J*=7.2 Hz), 5.41 (s, 2H), 7.55 (d, 1H, *J*=11.6 Hz), 8.21 (m, 1H), 8.36–8.51 (m, 4H), 10.27 (m, 1H).

Intermediate 8-e

[6-Fluoro-5-({[6-(trifluoromethyl)pyridin-2-yl]carbonyl}amino)-2H-indazol-2-yl]acetic acid

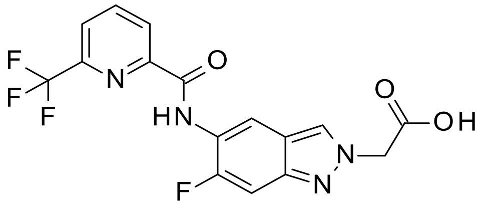

381 mg (0.93 mmol) of ethyl [6-fluoro-5-({[6-(trifluoromethyl)pyridin-2-yl]carbonyl}amino)-2H-indazol-2-yl]acetate were suspended in 9.2 mL of THF and 0.45 mL of ethanol and a solution of 222 mg (9.3 mmol) of lithium hydroxide in 2.3 mL of water was then added. The mixture was stirred at 25 °C for 30 min and then acidified to pH 2 with ice cooling using 2N HCl. 10 mL of water were added, and the precipitate was filtered off with suction. This resulted in 332 mg (93% yield) of the title compound.
MS (ESIpos): m/z = 383 $(M+H)^+$
^1H NMR (DMSO-d_6, 300 MHz) δ 5.30 (s, 2H), 7.55 (d, 1H), 8.22 (m, 1H), 8.34–8.54 (m, 4H), 10.26 (m, 1H), 13.30 (s br, 1H).

Compound 8 (step 8-f)

N-{2-[2-(4-Benzoylpiperazin-1-yl)-2-oxoethyl]-6-fluoro-2H-indazol-5-yl}-6-(trifluoromethyl)pyridine-2-carboxamide

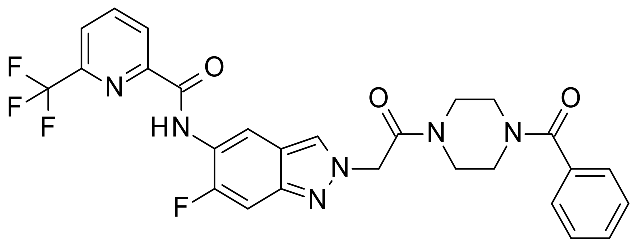

32 mg (0.21 mmol) of HOBt and 80 mg (0.42 mmol, 2 eq.) of EDC were added to 80 mg (0.21 mmol) of [6-fluoro-5-({[6-(trifluoromethyl)pyridin-2-yl]carbonyl}amino)-2H-indazol-2-yl]acetic acid in 3 mL THF and 330 µl DMF. After stirring for 30 min at rt, 60 mg (0.31 mmol) of phenyl(piperazin-1-yl)methanone were added and the mixture was stirred for 30 min at rt and poured into 50 mL water. The resulting solid was filtered off using suction, washed with water, and dried to provide 109 mg (94 yield) of the title compound.
LC-MS (method A): Rt = 1.15 min; MS (ESIpos): m/z = 555 $[M+H]^+$

^{1}H NMR (DMSO-d_6, 300 MHz) δ 3.38–3.75 (m, 8H), 5.51 (s, 2H), 7.40–7.56 (m, 6H), 8.19–8.26 (m, 1H), 8.35–8.49 (m, 4H), 10.24 (m, 1H).

Synthesis of compound 9

Intermediate 9-a

***tert*-Butyl 5-amino-6-chloro-1H-indazole-1-carboxylate**

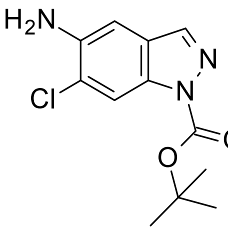

2.1 mL (11.8 mmol) of DIPEA and 2.34 g (10.7 mmol) of di-*tert*-butyl dicarbonate were added to 1.80 g (10.7 mmol) of 6-chloro-1H-indazole-5-amine (CAS Number 221681-75-0) in 18 mL of THF and the mixture was stirred at 25 °C for 18 h. The mixture was concentrated, and the residue was taken up in ethyl acetate and, during concentration, adsorbed on ISOLUTE. The ISOLUTE was applied to a Biotage SNAP cartridge (100 g; KP-Sil) that was pre-equilibrated with hexane and chromatography was carried out using the Isolera® flash purification system (Biotage) (mobile phase: hexane/ethyl acetate; flow rate: 50 ml/min; gradient: isocratic 100:0 [5 min], 100:0->75:25 [20 min], isocratic 75:25 [5min], 75:25->50:50 [15 min], isocratic 50:50 [5 min], 50:50->0:100 [15 min]). The combined product fractions were concentrated and dried under reduced pressure affording 1.23 g (43% yield) of the title compound.
MS (ESIpos): m/z = 268 $(M+H)^+$

Intermediate 9-b

***tert*-Butyl 6-chloro-5-({[6-(trifluoromethyl)pyridin-2-yl]carbonyl}amino)-1H-indazole-1-carboxylate**

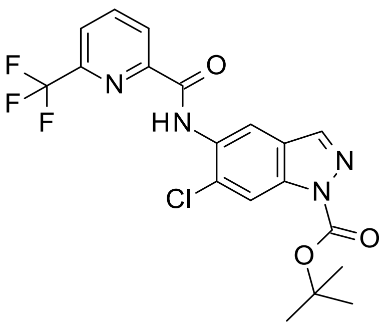

1.14 g (5.97 mmol) of 6-(trifluoromethyl)pyridine-2-carboxylic acid, 704 mg (4.59 mmol) HOBt, 1.76 g (8.19 mmol, 2 eq.) EDC and 1.9 mL (3 eq.) TEA were added to a solution of 1.23 g (4.59 mmol) of *tert*-butyl 5-amino-6-chloro-1H-indazole-1-carboxylate in 20 mL of DMF. After stirring at rt, 0.5 eq. 6-(trifluoromethyl)pyridine-2-carboxylic acid and 0.5 eq. HOBt were added a second time and the mixture was stirred for 3 days. Water was added, the mixture was stirred for 15 min, and the solid was filtered off with suction, washed three times with water, and dried under reduced pressure affording 2.02 g (98% yield) of the title compound.

MS (ESIpos): m/z = 441 $(M+H)^+$

^{1}H NMR (DMSO-d_6, 300 MHz) δ 1.65 (s, 9H), 8.19–8.27 (m, 2H), 8.37–8.53 (m, 3H), 8.75 (s, 1H), 10.59 (s, 1H).

<u>Intermediate 9-c</u>

N-(6-Chloro-1H-indazol-5-yl)-6-(trifluoromethyl)pyridine-2-carboxamide

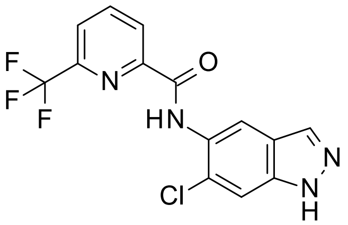

6.7 mL (87.3 mmol, 10 eq.) of TFA were added to 3.85 g (8.73 mmol) of *tert*-butyl 6-chloro-5-({[6-(trifluoromethyl)pyridine-2-yl]carbonyl}amino)-1H-indazole-1-carboxylate in 40 mL of DCM, and the mixture was stirred at rt for 18 h. After the addition of sat. aqueous sodium bicarbonate solution, the resulting solid was filtered with suction, washed with water and diethylether and dried to afford 2.98 g (100% yield) of the title compound.

MS (ESIpos): m/z = 341 $(M+H)^+$

^{1}H NMR (DMSO-d_6, 300 MHz) δ 7.83 (s, 1H), 8.14–8.27 (m, 2H), 8.36–8.49 (m, 2H), 8.60 (s, 1H), 10.50 (br. S., 1H), 13.25 (br. S., 1H).

Intermediate 9-d

***tert*-Butyl [6-chloro-5-({[6-(trifluoromethyl)pyridine-2-yl]carbonyl}amino)-2H-indazol-2-yl]acetate**

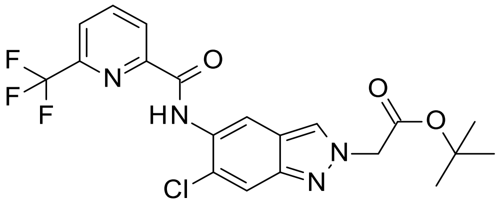

4.48 g (12.2 mmol) of *N*-(6-chloro-1H-indazol-5-yl)-6-(trifluoromethyl)pyridine-2-carboxamide were initially charged in 40 mL of THF. 3.61 mL (24.5 mmol) of *tert*-butyl bromoacetate and 5.19 mL (24.5 mmol) of *N*,*N*-dicyclohexylmethylamine were added. The mixture was stirred at 70 °C for 5.5 h. Another 3.61 mL (24.5 mmol) of *tert*-butyl bromoacetate and 5.19 mL (24.5 mmol) of *N*,*N*-dicyclohexylmethylamine were added and the mixture was stirred at 65 °C for 18 h. Then another 1.81 mL (12.3 mmol) of *tert*-butyl bromoacetate and 2.60 mL (12.3 mmol) of *N*,*N*-dicyclohexylmethylamine were added, and the mixture was stirred at 65 °C for 6 h. The mixture was filtered, water was added to the filtrate, the mixture was extracted three times with ethyl acetate, and the combined organic phases were washed with 1M HCl, sat. sodium bicarbonate solution, and brine and concentrated. Trituration of the crude product with ethyl acetate gave, after drying, 1.45 g (26% yield) of the title compound.
MS (ESIpos): m/z = 455 $(M+H)^+$

Intermediate 9-e

([6-Chloro-5-({[6-(trifluoromethyl)pyridin-2-yl]carbonyl}amino)-2H-indazol-2-yl]acetic acid

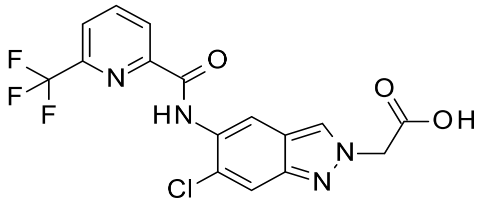

1.45 g (3.19 mmol) of *tert*-butyl [6-chloro-5-({[6-(trifluoromethyl)pyridin-2-yl]carbonyl}amino)-2H-indazol-2-yl]acetate were dissolved in 15 mL of DCM and 2.46 mL (31.9 mmol) of TFA were added at 25 °C. The solution was stirred at 25 °C for 18 h. Water was added, the resulting precipitate was filtered off with suction, washed three times with water and twice

with diethylether, and the solid was dried under reduced pressure. This gave 1.28 g (98% yield) of the title compound.

MS (ESIpos): m/z = 399 $(M+H)^+$

^{1}H NMR (DMSO-d_6, 400 MHz) δ 5.31 (s, 2H), 7.93 (s, 1H), 8.22 (dd, 1H, *J*=1.0, 7.6 Hz), 8.40 (t, 1H, *J*=7.7 Hz), 8.46 (d, 1H, *J*=7.7 Hz), 8.49 (d, 1H, *J*=0.8 Hz), 8.64 (s, 1H), 10.52 (s, 1H), 13.28 (br s, 1H).

Compound 9 (step 9-f)

N-{2-[2-(4-Benzoylpiperazin-1-yl)-2-oxoethyl]-6-chloro-2H-indazol-5-yl}-6-(trifluoromethyl)pyridine-2-carboxamide

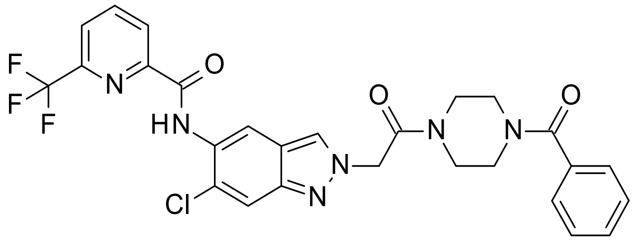

147 mg (0.77 mmol, 1.3 eq.) of phenyl(piperazin-1-yl)methanone, 91 mg (0.59 mmol) of HOBt, 228 mg (1.19 mmol, 2 eq.) of EDC and 0.25 mL of TEA (3.0 eq.) were added to 236 mg (0.59 mmol) if ([6-chloro-5-({[6-(trifluoromethyl)pyridin-2-yl]carbonyl}amino)-2H-indazol-2-yl]acetic acid in 4 mL THF. After stirring at rt overnight, water was added and the resulting solid was filtered with suction, washed with water and diethylether, and dried to afford 321 mg (95% yield) of the title compound.

LC-MS (method A): Rt = 1.22 min; MS (ESIpos): m/z = 571 $[M+H]^+$

^{1}H NMR (DMSO-d_6, 300 MHz) δ 3.5–3.7 (m, 8H), 5.54 (br s, 2H), 7.4–7.5 (m, 5H), 7.91 (s, 1H), 8.23 (dd, 1H, *J*=1.1, 7.5 Hz), 8.4–8.5 (m, 3H), 8.64 (s, 1H), 10.53 (s, 1H).

Synthesis of compound 10

Intermediate 10-a

N-(6-Ethoxy-1H-indazol-5-yl)-6-(trifluoromethyl)pyridine-2-carboxamide

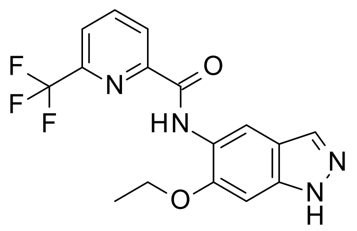

1.00 g (5.64 mmol) of 6-ethoxy-1H-indazole-5-amine and 1.29 g (6.77 mmol) of 6-(trifluoromethyl)pyridine-2-carboxylic acid were reacted with 864 mg (5.64 mmol) HOBt, 2.16 g (11.3 mmol, 2 eq.) EDC, and 2.4 mL (3.0 eq.) TEA in 50 mL of THF at rt overnight. After addition of water, the mixture was extracted three times with ethyl acetate and the combined organic layers were washed with brine, filtered through a water-repellent filter, and concentrated. Purification by column chromatography using the Isolera® flash purification system (eluent hexane/ethyl acetate) gave 1.30 g (64% yield) of the title compound.

MS (ESIpos): m/z = 351 $(M+H)^+$

^{1}H NMR (DMSO-d_6, 500 MHz) δ 1.51 (t, 3H, *J*=6.8 Hz), 4.24 (q, 2H, *J*=6.8 Hz), 7.10 (s, 1H), 8.00 (s, 1H), 8.20 (dd, 1H, *J*=0.8, 7.8 Hz), 8.41 (t, 1H, *J*=7.8 Hz), 8.47 (d, 1H, *J*=7.6 Hz), 8.79 (s, 1H), 10.67 (s, 1H), 12.87 (s, 1H).

<u>**Intermediate 10-b**</u>

Ethyl [6-ethoxy-5-({[6-(trifluoromethyl)pyridin-2-yl]carbonyl}amino)-2H-indazol-2-yl]acetate

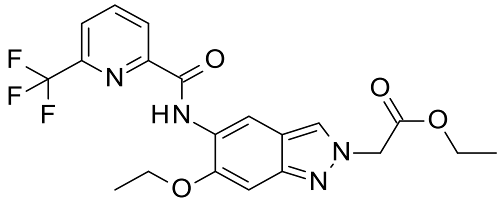

1.30 g (3.71 mmol) of *N*-(6-ethoxy-1H-indazol-5-yl)-6-(trifluoromethyl)pyridine-2-carboxamide, 826 µl (7.42 mmol) of ethyl bromoacetate, and 1.54 mL (7.42 mmol) of *N*,*N*-dicyclohexylmethylamine in 20 mL of THF were stirred at 65 °C for 18 h. 413 µl (3.71 mmol) of ethyl bromoacetate and 770 µl (3.71 mmol) of *N,N*-dicyclohexylmethylamine were added and the mixture was stirred at 65 °C for 6 h. The resulting solid was filtered and washed with ethyl acetate twice. The solid was then transferred to a mixture of water and ethyl acetate and the organic layer was separated. The aqueous layer was extracted with ethyl acetate and the combined organic layers were washed with brine, filtered with a water-repellent filter, and dried, affording 143 mg of the title compound (crude batch). A further 637 mg of the title

compound were obtained after the addition of water to the reaction filtrate; extraction with ethyl acetate; washing the organic phase with 1M HCl, sat. sodium bicarbonate solution, and brine; drying, concentration; and trituration of the residue with ethyl acetate.

MS (ESIpos): m/z = 437 $(M+H)^+$

^{1}H NMR (DMSO-d_6, 400 MHz) δ 1.23 (t, 3H, *J*=7.0 Hz), 1.51 (t, 3H, *J*=6.9 Hz), 4.14–4.27 (m, 4H), 5.31 (s, 2H), 7.10 (s, 1H), 8.18–8.23 (m, 1H), 8.31 (s, 1H), 8.37–8.44 (m, 1H), 8.45–8.49 (m, 1H), 8.73 (s, 1H), 10.74 (s, 1H).

Intermediate 10-c

[6-Ethoxy-5-({[6-(trifluoromethyl)pyridin-2-yl]carbonyl}amino)-2H-indazol-2-yl]acetic acid

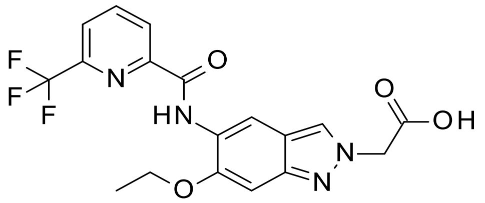

A solution of 745 mg (17.74 mmol) of lithium hydroxide monohydrate dissolved in 5 mL of water was added to 774 mg (1.77 mmol) of ethyl {[6-ethoxy-5-({[6-(trifluoromethyl)pyridin-2-yl]carbonyl}amino)-2H-indazol-2-yl]acetate in 1 mL of ethanol and 25 mL of THF, and the mixture was stirred at 25 °C for 3 days. After the addition of water, the mixture was acidified to pH 4 by adding aqueous citric acid solution (10%). The solid was filtered, washed with water and diethylether, and dried affording 698 mg (94% yield) of the title compound.

MS (ESIpos): m/z = 409 $(M+H)^+$

^{1}H NMR (DMSO-d_6, 300 MHz) δ 1.49 (t, 3H, *J*=7.0 Hz), 4.20 (q, 2H, *J*=7.0 Hz), 5.17 (s, 2H), 7.09 (s, 1H), 8.21 (dd, 1H, *J*=1.1, 7.5 Hz), 8.28 (s, 1H), 8.36–8.48 (m, 2H), 8.71 (s, 1H), 10.73 (s, 1H).

Compound 10 (step 10-d)

N-{2-[2-(4-Benzoylpiperazin-1-yl)-2-oxoethyl]-6-ethoxy-2H-indazol-5-yl}-6-(trifluoromethyl)pyridine-2-carboxamide

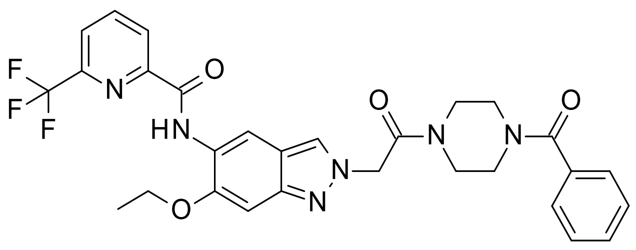

38 mg (0.25 mmol, 1.0 eq.) of HOBt, 94 mg (0.49 mmol, 2.0 eq.) of EDC and 0.10 mL (3.0 eq.) of TEA were added to a mixture of 100 mg (0.25 mmol) of [6-ethoxy-5-({[6-(trifluoromethyl)pyridin-2-yl]carbonyl}amino)-2H-indazol-2-yl]acetic acid and 61 mg (0.32 mmol, 1.3 eq.) of phenyl(piperazin-1-yl)methanone in 4 mL THF. After stirring at rt overnight, water was added and the resulting solid was filtered, washed with water and diethylether and dried, affording 101 mg (0.17 mmol, 71% yield) of the title compound.
LC-MS (method A): Rt = 1.23 min; MS (ESIpos): m/z = 581 $[M+H]^+$

^{1}H NMR (DMSO-d_6, 400 MHz) δ 1.49 (t, 3H, *J*=6.9 Hz), 3.33–3.79 (m, 8H), 4.20 (q, 2H, *J*=6.8 Hz), 5.41 (br. s., 2H), 7.08 (s, 1H), 7.41–7.50 (m, 5H), 8.19–8.24 (m, 2H), 8.37–8.47 (m, 2H), 8.72 (s, 1H), 10.7 (s, 1H).

Synthesis of compound 11

<u>**Intermediate 11-a**</u>

6-Isopropoxy-1H-indazole-5-amine

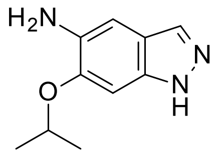

10 g (45.2 mmol) of 6-isopropoxy-5-nitro-1H-indazole (CAS Number 1082041-56-2) were dissolved in 200 mL of ethanol and hydrogenated with 1.20 g (1.13 mmol) of palladium on activated carbon under standard hydrogen pressure at 25 °C for 24 h. The reaction mixture was filtered through Celite®, the filter cake was washed with ethanol, and the filtrate was concentrated. Ethanol was added and after treatment with ultrasound, diethylether was added and the residue was digested further in the ultrasonic bath. The solid was filtered off with suction and washed with a little diethylether and hexane, giving 4.69 g (54%) of product. The filtrate was concentrated and applied to a Biotage SNAP cartridge (100 g; KP-Sil) pre-equilibrated with hexane and chromatography was carried out using the Isolera® flash

purification system (Biotage) (mobile phase: hexane/ethyl acetate; gradient: 90:10->35:65). The combined product fractions were concentrated and the residue was treated with ultrasound in a mixture of hexane and DCM (2:1) in an ultrasonic bath. The solid formed was filtered off. This gave an additional 2.36 g (27% yield) of the title compound.
MS (ESIpos): m/z = 192 $(M+H)^+$
^{1}H NMR (DMSO-d_6, 400 MHz) δ 1.31 (s, 3 H), 1.33 (s, 3 H), 4.43 (s, 2 H), 4.57–4.68 (m, 1 H), 6.81 (s, 1 H), 6.83 (s, 1 H), 7.64 (s, 1 H), 12.34 (br. S., 1 H).

The next steps for the synthesis of compound **11** were performed similarly to the methods described for the preparation of compounds **7** and **8**:

Intermediate 11-b

tert-Butyl (6-isopropoxy-1H-indazol-5-yl)carbamate

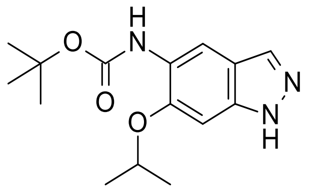

2.2 g (11.6 mmol) of 6-isopropoxy-1H-indazole-5-amine was reacted with 2.52 g (11.6 mmol) of di-*tert*-butyl dicarbonate and 2.21 mL (12.7 mmol) of DIPEA in 18 ml THF at rt for 16 h. After evaporation of the solvents, 11 mL DCM and 100 mL hexane were added. Cooling in a fridge, filtration with suction, washing with hexane, and drying of the solid gave 2.72 g (81% yield) of the title compound.
MS (ESIpos): m/z = 292 $(M+H)^+$

Intermediate 11-c

Ethyl {5-[(*tert*-butoxycarbonyl)amino]-6-isopropoxy-2H-indazol-2-yl}acetate

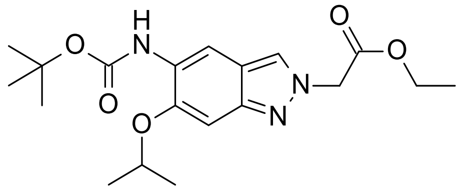

2.72 g (9.3 mmol) of *tert*-butyl (6-isopropoxy-1H-indazol-5-yl)carbamate were reacted with 3.10 mL (28.0 mmol) of ethyl bromoacetate. This gave 1.84 g (52% yield) of the title compound.

MS (ESIpos): m/z = 378 $(M+H)^+$

^{1}H NMR (DMSO-d_6, 600 MHz) δ 1.21 (t, 3H, *J*=7.2 Hz), 1.34 (d, 6H, *J*=6.0 Hz), 1.48 (s, 9H), 4.16 (q, 2H, *J*=7.1 Hz), 4.71 (spt, 1H, *J*=6.0 Hz), 5.27 (s, 2H), 6.98 (s, 1H), 7.63 (s, 1H), 7.97 (s, 1H), 8.17 (s, 1H).

Intermediate 11-d

Ethyl (5-amino-6-isopropoxy-2H-indazol-2-yl)acetate

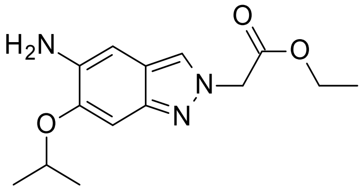

1.8 g (4.84 mmol) of ethyl {5-[(*tert*-butoxycarbonyl)amino]-6-isopropoxy-2H-indazol-2-yl}acetate were reacted with 2.8 mL (36.3 mmol) of TFA. This gave 1.3 g (100% yield) of the title compound.

MS (ESIpos): m/z = 278 $(M+H)^+$

^{1}H NMR (DMSO-d_6, 300 MHz) δ 1.20 (t, 3H, *J*=7.1 Hz), 1.32 (d, 6H, *J*=6.1 Hz), 4.15 (q, 2H, *J*=7.1 Hz), 4.59 (s, 1H), 4.60–4.69 (m, 1H), 5.16 (s, 2H), 6.64 (s, 1H), 6.80 (s, 1H), 7.83 (s, 1H).

Intermediate 11-e

Ethyl [6-isopropoxy-5-({[6-(trifluoromethyl)59yridine-2-yl]carbonyl}amino)-2H-indazol-2-yl]acetate

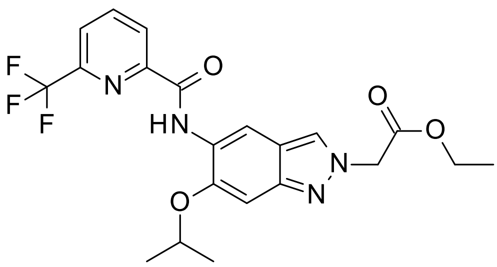

300 mg (1.08 mmol) of ethyl (5-amino-6-isopropoxy-2H-indazol-2-yl)acetate were reacted with 227 mg (1.19 mmol) of 6-(trifluoromethyl)pyridine-2-carboxylic acid. This resulted in 487 mg (100% yield) of the title compound.

MS (ESIpos): m/z = 451 $(M+H)^+$

^{1}H NMR (DMSO-d_6, 300 MHz) δ 1.21 (t, 3H, *J*=7.2 Hz), 1.40 (d, 6H, *J*=5.8 Hz), 4.17 (q, 2H, *J*=7.1 Hz), 4.79–4.92 (m, 1H), 5.32 (s, 2H), 7.18 (s, 1H), 8.22 (d, 1H), 8.33 (s, 1H), 8.37–8.50 (m, 2H), 8.75 (s, 1H), 10.75 (s, 1H).

<u>Intermediate 11-f</u>

[6-Isopropoxy-5-({[6-(trifluoromethyl)pyridine-2-yl]carbonyl}amino)-2H-indazol-2-yl]acetic acid

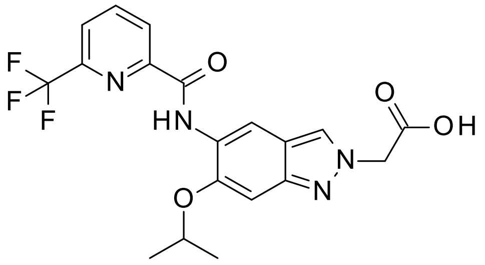

490 mg (1.1 mmol) of ethyl [6-isopropoxy-5-({[6-(trifluoromethyl)pyridine-2-yl]carbonyl}amino)-2H-indazol-2-yl]acetate were reacted with 260 mg (11 mmol) of lithium hydroxide. This gave 367 mg (80% yield) of the title compound.

MS (ESIpos): m/z = 423 $(M+H)^+$

^{1}H NMR (DMSO-d_6, 300 MHz) δ 1.40 (d, 6H, *J*=6.0 Hz), 1.7–1.8 (m, 2H), 3.5–3.6 (m, 2H), 4.85 (td, 1H, *J*=6.0, 12.0 Hz), 5.20 (s, 2H), 7.16 (s, 1H), 8.21 (dd, 1H, *J*=1.1, 7.5 Hz), 8.29 (s, 1H), 8.4–8.5 (m, 2H), 8.74 (s, 1H), 10.74 (s, 1H), 13.20 (br s, 1H)

<u>Compound 11 (step 11-g)</u>

N-{2-[2-(4-Benzoylpiperazin-1-yl)-2-oxoethyl]-6-isopropoxy-2H-indazol-5-yl}-6-methylpyridine-2-carboxamide

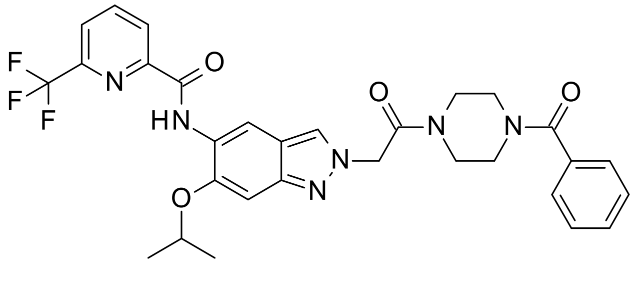

87 mg (0.17 mmol) of [6-Isopropoxy-5-({[6-(trifluoromethyl)pyridine-2-yl]carbonyl}amino)-2H-indazol-2-yl]acetic acid were reacted with 47.3 mg (1.5 eq.) of phenyl(piperazin-1-yl)methanone. This gave 68 mg (0.11 mmol, 69% yield) of the title compound.

LC-MS (method A): Rt = 1.26 min; MS (ESIpos): m/z = 595 $[M+H]^+$

^{1}H NMR (DMSO-d_6, 300 MHz) δ 1.40 (d, 7H, *J*=6.0 Hz), 3.37 (br s, 1H), 3.55 (br s, 3H), 3.64 (br s, 3H), 4.85 (td, 1H, *J*=6.0, 11.9 Hz), 5.41 (br s, 2H), 7.14 (s, 1H), 7.4–7.5 (m, 5H), 8.2–8.2 (m, 2H), 8.4–8.5 (m, 2H), 8.74 (s, 1H), 10.74 (s, 1H)

Synthesis of compound 12

<u>Compound 12 (step 12-a)</u>

N-{6-Fluoro-2-[2-(4-methylpiperazin-1-yl)-2-oxoethyl]-2H-indazol-5-yl}-6-(trifluoromethyl)pyridine-2-carboxamide

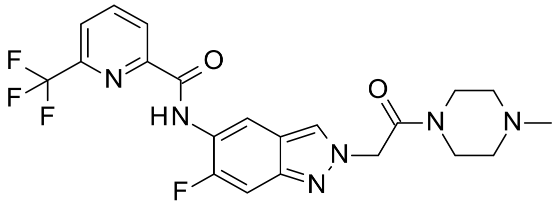

81 mg (0.42 mmol) EDC and 32 mg (0.21 mmol) HOBt were added to 80 mg (0.21 mmol) [6-fluoro-5-({[6-(trifluoromethyl)pyridin-2-yl]carbonyl}amino)-2H-indazol-2-yl]acetic acid (intermediate 8-e) in 3 mL THF and 0.33 mL DMF and the mixture was stirred for 30 min at rt. Then, 36 µL (0.31 mmol) 1-methylpiperazine were added and the resulting mixture was stirred for 30 minutes before being poured into 50 mL of water and, after stirring for a further 5 min, 10 mL ethyl acetate were added and the phases were separated. The aqueous phase was extracted with ethyl acetate twice, and the combined organic layers were washed with water, dried with magnesium sulfate, and evaporated. Preparative HPLC purification afforded 42 mg (43% yield) of the title compound.

LC-MS (method A): Rt = 0.93 min; MS (ESIpos): m/z = 465 $[M+H]^+$

^{1}H NMR (DMSO-d_6, 300 MHz) δ 2.21 (s, 3H), 2.29 (m, 2H), 2.38 (m, 2H), 3.47 (m, 2H), 3.55 (m, 2H), 5.47 (s, 2H), 7.52 (d, 1H), 8.22 (m, 1H), 8.34–8.48 (m, 4H), 10.24 (m, 1H).

Synthesis of compound 13

<u>**Intermediate 13-a**</u>

***tert*-Butyl 6-bromo-5-[(*tert*-butoxycarbonyl)amino]-1H-indazole-1-carboxylate and *tert*-butyl 6-bromo-5-[(*tert*-butoxycarbonyl)amino]-2H-indazol-2-carboxylate**

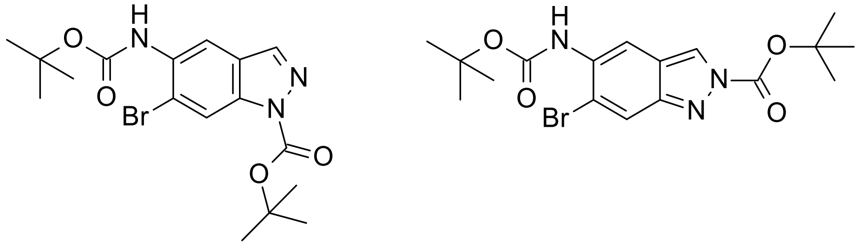

27.5 g (126.1 mmol) of di-*tert*-butyl dicarbonate were dissolved in 53.5 mL of THF and cooled to 0 °C. After the addition of 5.35 g (25.2 mmol) of 6-bromo-1H-indazole-5-amine (CAS Number 1360928-41-1) at 0 °C, the mixture was then stirred at 80 °C for 24 h. The reaction mixture was concentrated, DCM was added, and the reaction mixture was washed with 0.5 M HCl and brine; dried over sodium sulphate; and, during concentration, adsorbed onto ISOLUTE® HM-N (Biotage). The Isolute was then applied to a Biotage SNAP cartridge (340 g; KP-Sil) pre-equilibrated with hexane and chromatography was carried out using the Isolera® flash purification system (Biotage) (mobile phase: hexane/ethyl acetate; gradient: isocratic 80:20). This afforded 7.07 g (68% yield) of the regioisomeric product mixture (ratio: 1-isomer/2-isomer: 85%/15%).
MS (ESIneg): m/z = 410 (M(^{79}Br)-H)$^+$

<u>**Intermediate 13-b**</u>

***tert*-Butyl (6-bromo-1H-indazol-5-yl)carbamate**

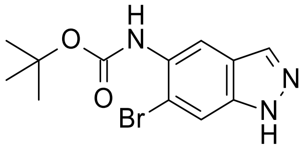

7.05 g (17.1 mmol) of a mixture of *tert*-butyl 6-bromo-5-[(*tert*-butoxycarbonyl)amino]-1H-indazole-1-carboxylate and *tert*-butyl 6-bromo-5-[(*tert*-butoxycarbonyl)amino]-2H-indazole-2-carboxylate (from the previous step) were dissolved in 141 mL of DMF and 2.17 g (20.5 mmol) of sodium carbonate in 71 mL of water was added. The reaction mixture was heated at 85 °C for 24 h. Dichloromethane was added and the reaction mixture was washed with 0.5

M HCl and brine, dried over sodium sulphate, and concentrated. The product was dried under reduced pressure. This gave 5.35 g (98% yield) of the title compound.
MS (ESIneg): m/z = 310 (M(^{79}Br)-H)$^+$
^{1}H NMR (CHLOROFORM-d, 400 MHz) δ 1.57 (s, 9 H), 7.01 (br. s., 1 H), 7.83 (s, 1 H), 8.07 (s, 1 H), 8.50 (s, 1 H).

Intermediate 13-c

Ethyl {6-bromo-5-[(*tert*-butoxycarbonyl)amino]-2H-indazol-2-yl}acetate

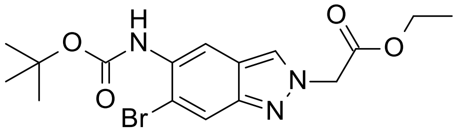

Similarly to the synthesis of intermediate 7-b, 4.85 g (15.5 mmol) of *tert*-butyl (6-bromo-1H-indazol-5-yl)carbamate, 6.89 mL (62.1 mmol) of ethyl bromoacetate, and 13.3 mL (62.1 mmol) of *N*,*N*-dicyclohexylmethylamine in 50 mL of THF were stirred at 70 °C for 24 h. Work-up and purification by column chromatography using the Isolera® flash purification system (Biotage) (mobile phase: hexane/DCM/ethyl acetate) gave 2.01 g (32% yield) of the title compound.
MS (ESIpos): m/z = 398 (M(^{79}Br)+H)$^+$
^{1}H NMR (DMSO-d$_6$, 400 MHz) δ 1.21 (t, 3H, *J*=7.2 Hz), 1.45 (s, 9H), 4.17 (q, 2H, *J*=7.0 Hz), 5.40 (s, 2H), 7.78 (s, 1H), 7.96 (s, 1H), 8.41 (d, 1H), 8.54 (s, 1H).

Intermediate 13-d

{6-Bromo-5-[(*tert*-butoxycarbonyl)amino]-2H-indazol-2-yl}acetic acid

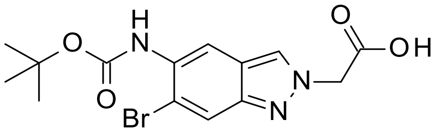

Similar to intermediate 7-c, 1.00 g (2.5 mmol) of ethyl {6-bromo-5-[(*tert*-butoxycarbonyl)amino]-2H-indazol-2-yl}acetate was dissolved in 50 mL of THF, a solution of 301 mg (12.6 mmol) of lithium hydroxide monohydrate in 4.5 mL of water was then added and the mixture was stirred at 25 °C for 24 h. Work-up gave 844 mg (82% yield) of the title compound.
MS (ESIpos): m/z = 370 (M(^{79}Br)+H)$^+$

^1H NMR (DMSO-d_6, 300 MHz) δ 1.45 (s, 9 H), 3.35 (s br, 1 H), 5.28 (s, 2 H), 7.76 (s, 1 H), 7.95 (s, 1 H), 8.38 (s, 1 H), 8.52 (s, 1 H).

Intermediate 13-e

tert-Butyl {6-bromo-2-[2-(4-methylpiperazin-1-yl)-2-oxoethyl]-2H-indazol-5-yl}carbamate

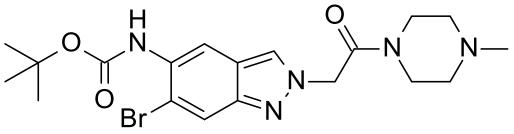

Similar to the synthesis of intermediate 7-d, 800 mg (1.97 mmol) of {6-bromo-5-[(*tert*-butoxycarbonyl)amino]-2H-indazol-2-yl}acetic acid was reacted with 246 µL (2.17 mmol) of 1-methylpiperazine. This gave 824 mg (93% yield) of the title compound.
MS (ESIpos): m/z = 452 (M(^{79}Br)+H)$^+$
^1H NMR (DMSO-d_6, 300 MHz) δ 1.45 (s, 9 H), 2.20 (s, 3 H), 2.25–2.34 (m, 2 H), 2.34–2.40 (m, 2 H), 3.43–3.49 (m, 2 H), 3.50–3.55 (m, 2 H), 5.47 (s, 2 H), 7.75 (s, 1 H), 7.93 (s, 1 H), 8.31 (s, 1 H), 8.54 (s, 1 H).

Intermediate 13-f

2-(5-Amino-6-bromo-2H-indazol-2-yl)-1-(4-methylpiperazin-1-yl)ethanone

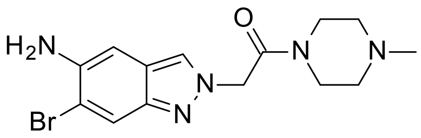

Similar to the synthesis of intermediate 7-e, 293 mg (0.65 mmol) of *tert*-butyl {6-bromo-2-[2-(4-methylpiperazin-1-yl)-2-oxoethyl]-2H-indazol-5-yl}carbamate was reacted with 499 µL (6.48 mmol) of TFA in 3 mL of DCM. Work-up gave 210 mg (92% yield) of the title compound.
MS (ESIpos): m/z = 352 (M(^{79}Br)+H)$^+$
^1H NMR (DMSO-d_6, 400 MHz) δ 2.18 (s, 3H), 2.2–2.4 (m, 4H), 3.4–3.5 (m, 4H), 4.90 (s, 2H), 5.33 (s, 2H), 6.91 (s, 1H), 7.76 (s, 1H), 7.94 (d, 1H, *J*=0.8 Hz).

Intermediate 13-g

{6-bromo-2-[2-(4-methylpiperazin-1-yl)-2-oxoethyl]-2H-indazol-5-yl}-6-(trifluoromethyl)pyridine-2-carboxamide

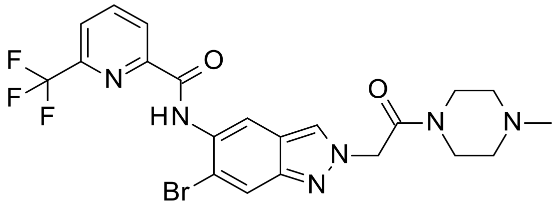

110 mg (0.31 mmol) 2-(5-Amino-6-bromo-2H-indazol-2-yl)-1-(4-methylpiperazin-1-yl)ethenone were dissolved in 2 mL THF and 2 mL DMF. Afterwards, 120 mg (0.62 mmol, 2.0 eq.) EDC, 47.8 mg (0.31 mmol, 1.0 eq.) HOBt, 0.31 mL TEA (3.0 eq.), and 71.6 mg (0.37 mmol, 1.2 eq.) 6-(trifluoromethyl)pyridine-2-carboxylic acid were added and the mixture was stirred at rt resulting in a suspension that was poured into water. The solid was filtered out with suction, washed with diethylether, and dried to afford 125 mg (76% yield) of a white solid.
MS (ESIpos): m/z = 525 (M(79Br)+H)$^+$
^{1}H NMR (DMSO-d$_6$, 300 MHz) δ 2.21 (s, 3 H), 2.29 (br. s., 2 H), 2.38 (br. s., 2 H), 3.47 (br. s., 2 H), 3.54 (br. s., 2 H), 5.50 (s, 2 H), 8.09 (s, 1 H), 8.24 (d, 1 H), 8.35–8.50 (m, 3 H), 8.64 (s, 1 H), 10.54 (s, 1 H).

<u>**Compound 13 (step 13-h)**</u>

N-{6-Cyano-2-[2-(4-methylpiperazin-1-yl)-2-oxoethyl]-2H-indazol-5-yl}-6-(trifluoromethyl)pyridine-2-carboxamide

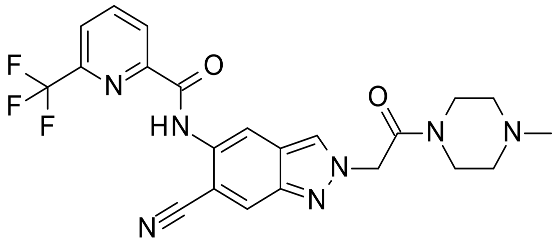

50 mg (0.10 mmol) of *N*{6-bromo-2-[2-(4-methylpiperazin-1-yl)-2-oxoethyl]-2H-indazol-5-yl}-6-(trifluoromethyl)pyridine-2-carboxamide, 5 mg (0.005 mmol) of tetrakis(triphenylphosphine)palladium(0), and 12 mg (0.10 mmol) of zinc cyanide were initially charged in a microwave vessel and suspended in 1 mL of DMF. The reaction mixture was stirred in the microwave at 150 °C for 15 minutes. Since the reaction was still incomplete, another 5 mg (0.005 mmol) of tetrakis(triphenylphosphine)palladium(0) and 5.5 mg (0.05 mmol) of zinc cyanide were added and the mixture was stirred in the microwave at 150 °C for a further 30 minutes. The reaction mixture was diluted with ethyl acetate and washed with water and brine. The solution was then filtered through a hydrophobic filter and concentrated.

The crude product was dissolved in 2.5 mL of DMF and purified by preparative HPLC. The product fraction was lyophilized. This gave 25 mg (56% yield) of the title compound.
LC-MS (method C): Rt = 0.81 min; MS (ESIpos): m/z = 472 [M+H]+
^{1}H NMR (DMSO-d_6, 400 MHz) δ 2.22 (s, 3 H), 2.27–2.33 (m, 2 H), 2.36–2.42 (m, 2 H), 3.44–3.50 (m, 2 H), 3.52–3.58 (m, 2 H), 5.59 (s, 2 H), 8.21–8.26 (m, 2 H), 8.37–8.43 (m, 2 H), 8.43–8.47 (m, 1 H), 8.51 (d, 1 H), 10.66 (s, 1 H).

Synthesis of compound 14

<u>Intermediate 14-a</u>

N-(6-Methoxy-1H-indazol-5-yl)-6-(trifluoromethyl)pyridine-2-carboxamide

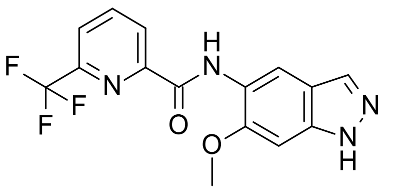

6-methoxy-1H-indazol-5-amine (25.0 g, 153 mmol, CAS Number 749223-61-8) and 6-(trifluoromethyl)pyridine-2-carboxylic acid (32.2 g, 169 mmol) were dissolved in THF (600 ml) and 1-(3-dimethylaminopropyl)-3-ethylcarbodiimide hydrochloride (44.1 g, 230 mmol), TEA (64 ml, 460 mmol), and HOBt (23.5 g, 153 mmol) were added. The reaction mixture was stirred for 22 h at 25 °C. Afterwards, the reaction mixture was diluted with water and extracted three times with ethyl acetate. The combined organic layers were washed with brine, filtered over a water-repellent filter, and concentrated *in vacuo.* The formed suspension was filtered, washed with diethylether, and dried. The residual filtrate was concentrated *in vacuo* and the residue was triturated with diethyl ether. This suspension was filtered, washed with diethylether and also dried. The dried solids were combined to afford 37.3 g (71% yield) of the title compound.
MS (ESIpos): m/z = 337 $[M+H]^+$
^{1}H NMR (DMSO-d_6, 400 MHz) δ 4.00 (s, 3H), 7.12 (s, 1H), 8.01 (s, 1H), 8.20 (dd, *J*=7.71, 0.88 Hz, 1H), 8.36–8.42 (m, 1H), 8.44–8.50 (m, 1H), 8.73 (s, 1H), 10.41 (s, 1H), 12.90 (br s, 1H).

<u>Intermediate 14-b</u>

tert-Butyl [6-methoxy-5-({[6-(trifluoromethyl)pyridin-2-yl]carbonyl}amino)-2H-indazol-2-yl]acetate

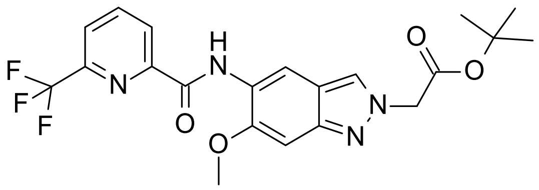

6.50 g (19.3 mmol) of *N*-(6-methoxy-1H-indazol-5-yl)-6-(trifluoromethyl)pyridine-2-carboxamide were dissolved in 100 mL THF and 8.56 mL (58.0 mmol) of *tert*-butyl bromoacetate and 8.20 mL (38.7 mmol) of *N*,*N*-dicyclohexylmethylamine were added at 25 °C. The solution was stirred at 70 °C for 18 h. 0.5 eq. of *tert*-butyl bromoacetate and 0.5 eq. *N*,*N*-dicyclohexylmethylamine were added, and the mixture was stirred at 70 °C for a further 7 h. The solid in the reaction mixture was filtered off and washed with ethyl acetate, water, and diethethylether and dried to afford 4.4 g (51% yield) of the title compound. A second batch crystallized from the filtrate produced 616 mg (7% yield) of the title compound.

MS (ESIpos): m/z = 451 $(M+H)^+$

^{1}H NMR (DMSO-d_6, 500 MHz) δ 1.45 (s, 9H), 3.99 (s, 3H), 5.20 (s, 2H), 7.14 (s, 1H), 8.21 (dd, 1H, *J*=1.0, 7.6 Hz), 8.30 (s, 1H), 8.41 (t, 1H, *J*=7.8 Hz), 8.47 (d, 1H, *J*=7.6 Hz), 8.71 (s, 1H), 10.51 (s, 1H)

<u>**Intermediate 14-c**</u>

[6-Methoxy-5-({[6-(trifluoromethyl)pyridine-2-yl]carbonyl}amino)-2H-indazol-2-yl]acetic acid

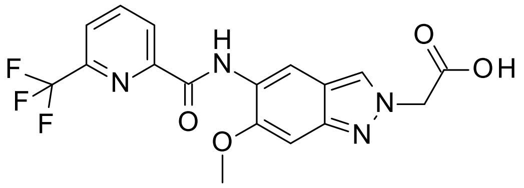

5.0 g (11.1 mmol) of *tert*-butyl [6-methoxy-5-({[6-(trifluoromethyl)pyridine-2-yl]carbonyl}amino)-2H-indazol-2-yl]acetate was stirred in 8.5 mL (48.8 mmol) trifluoracetic acid and 50 mL DCM for 3 days at 25 °C. Water was added and the resulting solid was filtered off, washed with water and diethylether, and dried to afford 5.1 g (batch contained water) of the title compound.

MS (ESIpos): m/z = 395 $(M+H)^+$

^1H NMR (DMSO-d_6, 400 MHz) δ 3.98 (s, within water signal), 5.20 (s, 2H), 7.13 (s, 1H), 8.20 (dd, 1H, *J*=1.0, 7.6 Hz), 8.30 (s, 1H), 8.39 (t, 1H, *J*=7.8 Hz), 8.46 (d, 1H, *J*=7.3 Hz), 8.70 (s, 1H), 10.50 (s, 1H)

__Intermediate 14-d__

N-{6-Methoxy-2-[2-(4-methylpiperazin-1-yl)-2-oxoethyl]-2H-indazol-5-yl}-6-(trifluoromethyl)pyridine-2-carboxamide

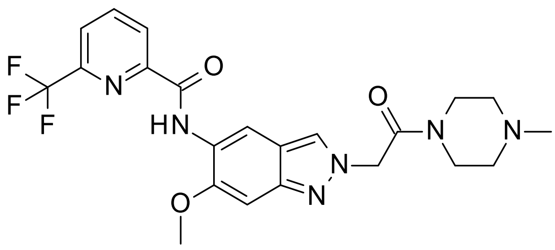

1.94 g (19.4 mmol) 1-methylpiperazine, 1.98 g HOBt, 4.96 g EDC, and 9.0 mL (65 mmol) TEA were added to 5.10 g [6-methoxy-5-({[6-(trifluoromethyl)pyridin-2-yl]carbonyl}amino)-2H-indazol-2-yl]acetic acid (batch yielded by the previous step) in 150 mL THF. The resulting mixture was stirred at rt for 24 h and at 50 °C for 2 h. The mixture was diluted with water, most of the solvents were evaporated, and the resulting solid was filtered off, washed with water and diethylether and dried *in vacuo* to afford 5.0 g (10.3 mmol) of the title compound.
LC-MS (method A): Rt = 0.90 min; MS (ESIpos): m/z = 477 $[M+H]^+$
^1H NMR (DMSO-d_6, 400 MHz) δ 2.20 (s, 3H), 2.28 (br t, 2H, *J*=4.8 Hz), 2.36 (br t, 2H, *J*=4.5 Hz), 3.4–3.5 (m, 2H), 3.5–3.6 (m, 2H), 3.98 (s, 3H), 5.37 (s, 2H), 7.10 (s, 1H), 8.20 (d, 1H, *J*=7.6 Hz), 8.22 (s, 1H), 8.39 (t, 1H, *J*=7.8 Hz), 8.45 (d, 1H, *J*=7.6 Hz), 8.69 (s, 1H), 10.50 (s, 1H)

Synthesis of compound 15

__Intermediate 15-a__

5-Nitro-6-(trifluoromethoxy)-1H-indazole

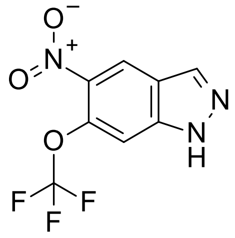

25.4 g (100 mmol) of 2-fluoro-5-nitro-4-(trifluoromethoxy)benzaldehyde was initially charged in 200 mL of absolute ethanol, and 25 mL (513.6 mmol) of hydrazine hydrate were added. The colour of the solution darkened. The reaction mixture was heated under reflux for 2 h. The reaction mixture was then added to 1.4 L of water and stirred vigorously for 10 minutes. The resulting precipitate was filtered off with suction and washed with 40 mL of water three times. The resulting solid was then dried. This gave 19.4 g (78% yield) of the title compound.
MS (ESIpos): m/z = 248 $(M+H)^+$
^{1}H NMR (DMSO-d_6, 400 MHz) δ 7.86 (s, 1 H), 8.46 (s, 1 H), 8.82 (s, 1 H), 13.87 (br. s., 1 H).

Intermediate 15-b

6-(Trifluoromethoxy)-1H-indazole-5-amine

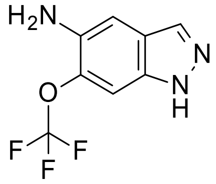

10.0 g (40.5 mmol) of 5-nitro-6-(trifluoromethoxy)-1H-indazole were dissolved in 400 mL of methanol. The solution was then degassed and flushed with nitrogen twice. 2.48 g (2.0 mmol) of palladium on activated carbon were added, after which the flask was evacuated and flushed with hydrogen. The reaction mixture was hydrogenated under standard hydrogen pressure at rt for 5 h. The reaction mixture was filtered through a polytetrafluoroethylene filter with Celite® and concentrated. This gave 7.2 g (74% yield) of the title compound.
MS (ESIpos): m/z = 218 $(M+H)^+$
^{1}H NMR (DMSO-d_6, 400 MHz) δ 4.91 (s, 2 H), 7.04 (s, 1 H), 7.32 (s, 1 H), 7.83 (s, 1 H), 12.72 (br. s., 1 H).

Intermediate 15-c

***tert*-Butyl [6-(trifluoromethoxy)-1H-indazol-5-yl]carbamate**

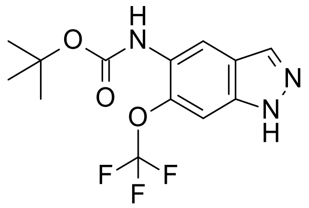

5.0 g (23.0 mmol) of 6-(trifluoromethoxy)-1H-indazole-5-amine were suspended in 100 mL of THF, 4.81 mL (27.6 mmol) of DIPEA were added and the mixture was cooled to 0 °C. After the addition of 5.52 g (25.3 mmol) of di-*tert*-butyl dicarbonate at 0 °C, the mixture was stirred at 25 °C for 18 h. A further 3.52 g (16.1 mmol) of di-*tert*-butyl dicarbonate was added, and the mixture was stirred at 25 °C for a further 24 h. The reaction mixture was then heated at reflux for a further 24 h, after which it was concentrated, taken up in ethyl acetate, and washed with 0.5 M HCl, sat. sodium bicarbonate solution, and brine. The combined organic phases were dried over sodium sulphate and the solution was concentrated after filtration. Chromatography was carried out using the Isolera® flash purification system (Biotage) (mobile phase: hexane/ethyl acetate). The combined product fractions were concentrated and the brownish solid was dried under reduced pressure. This gave 3.48 g (48% yield) of the title compound.

MS (ESIpos): m/z = 318 $(M+H)^+$

^{1}H NMR (DMSO-d_6, 300 MHz) δ =1.44 (s, 9 H), 7.51 (s, 1 H), 7.83 (s, 1 H), 8.11 (s, 1 H), 8.80 (s, 1 H).

Intermediate 15-d

Ethyl {5-[(*tert*-butoxycarbonyl)amino]-6-(trifluoromethoxy)-2H-indazol-2-yl}acetate

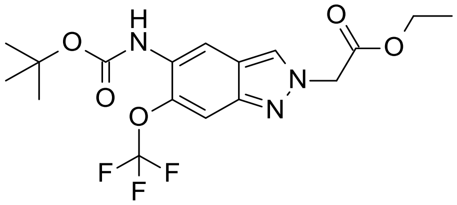

3.17 g (10.0 mmol) of *tert*-butyl [6-(trifluoromethoxy)-1H-indazol-5-yl]carbamate, 5.54 mL (50 mmol) of ethyl bromoacetate, and 10.7 mL (50 mmol) of *N*,*N*-dicyclohexylmethylamine in 20 mL of THF were heated at 70 °C for 24 h. Work-up and purification by column chromatography using the Isolera® flash purification system (Biotage) (mobile phase: hexane/DCM/ethyl acetate) gave 535 mg (13% yield) of the title product.

MS (ESIpos): m/z = 404 $(M+H)^+$

Intermediate 15-e

{5-[(*tert*-Butoxycarbonyl)amino]-6-(trifluoromethoxy)-2H-indazol-2-yl}acetic acid

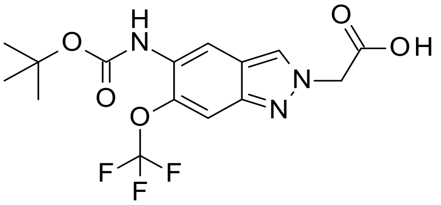

530 mg (1.31 mmol) of ethyl {5-[(*tert*-butoxycarbonyl)amino]-6-(trifluoromethoxy)-2H-indazol-2-yl}acetate were suspended in 20 mL of THF. A solution of 157 mg (6.57 mmol) of lithium hydroxide monohydrate in 2.4 mL of water was then added and the mixture was stirred at 25 °C for 24 h and partly concentrated. Then, aqueous hydrogen chloride solution (1 M) was added and the resulting solid was filtered with suction and washed with cold water to afford 437 mg (81% yield) of the title compound.
MS (ESIpos): m/z = 376 $(M+H)^+$
^{1}H NMR (DMSO-d_6, 400 MHz) δ 1.43 (s, 9 H), 5.28 (s, 2 H), 7.56 (s, 1 H), 7.80 (s, 1 H), 8.40 (d, 1 H, *J*=1.0 Hz), 8.73 (s, 1 H).

Intermediate 15-f

***tert*-Butyl {2-[2-(4-methylpiperazin-1-yl)-2-oxoethyl]-6-(trifluoromethoxy)-2H-indazol-5-yl}carbamate**

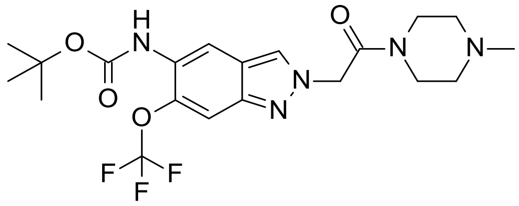

350 mg (0.85 mmol) of *tert*-butyl {2-[2-(4-methylpiperazin-1-yl)-2-oxoethyl]-6-(trifluoromethoxy)-2H-indazol-5-yl}carbamate, 130 mg (0.85 mmol) of HOBt, and 325 mg (1.70 mmol) of EDC in 3.5 mL of DMF and 473 µL (3.40 mmol) of TEA were stirred at 25 °C for 30 min. 103 µL (0.93 mmol) of 1-methylpiperazine were then added and the mixture was stirred at 25 °C for 24 h. The mixture was poured into 50 mL of water, filtered off with suction, washed with water and dried. This gave 305 mg (78% yield) of the title compound.
MS (ESIpos): m/z = 376 $(M+H)^+$

^{1}H NMR (DMSO-d_6, 400 MHz) δ 1.44 (s, 9 H), 2.23 (s, 3 H), 2.28–2.38 (m, 2 H), 2.41 (br. s., 2 H), 3.47 (br. s., 2 H), 3.55 (br. s., 2 H), 5.49 (s, 2 H), 7.54 (s, 1 H), 7.80 (s, 1 H), 8.34 (d, 1 H, *J*=1.0 Hz), 8.73 (s, 1 H), 9.93 (br. s., 1 H).

Intermediate 15-g

2-[5-Amino-6-(trifluoromethoxy)-2H-indazol-2-yl]-1-(4-methylpiperazin-1-yl)ethanone

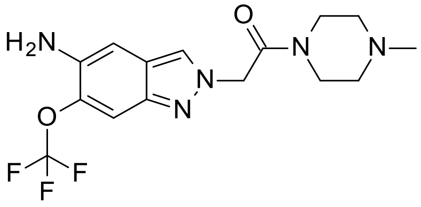

484 mg (1.06 mmol) of *tert*-butyl {2-[2-(4-methylpiperazin-1-yl)-2-oxoethyl]-6-(trifluoromethoxy)-2H-indazol-5-yl}carbamate were reacted with 815 µL of TFA in 5 mL of DCM overnight at rt. The mixture was then heated to 50 °C for 2 h. The mixture was washed with sat. sodium bicarbonate solution, brine, filtered with a water-repellent filter, and concentrated to afford 320 mg (85% yield) of the title compound.
MS (ESIpos): m/z = 357 $(M+H)^+$
^{1}H NMR (DMSO-d_6, 400 MHz) δ 2.18 (s, 3H), 2.2–2.4 (m, 4H), 3.4–3.5 (m, 4H), 4.93 (s, 2 H), 5.34 (s, 2 H), 6.87 (s, 1 H), 7.38 (s, 1 H), 7.97 (s, 1 H)

Compound 15 (step 15-h)

N-{2-[2-(4-Methylpiperazin-1-yl)-2-oxoethyl]-6-(trifluoromethoxy)-2H-indazol-5-yl}-6-(trifluoromethyl)pyridine-2-carboxamide

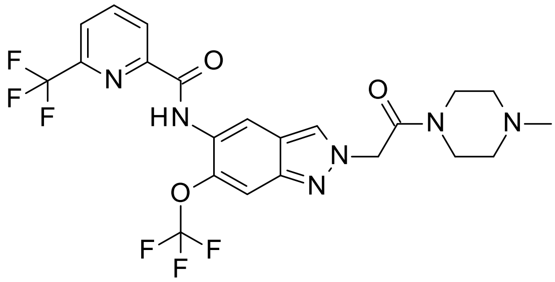

15 mg (1.0 eq.) HOBt, 37.6 mg (2.0 eq.) EDC, 41 µL (3.0 eq.) TEA and 22.5 mg (1.2 eq.) 6-(trifluoromethyl)pyridine-2-carboxylic acid were added to 35.0 mg (100 µmol) 2-[5-amino-6-(trifluoromethoxy)-2H-indazol-2-yl]-1-(4-methylpiperazin-1-yl)ethanone in 2 mL DMF. The mixture was stirred at rt overnight. Purification by HPLC afforded 25 mg (47% yield) of the title compound.

LC-MS (method C): Rt = 0.95 min; MS (ESIpos): m/z = 531 [M+H]$^+$

^{1}H NMR (DMSO-d_6, 400 MHz) δ 2.22 (s, 3H), 2.27–2.36 (m, 2H), 2.37–2.44 (m, 2H), 3.44–3.52 (m, 2H), 3.52–3.60 (m, 2H), 5.52 (s, 2H), 7.75 (s, 1H), 8.23 (dd, 1H, *J*=1.1, 7.7 Hz), 8.38–8.50 (m, 3H), 8.71 (s, 1H), 10.40 (s, 1H)

Synthesis of compound 16

Compound 16 (step 16-a)

N-{6-Ethoxy-2-[2-(4-methylpiperazin-1-yl)-2-oxoethyl]-2H-indazol-5-yl}-6-(trifluoromethyl)pyridine-2-carboxamide

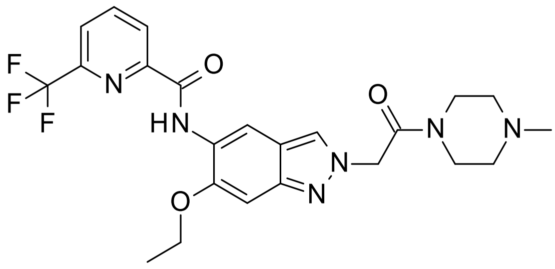

38 mg (1.0 eq.) HOBt, 94 mg (2.0 eq.) EDC and 0.10 mL (3.0 eq.) TEA were added to a mixture of 100 mg (0.25 mmol) [6-ethoxy-5-({[6-(trifluoromethyl)pyridin-2-yl]carbonyl}amino)-2H-indazol-2-yl]acetic acid (Intermediate 10-c) and 1-methylpiperazine (32 mg, 1.3 eq.) in 3 mL THF. After stirring at rt overnight, water and ethyl acetate were added. Evaporation, stirring in a mixture of DMSO and DMF, filtration, washing of the filter residue with diethylether, and drying *in vacuo* afforded 62 mg (51% yield) of the title compound.
LC-MS (method A): Rt = 0.92 min; MS (ESIpos): m/z = 491 [M+H]$^+$

^{1}H NMR (DMSO-d_6, 400 MHz) δ 1.49 (t, 3H, *J*=6.9 Hz), 2.20 (s, 3H), 2.3-2.4 (m, 4H), 3.4-3.5 (m, 2H), 3.54 (br s, 2H), 4.20 (q, 2H, *J*=7.0 Hz), 5.37 (s, 2H), 7.07 (s, 1H), 8.20 (d, 1H, *J*=7.9 Hz), 8.21 (s, 1H), 8.40 (t, 1H, *J*=7.8 Hz), 8.45 (d, 1H, *J*=7.6 Hz), 8.71 (s, 1H), 10.73 (s, 1H).

Synthesis of compound 17

Compound 17 (step 17-a)

N-{6-Isopropoxy-2-[2-(4-methylpiperazin-1-yl)-2-oxoethyl]-2H-indazol-5-yl}-6-(trifluoromethyl)pyridine-2-carboxamide

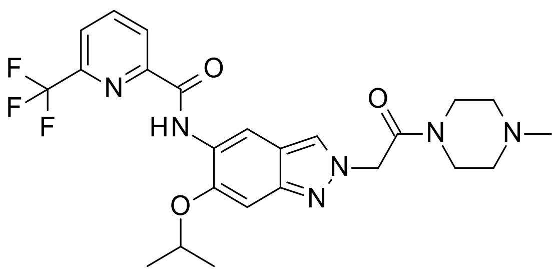

25 mg (1.0 eq.) HOBt and 64 mg (2.0 eq.) EDC were added to a mixture of 70 mg (0.17 mmol) [6-isopropoxy-5-({[6-(trifluoromethyl)pyridine-2-yl]carbonyl}amino)-2H-indazol-2-yl]acetic acid (intermediate 11-f) and 1-methylpiperazine (25 mg, 1.5 eq.) in 2.3 mL THF and 0.25 mL DMF. After stirring at rt overnight the mixture was partly concentrated *in vacuo* and purified by HPLC to afford 30 mg (36% yield) of the title compound.
LC-MS (method C): Rt = 1.04 min; MS (ESIpos): m/z = 505 $[M+H]^+$
^{1}H NMR (DMSO-d_6, 500 MHz) δ 1.40 (d, 6H, *J*=6.0 Hz), 2.2–2.8 (br s), 3.4–3.7 (br s, 2H), 4.84 (td, 1H, *J*=6.0, 12.0 Hz), 5.39 (s, 2H), 7.14 (s, 1H), 8.20 (d, 1H, *J*=6.5 Hz), 8.21 (s, 1H), 8.40 (t, 1H, *J*=7.9 Hz), 8.45 (d, 1H, *J*=7.6 Hz), 8.74 (s, 1H), 10.73 (s, 1H).

Synthesis of compound 18

<u>Intermediate 18-a</u>

5-Amino-1H-indazol-6-ol

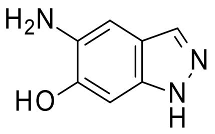

6.5 g (36.3 mmol) of 5-nitro-1H-indazol-6-ol (CAS No. 1082041-56-2) were dissolved in 1.5 L of methanol and hydrogenated with 193 mg (1.8 mmol) of palladium on activated carbon under standard hydrogen pressure at 25 °C for 5 h. This gave, after filtration using Celite®, 5.28 g (98% yield) of the title compound.
MS (ESIpos): m/z = 150 $(M+H)^+$
^{1}H NMR (DMSO-d_6, 400 MHz) δ 4.37 (br. s., 2H), 6.71–6.78 (m, 2H), 7.59 (s, 1H), 12.17 (br. s., 1H).

<u>Intermediate 18-b</u>

***tert*-Butyl (6-hydroxy-1H-indazol-5-yl)carbamate**

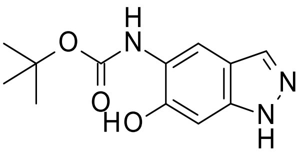

8.05 g (36.8 mmol) of di-*tert*-butyl dicarbonate were suspended in 125 mL of THF and 5.0 g (33.5 mmol) of 5-amino-1H-indazol-6-ol were added with stirring. The reaction mixture was stirred at 25 °C for 24 hand subsequently concentrated. the residue was taken up in methanol and 2 mL of 1 M aqueous sodium hydroxide solution and 2 mL of water were added. The mixture was stirred for another 30 min and the methanol was then distilled off. 1 M HCl was added to the residue until a pH of 7 had been reached. The mixture was then extracted with DCM and the combined organic layers were dried over sodium sulphate, filtered, and concentrated. This gave 7.50 g (90% yield) of the title compound.

MS (ESIpos): m/z = 250 $(M+H)^+$

^{1}H NMR (DMSO-d_6, 300 MHz) δ 1.47 (s, 9H), 6.88 (s, 1H), 7.66 (s, 1H), 7.82 (s, 1H), 7.91 (s, 1H), 10.19 (br. s., 1H), 12.50 (s, 1H).

Intermediate 18-c

***tert*-Butyl [6-(benzyloxy)-1H-indazol-5-yl]carbamate**

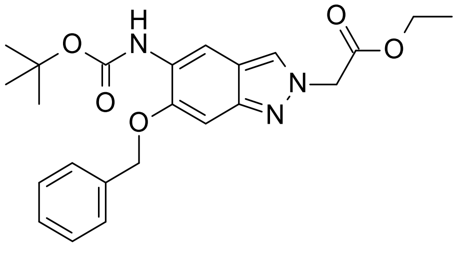

7.50 g (30.1 mmol) of *tert*-butyl (6-hydroxy-1H-indazol-5-yl)carbamate were dissolved in 150 mL of DMF and 5.66 g (33.1 mmol) of benzyl bromide and 8.32 g (60.2 mmol) of potassium carbonate were added with stirring. The reaction mixture was stirred at 25 °C for 24 h. The reaction mixture was then diluted with water and extracted with ethyl acetate. The combined organic phases were washed with saturated sodium chloride solution and the phases were separated and filtered through a water-repellent filter. The residue was taken up in DCM and adsorbed on ISOLUTE. The Isolute was applied to a Biotage SNAP cartridge (340 g; KP-Sil) pre-equilibrated with hexane and chromatography was carried out using the Isolera® flash purification system (Biotage, mobile phase: hexane/ethyl acetate). The combined product

fractions were concentrated and dried under reduced pressure. This gave 3.46 g (34% of theory) of the title product.

MS (ESIpos): m/z = 340 $(M+H)^+$

^{1}H NMR (CHLOROFORM-d, 300 MHz) δ 1.55 (s, 9H), 5.20 (s, 2H), 6.92 (s, 1H), 7.14 (s, 1H), 7.36–7.49 (m, 5H), 7.94 (d, *J*=0.75 Hz, 1H), 8.44 (s, 1H).

Intermediate 18-d

Ethyl {6-(benzyloxy)-5-[(tert-butoxycarbonyl)amino]-2H-indazol-2-yl}acetate

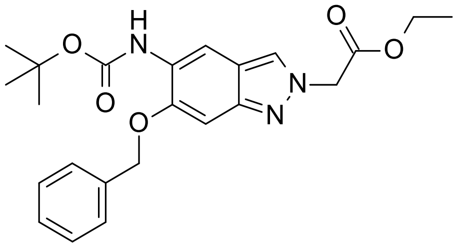

3.45 g (10.2 mmol) of tert-butyl [6-(benzyloxy)-1H-indazol-5-yl]carbamate), 2.26 mL (20.3 mmol) of ethyl bromoacetate, and 4.36 mL (20.3 mmol) of *N,N*-dicyclohexylmethylamine in 50 mL of THF were heated at 70 °C for 2 h. Another 2.26 mL (20.3 mmol) of ethyl bromoacetate and 4.36 mL (20.3 mmol) of *N,N*-dicyclohexylamine were added, and the mixture was stirred at 70 °C for a further 22 h and filtered, after which the filtrate was evaporated. Water and ethyl acetate were added to the residue and the organic layer was separated, the aqueous layer was extracted with ethyl acetate, and the combined organic layers were washed with 1N aqueous hydrochloride solution, saturated aqueous sodium bicarbonate solution, brine, filtered with a water-repellent filter, and concentrated. Purification by column chromatography using a Isolera® flash purification system (Biotage, mobile phase: hexane/ethyl acetate) gave 2.37 g (55% of theory) of the title compound.

MS (ESIpos): m/z = 426 $(M+H)^+$

^{1}H NMR (CHLOROFORM-d, 400 MHz) δ = 1.28 (t, *J*=7.20 Hz, 3 H) 1.54 (s, 9 H) 4.25 (q, *J*=7.24 Hz, 2 H) 5.09 (s, 2 H) 5.19 (s, 2 H) 7.03 (s, 1 H) 7.25 (s, 1 H) 7.32–7.49 (m, 5 H) 7.82 (s, 1 H) 8.30 (s, 1 H).

Intermediate 18-e

Ethyl [5-amino-6-(benzyloxy)-2H-indazol-2-yl]acetate

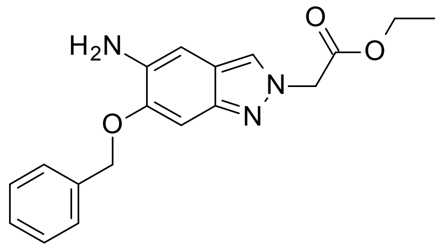

2.37 g (5.56 mmol) of ethyl {6-(benzyloxy)-5-[(tert-butoxycarbonyl)amino]-2H-indazol-2-yl}acetate were reacted with 3.24 mL (41.8 mmol) of trifluoroacetic acid in 25 mL of DCM at rt overnight. The mixture was poured into saturated aqueous sodium bicarbonate solution and stirred for 20 minutes. The organic layer was separated and the aqueous phase was extracted with DCM. The combined organic layers were washed with brine, dried with sodium sulfate, filtered, and dried again, affording 1.79 g (99% yield) of the title compound.

MS (ESIpos): m/z = 326 $(M+H)^+$

^{1}H NMR (CHLOROFORM-d, 400 MHz) δ 1.29 (t, 3 H, *J*=7.2 Hz), 4.25 (q, 2 H, *J*=7.2 Hz), 5.07 (s, 2 H), 5.15 (s, 2 H), 6.81 (s, 1 H), 7.01 (s, 1 H), 7.31–7.45 (m, 3 H), 7.45–7.52 (m, 2 H), 7.67 (s, 1 H).

Intermediate 18-f

Ethyl [6-(benzyloxy)-5-({[6-(trifluoromethyl)pyridin-2-yl]carbonyl}amino)-2H-indazol-2-yl]acetate

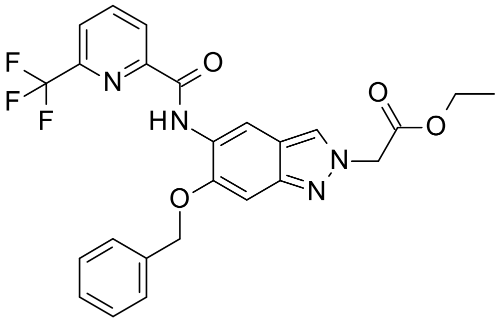

1.79 g (5.5 mmol) of ethyl [5-amino-6-(benzyloxy)-2H-indazol-2-yl]acetate, 1.26 g (6.6 mmol) of 6-(trifluoromethyl)pyridine-2-carboxylic acid, 842 mg (5.5 mmol) of HOBt, 2.11 g (11.0 mmol) of EDC, and 2.3 mL (16.5 mmol) of TEA were stirred in 75 mL of THF at 25 °C for 24 h. The reaction mixture was concentrated and water was added to the residue. The resulting solid was filtered off with suction and washed twice with water and twice with diethyl ether. The yellow solid was dried under reduced pressure. This gave 2.44 g (89% yield) of the title compound.

MS (ESIpos): m/z = 499 $(M+H)^+$

^{1}H NMR (DMSO-d$_6$, 400 MHz) δ 1.23 (t, 3H, *J*=7.1 Hz), 4.18 (q, 2H, *J*=7.2 Hz), 5.31 (s, 2H), 5.33 (s, 2H), 7.32 (s, 1H), 7.34–7.47 (m, 3H), 7.54–7.61 (m, 2H), 8.18 (d, *J*=7.58 Hz, 1H), 8.32–8.42 (m, 2H), 8.43–8.52 (m, 1H), 8.81 (s, 1H), 10.47 (s, 1H).

Intermediate 18-g

Ethyl [6-hydroxy-5-({[6-(trifluoromethyl)pyridin-2-yl]carbonyl}amino)-2H-indazol-2-yl]acetate

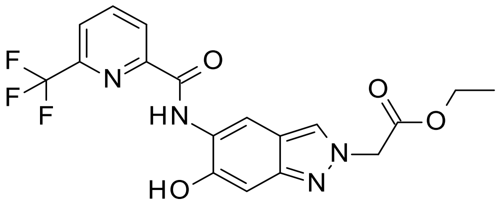

1.0 g (2.01 mmol) of ethyl [6-(benzyloxy)-5-({[6-(trifluoromethyl)pyridin-2-yl]carbonyl}amino)-2H-indazol-2-yl]acetate was dissolved in 40 mL of ethanol, after which the flask was evacuated and then flushed with nitrogen (this procedure was repeated two more times). 213 mg (0.2 mmol) of palladium on carbon were added and the flask was evacuated and flushed with hydrogen. The reaction mixture was hydrogenated under standard hydrogen pressure at 25 °C for 6 h. The reaction mixture was then filtered through a PTFE filter with Celite® and concentrated, affording 783 mg (96% yield) of product.

MS (ESIpos): m/z = 409 $(M+H)^+$

^{1}H NMR (DMSO-d$_6$, 400 MHz) δ = 1.22 (t, 3H, *J*=7.1 Hz,) 4.17 (q, 2 H, *J*=7.2 Hz) 5.28 (s, 2H) 6.92 (s, 1H) 8.21 (d, 1H, *J*=7.33 Hz,) 8.27 (s, 1H) 8.40 (t, 1H, *J*=7.83 Hz,) 8.47 (d, 1H, *J*=7.58 Hz,) 8.70 (s, 1H) 10.55 (s, 1H) 10.72 (s, 1H)

Intermediate 18-h

Ethyl [6-(cyclopropylmethoxy)-5-({[6-(trifluoromethyl)pyridin-2-yl]carbonyl}amino)-2H-indazol-2-yl]acetate

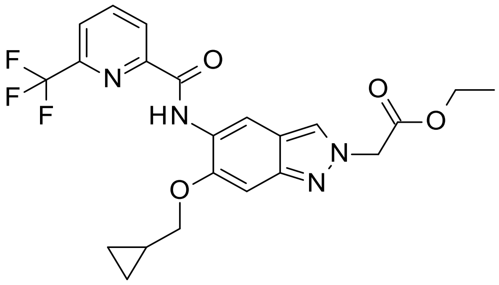

200 mg (0.49 mmol) ethyl [6-hydroxy-5-({[6-(trifluoromethyl)pyridin-2-yl]carbonyl}amino)-2H-indazol-2-yl]acetate were dissolved in 8.6 mL DMF and 203 mg (3.0 eq.) potassium carbonate and 71 µL (1.3 eq.) (bromomethyl)cyclopropane were added. After stirring for 1 h at 100 °C in a microwave reactor, water was added and the mixture was extracted with ethyl acetate. The combined organic layers were washed with brine and concentrated to afford 243 mg of the title compound which was used without further purification in the next step.

^{1}H NMR (CHLOROFORM-d, 400 MHz) δ 0.38–0.50 (m, 2H), 0.69–0.84 (m, 2H), 1.30 (t, *J*=7.16 Hz, 3H), 1.45 (br. s., 1H), 3.98 (d, *J*=6.97 Hz, 2H), 4.27 (q, *J*=7.16 Hz, 2H), 5.15 (s, 2H), 6.98 (s, 1H), 7.87 (d, *J*=7.54 Hz, 1H), 7.93 (s, 1H), 8.13 (t, *J*=7.72 Hz, 1H), 8.51 (d, *J*=7.72 Hz, 1H), 8.88 (s, 1H), 10.91 (s, 1H).

Intermediate 18-i

[6-(cyclopropylmethoxy)-5-({[6-(trifluoromethyl)pyridin-2-yl]carbonyl}amino)-2H-indazol-2-yl]acetic acid

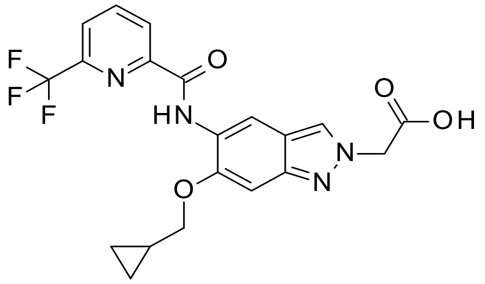

220 mg (0.48 mmol) ethyl [6-(cyclopropylmethoxy)-5-({[6-(trifluoromethyl)pyridin-2-yl]carbonyl}amino)-2H-indazol-2-yl]acetate were dissolved in 10 mL THF and 57 mg (5.0 eq.) lithium hydroxide and 0.9 mL water were added, after which the mixture was stirred for 4 h at rt and acidified to pH = 3 by the addition of 1N aqueous hydrochloride solution. The resulting solid was filtered off with suction, washed with suction, and dried to afford 181 mg (88% yield) of the title compound.

^{1}H NMR (DMSO-d$_6$, 400 MHz) δ 0.42–0.48 (m, 2H), 0.63–0.69 (m, 2H), 1.29–1.41 (m, 1H), 4.03 (d, *J*=6.82 Hz, 2H), 5.20 (s, 2H), 7.07 (s, 1H), 8.21 (dd, *J*=7.71, 0.88 Hz, 1H), 8.29 (s, 1H), 8.37–8.44 (m, 1H), 8.46–8.50 (m, 1H), 8.76 (s, 1H), 10.71 (s, 1H).

Compound 18 (step 18-j)

N-{6-(Cyclopropylmethoxy)-2-[2-(4-methylpiperazin-1-yl)-2-oxoethyl]-2H-indazol-5-yl}-6-(trifluoromethyl)pyridine-2-carboxamide

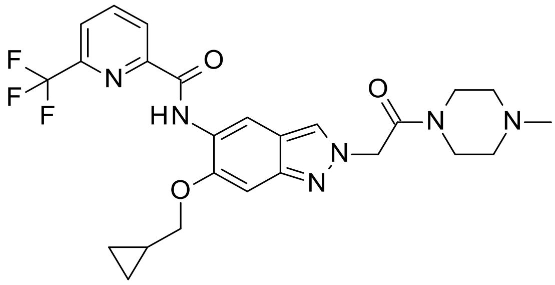

40 mg (0.092 mmol) [6-(cyclopropylmethoxy)-5-({[6-(trifluoromethyl)pyridin-2-yl]carbonyl}amino)-2H-indazol-2-yl]acetic acid were dissolved in 1.5 mL DMF. 35.3 mg (2.0 eq.) EDC, 14 mg (1.0 eq.) HOBt, 30 µL (3.0 eq.) TEA, and 11 mg (1.2 eq.) 1-methylpiperazine were added and the mixture was stirred at rt overnight and filtered. The filtrate was purified by HPLC affording 36.5 mg (75% yield) of the title compound.

LC-MS (method D): Rt = 1.20 min; MS (ESIpos): m/z = 517 $[M+H]^+$

^{1}H NMR (DMSO-d_6, 400 MHz) δ 0.39–0.51 (m, 2H), 0.57–0.70 (m, 2H), 1.27–1.43 (m, 1 H), 2.21 (s, 3H), 2.29 (t, 2H, *J*=4.8 Hz), 2.34–2.39 (m, 2H), 3.43–3.49 (m, 2H), 3.51–3.57 (m, 2H), 4.03 (d, 2H, *J*=6.8 Hz), 5.37 (s, 2H), 7.05 (s, 1H), 8.19–8.23 (m, 2H), 8.41 (t, 1H, *J*=7.8 Hz), 8.48 (d, 1H, *J*=7.8 Hz), 8.76 (s, 1H), 10.71 (s, 1H).

Synthesis of compound 19

<u>Compound 19 (step 19-a)</u>

N-{6-Methoxy-2-[2-(morpholin-4-yl)-2-oxoethyl]-2H-indazol-5-yl}-6-(trifluoromethyl)pyridine-2-carboxamide

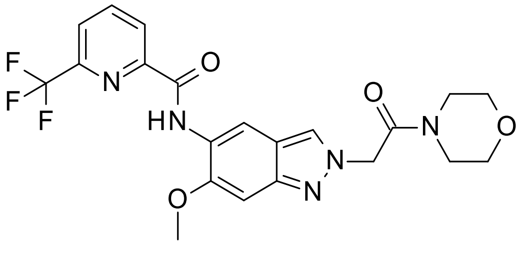

169 mg [6-methoxy-5-({[6-(trifluoromethyl)pyridin-2-yl]carbonyl}amino)-2H-indazol-2-yl]acetic acid (intermediate 14-c, contained approximately 1 eq. TFA) in 13 mL THF and 1.3 mL DMF were treated with 127 mg EDC, 50.9 mg HOBt and 185 µL TEA and the mixture was stirred at rt overnight. The mixture was partly concentrated and poured into 25 mL water. The resulting solid was filtered off with suction, washed with water and diethylether and dried to afford 138 mg of the title compound.

LC-MS (method C): Rt = 1.07 min; MS (ESIpos): m/z = 464 $[M+H]^+$

^{1}H NMR (DMSO-d_6, 400 MHz) δ 3.41–3.51 (m, 2H), 3.55–3.62 (m, 4H), 3.62–3.68 (m, 2H), 3.99 (s, 3H), 5.40 (s, 2H), 7.12 (s, 1H), 8.19–8.26 (m, 2H), 8.40 (t, 1H, *J*=7.7 Hz), 8.47 (d, 1H, *J*=7.6 Hz), 8.71 (s, 1H), 10.51 (s, 1H).

Synthesis of compound 20

<u>Compound 20 (step 20-a)</u>

N-{6-Ethoxy-2-[2-(morpholin-4-yl)-2-oxoethyl]-2H-indazol-5-yl}-6-(trifluoromethyl)pyridine-2-carboxamide

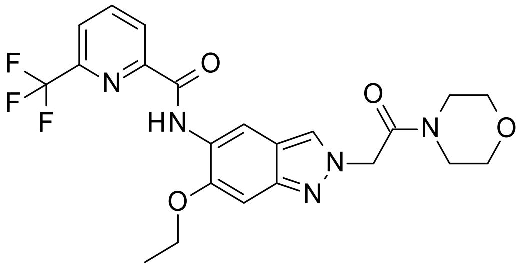

23.3 mg (1.3 eq.) morpholine, 78.9 mg EDC, 31.5 mg (HOBt) and 86 µL TEA were added to 84.0 mg (0.21 mmol) [6-ethoxy-5-({[6-(trifluoromethyl)pyridin-2-yl]carbonyl}amino)-2H-indazol-2-yl]acetic acid (intermediate 10-c) in 2.0 mL DMF and the mixture was stirred at 50 °C overnight. Water and ethyl acetate were added and the resulting solid was filtered off with suction, washed three times with water and three times with diethylether, and dried to afford 90.3 mg (89% yield) of the title compound.

LC-MS (method A): Rt = 1.16 min; MS (ESIpos): m/z = 478 $[M+H]^+$

^{1}H NMR (DMSO-d_6, 300 MHz) δ 1.49 (t, 3H, *J*=6.9 Hz), 3.4–3.7 (m), 4.20 (q, 2H, *J*=6.7 Hz), 5.38 (s, 2H), 7.07 (s, 1H), 8.2–8.2 (m, 2H), 8.4–8.5 (m, 2H), 8.71 (s, 1H), 10.73 (s, 1H)

Synthesis of compound 21

<u>Intermediate 21-a</u>

***N*-(6-Methoxy-1H-indazol-5-yl)-6-methylpyridine-2-carboxamide**

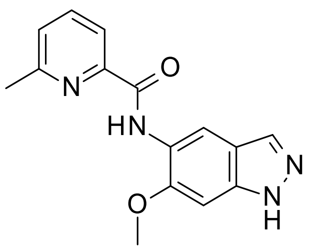

5.00 g (30.64 mmol) of 6-methoxy-1H-indazole-5-amine (CAS Number 749223-61-8) and 4.62 g (33.70 mmol) of 6-methylpyridine-2-carboxylic acid were dissolved in 100 mL of THF and stirred with 4.69 g (30.64 mmol) of HOBt, 11.74 g (61.28 mmol) of EDC, and 21.35 mL (153.2 mmol) of TEA at 25 °C for 20 h. Water was added and the reaction mixture was concentrated. The resulting precipitate was filtered off with suction, washed three times with water and three times with diethylether and dried in a drying cabinet. This gave 7.89 g (65% yield) of the title compound.
MS (ESIpos): m/z = 283 $(M+H)^+$

<u>Intermediate 21-b</u>

Benzyl (6-methoxy-5-{[(6-methylpyridin-2-yl)carbonyl]amino}-2H-indazol-2-yl)acetate

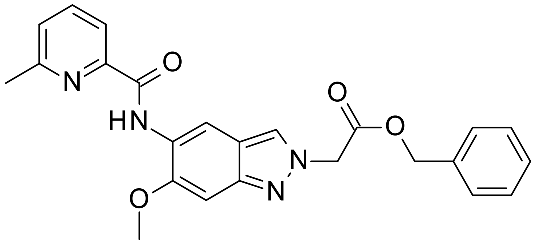

7.57 g (19.0 mmol) of *N*-(6-methoxy-1H-indazol-5-yl)-6-methylpyridine-2-carboxamide were stirred with 6.03 mL (38.1 mmol) of benzyl bromoacetate in 100 mL of THF in the presence of 8.01 mL (38.1 mmol) of *N*,*N*-dicyclohexylmethylamine at 70 °C for 2.5 h and at 60 °C for 17 h. Another 3.02 mL (19.1 mmol) of benzyl bromoacetate and 4.01 mL (19.1 mmol) of *N*,*N*-dicyclohexylmethylamine were added and the mixture was stirred at 70 °C for a further 24 h. The solid was filtered off with suction and washed with ethyl acetate. The filtrate was filtered once more and washed twice with ethyl acetate and the solid was dried. Water was added to the filtrate and, after phase separation, the aqueous phase was washed once more with ethyl acetate. The combined organic phases were washed with brine, filtered through a hydrophobic filter, and concentrated. Ethyl acetate was added to the crude product, and the mixture was stirred for 15 minutes. The solid was filtered off with suction, washed three times with ethyl acetate and dried. This gave a total of 6.02 g (63% yield) of the title compound.

MS (ESIpos): m/z = 431 (M+H)+

^{1}H NMR (DMSO-d_6, 500 MHz) δ 2.63 (s, 3H), 4.01 (s, 3H), 5.21 (s, 2H), 5.40 (s, 2H), 7.11 (s, 1H), 7.34–7.40 (m, 5H), 7.55 (dd, 1H, *J*=1.0, 7.3 Hz), 7.93–8.02 (m, 2H), 8.30–8.33 (m, 1 H), 8.73 (s, 1H), 10.72 (s, 1H).

Intermediate 21-c

(6-Methoxy-5-{[(6-methylpyridin-2-yl)carbonyl]amino}-2H-indazol-2-yl)acetic acid

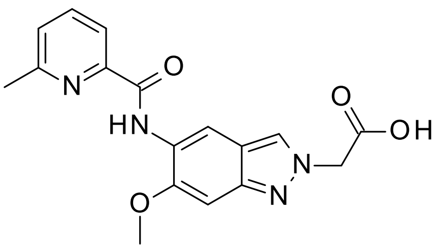

2.28 g (3.92 mmol, crude batch, purity 74% according to HPLC analysis) of benzyl (6-methoxy-5-{[(6-methylpyridin-2-yl)carbonyl]amino}-2H-indazol-2-yl)acetate were dissolved in 20 mL of THF and 3.0 mL of methanol, a solution of 1.65 g (39.2 mmol) of lithium hydroxide monohydrate in 3.0 mL of water was then added. The mixture was diluted with water and acidified to pH 4 using aqueous citric acid (10%). The precipitated solid was filtered off, washed three times with water and three times with diethylether and dried under reduced pressure. This gave 2.43 g of the title compound as a crude product.

MS (ESIpos): m/z = 341 (M+H)+

Compound 21 (step 21-d)

N-{6-Methoxy-2-[2-(morpholin-4-yl)-2-oxoethyl]-2H-indazol-5-yl}-6-methylpyridine-2-carboxamide

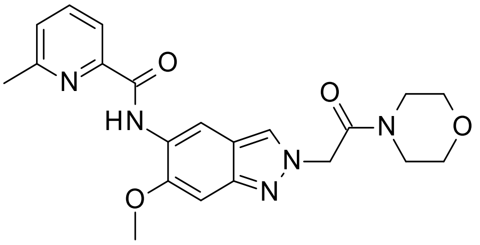

100 mg (0.29 mmol) (6-Methoxy-5-{[(6-methylpyridin-2-yl)carbonyl]amino}-2H-indazol-2-yl)acetic acid in DMF (2.0 ml) was treated with 51 mg (2.0 eq.) morpholine, 113 mg (2.0 eq.) EDC, and 45 mg (1.0 mg) HOBt and the mixture was stirred for 20 h at RT. Water was added

and the resulting solid was filtered with suction and washed with water and diethylether and dried resulting in 54.2 mg (43% yield) of the title compound.
LC-MS (method A): Rt = 1.00 min; MS (ESIpos): m/z = 410 $[M+H]^+$
^{1}H NMR (DMSO-d_6, 400 MHz) δ 2.63 (s, 3H), 3.47 (s, 2H), 3.53–3.62 (m, 4H), 3.64 (s, 2H), 4.01 (s, 3H), 5.39 (s, 2H), 7.09 (s, 1H), 7.56 (dd, 1H, *J*=7.1, 1.5 Hz,), 7.93–8.03 (m, H), 8.21 (s, 1H), 8.72 (s, 1H), 10.71 (s, 1H).

Synthesis of compound 22

Intermediate 22-a

Benzyl (5-amino-6-methoxy-2H-indazol-2-yl)acetate

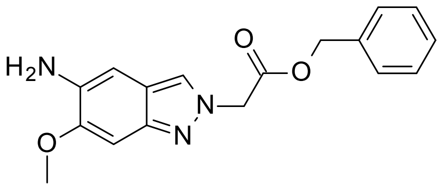

Similar to the preparation of intermediate 7-e, 25.7 g (60.1 mmol) of benzyl {5-[(tert-butoxycarbonyl)amino]-6-methoxy-2H-indazol-2-yl}acetate (intermediate 23-b) were reacted with 23.1 mL (300 mmol) of TFA. This gave 20.5 g (98% yield) of the title compound.
MS (ESIpos): m/z = 312 $(M+H)^+$

Intermediate 22-b

Benzyl [5-({[6-(difluoromethyl)pyridin-2-yl]carbonyl}amino)-6-methoxy-2H-indazol-2-yl]acetate

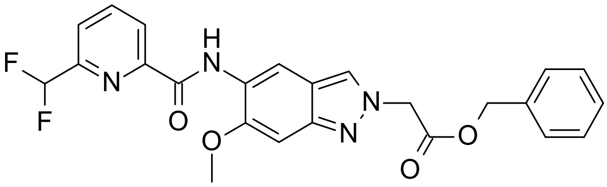

660 mg (2.12 mmol) benzyl (5-amino-6-methoxy-2H-indazol-2-yl)acetate and 440 mg (2.54 mmol, 1.2 eq.) 6-(difluoromethyl)pyridine-2-carboxylic acid in 20 mL THF were treated with 325 mg (1.0 eq.) HOBt, 813 mg (2.0 eq.) EDC, and 0.89 mL (3.0 eq.) TEA and the mixture was stirred at RT for 19 h. Water was added and the resulting solid was filtered off, washed with water, and dried to afford 613 mg (53% yield) of the title compound.

MS (ESIpos): m/z = 312 $(M+H)^+$

Intermediate 22-c

[5-({[6-(Difluoromethyl)pyridin-2-yl]carbonyl}amino)-6-methoxy-2H-indazol-2-yl]acetic acid

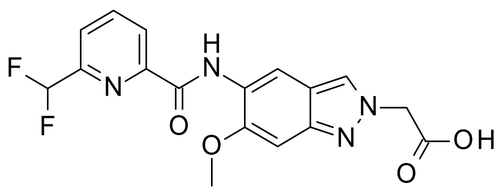

Similar to the preparation of intermediate 7-c, 613 mg of benzyl [5-({[6-(difluoromethyl)pyridin-2-yl]carbonyl}amino)-6-methoxy-2H-indazol-2-yl]acetate were stirred at rt with 469 mg of lithium hydroxide monohydrate in 3 mL of water, 15 mL of THF, and 1 mL of methanol for 3 h. This gave, after analogous work-up, 378 mg of the title compound.
MS (ESIpos): m/z = 312 $(M+H)^+$

Compound 22 (step 22-d)

6-(Difluoromethyl)-N-{6-methoxy-2-[2-(morpholin-4-yl)-2-oxoethyl]-2H-indazol-5-yl}pyridine-2-carboxamide

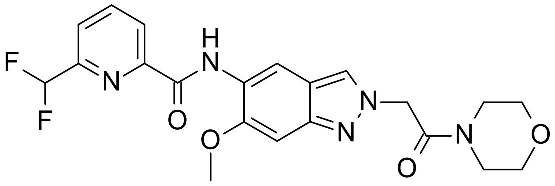

48 mg (1.5 eq.) morpholine, 142 mg (2 eq.) EDC, 56.6 mg HOBt (1.0 eq.) and 154 microliter (3.0 eq.) TEA were added to 139 mg (0.369 mmol) [5-({[6-(difluoromethyl)pyridin-2-yl]carbonyl}amino)-6-methoxy-2H-indazol-2-yl]acetic acid in 3.0 mL THF and the mixture was stirred for 22 h at rt. Water was added and the mixture was stirred for 10 min and the resulting solid was filtered with suction, washed three times with water and three times with diethylether, and dried to afford 121 mg (73% yield) of the title compound.
LC-MS (method A): Rt = 0.99 min; MS (ESIpos): m/z = 446 $[M+H]^+$
^1H NMR (DMSO-d_6, 400 MHz) δ 3.4–3.5 (m, 2H), 3.5–3.7 (m, 6H), 3.99 (s, 3H), 5.39 (s, 2H), 7.01 (s, 1H), 7.10 (s, 1H), 7.14 (s, 1H), 7.28 (s, 1H), 7.99 (d, 1H, *J*=7.3 Hz), 8.22 (s, 1H), 8.3-8.4 (m, 2H), 8.70 (s, 1H), 10.55 (s, 1H).

Synthesis of compound 23 and compound 24

Intermediate 23-a

***tert*-Butyl (6-methoxy-1H-indazol-5-yl)carbamate**

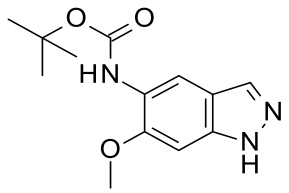

4.0 g (24.5 mmol) of 6-methoxy-1H-indazol-5-amine (CAS Number 749223-61-8) were dissolved in 30 mL of THF and 5.35 g (24.5 mmol) of di-tert-butyl dicarbonate were added. The reaction mixture was stirred at 25 °C for 18 h, concentrated, and the residue was suspended in 20 mL of DCM. 200 mL of hexane were added and the resulting suspension was stirred in an ice bath for 25 minutes. The precipitate was filtered off with suction, washed twice with 25 mL of hexane and dried. This gave 4.83 g (75% yield) of the title compound.
MS (ESIpos): m/z = 264 $(M+H)^+$
^1H NMR (CHLOROFORM-d, 400 MHz) δ 1.56 (s, 9H), 3.95 (s, 3H), 6.88 (s, 1H), 7.12 (br. s., 1H), 7.94 (d, 1H, *J*=0.76 Hz), 8.40 (br. s., 1H).

Intermediate 23-b

Benzyl {5-[(*tert*-butoxycarbonyl)amino]-6-methoxy-2H-indazol-2-yl}acetate

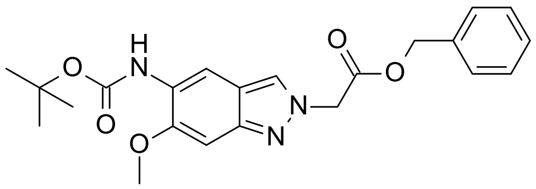

4.17 g (15.8 mmol) of *tert*-butyl (6-methoxy-1H-indazol-5-yl)carbamate in 50 mL of THF were stirred with 2.51 mL (15.8 mmol) of benzyl bromoacetate and 3.36 mL (15.8 mmol) of *N*,*N*-dicyclohexylmethylamine at 65 °C for 4 h, 2.51 mL (15.8 mmol) of benzyl bromoacetate and 3.36 mL (15.8 mmol) of *N*,*N*-dicyclohexylmethylamine were then added and the mixture was stirred at 65 °C for a further 18 h. Work-up was performed as described for the synthesis of intermediate 7-b. Purification by column chromatography was then performed using the Isolera® flash purification system (Biotage) (mobile phase: hexane/ethyl acetate) and gave 3.22 g (47% yield) of the title compound.

MS (ESIpos): m/z = 412 $(M+H)^+$

^{1}H NMR (DMSO-d_6, 500 MHz) δ 1.47 (s, 9H), 3.86 (s, 3H), 5.20 (s, 2H), 5.37 (s, 2H), 6.97 (s, 1H), 7.28–7.42 (m), 7.79 (s, 1H), 7.94 (br. s., 1H), 8.21 (s, 1H).

Intermediate 23-c

{5-[(*tert*-Butoxycarbonyl)amino]-6-methoxy-2H-indazol-2-yl}acetic acid

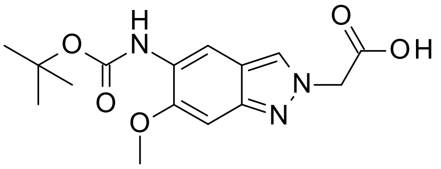

1.13 g (26.8 mmol) lithium hydroxide hydrate in 10 mL water were added to 937 mg (2.68 mmol) benzyl {5-[(*tert*-butoxycarbonyl)amino]-6-methoxy-2H-indazol-2-yl}acetate in 20 mL THF and 1.5 mL ethanol and the mixture was stirred at rt overnight. Water was added and then aqueous citric acid solution (10% citric acid) was added until a pH of 4 was reached. A small amount of ethyl acetate was added and the resulting solid was filtered off, washed two times with water and three times with diethylether, and dried to afford 548 mg of the title compound.

^{1}H NMR (DMSO-d_6, 500 MHz) δ 1.47 (s, 9H), 3.86 (s, 3H), 5.16 (s, 2H), 6.96 (s, 1H), 7.78 (s, 1H), 7.93 (br. s., 1H), 8.16 (d, 1H), 13.13 (br. s., 1H).

Intermediate 23-d

***tert*-butyl {6-methoxy-2-[2-(morpholin-4-yl)-2-oxoethyl]-2H-indazol-5-yl}carbamate**

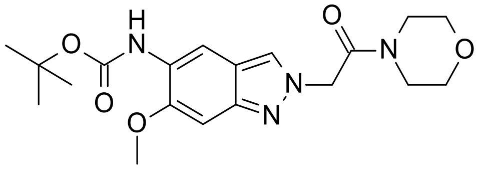

1.00 g (3.11 mmol) ({5-[(*tert*-butoxycarbonyl)amino]-6-methoxy-2H-indazol-2-yl}acetic acid and 407 mg (1.5 eq.) morpholine in 40 mL THF were treated with 477 mg (1.0 eq.) HOBt, 1.19 g (2.0 eq.) EDC, and 1.30 mL (3 eq.) TEA and the mixture was stirred for 70 h at rt. Water was added and the mixture was extracted with ethyl acetate three times. The combined organic layers were washed with brine and filtered through a water-repellent filter and concentrated to afford 1.47 g of the title compound which was used without further purification.

MS (ESIpos): m/z = 391 $(M+H)^+$

^{1}H NMR (300 MHz, CHLOROFORM-*d*) δ 1.55 (s, 9H) 3.58 (s, 4) 3.66 (s, 4H) 3.93 (s, 3H) 5.18 (s, 2H) 6.94 (s, 1H) 7.22 (s, 1H) 7.81 - 7.90 (m, 1H) 8.25 (s, 1H)

<u>Intermediate 23-e</u>

2-(5-Amino-6-methoxy-2H-indazol-2-yl)-1-(morpholin-4-yl)ethanone

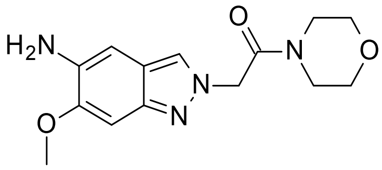

2.87 mL (10 eq.) TFA were added to 1.47 g (3.73 mmol) *tert*-butyl {6-methoxy-2-[2-(morpholin-4-yl)-2-oxoethyl]-2H-indazol-5-yl}carbamate in 15 mL DCM and the mixture was stirred at rt for 15.5 h and concentrated. Toluene was added and was removed a total of three times, affording 2.43 g of a residue that was purified by HPLC (method, table S7), affording 670 mg (62% yield) of the title compound.

Table S7. HPLC methodology.

HPLC system:	Waters autopurification system: Pump 254, Sample Manager 2767, CFO, DAD 2996, ELSD 2424, SQD 3100
Column:	XBrigde C18 5µm 100x30 mm
Solvent:	A = water+0.2% Vol. NH_3 (32%)
	B = methanol
Gradient:	0-8 min 5-30% B
Flow:	70 mL/min
temperature:	RT
Detection:	DAD scan range 210–400 nm
	MS ESI+, ESI-, scan range 160-1000 m/z
	ELSD

^{1}H NMR (DMSO-d_6, 400 MHz) δ 7.75 (s, 1H), 6.78 (s, 1H), 6.62 (s, 1H), 5.24 (s, 2H), 4.60 (br s, 2H), 3.81 (s, 3H), 3.5–3.6 (m, 6H), 3.4–3.5 (m, 2H).

<u>**Compound 23 and Compound 24 (step 23/24-f)**</u>

N-[6-Methoxy-2-(2-morpholino-2-oxo-ethyl)indazol-5-yl]-6-[(1*R*)-2,2,2-trifluoro-1-hydroxy-ethyl]pyridine-2-carboxamide (compound 23)

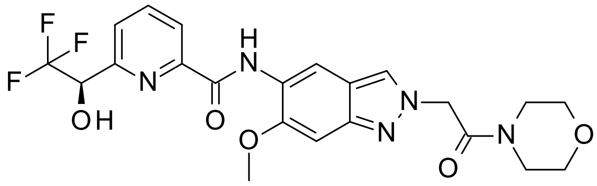

N-[6-Methoxy-2-(2-morpholino-2-oxo-ethyl)indazol-5-yl]-6-[(1S)-2,2,2-trifluoro-1-hydroxy-ethyl]pyridine-2-carboxamide (compound 24)

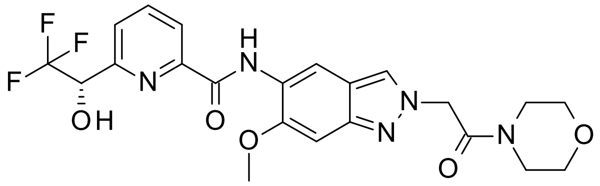

200 mg (0.689 mmol) 2-(5-amino-6-methoxy-2H-indazol-2-yl)-1-(morpholin-4-yl)ethanone and 238 mg (potassium 6-(2,2,2-trifluoro-1-hydroxyethyl)pyridine-2-carboxylate) in 5.0 mL THF were treated with 105 mg (1.0 eq.) HOBt, 264 mg (2.0 eq.) EDC, and 0.57 mL (6.0 eq.) TEA and the mixture was stirred for 17 h at rt. After the addition of water, the mixture was extracted three times with ethylacetat and the combined organic layers were evaporated. Chiral HPLC purification (methods see Table S8 and Table S9) afforded 70.0 mg (compound **24**) and 59.0 mg (compound **23**). The absolute configuration of compound **23** was determined by X-ray crystallography.

Table S8. Chiral HPLC methodology.

System:	Waters: Alliance 2695, DAD 996, ESA: Corona
Column:	Chiralpak IC 5µm 150x4.6 mm
Solvent:	Ethanol / Methanol 50:50 (v/v)
Flow:	1.0 mL/min
Temperature:	25 °C
Solution:	1.0 mg/mL EtOH/MeOH 1:1
Injection:	5.0 µL
Detection:	DAD 280 nm

Table S9. Chiral HPLC methodology (Preparation).

System:	Agilent: Prep 1200, 2xPrep Pump, DLA, MWD, Gilson: Liquid Handler 215		
Column:	Chiralpak IC 5µm 250x30 mm		
Solvent:	Ethanol / Methanol 50:50 (v/v)		
Flow:	35 mL/min		
Temperature:	RT		
Solution:	401 mg / 8 mL DCM/MeOH		
Injection:	10 x 0.8 mL		
Detection:	UV 280 nm		
Compound	Rt in min	Purity in %	Amount in mg
**24**	8.0–8.7	98.8	70
**23**	10.1–11.1	99.1	59
Workup:	Fractions were evaporated, treated with *t*-BuOH, cooled to -65 °C, and freeze-dried.		

NMR data for compound **23**:

^{1}H NMR (DMSO-d$_6$, 300 MHz) δ 3.4–3.5 (m, 2H), 3.5–3.7 (m, 6H), 3.97 (s, 3H), 5.3–5.4 (m, 1H), 5.39 (s, 2H), 7.08 (s, 1H), 7.29 (d, 1H, *J*=6.2 Hz), 7.91 (t, 1H, *J*=4.4 Hz), 8.2–8.2 (m, 3H), 8.66 (s, 1H), 10.69 (s, 1H).

Synthesis of compound 25

Compound 25 (step 25-a)

6-(1-Hydroxyethyl)-N-{6-methoxy-2-[2-(morpholin-4-yl)-2-oxoethyl]-2H-indazol-5-yl}pyridine-2-carboxamide

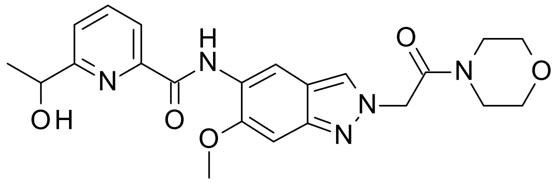

200 mg (689 µmol) 2-(5-amino-6-methoxy-2H-indazol-2-yl)-1-(morpholin-4-yl)ethanone (intermediate 23-e) and 212 mg (crude, ap. 1.5 eq.) potassium 6-(1-hydroxyethyl)pyridine-2-carboxylate (prepared by the reaction of methyl 6-(1-hydroxyethyl)pyridine-2-carboxylate with potassium hydroxide at 50 °C and subsequent evaporation of solvent) in 5.0 mL THF were treated with 105 mg (1.5 eq.) HOBt, 264 mg (2.0 eq.) EDC, and 0.58 mL (6.0 eq.) TEA and the mixture was stirred at rt for 18 h. Water and a small amount of ethyl acetate were added. The resulting solid was filtered off, washed two times with water, three times with diethylether, and dried to afford 205 mg (64% yield) of the title compound.
LC-MS (method A): Rt = 0.85 min; MS (ESIpos): m/z = 440 $[M+H]^+$
^1H NMR (DMSO-d_6, 300 MHz) δ 1.51 (d, 3H, *J*=6.6 Hz), 3.4–3.5 (m, 2H), 3.5–3.7 (m, 6H), 3.99 (s, 3H), 4.8–4.9 (m, 1H), 5.38 (s, 2H), 5.58 (d, 1H, *J*=4.9 Hz), 7.09 (s, 1H), 7.8-7.8 (m, 1H), 8.0-8.1 (m, 2H), 8.20 (s, 1H), 8.68 (s, 1H), 10.78 (s, 1H).

Synthesis of compound 26

<u>Intermediate 26-a</u>

Benzyl [5-({[6-(2-hydroxypropan-2-yl)pyridin-2-yl]carbonyl}amino)-6-methoxy-2H-indazol-2-yl]acetate

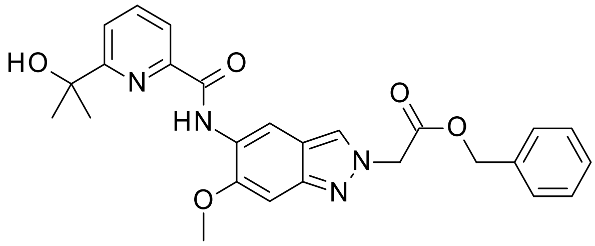

300 mg (0.96 mmol) of benzyl (5-amino-6-methoxy-2H-indazol-2-yl)acetate (intermediate 22-a), 295 mg (1.16 mmol) of potassium 6-(2-hydroxypropan-2-yl)pyridine-2-carboxylate (prepared by the reaction of 535 mg methyl 6-(2-hydroxypropan-2-yl)pyridine-2-carboxylate (CAS Number 1799836-56-8) with 282 mg (2.0 eq.) potassium hydroxide in 6 mL methanol at 50 °C for 3 h and evaporation to dryness), 148 mg (0.96 mmol) of HOBt, 277 mg (1.45

mmol) of 1-(3-dimethylaminopropyl)-3-ethylcarbodiimide hydrochloride, and 403 µL (2.89 mmol) of TEA in 10 mL of THF were stirred at 25 °C for 24 h. The reaction mixture was diluted with water and extracted three times with ethyl acetate. The combined organic phases were washed with brine and concentrated. The crude product was dissolved in 4 mL of DMSO and purified by preparative HPLC. The product fractions were lyophilized. This gave 209 mg (46% yield) of the title compound.
MS (ESIpos): m/z = 475 $(M+H)^+$
^{1}H NMR (DMSO-d_6, 300 MHz) δ 1.56 (s, 6H), 3.99 (s, 3H), 5.20 (s, 2H), 5.43 (d, 3H, *J*=17.5 Hz), 7.12 (s, 1H), 7.3–7.4 (m, 5H), 7.93 (dd, 1H, *J*=1.4, 7.6 Hz), 8.0–8.0 (m, 1H), 8.05 (d, 1H, *J*=7.5 Hz), 8.32 (s, 1H), 8.68 (s, 1H), 10.93 (s, 1H).

Intermediate 26-b

2-[5-[[6-(1-Hydroxy-1-methyl-ethyl)pyridine-2-carbonyl]amino]-6-methoxy-indazol-2-yl]acetic acid

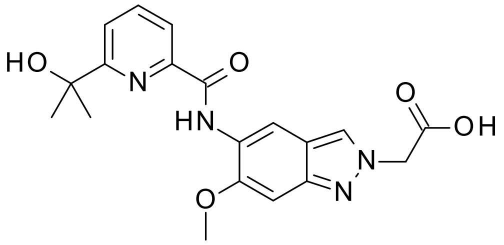

206 mg (0.43 mmol) benzyl [5-({[6-(2-hydroxypropan-2-yl)pyridin-2-yl]carbonyl}amino)-6-methoxy-2H-indazol-2-yl]acetate was suspended in 10 mL of THF and 1.0 mL of methanol, a solution of 182 mg (4.33 mmol) of lithium hydroxide monohydrate in 1.5 mL of water was then added and the mixture was stirred at 25 °C for 24 h. The mixture was diluted with water, acidified to pH 4 using aqueous citric acid solution (10% citric acid) and concentrated. The precipitated solid was filtered off, washed once with water and three times with diethylether and dried under reduced pressure. This gave 155 mg (93% yield) of the title compound.
MS (ESIpos): m/z = 421 $(M+H)^+$
^{1}H NMR (DMSO-d_6, 400 MHz) δ 1.57 (s, 6H), 3.99 (s, 3H), 5.20 (s, 2H), 5.47 (s, 1H), 7.12 (s, 1H), 7.93 (dd, 1H, *J*=7.5, 1.3 Hz), 7.98–8.11 (m, 2H), 8.28 (s, 1H), 8.68 (s, 1H), 10.93 (s, 1H).

Compound 26 (step 26-c)

6-(2-Hydroxypropan-2-yl)-N-{6-methoxy-2-[2-(morpholin-4-yl)-2-oxoethyl]-2H-indazol-5-yl}pyridine-2-carboxamide

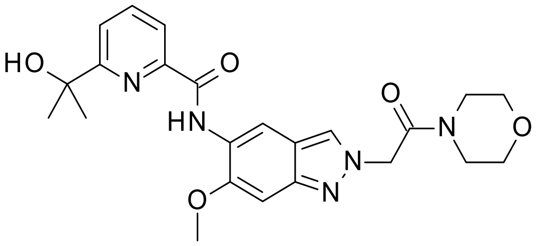

58 mg (1.0 eq.) HOBt, 144 mg (2.0 eq.) EDC, and 0.16 mL (3.0 eq.) triethylamine were added to 145 mg (0.38 mmol) 2-[5-[[6-(1-Hydroxy-1-methyl-ethyl)pyridine-2-carbonyl]amino]-6-methoxy-indazol-2-yl]acetic acid and 49 mg (1.5 eq.) morpholine in 3.0 mL THF and the mixture was stirred at rt for 67 h. Water was added, resulting in the precipitation of a solid. The solid was filtered off, washed with water, diethylether, and dried *in vacuo* affording 120 mg of the title compound.

LC-MS (method A): Rt = 0.89 min; MS (ESIpos): m/z = 454 $[M+H]^+$

^1H NMR (DMSO-d_6, 400 MHz) δ 1.57 (s, 6H), 3.42–3.52 (m, 2H), 3.52–3.62 (m, 4H), 3.62–3.68 (m, 2H), 3.99 (s, 3H), 5.39 (s, 2H), 5.47 (s, 1H), 7.10 (s, 1H), 7.93 (dd, 1H, *J*=1.3, 7.6 Hz), 7.99–8.10 (m, 2H), 8.19–8.23 (m, 1H), 8.68 (s, 1H), 10.93 (s, 1H).

Synthesis of compound 27

<u>**Compound 27 (step 27-a)**</u>

N-{6-Methoxy-2-[2-(morpholin-4-yl)-2-oxoethyl]-2H-indazol-5-yl}-6-[(2-methylpropyl)amino]pyridine-2-carboxamide

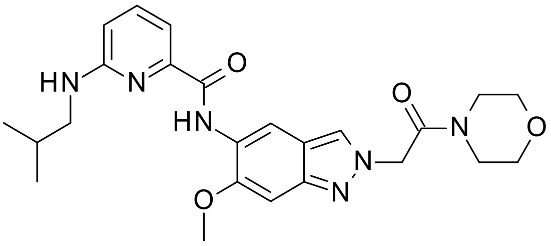

40 mg (0.17 mmol) 2-(5-amino-6-methoxy-2H-indazol-2-yl)-1-(morpholin-4-yl)ethanone (intermediate 23-e) were dissolved in 1.0 mL DMF. 53 mg (0.28 mmol) EDC, 21 mg (1.0 eq.) HOBt, 58 µL (3.0 eq.) TEA, and 32 mg 6-[(2-methylpropyl)amino]pyridine-2-carboxylic acid (crude batch) were added and the mixture was stirred at rt overnight. 10 mL water were added and aqueous workup using water and ethyl acetate led to a solid that was stirred with diethylether, filtered, and dried affording 7 mg of the title compound.

LC-MS (method A): Rt = 1.12 min; MS (ESIpos): m/z = 467 $[M+H]^+$

^{1}H NMR (DMSO-d_6, 300 MHz) δ 1.00 (d, 6H, *J*=6.97 Hz), 1.84–2.02 (m, 1H), 3.25 (t, 2H, *J*=6.22 Hz), 3.47 (d, 2H, *J*=4.90 Hz), 3.52–3.72 (m, 6H), 3.99 (s, 3H), 5.38 (s, 2H), 6.75 (d, 1H, *J*=8.29 Hz), 6.98–7.13 (m, 2H), 7.26 (d, 1H, *J*=6.78 Hz), 7.57 (dd, 1H, *J*=8.29, 7.35 Hz), 8.19 (s, 1H), 8.70 (s, 1H), 10.75 (s, 1H).

Synthesis of compound 28

<u>Compound 28 (step 28-a)</u>

N-{6-Methoxy-2-[2-(morpholin-4-yl)-2-oxoethyl]-2H-indazol-5-yl}-6-(morpholin-4-yl)pyridine-2-carboxamide

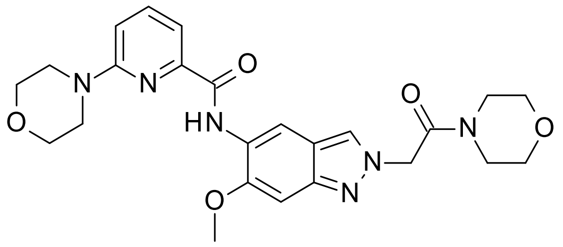

40 mg (0.17 mmol) 2-(5-amino-6-methoxy-2H-indazol-2-yl)-1-(morpholin-4-yl)ethanone (intermediate 23-e) and 43 mg (1.2 eq.) (6-(morpholin-4-yl)pyridine-2-carboxylic acid were transformed into 40 mg (83% yield) of the title compound following the same protocol used in the synthesis of compound **27**.

LC-MS (method A): Rt = 0.96 min; MS (ESIpos): m/z = 481 $[M+H]^+$

^{1}H NMR (DMSO-d_6, 400 MHz) δ 3.42–3.50 (m, 2H), 3.53–3.68 (m, 10H), 3.75–3.84 (m, 4H), 3.97 (s, 3H), 5.38 (s, 2H), 7.09 (s, 1H), 7.15 (d, 1H, *J*=8.3 Hz), 7.46 (d, 1H, *J*=7.1 Hz), 7.81 (dd, 1H, *J*=8.6, 7.3 Hz), 8.17–8.21 (m, 1H), 8.66 (s, 1H), 10.79 (s, 1H).

Synthesis of compound 29

<u>Intermediate 29-a</u>

N-(6-Methoxy-1H-indazol-5-yl)-2-(tetrahydro-2H-pyran-4-yl)-1,3-oxazole-4-carboxamide

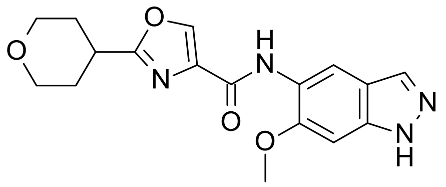

782 mg (4.80 mmol) of 6-methoxy-1H-indazole-5-amine (CAS Number 749223-61-8) and 1.04 g (5.27 mmol) of 2-(tetrahydro-2H-pyran-4-yl)-1,3-oxazole-4-carboxylic acid (CAS number 955401-82-8) were dissolved in 15 mL of THF and stirred with 734 mg (4.80 mmol) of HOBt, 1.84 g (9.59 mmol) of EDC, and 3.34 mL (24.0 mmol) of TEA at 25 °C for 26 h. Water was added, and the reaction mixture was concentrated. The resulting precipitate was filtered off with suction, washed three times with water and three times with diethyl ether, and dried in a drying cabinet. This gave 1.19 g (37% of theory) of the title compound.
MS (ESIpos): m/z = 343 $(M+H)^+$

<u>**Intermediate 29-b**</u>

***tert*-Butyl [6-methoxy-5-({[2-(tetrahydro-2H-pyran-4-yl)-1,3-oxazol-4-yl]carbonyl}amino)-2H-indazol-2-yl]acetate**

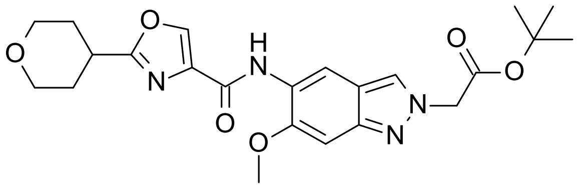

1.19 g (1.77 mmol) of *N*-(6-methoxy-1H-indazol-5-yl)-2-(tetrahydro-2H-pyran-4-yl)-1,3-oxazole-4-carboxamide were stirred with 524 µL (3.55 mmol) of *tert*-butyl bromoacetate in 10 mL of tetrahydrofuran in the presence of 752 µL (3.55 mmol) of *N*,*N*-dicyclohexylmethylamine at 70 °C for 2.5 h and at 60 °C for 17 h. 1.51 mL (9.5 mmol) of *tert*-butyl bromoacetate and 2.00 mL (9.5 mmol) of *N*,*N*-dicyclohexylmethylamine were added and the mixture was stirred at 70 °C for 6 h. The solid was filtered off with suction and washed three times with ethyl acetate. Water was added to the filtrate, and, after phase separation, the aqueous phase was washed once more with ethyl acetate. The combined organic phases were washed with saturated sodium chloride solution, filtered through a hydrophobic filter, and concentrated. Ethyl acetate was added to the crude product and the solid was filtered off with suction, washed three times with ethyl acetate, and dried. This gave a total of 330 mg (41% yield) of the title compound.
MS (ESIpos): m/z = 457 $(M+H)^+$

^{1}H NMR (DMSO-d_6, 500 MHz) δ 1.44 (s, 9H), 1.72–1.86 (m, 2H), 1.91–2.02 (m, 2H), 3.17–3.27 (m, 1H), 3.48 (td, 2H, *J*=2.2, 11.4 Hz), 3.92 (dt, 2H, *J*=3.7, 11.1 Hz), 3.97 (s, 3H), 5.18 (s, 2H), 7.10 (s, 1H), 8.26 (d, 1H), 8.57 (s, 1H), 8.74 (s, 1H), 9.41 (s, 1H).

Intermediate 29-c

6-Methoxy-5-({[2-(tetrahydro-2H-pyran-4-yl)-1,3-oxazol-4-yl]carbonyl}amino)-2H-indazol-2-yl]acetic acid

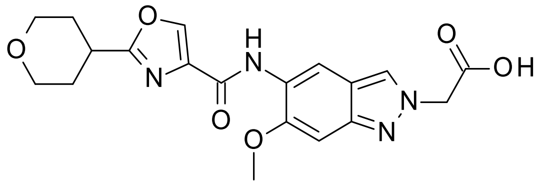

325 mg (0.71 mmol) of *tert*-butyl [6-methoxy-5-({[2-(tetrahydro-2H-pyran-4-yl)-1,3-oxazol-4-yl]carbonyl}amino)-2H-indazol-2-yl]acetate were dissolved in 5 mL of DCM and stirred with 549 µL (7.12 mmol) of trifluoroacetic acid at 25 °C for 21 h. 275 µL (3.56 mmol) of TFA were added and the mixture was stirred at 25 °C for 70 h. Water was added, the resulting precipitate was filtered off with suction, washed three times with water and three times with diethyl ether, and the solid was dried under reduced pressure. This gave 313 mg of the title compound (crude product, contained water).

MS (ESIpos): m/z = 401 $(M+H)^+$

^{1}H NMR (DMSO-d_6, 300 MHz) δ 1.67–1.90 (m, 2H), 1.9–2.0 (m, 2H), 3.2–3.3 (m, 1H), 3.40–3.54 (m, 2H), 3.87–4.01 (m, 6H), 5.20 (s, 2H), 7.10 (s, 1H), 8.27 (s, 1H), 8.56 (s, 1H), 8.75 (s, 1H), 9.42 (s, 1H).

Compound 29 (step 29-d)

N-{6-Methoxy-2-[2-(morpholin-4-yl)-2-oxoethyl]-2H-indazol-5-yl}-2-(tetrahydro-2H-pyran-4-yl)-1,3-oxazole-4-carboxamide

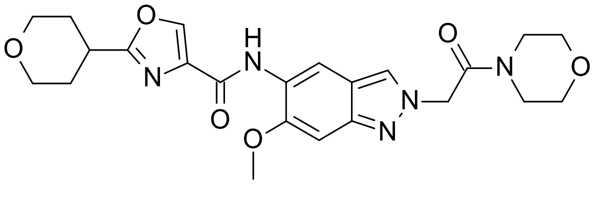

100 mg (0.25 mmol) 6-methoxy-5-({[2-(tetrahydro-2H-pyran-4-yl)-1,3-oxazol-4-yl]carbonyl}amino)-2H-indazol-2-yl]acetic acid and 44 mg (2.0 eq.) morpholine were

transformed into 65 mg (55% yield) of the title compound following the synthesis protocol of compound **27**.

LC-MS (method A): Rt = 0.88 min; MS (ESIpos): m/z = 470 $[M+H]^+$

^{1}H NMR (DMSO-d_6, 400 MHz) δ 1.71–1.84 (m, 2H), 1.92–2.02 (m, 2H), 3.16–3.28 (m, 1H), 3.4–3.7 (m, 10H), 3.88–3.95 (m, 2H), 3.97 (s, 3H), 5.38 (s, 2H), 7.08 (s, 1H), 8.20 (s, 1H), 8.56 (s, 1H), 8.74 (s, 1H), 9.41 (s, 1H).

Synthesis of compound 30

<u>Compound 30 (step 30-a)</u>

N-[6-Methoxy-2-(3-methoxypropyl)-2H-indazol-5-yl]-6-(trifluoromethyl)pyridine-2-carboxamide

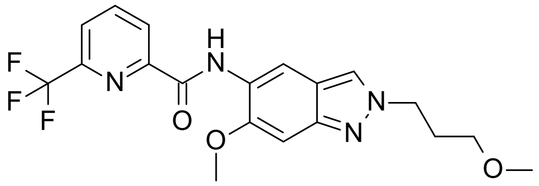

300 mg (0.60 mmol) *N*-(6-methoxy-1H-indazol-5-yl)-6-(trifluoromethyl)pyridine-2-carboxamide (intermediate 14-a) were dissolved in 4 mL DMF and 121 µL (1.07 mmol) 1-bromo-3-methoxypropane. 370 mg (2.68 mmol) potassium carbonate, and 178 mg (1.07 mmol) potassium iodide were added with stirring. After stirring for 17 h at 100 °C, the mixture was diluted with water and extracted with ethyl acetate. The combined organic layers were washed with brine, filtered with a water-repellent filter, and concentrated. The residue was purified by preparative HPLC and freeze-dried, affording 81 mg (22% yield) of the title compound.

LC-MS (method A): Rt = 1.27 min; MS (ESIpos): m/z = 409 $[M+H]^+$

^{1}H NMR (DMSO-d_6, 500 MHz) δ 2.13 (quin, 2H, *J*=6.6 Hz), 3.24 (s, 3H), 3.99 (s, 3H), 4.40 (t, 2H, *J*=7.0 Hz), 7.16 (s, 1H), 8.21 (dd, 1H, *J*=1.0, 7.6 Hz), 8.3-8.3 (m, 1H), 8.40 (t, 1H, *J*=7.8 Hz), 8.47 (d, 1H, *J*=7.6 Hz), 8.69 (s, 1H), 10.50 (s, 1H).

Synthesis of compound 31

<u>Compound 31 (step 31-a)</u>

N-[2-(3-Hydroxy-3-methylbutyl)-6-methoxy-2H-indazol-5-yl]-6-(trifluormethyl)pyridin-2-carboxamid

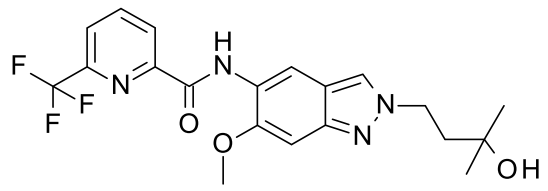

150 mg (446 µmol) *N*-(6-methoxy-1H-indazol-5-yl)-6-(trifluoromethyl)pyridine-2-carboxamide (intermediate 14-a) were dissolved in 4 mL DMF and 112 mg (669 µmol) 4-bromo-2-methylbutan-2-ol, 185 mg (1.34 mmol) potassium carbonate, and 111 mg (669 µmol) potassium iodide were added with stirring. After stirring of the suspension for 5.5 h at 120 °C, water was added, and the reaction mixture was extracted with ethyl acetate three times. The combined organic layers were washed with brine, filtered with a water-repellent filter and concentrated. The residue was dissolved in 2 mL DMSO, purified with preparative HPLC, and then freeze-dried, affording 41.4 mg (22% yield) of the title compound.
LC-MS (method A): Rt = 1.18 min; MS (ESIpos): m/z = 423 $[M+H]^+$
^{1}H NMR (DMSO-d_6, 400 MHz) δ 1.16 (s, 6H), 1.97–2.08 (m, 2H), 3.99 (s, 3H), 4.39–4.48 (m, 2H), 4.52 (s, 1H), 7.15 (s, 1H), 8.22 (dd, 1H, *J*=1.0, 7.6 Hz), 8.33 (s, 1H), 8.37–8.51 (m, 2H), 8.69 (s, 1H), 10.50 (s, 1H).

Synthesis of compound 32

<u>Intermediate 32-a</u>

5-Nitro-6-[(3*S*)-tetrahydrofuran-3-yloxy]-1H-indazole

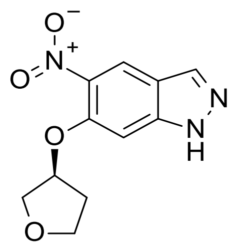

2.00 g (11.2 mmol) 5-nitro-1H-indazol-6-ol, 940 µL (12 mmol) (3*S*)-tetrahydrofuran-3-ol and 4.39 g (16.7 mmol) triphenylphosphine in 25 mL THF were treated with 3.2 mL (17 mmol) diisopropylazodicarboxylate dropwise and the mixture was stirred at 25 °C for 67 h. The

mixture was concentrated and purified using flash chromatography (Biotage SNAP cartridge (100 g; KP-Sil), eluent: hexane-ethyl acetate), affording 3.08 g of the title compound (crude batch).
MS (ESIpos): m/z = 250 $[M+H]^+$

Intermediate 32-b

2-Methyl-4-{5-nitro-6-[(3*S*)-tetrahydrofuran-3-yloxy]-2H-indazol-2-yl}butan-2-ol

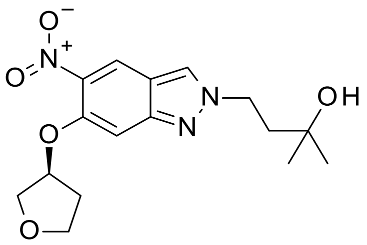

3.08 g of 5-nitro-6-[(3*S*)-tetrahydrofuran-3-yloxy]-1H-indazole (crude batch) dissolved in 50 mL of DMF were treated with 3.84 g (27.8 mmol) of potassium carbonate, after which 4.55 g of (crude batch) 3-hydroxy-3-methylbutyl-4-methylbenzolsulfonate were added. After stirring for 22 h at 80 °C, the mixture was diluted with water and extracted with ethyl acetate three times. The combined organic layers were washed with brine, filtered using a water-repellent filter, and evaporated. The residue was purified with flash-chromatography (Biotage SNAP cartridge, 100 g; KP-Sil, eluent hexane-ethyl acetate) affording 618 mg of the title compound (crude batch).

Intermediate 32-c

4-{5-Amino-6-[(3*S*)-tetrahydrofuran-3-yloxy]-2H-indazol-2-yl}-2-methylbutan-2-ol

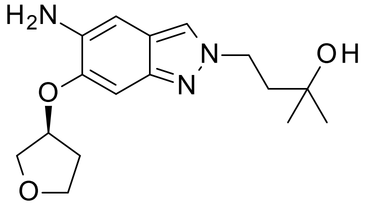

610 mg 2-methyl-4-{5-nitro-6-[(3*S*)-tetrahydrofuran-3-yloxy]-2H-indazol-2-yl}butan-2-ol dissolved in 10 mL ethanol and 3 mL water. Then, 843 mg (15.1 mmol) iron powder and 40 mg ammonium chloride were added and the mixture was stirred for 5 h at 90 °C. The mixture was cooled and filtered with Celite®. The filtrate was evaporated and the residue was treated

with THF. The mixture was then filtered and the filtrate was evaporated. The residue was dissolved in 2 mL DMF and purified by preparative HPLC affording 348 mg of a crude product. MS (ESIpos): m/z = 305 $[M+H]^+$.

Compound 32 (step 32-d)

N-{2-(3-Hydroxy-3-methylbutyl)-6-[(3S)-tetrahydrofuran-3-yloxy]-2H-indazol-5-yl}-6-(trifluoromethyl)pyridine-2-carboxamide

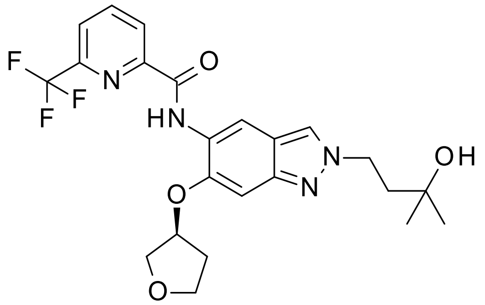

116 mg (379 µmol) 4-{5-amino-6-[(3S)-tetrahydrofuran-3-yloxy]-2H-indazol-2-yl}-2-methylbutan-2-ol, 87.0 mg (455 µmol) 6-(trifluoromethyl)pyridine-2-carboxylic acid, 173 mg (455 µmol) HATU, and 79 µL (460 µmol) DIPEA were dissolved in 2 mL THF. After stirring for 16.5 h at 25 °C, the mixture was diluted with water and extracted with ethyl acetate three times. The combined organic layers were washed with brine, filtered with a water-repellent filter, and concentrated. The residue was dissolved in 2 mL DMF, purified using preparative HPLC and freeze-dried, affording 105 mg of the title compound.
LC-MS (method C): Rt = 1.15 min; MS (ESIpos): m/z = 479 $[M+H]^+$
^{1}H NMR (DMSO-d_6, 400 MHz) δ 1.16 (s, 6H), 1.97–2.06 (m, 2H), 2.10–2.21 (m, 1H), 2.31–2.43 (m, 1H), 3.83–3.98 (m, 3H), 4.06 (dd, 1H, *J*=4.6, 10.4 Hz), 4.39–4.46 (m, 2H), 4.54 (s, 1H), 7.12 (s, 1H), 8.22 (dd, 1H, *J*=1.3, 7.6 Hz), 8.33 (s, 1H), 8.38–8.49 (m, 2H), 8.73 (s, 1H), 10.63 (s, 1H).

Synthesis of compound 33

Intermediate 33-a

Methyl 2-(3-methoxypropyl)-5-({[6-(trifluoromethyl)pyridin-2-yl]carbonyl}amino)-2H-indazole-6-carboxylate

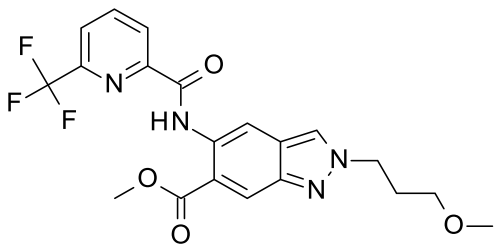

1.00 g (2.75 mmol) methyl 5-({[6-(trifluoromethyl)pyridin-2-yl]carbonyl}amino)-1H-indazole-6-carboxylate **V** (synthesis described in main manuscript) was dissolved in 5 mL of DMF, and 460 µL (4.12 mmol) of 1-bromo-3-methoxypropane, 1.14 g (8.23 mmol) of potassium carbonate, and 228 mg (1.37 mmol) of potassium iodide were added while stirring. The reaction mixture was stirred at 25 °C for 72 h, diluted with water, and extracted twice with ethyl acetate. The combined organic phases were filtered through a hydrophobic filter and concentrated. The residue was purified using column chromatography on silica gel (hexane/ethyl acetate). 28 mg (2% yield) of a pure batch of the title compound was obtained.
MS (ESIpos): m/z = 437 $(M+H)^+$
^{1}H NMR (DMSO-d_6, 400 MHz) δ 2.17 (quin, 2H, *J*=6.6 Hz), 3.24 (s, 3H), 3.33–3.36 (m, 2H), 3.96 (s, 3H), 4.53 (t, 2H, J=7.0 Hz), 8.21 (dd, 1H, J=7.7, 0.9 Hz), 8.35–8.42 (m, 1H), 8.45–8.49 (m, 2H), 8.54 (d, 1H, *J*=1.0 Hz), 9.06 (s, 1H), 12.54 (s, 1H).

Compound 33 (step 33-b)

N-[6-(2-Hydroxypropan-2-yl)-2-(3-methoxypropyl)-2H-indazol-5-yl]-6-(trifluoromethyl)pyridine-2-carboxamide

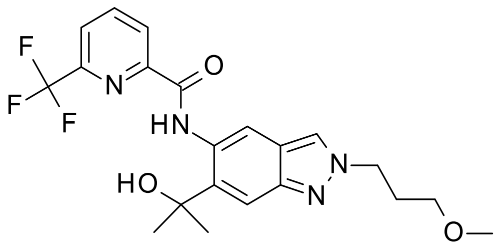

75 mg (0.17 mmol) methyl 2-(3-methoxypropyl)-5-({[6-(trifluoromethyl)pyridin-2-yl]carbonyl}amino)-2H-indazole-6-carboxylate were dissolved in 500 µL of THF and admixed with 859 µL (0.86 mmol) of a 1 M methylmagnesium bromide solution in THF. The reaction mixture was stirred at 25 °C for 60 min. Subsequently, 1 mL of a sat. ammonium chloride solution was added cautiously, and the mixture was filtered. The aqueous phase was extracted twice with ethyl acetate, and the organic phases were combined, filtered through a

hydrophobic filter, and concentrated. The residue was dissolved in 3 mL of DMSO and purified using preparative HPLC. The product-containing fractions were freeze-dried. 25 mg (32% yield) of the title compound were obtained.

LC-MS (method B): Rt = 1.14 min; MS (ESIpos): m/z = 437 $[M+H]^+$

^{1}H NMR (DMSO-d_6, 400 MHz) δ 1.62 (s, 6H), 2.14 (quin, 2H, *J*=6.6 Hz), 3.23 (s, 3H), 3.26–3.32 (m, 2H), 4.44 (t, 2H, *J*=7.0 Hz), 5.95 (s, 1H), 7.58 (s, 1H), 8.16 (d, 1H, *J*=7.9 Hz), 8.31–8.40 (m, 2H), 8.43–8.48 (m, 1H), 8.72 (s, 1H), 12.36 (s, 1H).

Synthesis of compound 34

<u>Compound 34 (step 34-a)</u>

N-[2-(1,1-Dioxothian-3-yl)-6-methoxy-indazol-5-yl]-6-(trifluoromethyl)pyridine-2-carboxamide

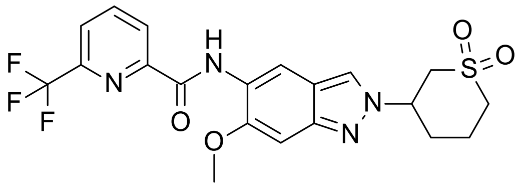

N-(6-methoxy-1H-indazol-5-yl)-6-(trifluoromethyl)pyridine-2-carboxamide (intermediate 14-a, 200 mg, 595 µmol) and 4-bromo-2H-tetrahydro-thiopyran 1,1-dioxide (190 mg, 892 µmol) were dissolved in DMF (4.0 ml, 52 mmol) and potassium carbonate (247 mg, 1.78 mmol) was added. The reaction mixture was stirred for 20 h at 100 °C. Afterwards, the reaction mixture was diluted with water and extracted three times with ethyl acetate. The combined organic extracts were washed with brine, filtered over a water-free filter, and concentrated *in vacuo.* The residue was diluted with 5.5 mL DMSO / acetonitrile and purified using preparative HPLC to afford 70.2 mg (23% yield, 90% NMR purity) of the title compound.

LC-MS (method A): Rt = 1.27 min; MS (ESIpos): m/z = 469 $[M+H]^+$

^{1}H NMR (DMSO-d_6, 500 MHz) δ 1.89–2.03 (m, 1H), 2.15–2.23 (m, 3H), 2.52 (d, 1H, *J*=1.91 Hz), 3.15–3.20 (m, 1H), 3.26–3.30 (m, 1H), 3.63 (dt, 1H, *J*=13.35, 3.50 Hz,), 3.76–3.83 (m, 1H), 3.99 (s, 3H), 4.89–5.00 (m, 1H), 7.16 (s, 1H), 8.21 (dd, 1H, *J*=7.95, 0.95 Hz), 8.38–8.41 (m, 1H), 8.42 (d, 1H, *J*=0.95 Hz), 8.45–8.48 (m, 1H), 8.70 (s, 1H).

Synthesis of compound 35

Intermediate 35-a

5-fluoro-2,4-dinitroaniline

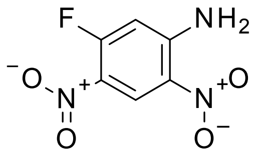

1,5-difluoro-2,4-dinitrobenzene (9.58 g, 46.9 mmol) was dissolved in THF (48 ml, 590 mmol) and ammonium hydroxide (3.7 ml, 94 mmol) was added. The reaction mixture was stirred for 72 h at 25 °C. Additional ammonium hydroxide (1.8 ml, 47 mmol) was added and the mixture was stirred for 1 h at 25 °C. Afterwards, the reaction mixture was partly concentrated *in vacuo* and diluted with water. After stirring for 5 minutes, the formed suspension was filtered, washed with hexane, and dried to afford 8.85 g (94% yield) of the title compound.
LC-MS (method B): Rt = 0.89 min; MS (ESIneg): m/z = 200 $[M-H]^-$
^{1}H NMR (DMSO-d_6, 400 MHz) δ 6.89 (d, 1H, *J*=13.94 Hz), 8.22-8.66 (m, 2H), 8.82 (d, 1H, *J*=8.11 Hz).

Intermediate 35-b

5-Methoxy-2,4-dinitroaniline

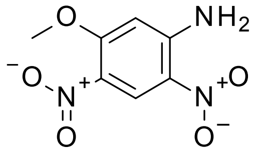

5-Fluoro-2,4-dinitroaniline (8.94 g, 44.5 mmol) was dissolved in THF (89 ml, 1.1 mol) and methanol (89 ml, 2.2 mol) and sodium hydroxide solution (44 ml, 1.0 M, 44 mmol) was added. The reaction mixture was stirred for 30 minutes at 25 °C and concentrated *in vacuo.* The residue was triturated with water and the resulting suspension was filtrated, washed with hexane, and dried to afford 9.17 g (97% yield) of the title compound.
LC-MS (method B): Rt = 0.89 min; MS (ESIpos): m/z = 214 $[M+H]^+$
^{1}H NMR (DMSO-d_6, 400 MHz) δ 3.91 (s, 3H), 6.65 (s, 1H), 8.14 (br s, 2H), 8.73 (s, 1H).

Intermediate 35-c

4-Methoxy-5-nitrobenzene-1,2-diamine

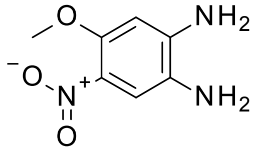

Sodium sulfide nonahydrate (25.6 g, 106 mmol) was dissolved in water (71 ml) and sodium hydrogencarbonate (8.49 g, 101 mmol) was slowly added. After 10 minutes of stirring, methanol (71 ml, 1.8 mol) was added. The formed suspension was stirred at 0 °C for additional 10 minutes before filtration. The filter cake was washed with additional methanol (23 ml, 560 mmol) and the combined filtrate was slowly added to a preformed solution of 5-methoxy-2,4-dinitroaniline (8.07 g, 37.9 mmol) in THF (19 ml, 230 mmol) and methanol (38 ml, 940 mmol). The resulting reaction mixture was stirred for 1 h at 70 °C and then cooled to 0 °C. After stirring for 20 minutes, the formed solid was filtered off, washed with ethyl acetate, and dried, affording an initial yield of the crude product. The filtrate was concentrated *in vacuo* and triturated with a 1:1 mixture of ethyl acetate and water. The resulting suspension was filtered, washed with ethyl acetate, and dried to afford a second portion of the crude product. The combined product fractions were purified by Biotage Isolera™ chromatography (SNAP KP-Sil – 340 g, eluting with hexane-ethyl acetate, 1:0 to 1:9) to afford 2.85 g (16% yield) of the title compound.

LC-MS (method B): Rt = 0.54 min; MS (ESIpos): m/z = 184 $[M+H]^+$

^1H NMR (DMSO-d_6, 400 MHz) δ 3.74 (s, 3H), 4.61 (br s, 2H), 6.12 (s, 2H), 6.29 (s, 1H), 7.28 (s, 1H).

__Intermediate 35-d__

N-(2-Amino-5-methoxy-4-nitro-phenyl)-1,1-dioxo-thiane-3-carboxamide

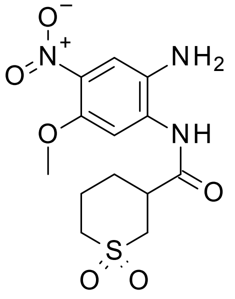

4-Methoxy-5-nitrobenzene-1,2-diamine (2.75 g, 15.0 mmol) and 1,1-dioxo-1lambda6-thiane-3-carboxylic acid (2.67 g, 15.0 mmol) were dissolved in THF (93 ml, 1.2 mol) and TEA (3.1

ml, 22 mmol) and HATU (8.55 g, 22.5 mmol) was added. The reaction mixture was stirred for 24 h at 25 °C. The reaction mixture was concentrated *in vacuo* and triturated with water. The resulting suspension was collected by filtration and washed with water. The filter cake was taken up in a 2:1 mixture of ethyl acetate and water and basified to pH 9 with sat. sodium bicarbonate solution. The precipitate obtained was collected by filtration and washed with water and ethyl acetate. The resulting filter cake was again taken up in sat. sodium bicarbonate solution and diluted with water. The precipitate obtained was collected by filtration, washed with water, and dried to afford 4.14 g (81% yield) of the title compound.
LC-MS (method B): Rt = 0.62 min; MS (ESIpos): m/z = 344 $[M+H]^+$
^1H NMR (DMSO-d_6, 400 MHz) δ 1.62 (qd, 1H, *J*=12.84, 3.30 Hz), 1.80–1.88 (m, 1H), 2.02 (br dd, 1H, *J*=13.31, 2.41 Hz), 2.08–2.17 (m, 1H), 2.98–3.10 (m, 2H), 3.13–3.24 (m, 3H), 3.82 (s, 3H), 6.39 (s, 1H), 6.46 (br s, 2H), 7.92 (s, 1H), 9.30 (br s, 1H).

<u>Intermediate 35-e</u>

3-(5-Methoxy-6-nitro-1H-benzimidazol-2-yl)thiane 1,1-dioxide

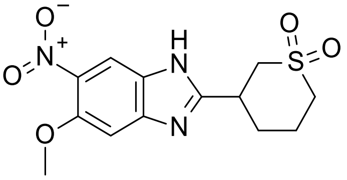

N-(2-Amino-5-methoxy-4-nitro-phenyl)-1,1-dioxo-thiane-3-carboxamide (4.14 g, 12.1 mmol) was dissolved in acetic acid (31 ml) and stirred for 24 h at 90 °C. Additional acetic acid (51 ml) was added and the mixture was stirred at 90 °C for a further 24 h. The reaction mixture was then concentrated *in vacuo.* Toluene was added and *in vacuo* concentration continued. After two repetitions, the product was dried to afford 4.21 g (crude) of the title compound.
LC-MS (method B): Rt = 0.64 min; MS (ESIpos): m/z = 326 $[M+H]^+$
^1H NMR (DMSO-d_6, 400 MHz) δ 1.75–2.01 (m, 2H), 2.12–2.22 (m, 2H), 3.10–3.27 (m, 3H), 3.45–3.51 (m, 2H), 3.92 (s, 3H), 7.33 (s, 1H), 8.10 (s, 1H).

<u>Intermediate 35-f</u>

2-(1,1-Dioxothian-3-yl)-6-methoxy-3H-benzimidazol-5-amine

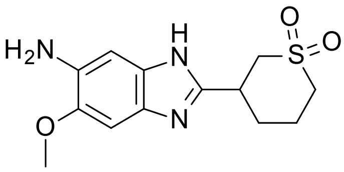

3-(5-Methoxy-6-nitro-1H-benzimidazol-2-yl)thiane 1,1-dioxide (4.16 g, 12.8 mmol) was dissolved in ethanol (87 ml, 1.5 mol) and palladium on charcoal (2.82 g, 10 % Pd, 3.20 mmol) and ammonium formate (4.03 g, 100 % purity, 63.9 mmol) were added. The reaction mixture was stirred for 30 minutes at 60 °C. Afterwards, palladium on charcoal was removed by filtration and washed with ethyl acetate. The filtrate was concentrated *in vacuo* and the residue was partitioned using ethyl acetate and water. After phase separation and a second extraction with ethyl acetate, the combined organic layers were washed with brine, dried over anhydrous magnesium sulfate, filtered, and concentrated *in vacuo.* The aqueous layer was brought to pH 9 using sat. sodium bicarbonate solution and extracted with ethyl acetate. After drying over anhydrous magnesium sulfate and filtration, the filtrate was concentrated *in vacuo.* The combined crude product fractions were triturated with ethyl acetate in an ultrasonic bath for 20 minutes. After filtration, the product was triturated with acetonitrile in an ultrasonic bath. The product was again collected by filtration and dried to afford 1.54 g (41% yield) of the title compound.

LC-MS (method B): Rt = 0.60 min; MS (ESIpos): m/z = 296 $[M+H]^+$

^1H NMR (DMSO-d_6, 400 MHz) δ 1.68–1.81 (m, 1H), 1.89–1.98 (m, 1H), 2.10–2.18 (m, 2H), 3.06–3.13 (m, 1H), 3.14–3.24 (m, 1H), 3.36–3.43 (m, 3H), 3.76 (s, 3H), 4.58 (br s, 2H), 6.66 (s, 1H), 6.96 (s, 1H), 11.66 (s, 1H).

<u>Compound 35 (step 35-g)</u>

N-[2-(1,1-Dioxothian-3-yl)-6-methoxy-3H-benzimidazol-5-yl]-6-(trifluoromethyl)pyridine-2-carboxamide

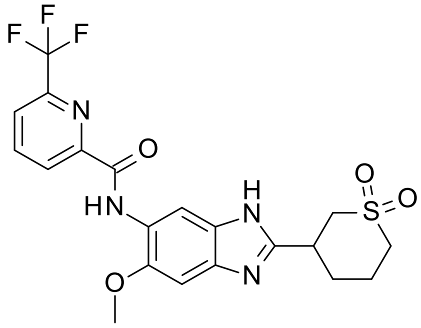

2-(1,1-Dioxothian-3-yl)-6-methoxy-3H-benzimidazol-5-amine (1.27 g, batch contained impurities), 6-(trifluoromethyl)pyridine-2-carboxylic acid (411 mg, 2.15 mmol), and HATU (1.23 g, 3.22 mmol) were dissolved in DMF (9.5 ml, 260 mmol) and TEA (450 µL, 3.2 mmol) was added. The reaction mixture was stirred for 24 h at 25 °C and then diluted with water. The precipitate obtained was collected by filtration, washed with water, and dried. The crude product was dissolved in acetonitrile and concentrated *in vacuo.* The residue was diluted with DMSO and purified using preparative HPLC. The product fractions were pooled and concentrated *in vacuo* to afford 518 mg of the title compound.

LC-MS (method B): Rt = 1.05 min; MS (ESIpos): m/z = 469 $[M+H]^+$

^1H NMR (DMSO-d_6, 400 MHz) δ 1.11 (s, 3H), 1.74–1.89 (m, 1H), 1.89–2.03 (m, 1H), 2.11–2.24 (m, 2H), 3.08–3.16 (m, 1H), 3.18–3.28 (m, 1H), 3.41–3.53 (m, 3H), 3.91 – 4.00 (m, 3H), 7.16 (s, 0.43H) +7.33 (s, 0.57H), 8.22 (d, J=7.86 Hz, 1H), 8.36–8.43 (m, 1H), 8.44–8.49 (m, 1H), 8.53–8.63 (m, 1H), 10.41 (s, 0.45H), 10.51 (0.6H), 12.28 (br s), rotamers present.

Synthesis of compound 36

Intermediate 36-a

5-Chloro-2-methoxy-4-nitroaniline

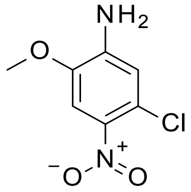

2-Amino-4-chloro-5-nitrophenol (51.3 g, 272 mmol) and iodomethane (17 ml, 270 mmol; CAS Number 74-88-4) were dissolved in DMF (990 ml) and potassium carbonate (56.4 g, 408 mmol) was added. The reaction mixture was stirred for 1 h at 25 °C and subsequently concentrated *in vacuo.* The residue was suspended in water and stirred for 30 minutes. After filtration and washing with water, the crude product was dissolved in a 10:7 mixture of ethyl acetate and water, dried over anhydrous magnesium sulfate, filtered, and concentrated *in vacuo* to afford 49.4 g (90% yield) of the title compound.

LC-MS (method B): Rt = 0.98 min; MS (ESIpos): m/z = 203 $[M+H]^+$

^1H NMR (DMSO-d_6, 400 MHz) δ 3.87 (s, 3H), 6.53 (s, 2H), 6.73 (s, 1H), 7.57 (s, 1H).

Intermediate 36-b

***tert*-Butyl (5-chloro-2-methoxy-4-nitrophenyl)carbamate**

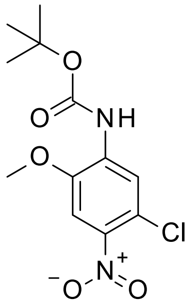

5-Chloro-2-methoxy-4-nitroaniline (49.4 g, 244 mmol), di-*tert*-butyl dicarbonate (63.9 g, 293 mmol), and 4-dimethylaminopyridine (238 mg, 1.95 mmol) were dissolved in DCM (490 ml). The reaction mixture was stirred for 4 h at 45 °C and for 20 h at 25 °C and concentrated *in vacuo.* The crude product (81.2 g) was directly used in the next step.
LC-MS (method A): Rt = 1.45 min; MS (ESIneg): m/z = 301 $[M-H]^-$
^1H NMR (DMSO-d_6, 400 MHz) δ 1.48 (s, 9H), 3.90 (s, 3H), 7.73 (s, 1H), 8.14 (s, 1H), 8.78 (br s, 1H).

Intermediate 36-c

***tert*-Butyl [2-methoxy-5-(methylamino)-4-nitrophenyl]carbamate**

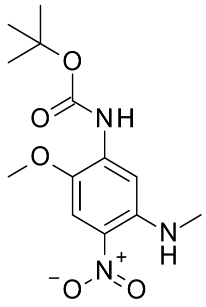

tert-Butyl (5-chloro-2-methoxy-4-nitrophenyl)carbamate (15.9 g, 52.5 mmol) was dissolved in methanamine in ethanol (160 ml, 1.8 mol) and the reaction mixture was stirred for 24 h at 70 °C. After concentration *in vacuo* the residue was purified using Biotage Isolera™ chromatography (SNAP KP-Sil – 1500 g, eluting with DCM) to afford 8.40 g (54% yield) of the title compound.
LC-MS (method B): Rt = 1.34 min; MS (ESIpos): m/z = 298 $[M+H]^+$

^1H NMR (DMSO-d_6, 400 MHz) δ 1.49 (s, 9H), 2.94 (d, 3H, *J*=4.82 Hz), 3.81 (s, 3H), 7.49 (s, 1H), 7.61 (s, 1H), 8.35 (s, 1H), 8.36–8.40 (m, 1H).

Intermediate 36-d

***tert*-Butyl [4-amino-2-methoxy-5-(methylamino)phenyl]carbamate**

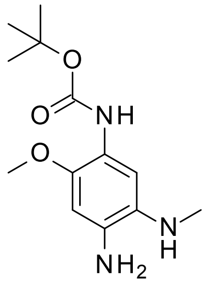

Palladium on charcoal (14.8 g, 10 % Pd content, 16.8 mmol; CAS Number 123-91-1) was flushed with argon in a three-neck flask equipped with a mechanical stirrer. Ethanol (400 ml, 6.9 mol), *tert*-butyl [2-methoxy-5-(methylamino)-4-nitrophenyl]carbamate (20.0 g, 67.3 mmol), and ammonium formiate (21.2 g, 336 mmol) were added and the reaction mixture was stirred for 2 h at 60 °C. After filtration and concentration *in vacuo*, the residue was diluted with water and extracted with ethyl acetate two times. The combined organic extracts were washed with water and brine, dried over anhydrous magnesium sulfate, filtered, and concentrated *in vacuo* to afford 16.23 g (90% yield) of the title compound.
MS (ESIpos): m/z = 268 $[M+H]^+$.
^1H NMR (DMSO-d_6, 400 MHz) δ 1.42 (s, 9H), 2.63 (s, 3H), 3.62 (s, 3H), 4.22 (br s, 1H), 4.39 (br d, 2H, *J*=3.55 Hz), 6.32 (s, 1H), 6.63 (br s, 1H), 7.46 (s, 1H)

Intermediate 36-e

***tert*-Butyl *N*-[4-[(1,1-dioxothiane-3-carbonyl)amino]-2-methoxy-5-(methylamino)phenyl]carbamate**

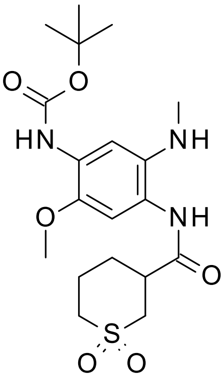

tert-Butyl [4-amino-2-methoxy-5-(methylamino)phenyl]carbamate (34.1 g, 80 % purity, 102 mmol) and tetrahydro-2H-thiopyran-3-carboxylic acid 1,1-dioxide (18.2 g, 102 mmol) were dissolved in DMF (650 ml) and TEA (21 ml, 150 mmol) and HATU (58.2 g, 153 mmol) was added. The reaction mixture was stirred for 1 h at 25 °C and then diluted with water. After basification to pH 8 using aqueous sodium bicarbonate solution, the resulting precipitate was collected by filtration and washed with water. The filter cake was dissolved in a 1:1 mixture of ethyl acetate and DCM, dried over anhydrous magnesium sulfate, filtered, and concentrated *in vacuo* to afford 43.9 g (98% yield) of the title compound.

MS (ESIpos): m/z = 428 $[M+H]^+$

^{1}H NMR (DMSO-d_6, 400 MHz) δ 1.45 (s, 9H), 1.62 (qd, 1H, *J*=12.84, 3.30 Hz), 1.76–1.91 (m, 1H), 1.96–2.04 (m, 1H), 2.07–2.18 (m, 1H), 2.65 (d, 3H, *J*=5.07 Hz), 2.98–3.10 (m, 2H), 3.11–3.22 (m, 2H), 3.30 (br d, 1H, *J*=3.30 Hz), 3.66 (s, 3H), 4.73 (q, 1H, *J*=4.90 Hz), 6.87 (s, 1H), 7.06 (s, 1H), 7.71 (s, 1H), 9.17 (s, 1H).

<u>Intermediate 36-f</u>

***tert*-Butyl *N*-[2-(1,1-dioxothian-3-yl)-6-methoxy-3-methyl-benzimidazol-5-yl]carbamate**

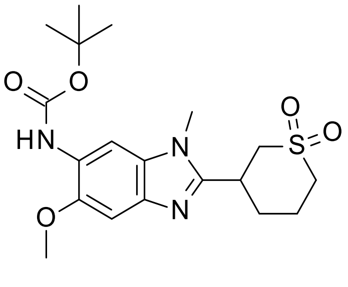

tert-Butyl *N*-[4-[(1,1-dioxothiane-3-carbonyl)amino]-2-methoxy-5-(methylamino)phenyl]carbamate (43.9 g, 103 mmol) was dissolved in acetic acid (270 ml) and stirred for 24 h at 40 °C. The reaction was concentrated *in vacuo* and toluene was added

to the residue. The resulting suspension was again concentrated *in vacuo*. Toluene was added twice and after each addition the mixture was concentrated. Then, the crude product was dried to afford 42.4 g (crude material) of the title compound.

^{1}H NMR (DMSO-d$_6$, 400 MHz) δ 1.40–1.45 (m, 1H), 1.47 (s, 9H), 1.67–1.79 (m, 1H), 1.98–2.07 (m, 1H), 2.07–2.17 (m, 1H), 3.07–3.16 (m, 1H), 3.22–3.31 (m, 1H), 3.48 (t, *J*=12.80 Hz, 1H), 3.53–3.60 (m, 1H), 3.72 (s, 3H), 3.82 (s, 3H), 7.18 (s, 1H), 7.80 (br s, 1H), 7.89 (s, 1H), 11.98 (br s, 1H).

LC-MS (method B): Rt = 1.09 min; MS (ESIpos): m/z = 410 $[M+H]^+$

Intermediate 36-g

2-(1,1-Dioxothian-3-yl)-6-methoxy-3-methyl-benzimidazol-5-amine

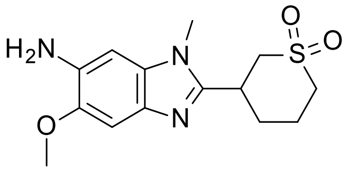

tert-Butyl *N*-[2-(1,1-dioxothian-3-yl)-6-methoxy-3-methyl-benzimidazol-5-yl]carbamate (42.4 g, 104 mmol) was dissolved in DCM (390 ml, 6.1 mol) and TFA (60 ml) was added. The reaction mixture was stirred for 1 h at 25 °C. More TFA (60 ml) was added and stirring continued for 1 h at 25 °C. The reaction mixture was poured into water and the pH was adjusted to 8 - 9 by the addition of sodium bicarbonate solution. After concentration *in vacuo* to remove the excess of DCM, ethyl acetate was added and the aqueous layer was extracted three times. The combined organic extracts were washed with water, brine, dried over anhydrous magnesium sulfate, filtered, and concentrated *in vacuo* to afford 5.0 g of a solid. While standing, the aqueous layer transformed into a suspension that was then filtered. The filter cake was dissolved in a 1:1 mixture of DCM and ethyl acetate, filtered, and the filtrate was concentrated *in vacuo* and dried to afford 18.6 g of solid. The two solids were combined to afford 23.6 g (74% yield) of the title compound.

^{1}H NMR (DMSO-d$_6$, 400 MHz) δ 1.64–1.77 (m, 1H), 1.95–2.04 (m, 2H), 2.05–2.15 (m, 1H), 3.06–3.16 (m, 1H), 3.19–3.27 (m, 1H), 3.28–3.32 (m, 1H), 3.40–3.54 (m, 2H), 3.61 (s, 3H), 3.77 (s, 3H), 4.67 (s, 2H), 6.64 (s, 1H), 6.98 (s, 1H).

LC-MS (method B): Rt = 0.63 min; MS (ESIpos): m/z = 310 $[M+H]^+$

Compound 36 (step 36-h)

N-[2-(1,1-Dioxothian-3-yl)-6-methoxy-3-methyl-benzimidazol-5-yl]-6-(trifluoromethyl)pyridine-2-carboxamide

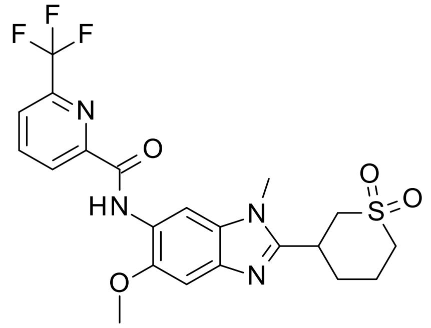

2-(1,1-Dioxothian-3-yl)-6-methoxy-3-methyl-benzimidazol-5-amine (267 mg, 604 µmol), 6-(trifluoromethyl)pyridine-2-carboxylic acid (115 mg, 604 µmol), HATU (345 mg, 906 µmol), and TEA (130 µL, 910 µmol) were dissolved in DMF (3.0 ml, 82 mmol) and the reaction mixture was stirred for 24 h at 25 °C. The reaction mixture was diluted with acetonitrile and purified by preparative HPLC to afford 165 mg (57% yield) of the title compound.
LC-MS (method B): Rt = 1.13 min; MS (ESIpos): m/z = 483 $[M+H]^+$
^{1}H NMR (DMSO-d_6, 400 MHz) δ 1.70–1.83 (m, 1H), 2.01–2.09 (m, 2H), 2.10–2.19 (m, 1H), 3.11–3.17 (m, 1H), 3.22–3.30 (m, 2H), 3.50 (t, 1H, *J*=12.93 Hz), 3.58–3.67 (m, 1H), 3.79 (s, 3H), 3.96 (s, 3H), 7.36 (s, 1H), 8.23 (dd, 1H, *J*=7.60, 1.27 Hz), 8.39–8.45 (m, 1H), 8.45–8.49 (m, 1H), 8.58 (s, 1H), 10.53 (s, 1H).

Synthesis of compound 37

<u>Intermediate 37-a</u>

1-(2-Fluoro-4-hydroxy-5-nitrophenyl)ethan-1-one

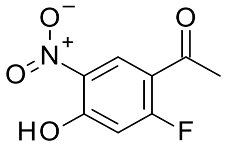

1-(2-Fluoro-4-hydroxyphenyl)ethan-1-one (10.0 g, 64.9 mmol) was dissolved in sulfuric acid (150 ml, 2.8 mol) and potassium nitrate (7.87 g, 77.9 mmol) was slowly added at -5 °C. The reaction mixture was stirred for 2 h at -5 °C. The reaction mixture was slowly poured into iced

water and the resulting precipitate obtained was collected by filtration, washed with water and hexane, and dried to afford 12.28 g (95% yield) of the title compound.

MS (ESIpos): m/z = 200 $[M+H]^+$

^{1}H NMR (DMSO-d_6, 400 MHz) δ 2.55 (d, 3H, $^5J_{HF}$=4.56 Hz), 6.98 (d, 1H, $^3J_{HF}$=12.42 Hz), 8.39 (d, 1H, $^4J_{HF}$=7.86 Hz), 12.48 (br s, 1H).

Intermediate 37-b

3-Methyl-5-nitro-1H-indazol-6-ol

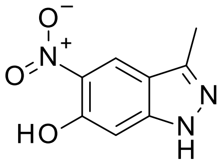

1-(2-Fluoro-4-hydroxy-5-nitrophenyl)ethan-1-one (12.3 g, 61.5 mmol) was dissolved in ethanol (120 ml, 2.1 mol) and hydrazine hydrate (15 ml, 310 mmol) was slowly added. The reaction mixture was stirred for 2 h at 100 °C. After cooling, water was added and the pH was adjusted to 3 to 4 with 1 N HCl. The precipitate obtained was collected by filtration and dried to afford 6.67 g (56% yield) of the title compound.

MS (ESIpos): m/z = 194 $[M+H]^+$

^{1}H NMR (DMSO-d_6, 400 MHz) δ 2.47 (s, 3H), 6.94 (s, 1H), 8.42 (s, 1H), 10.74 (s, 1H), 12.69 (s, 1H).

Intermediate 37-c

6-Methoxy-3-methyl-5-nitro-1H-indazole

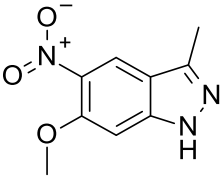

3-Methyl-5-nitro-1H-indazol-6-ol (3.90 g, 20.2 mmol) and iodomethane (1.5 ml, 24 mmol) were dissolved in DMF (75 ml) and potassium carbonate (4.19 g, 30.3 mmol) was added. The reaction mixture was stirred for 2 h at 60 °C. After concentration *in vacuo* the residue was diluted with water and extracted with ethyl acetate three times. The combined organic

extracts were washed with water, brine, dried over anhydrous magnesium sulfate, filtered, and concentrated *in vacuo* to afford 4.03 g (96% yield) of the title compound.
MS (ESIpos): m/z = 208 $[M+H]^+$.
^{1}H NMR (DMSO-d_6, 400 MHz) δ 2.48 (s, 3H), 3.94 (s, 3H), 7.11 (s, 1H), 8.40 (s, 1H), 12.95 (s, 1H).

Intermediate 37-d

6-Methoxy-3-methyl-1H-indazol-5-amine

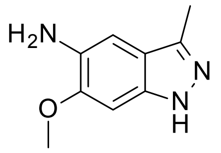

6-Methoxy-3-methyl-5-nitro-1H-indazole (2.00 g, 9.65 mmol) was dissolved in a mixture of THF (60 ml) and methanol (23 ml). Palladium on charcoal (1.03 g, 10 % purity, 1.17 mmol) was added under argon and the reaction mixture was stirred for 3 h at 25 °C in a hydrogen atmosphere (1 bar). The reaction mixture was filtered and the filtrate was concentrated *in vacuo* to afford 1.63 g (95% yield) of the title compound.
MS (ESIpos): m/z = 178 $[M+H]^+$
^{1}H NMR (DMSO-d_6, 400 MHz) δ 2.32 (s, 3H), 3.82 (s, 3H), 4.46 (br s, 2H), 6.72 (s, 1H), 6.74 (s, 1H), 11.99 (s, 1H).

Intermediate 37-e

N-(6-Methoxy-3-methyl-1H-indazol-5-yl)-6-(trifluoromethyl)pyridine-2-carboxamide

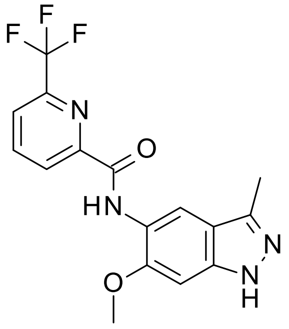

6-Methoxy-3-methyl-1H-indazol-5-amine (800 mg, 74 % purity, 3.34 mmol), 6-(trifluoromethyl)pyridine-2-carboxylic acid (638 mg, 3.34 mmol), HATU (1.91 g, 5.01 mmol), and TEA (700 µL, 5.0 mmol) were dissolved in DMF (10 ml, 270 mmol) and the reaction

mixture was stirred for 24 h at 25 °C. The reaction mixture was poured into water and stirred for 30 minutes. The precipitate that was obtained was collected by filtration and washed with water. The filter cake was dissolved in DCM, dried over anhydrous magnesium sulfate, filtered, and concentrated *in vacuo* to afford 1.31 g (crude) of the title compound.

^{1}H NMR (DMSO-d$_6$, 400 MHz) δ 2.46 (s, 3H), 4.00 (s, 3H), 7.05 (s, 1H), 8.22 (dd, 1H, *J*=7.60, 1.27 Hz), 8.38-8.44 (m, 1H), 8.44-8.47 (m, 1H), 8.65 (s, 1H), 10.41 (s, 1H), 12.51 (s, 1H).

LC-MS (method B): Rt = 1.15 min; MS (ESIpos): m/z = 351 [M+H]$^+$

<u>**Compound 37 (step 37-f)**</u>

N-[2-(3-Hydroxy-3-methylbutyl)-6-methoxy-3-methyl-2H-indazol-5-yl]-6-(trifluoromethyl)pyridine-2-carboxamide

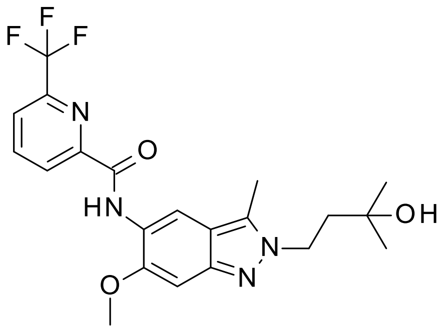

N-(6-Methoxy-3-methyl-1H-indazol-5-yl)-6-(trifluoromethyl)pyridine-2-carboxamide (959 mg, 2.74 mmol) and 4-bromo-2-methylbutan-2-ol (330 µL, 2.7 mmol) were dissolved in DMF (9.6 ml, 120 mmol) and potassium carbonate (1.14 g, 8.21 mmol) was added. The reaction mixture was stirred for 24 h at 60 °C. After filtration, the filtrate was diluted with acetonitrile and purified using preparative HPLC. The product fractions were pooled and concentrated *in vacuo* to afford 93 mg (8% yield) of the title compound.

^{1}H NMR (DMSO-d$_6$, 600 MHz) δ 1.17 (s, 6H), 1.88–1.94 (m, 2H), 2.59 (s, 3H), 3.97 (s, 3H), 4.32–4.39 (m, 2H), 7.06 (s, 1H), 8.22 (d, 1H, *J*=7.63 Hz), 8.39-8.44 (m, 1H), 8.44–8.48 (m, 1H), 8.61 (s, 1H), 10.48 (s, 1H).

LC-MS (method B): Rt = 1.27 min; MS (ESIpos): m/z = 437 [M+H]$^+$

Synthesis of compound 38

<u>**Intermediate 38-a**</u>

Methyl 3-(4-methoxy-4-oxobutanamido)-4-(methylamino)benzoate

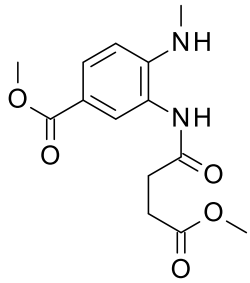

Methyl 3-amino-4-(methylamino)benzoate (5.00 g, 27.7 mmol), 4-methoxy-4-oxobutanoic acid (5.50 g, 41.6 mmol), HATU (15.8 g, 41.6 mmol), and TEA (5.8 ml, 42 mmol) were dissolved in DMF (75 ml) and the reaction mixture was stirred for 6 h at 25 °C. The reaction mixture was diluted with acetonitrile and purified using preparative HPLC (method C). The product fractions were pooled and concentrated *in vacuo* to afford 6.58 g (81% yield) of the title compound.

MS (ESIpos): m/z = 295 $[M+H]^+$

^{1}H NMR (DMSO-d_6, 400 MHz) δ 2.62 (t, 4H, *J*=3.80 Hz), 2.78 (d, *J*=4.82 Hz, 3H), 3.62 (s, 3H), 3.76 (s, 3H), 5.90 (q, *J*=4.82 Hz, 1H), 6.61 (d, *J*=8.36 Hz, 1H), 7.66-7.71 (m, 2H), 9.16 (s, 1H).

<u>**Intermediate 38-b**</u>

Methyl 2-(3-methoxy-3-oxopropyl)-1-methyl-1H-benzimidazole-5-carboxylate

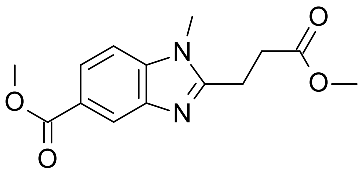

Methyl 3-(4-methoxy-4-oxobutanamido)-4-(methylamino)benzoate (6.58 g, 22.4 mmol) was dissolved in acetic acid (53 ml) and the reaction mixture was stirred for 48 h at 40 °C. The reaction mixture was concentrated *in vacuo* to afford 6.43 g (crude) of the title compound.

MS (ESIpos): m/z = 277 $[M+H]^+$

^{1}H NMR (DMSO-d_6, 400 MHz) δ 2.89–2.98 (m, 2H), 3.12–3.20 (m, 2H), 3.62 (s, 3H), 3.80 (s, 3H), 3.86 (s, 3H), 7.63 (d, 1H, *J*= 8.36 Hz), 7.86 (dd, 1H, *J*=8.36, 1.52), 8.14 (d, 1H, *J*=1.01 Hz).

<u>**Intermediate 38-c**</u>

Methyl 2-(3-methoxy-3-oxopropyl)-1-methyl-6-nitro-1H-benzimidazole-5-carboxylate

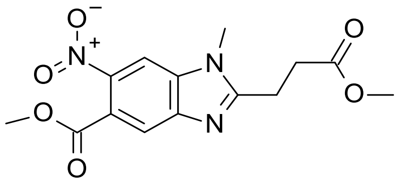

Methyl 2-(3-methoxy-3-oxopropyl)-1-methyl-1H-benzimidazole-5-carboxylate (6.43 g, 23.3 mmol) was dissolved in concentrated sulfuric acid (52 ml, 980 mmol) and a mixture of concentrated nitric acid and sulfuric acid (1:1.4) (2.7 ml, 9.0 M, 24 mmol) was slowly added at 0 °C. The reaction mixture was stirred for 1 h at 25 °C. The reaction mixture was poured into iced water and the pH was adjusted to 10 with sat. sodium bicarbonate solution. The precipitate obtained was collected by filtration and washed with water. The filter cake was dissolved in a mixture of ethyl acetate (300 ml), DCM (200 ml), and acetonitrile (100 ml); dried over anhydrous magnesium sulfate; filtered; and concentrated *in vacuo* to afford 5.48 g (78% yield) of the title compound.

MS (ESIpos): m/z = 322 $[M+H]^+$.

^{1}H NMR (DMSO-d_6, 400 MHz) δ 2.92–2.99 (m, 2H), 3.19–3.24 (m, 2H), 3.62 (s, 3H), 3.84 (s, 3H), 3.89 (s, 3H), 7.96 (s, 1H), 8.46 (s, 1H).

<u>**Intermediate 38-d**</u>

Methyl 6-amino-2-(3-methoxy-3-oxopropyl)-1-methyl-1H-benzimidazole-5-carboxylate

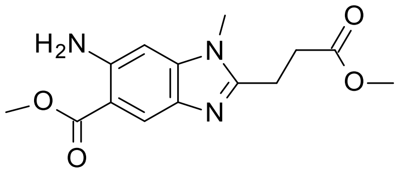

Methyl 2-(3-methoxy-3-oxopropyl)-1-methyl-6-nitro-1H-benzimidazole-5-carboxylate (5.84 g, 18.2 mmol) was dissolved in a mixture of THF (180 mL, 2.2 mol) and methanol (77 mL, 1.9 mol). Palladium on charcoal (10 % Pd, 1.8 mmol) was added and the reaction mixture was stirred for 2 h at 25 °C in a hydrogen atmosphere. After filtration, the filtrate was concentrated *in vacuo* to afford 5.45 g of the title compound (crude batch) that was used without further purification in the next step.

MS (ESIpos): m/z = 292 $[M+H]^+$

^{1}H NMR (DMSO-d_6, 400 MHz) δ 2.83–2.89 (m, 2H), 3.01–3.07 (m, 2H), 3.58 (s, 3H), 3.61 (s, 3H), 3.79 (s, 3H), 6.43 (s, 2H), 6.64 (s, 1H), 7.91 (s, 1H).

<u>Intermediate 38-e</u>

Methyl 2-(3-methoxy-3-oxopropyl)-1-methyl-6-{[6-(trifluoromethyl)pyridine-2-carbonyl]amino}-1H-benzimidazole-5-carboxylate

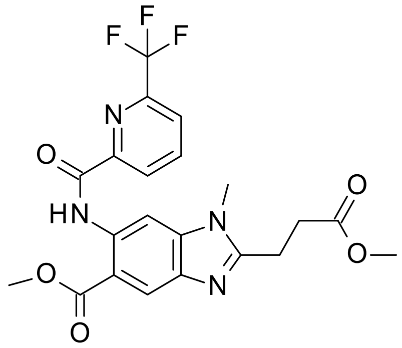

Methyl 6-amino-2-(3-methoxy-3-oxopropyl)-1-methyl-1H-benzimidazole-5-carboxylate (500 mg, 1.72 mmol), 6-(trifluoromethyl)pyridine-2-carboxylic acid (492 mg, 2.57 mmol), HATU (979 mg, 2.57 mmol), and TEA (360 µL, 2.6 mmol) were dissolved in DMF (4.6 ml). The reaction mixture was stirred for 1.5 h at 25 °C. The reaction mixture was poured into water (100 ml) and stirred for 15 minutes. The precipitate obtained was collected by filtration, washed with water, and dried. The filter cake was dissolved in DCM (40 ml), dried over anhydrous magnesium sulfate, filtered, and concentrated *in vacuo* to afford 700 mg (88% yield) of the title compound.
MS (ESIpos): m/z = 465 $[M+H]^+$
^{1}H NMR (DMSO-d_6, 400 MHz) δ 2.91–2.97 (m, 2H), 3.14–3.20 (m, 2H), 3.63 (s, 3H), 3.79 (s, 3H), 3.94 (s, 3H), 8.23 (dd, 1H, *J*=7.73, 0.89 Hz), 8.28 (s, 1H), 8.38–8.44 (m, 1H), 8.47–8.51 (m, 1H), 8.95 (s, 1H), 12.98 (s, 1H).

<u>Compound 38 (step 38-f)</u>

N-[2-(3-Hydroxy-3-methylbutyl)-5-(2-hydroxypropan-2-yl)-1-methyl-1H-benzimidazol-6-yl]-6-(trifluoromethyl)pyridine-2-carboxamide

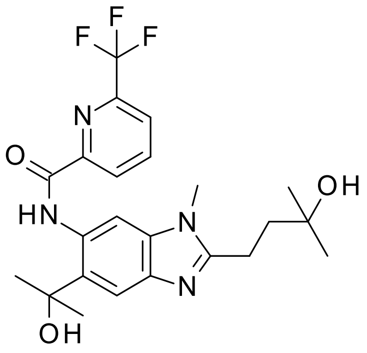

Methyl 2-(3-methoxy-3-oxopropyl)-1-methyl-6-{[6-(trifluoromethyl)pyridine-2-carbonyl]amino}-1H-benzimidazole-5-carboxylate (700 mg, 1.51 mmol) was dissolved in THF (46 ml, 570 mmol) and cooled to 0 °C. Methylmagnesium bromide solution in 2-methyltetrahydrofuran (5.3 ml, 3.4 M, 18 mmol) was added slowly and the reaction mixture was stirred for 24 h at 25 °C. After the addition of sat. ammonium chloride solution (30 ml) the two-phase mixture was stirred for an additional 5 minutes. After phase separation, the organic layer was dried over anhydrous magnesium sulfate, filtered, and concentrated *in vacuo.* The residue was dissolved in THF (45 ml) and cooled to 0 °C. Additional methylmagnesium bromide solution in 2-methyltetrahydrofuran (5.3 ml, 3.4 M, 18 mmol) was added slowly and stirring continued for 1 h at 0 °C. After quenching with sat. ammonium chloride solution and stirring for 5 minutes, the phases were separated, and the organic layer was dried over anhydrous magnesium sulfate, filtered, and concentrated *in vacuo.* The residue was diluted with acetonitrile and purified using preparative HPLC (method D). The product fractions were pooled and concentrated *in vacuo* to afford 105 mg (15% yield) of the title compound.

LC-MS (method A): Rt = 0.90 min; MS (ESIpos): m/z = 465 $[M+H]^+$

^{1}H NMR (DMSO-d_6, 400 MHz) δ 1.18 (s, 6H), 1.61 (s, 6H), 1.81–1.90 (m, 2H), 2.86–2.95 (m, 2H), 3.73 (s, 3H), 4.48 (s, 1H), 5.90 (s, 1H), 7.52 (s, 1H), 8.18 (dd, 1H, *J*=7.73, 0.89 Hz), 8.35–8.41 (m, 1H), 8.44–8.49 (m, 1H), 8.55 (s, 1H), 12.49 (s, 1H).

Synthesis of compound 39

Intermediate 39-a

Methyl 4-(ethylamino)-3-(4-methoxy-4-oxobutanamido)benzoate

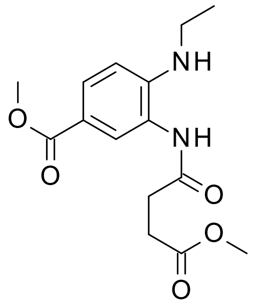

Methyl 3-amino-4-(ethylamino)benzoate (5.00 g, 25.7 mmol), 4-methoxy-4-oxobutanoic acid (5.10 g, 38.6 mmol), HATU (14.7 g, 38.6 mmol), and TEA (5.4 ml, 39 mmol) were dissolved in DMF (66 ml). The reaction mixture was stirred for 24 h at 25 °C and poured into water. The formed suspension was stirred for an additional 10 minutes and the precipitate was collected by filtration and washed with water. The filter cake was dissolved in a mixture of DCM (30 ml), THF (7.5 ml), and acetonitrile (7.5 ml) and dried over anhydrous magnesium sulfate, filtered, and concentrated *in vacuo* to afford 7.73 g (97% yield) of the title compound.
MS (ESIpos): m/z = 309 $[M+H]^+$

Intermediate 39-b

Methyl 1-ethyl-2-(3-methoxy-3-oxopropyl)-1H-benzimidazole-5-carboxylate

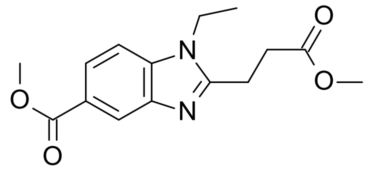

Methyl 4-(ethylamino)-3-(4-methoxy-4-oxobutanamido)benzoate (7.73 g, 25.1 mmol) was dissolved in acetic acid (150 ml). The reaction mixture was stirred for 72 h at 25 °C and 48 h at 60 °C. After concentration *in vacuo* the residue was diluted with toluene and again concentrated *in vacuo*. After repeating this process three times, the product was dried to afford 7.90 g (crude) of the title compound.

MS (ESIpos): m/z = 291 $[M+H]^+$

Intermediate 39-c

Methyl 1-ethyl-2-(3-methoxy-3-oxopropyl)-6-nitro-1H-benzimidazole-5-carboxylate

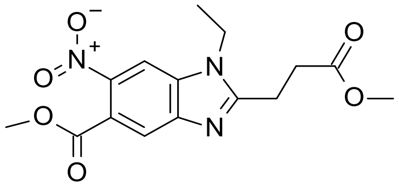

Methyl 1-ethyl-2-(3-methoxy-3-oxopropyl)-1H-benzimidazole-5-carboxylate (7.90 g, 27.2 mmol) was dissolved in concentrated sulfuric acid (61 ml, 1.1 mol) and cooled to 0 °C. A mixture of nitric acid and sulfuric acid (3.2 ml, 9.0 M, 29 mmol) was added. The reaction mixture was stirred for 1.5 h at 25 °C and poured slowly into iced water. The pH was adjusted to 10 via the slow addition of sat. sodium bicarbonate solution. The precipitate was collected by filtration and washed with water. The filter cake was redissolved in DCM, dried over anhydrous magnesium sulfate, filtered, and concentrated *in vacuo.* The crude material was purified using Biotage Isolera™ chromatography (SNAP KP-Sil – 340 g, eluting with hexane-ethyl acetate, 1:0 to 2:3) to afford 7.44 g (82% yield) of the title compound.
MS (ESIpos): m/z = 336 $[M+H]^+$

Intermediate 39-d

Methyl 6-amino-1-ethyl-2-(3-methoxy-3-oxopropyl)-1H-benzimidazole-5-carboxylate

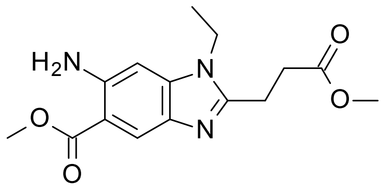

Under argon methyl 1-ethyl-2-(3-methoxy-3-oxopropyl)-6-nitro-1H-benzimidazole-5-carboxylate (6.94 g, 20.7 mmol) was dissolved in THF (200 ml, 2.5 mol) and methanol (77 ml, 1.9 mol) and palladium on charcoal (1.8 ml, 10 % purity, 2.1 mmol) was added. The reaction mixture was stirred for 3 h at 25 °C under a hydrogen atmosphere (1 bar). The reaction mixture was filtered, washed with ethyl acetate and the filtrate was concentrated *in vacuo* to afford 6.25 g (99% yield) of the title compound.
MS (ESIpos): m/z = 306 $[M+H]^+$.

^{1}H NMR (DMSO-d_6, 400 MHz) δ 1.27 (t, 3H, *J*=7.22 Hz), 2.85–2.91 (m, 2H), 3.00–3.06 (m, 2H), 3.60 (s, 3H), 3.79 (s, 3H), 4.06 (q, 2H, *J*=7.10 Hz), 6.41 (s, 2H), 6.67 (s, 1H), 7.91 (s, 1H).

Intermediate 39-e

Methyl 1-ethyl-2-(3-methoxy-3-oxopropyl)-6-{[6-(trifluoromethyl)pyridine-2-carbonyl]amino}-1H-benzimidazole-5-carboxylate

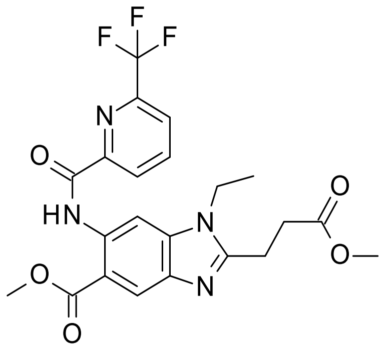

Methyl 6-amino-1-ethyl-2-(3-methoxy-3-oxopropyl)-1H-benzimidazole-5-carboxylate (200 mg, 655 µmol), 6-(trifluoromethyl)pyridine-2-carboxylic acid (188 mg, 983 µmol), HATU (374 mg, 983 µmol), and TEA (140 µL, 980 µmol) were dissolved in DMF (2.9 ml). The reaction mixture was stirred for 24 h at 25 °C. Water was added and the precipitate was collected using filtration, washed with water and hexane, and dried to afford 317 mg (96% yield) of the title compound.

MS (ESIpos): m/z = 479 $[M+H]^+$

^{1}H NMR (CHLOROFORM-d, 400 MHz) δ 1.48 (t, 3H, *J*=7.22 Hz), 3.02–3.11 (m, 2H), 3.16–3.22 (m, 2H), 3.72 (s, 3H), 4.03 (s, 3H), 4.27 (q, 2H, *J*=7.27 Hz), 7.89 (1H, dd, *J*=7.86, 1.01 Hz), 8.13 (t, 1H, *J*=7.86 Hz), 8.49 (d, 1H, *J*=7.35 Hz), 8.52 (s, 1H), 9.03 (s, 1H), 13.28 (s, 1H).

Compound 39 (step 39-f)

N-[1-Ethyl-2-(3-hydroxy-3-methylbutyl)-5-(2-hydroxypropan-2-yl)-1H-benzimidazol-6-yl]-6-(trifluoromethyl)pyridine-2-carboxamide

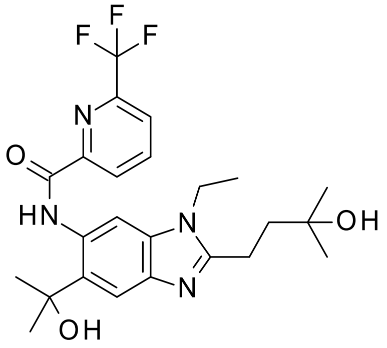

Methyl 1-ethyl-2-(3-methoxy-3-oxopropyl)-6-{[6-(trifluoromethyl)pyridine-2-carbonyl]amino}-1H-benzimidazole-5-carboxylate (282 mg, 590 µmol) was dissolved in THF (18 ml) and cooled to 0 °C under an argon atmosphere. A solution of methylmagnesium bromide in 2-methyltetrahydrofuran (2.1 ml, 3.4 M, 7.1 mmol) was slowly added and the reaction mixture was stirred for 1 h at 0 °C and for 24 h at 25 °C. After the addition of sat. ammonium chloride solution, the phases were separated and the organic layer was dried over anhydrous magnesium sulfate, filtered, and concentrated *in vacuo.* The residue was diluted with acetonitrile and purified using preparative HPLC. The product fractions were pooled and concentrated *in vacuo* to afford 55 mg (19% yield) of the title compound.

LC-MS (method A): Rt = 0.96 min; MS (ESIpos): m/z = 479 $[M+H]^+$

^{1}H NMR (DMSO-d_6, 400 MHz) δ 1.18 (s, 6H), 1.35 (t, 3H, *J*=7.10 Hz), 1.61 (s, 6H), 1.84–1.92 (m, 2H), 2.85–2.93 (m, 2H), 4.20 (q, 2H, *J*=6.93 Hz), 4.48 (s, 1H), 5.88 (s, 1H), 7.52 (s, 1H), 8.17 (dd, 1H, *J*=7.73, 0.89 Hz), 8.35–8.42 (m, 1H), 8.44–8.49 (m, 1H), 8.58 (s, 1H), 12.48 (s, 1H).

Synthesis of compound 40

Intermediate 40-a

***tert*-Butyl {2-[2-(methanesulfonyl)ethyl]-6-methoxy-2H-indazol-5-yl}carbamate**

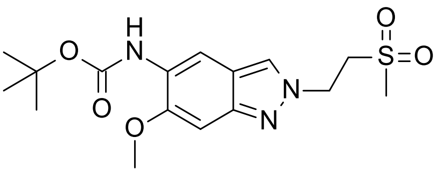

tert-Butyl (6-methoxy-1H-indazol-5-yl)carbamate (intermediate 23-a, 2.50 g, 9.49 mmol) and 1-bromo-2-(methanesulfonyl)ethane (2.66 g, 14.2 mmol) were dissolved in DMF (40 ml) and potassium carbonate (5.25 g, 38.0 mmol) was added. The reaction mixture was stirred for 21 h at 25 °C. After dilution with water, the reaction mixture was extracted with ethyl acetate three times. The combined organic extracts were washed with brine, filtered over a water-free filter, and concentrated *in vacuo.* The crude material was purified using Biotage Isolera™ chromatography (SNAP KP-Sil – 100 g, eluting with hexane-ethyl acetate, 1:0 to 0:1) to afford 1.36 g (35% yield, 89% purity) of the title compound.

MS (ESIpos): m/z = 370 $[M+H]^+$

^1H NMR (DMSO-d_6, 500 MHz) δ 1.47 (s, 9H), 2.86 (s, 3H), 3.81 (t, 2H, *J*=6.83 Hz), 3.86 (s, 3H), 4.77 (t, 2H, *J*=6.99 Hz), 6.99 (s, 1H), 7.80 (s, 1H), 7.92 (br s, 1H), 8.26 (d, 1H, *J*=0.64 Hz).

Intermediate 40-b

2-[2-(Methanesulfonyl)ethyl]-6-methoxy-2H-indazol-5-amine

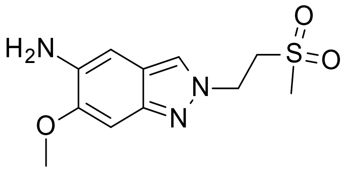

tert-Butyl {2-[2-(methanesulfonyl)ethyl]-6-methoxy-2H-indazol-5-yl}carbamate (1.36 g, 3.68 mmol) was dissolved in DCM (20 ml) and TFA (2.8 ml, 37 mmol) was added. The reaction mixture was stirred for 23 h at 25 °C. The reaction mixture was slowly diluted with sat. sodium bicarbonate solution and, after stirring for 10 minutes, extracted with DCM two times. The

combined organic extracts were washed with brine, filtered over a water-free filter, and concentrated *in vacuo* to afford 930 mg (81% yield) of the title compound.
LC-MS (method B): Rt = 0.58 min; MS (ESIpos): m/z = 270 $[M+H]^+$

Compound 40 (step 40-c)

6-(2-Hydroxypropan-2-yl)-N-{2-[2-(methanesulfonyl)ethyl]-6-methoxy-2H-indazol-5-yl}pyridine-2-carboxamide

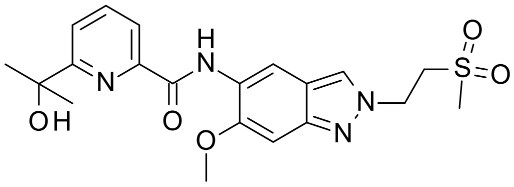

2-[2-(Methanesulfonyl)ethyl]-6-methoxy-2H-indazol-5-amine (100 mg, 371 µmol) was dissolved in THF (2.5 ml) and potassium 6-(2-hydroxypropan-2-yl)pyridine-2-carboxylate (114 mg, crude batch, prepared from methyl 6-(2-hydroxypropan-2-yl)pyridine-2-carboxylate using potassium hydroxide in methanol at 50 °C and subsequent evaporation), EDC (142 mg, 743 µmol), HOBt (56.9 mg, 371 µmol), and TEA (160 µL, 1.1 mmol) were added. The reaction mixture was stirred for 17.5 h at 25 °C, diluted with water, and extracted with ethyl acetate three times. The combined organic extracts were filtered over a water-repellent filter and concentrated *in vacuo.* The residue was diluted with 2.5 mL DMSO and purified using preparative HPLC. The product fractions were pooled and concentrated *in vacuo* to afford 95.3 mg (95% yield) of the title compound.
LC-MS (method B): Rt = 0.89 min; MS (ESIpos): m/z = 433 $[M+H]^+$
^{1}H NMR (DMSO-d_6, 400 MHz) δ 1.56 (s, 6H), 2.89 (s, 3H), 3.82 (t, 2H, *J*=6.95 Hz), 3.99 (s, 3H), 4.79 (t, 2H, *J*=6.95 Hz), 5.43 (s, 1H), 7.13 (s, 1H), 7.92 (dd, 1H, *J*=7.83, 1.26 Hz), 7.98–8.02 (m, 1H), 8.03–8.08 (m, 1H), 8.36 (s, 1H), 8.66 (s, 1H), 10.91 (s, 1H).

Synthesis of compound 41

Intermediate 41-a

5-Bromo-1,2-dimethyl-6-nitro-1H-benzimidazole

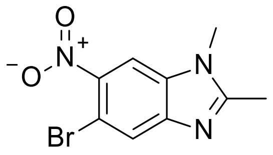

5-Bromo-1,2-dimethyl-1H-benzimidazole 5.60 g (24.9 mmol) was dissolved in concentrated sulfuric acid (16.2 ml). The reaction mixture was cooled to 0 °C and fuming nitric acid (0.98 ml) was then added dropwise overnight while stirring at rt. The reaction mixture was then poured into crushed ice and brought to a pH of 5–6 by adding aqueous ammonia solution (40%). The resulting precipitate was removed by filtration. The residue was purified using silica gel column chromatography (petroleum ether/ethyl acetate gradient). The solvent was removed *in vacuo* and evaporated to dryness to give 6.0 g (72% yield) of the product as a yellow solid.

MS (ESIpos): m/z =270 $(M+H)^+$

Intermediate 41-b

5-Bromo-1,2-dimethyl-1H-benzimidazol-6-amine

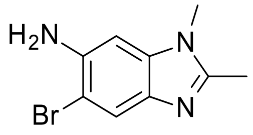

5-Bromo-1,2-dimethyl-6-nitro-1H-benzimidazole (6.00 g, 22.2 mmol) was dissolved in a mixture of THF (100 ml), water (100 ml), and ethanol (25 ml). Ammonium chloride (2.36 g, 44.4 mmol, 2 eq.) and iron (6.22 g, 111 mmol, 5.0 eq.) were added. The resulting mixture was stirred at 80 °C for 1 h. After cooling to rt, iron was removed by filtration. The residue was purified using silica gel column chromatography with methanol and DCM. After evaporation of the solvents *in vacuo*, the product (3.3 g, 61% yield) was obtained as a yellow solid.

MS (ESIpos): m/z = 240 $(M+H)^+$

Intermediate 41-c

N-(5-Bromo-1,2-dimethyl-1H-benzimidazol-6-yl)-6-(trifluoromethyl)pyridine-2-carboxamide

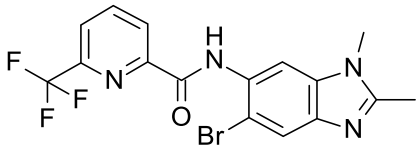

HATU (5.94 g, 15.6 mmol, 1.5 eq.) and TEA (3.6 ml, 26 mmol, 2.5 eq.) were added to a mixture of 5-bromo-1,2-dimethyl-1H-benzimidazol-6-amine (2.50 g, 10.4 mmol) and 6-(trifluoromethyl)pyridine-2-carboxylic acid (2.39 g, 12.5 mmol) in 2-methyltetrahydrofuran and the mixture was stirred for 16 h at rt. Water was added and the solid was removed by filtration with suction and washed with MTBE twice. Afterwards, the solid was stirred in MTBE, filtered, and dried to afford 4.26 g (crude material) of a batch that was used without further purification.
MS (ESIneg): m/z = 411 $[M-H]^-$
^{1}H NMR (DMSO-d_6, 400 MHz) δ selected signals: 3.74 (s, 3H), 7.89 (s, 1H), 8.25 (dd, 1H, *J*=1.0, 7.6 Hz), 8.38–8.49 (m, 3H), 10.55 (s, 1H).

Compound 41 (step 41-d)

N-[1,2-Dimethyl-5-(1H-pyrazol-3-yl)-1H-benzimidazol-6-yl]-6-(trifluoromethyl)pyridine-2-carboxamide

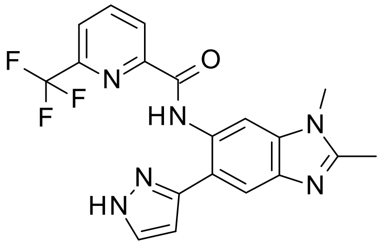

N-(5-Bromo-1,2-dimethyl-1H-benzimidazol-6-yl)-6-(trifluoromethyl)pyridine-2-carboxamide (120 mg, 290 µmol), 1H-pyrazol-3-ylboronic acid (65.0 mg, 581 µmol), potassium carbonate, and XPhos (dicyclohexyl(2',4',6'-triisopropylbiphenyl-2-yl)phosphan, 8.31 mg, 17.4 µmol) were added to a microwave vial. Afterwards, 1,4-dioxane (2.4 ml) and water (0.8 ml) were added and the vial was flushed with nitrogen for 5 minutes. XPhos Pd G2 ((2'-amino[biphenyl]-2-yl)(chloro)palladium–dicyclohexyl(2',4',6'-triisopropyl[biphenyl]-2-yl)phosphine (1:1), 6.86 mg, 8.71 µmol) was added and the mixture was stirred in a

microwave reactor at 120 °C for 2 h. Water and ethyl acetate were added and the resulting solid was filtered off, washed two times with water and three times with MTBE, and dried to afford 62.1 mg (53% yield) of the title compound.

LC-MS (method B): Rt = 1.00 min; MS (ESIpos): m/z = 401 $[M+H]^+$

^{1}H NMR (DMSO-d_6, 400 MHz) δ 2.55 (s, 3H), 3.38 (s, 3H), 6.84–6.87 (m, 1H), 7.86–7.89 (m, 1H), 7.92 (s, 1H), 8.16–8.20 (m, 1H), 8.38 (t, 1H, *J*=7.7 Hz), 8.46 (d, 1H, *J*=7.9 Hz), 8.89 (s, 1H), 12.75 (br s, 1H), 13.03 (s, 1H).

Figure S5. Original analytical data of test compounds

Compound 6

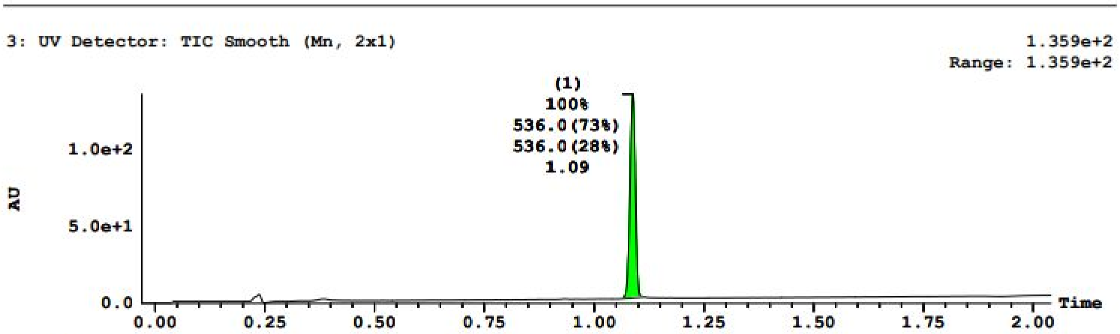

Peak Number	Compound	Time	AreaAbs	Area %Total	Mass Found
1	Found	1.09	1880513	100.00	536.00, 536.00

Compound 7

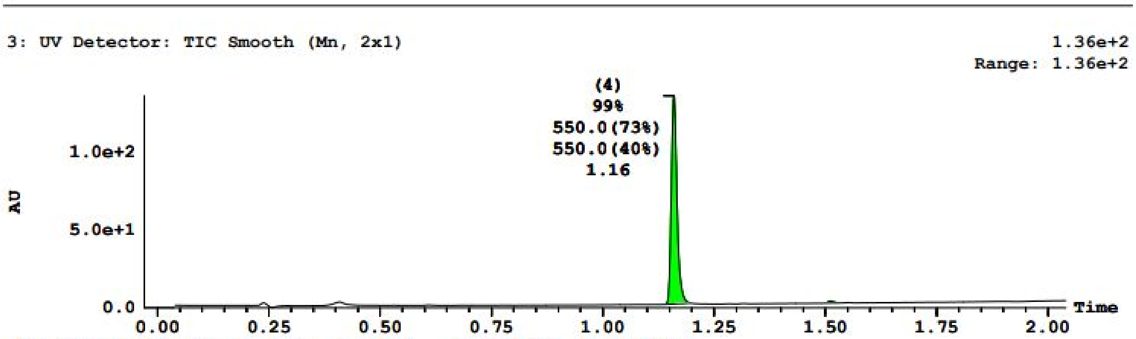

Peak Number	Compound	Time	AreaAbs	Area %Total	Mass Found
4	Found	1.16	2001836	99.00	550.00, 550.00
6	Found	1.51	20185	1.00	550.00

Compound 8

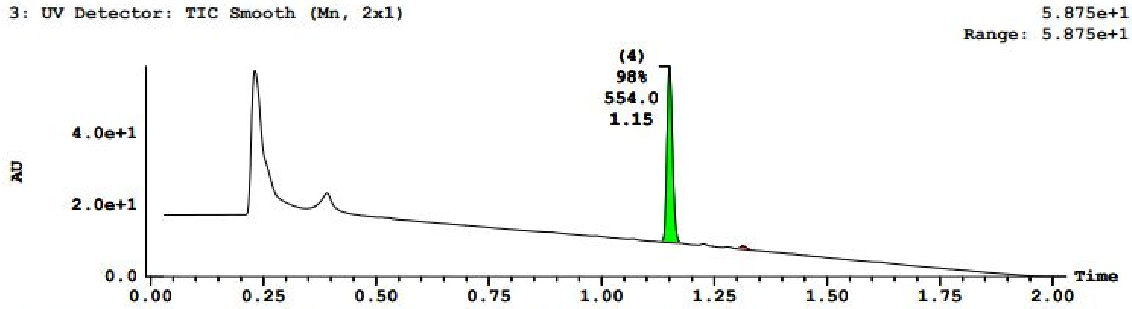

Peak Number	Compound	Time	AreaAbs	Area %Total	Mass Found
4	Found	1.15	7e+005	98.01	554.00
5		1.31	1e+004	1.99	

Compound 9

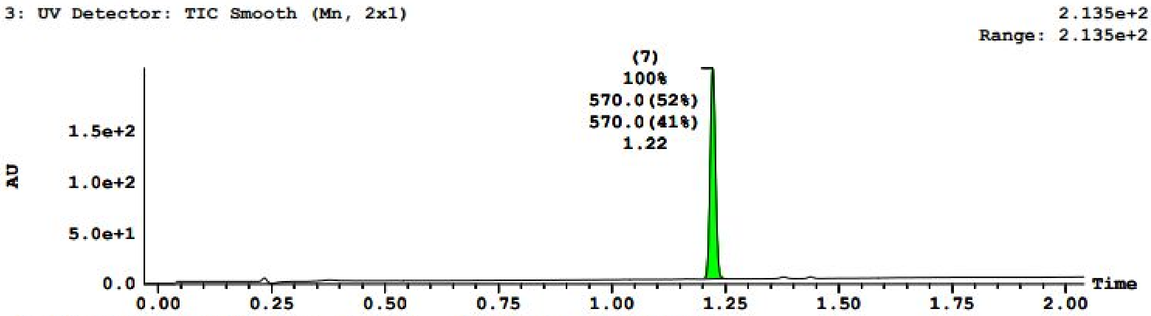

Peak Number	Compound	Time	AreaAbs	Area %Total	Mass Found
7	Found	1.22	2902978	100.00	570.00, 570.00

Compound 10

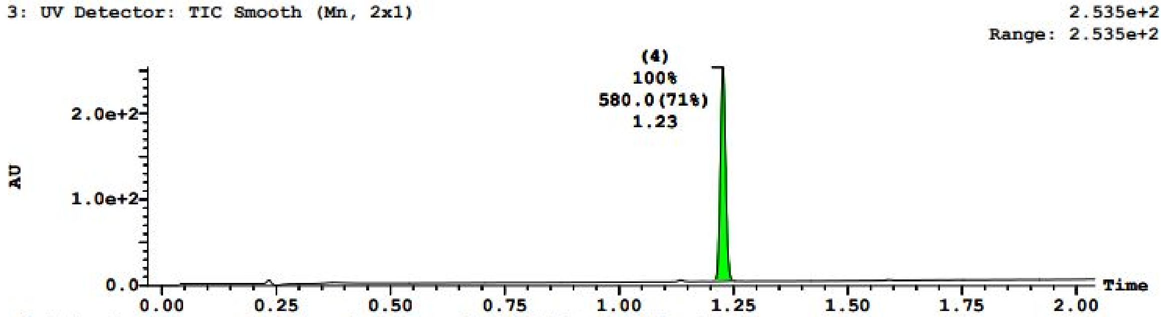

Peak Number	Compound	Time	AreaAbs	Area %Total	Mass Found
4	Found	1.23	3382051	100.00	580.00

Compound 11

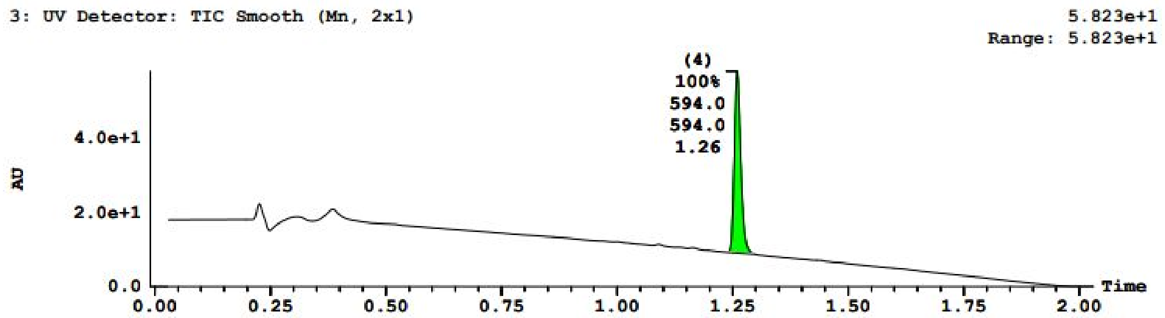

Peak Number	Compound	Time	AreaAbs	Area %Total	Mass Found
4	Found	1.26	8e+005	100.00	594.00, 594.00

Compound 12

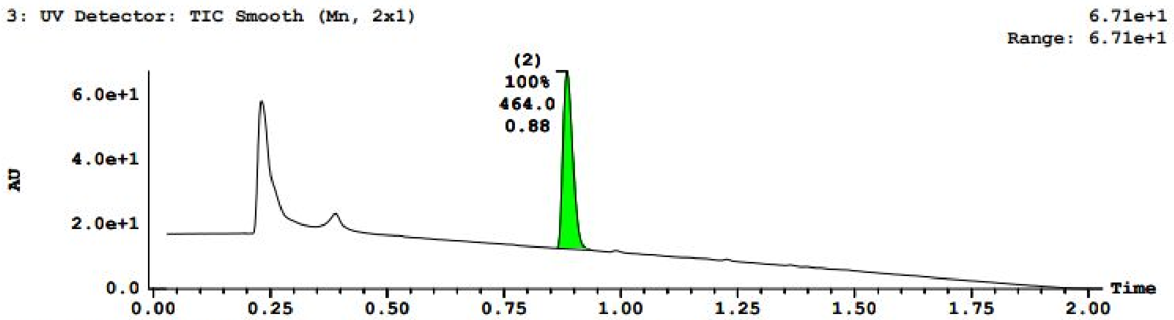

Peak Number	Compound	Time	AreaAbs	Area %Total	Mass Found
2	Found	0.88	1e+006	100.00	464.00

Compound 13

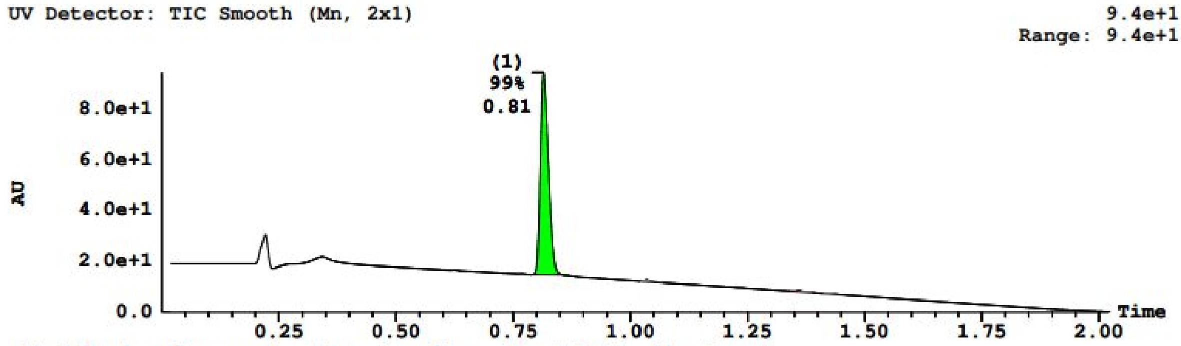

Peak Number	Compound	Time	AreaAbs	Area %Total	Mass Found
1	Found	0.81	1589592	99.03	471.16, 471.16
2		1.03	6976	0.43	
4		1.36	5200	0.32	
5		1.43	3387	0.21	

Compound 14

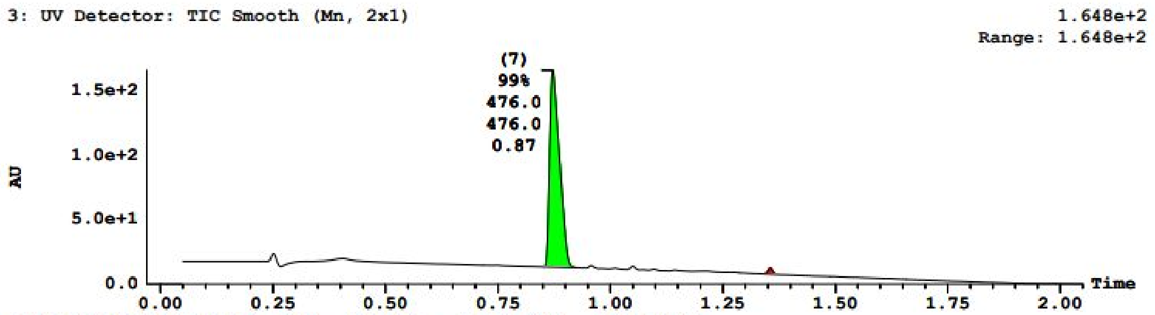

Peak Number	Compound	Time	AreaAbs	Area %Total	Mass Found
7	Found	0.87	4e+006	98.55	476.00, 476.00
10		1.36	6e+004	1.45	

Compound 15

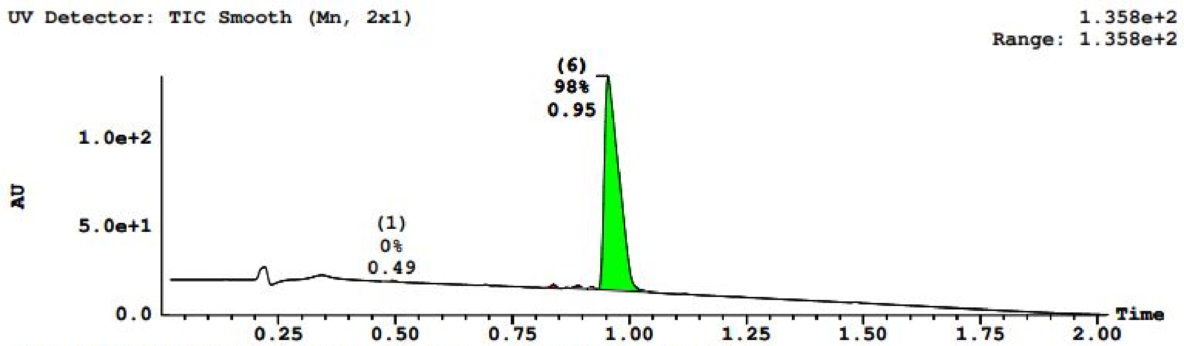

Peak Number	Compound	Time	AreaAbs	Area %Total	Mass Found
1		0.49	12419	0.28	
3		0.84	26138	0.59	
4		0.89	27282	0.61	
5		0.92	16776	0.38	
6	Found	0.95	4358943	98.14	530.15, 530.15

Compound 16

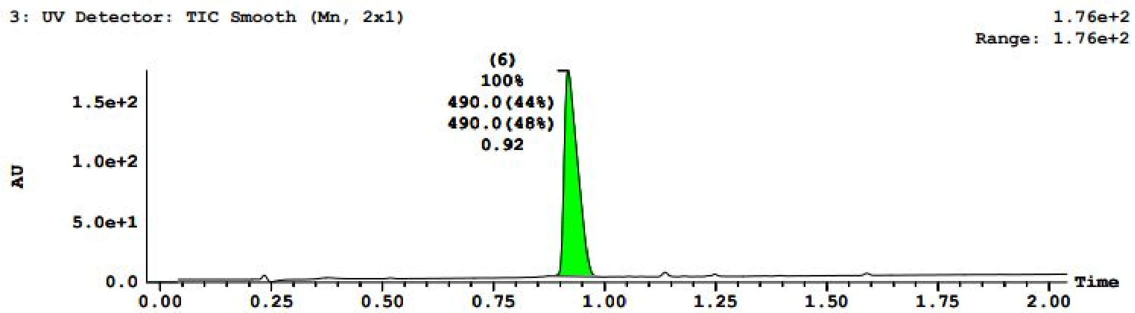

Peak Number	Compound	Time	AreaAbs	Area %Total	Mass Found
6	Found	0.92	5940892	100.00	490.00, 490.00

Compound 17

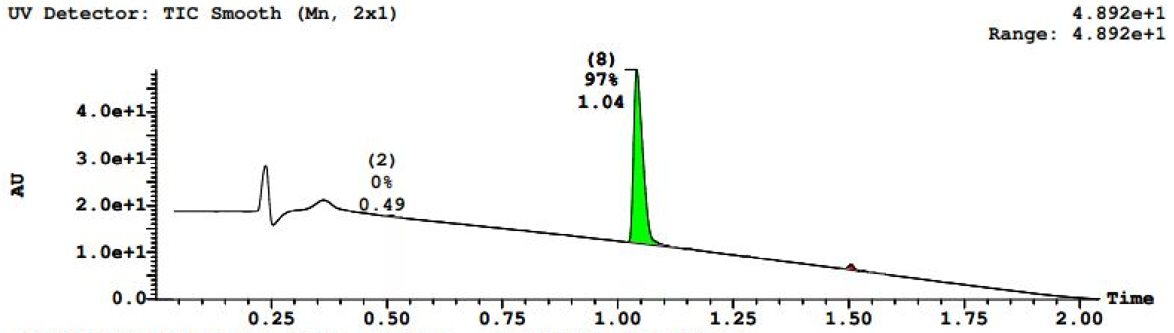

Peak Number	Compound	Time	AreaAbs	Area %Total	Mass Found
2		0.51	3527	0.41	
8	Found	1.04	841929	97.25	504.21, 504.21
11		1.51	16318	1.88	
11		1.52	1761	0.20	
12	Found	1.54	2202	0.25	504.21

Compound 18

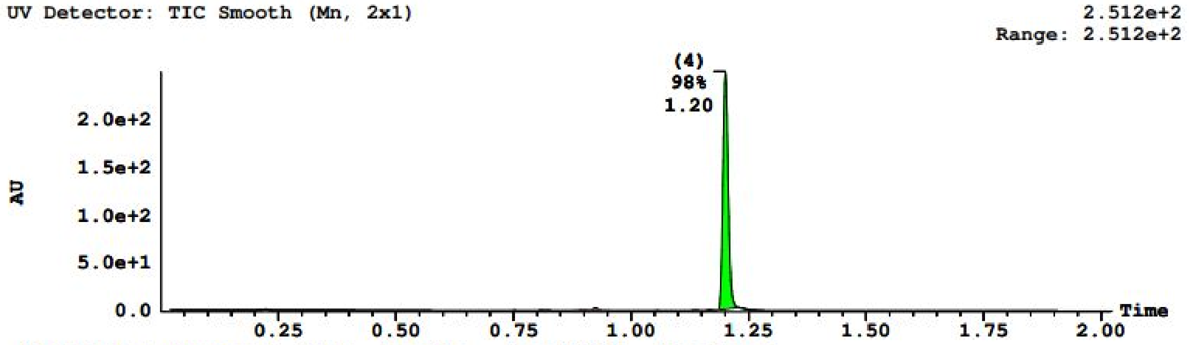

Peak Number	Compound	Time	AreaAbs	Area %Total	Mass Found
1		0.90	17166	0.51	
2		0.92	31265	0.92	
3		1.14	15764	0.47	
4	Found	1.20	3316022	98.10	516.21, 516.21

Compound 19

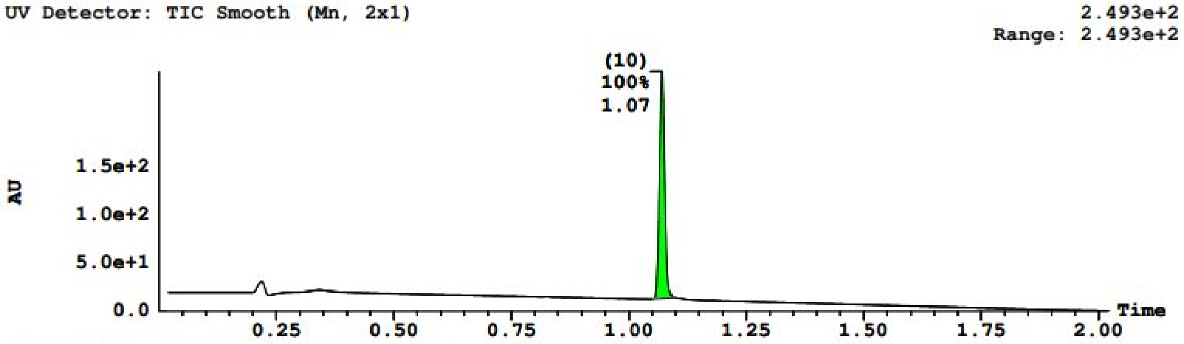

Peak Number	Compound	Time	AreaAbs	Area %Total	Mass Found
10	Found	1.07	3060362	100.00	463.15, 463.15

Compound 20

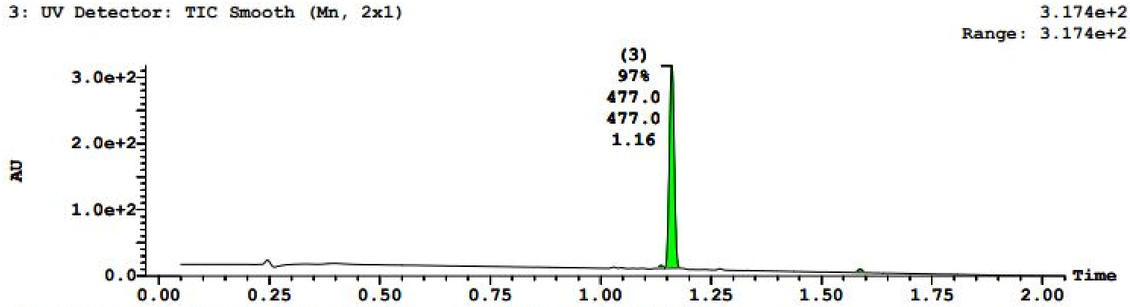

Peak Number	Compound	Time	AreaAbs	Area %Total	Mass Found
3	Found	1.14	5e+004	1.14	477.00, 477.00
3	Found	1.16	4e+006	97.27	477.00, 477.00
6	Found	1.59	6e+004	1.60	477.00

Compound 21

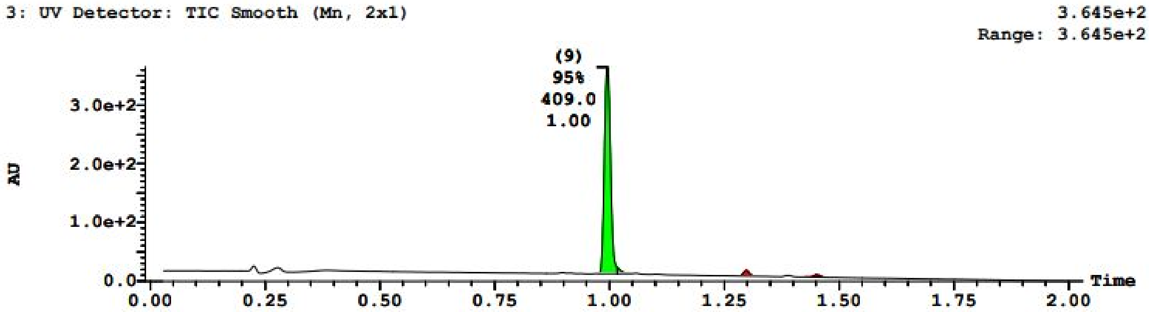

Peak Number	Compound	Time	AreaAbs	Area %Total	Mass Found
9	Found	1.00	5e+006	94.53	409.00
9	Found	1.02	9e+004	1.66	409.00
10		1.30	1e+005	2.69	
11		1.45	6e+004	1.12	

Compound 22

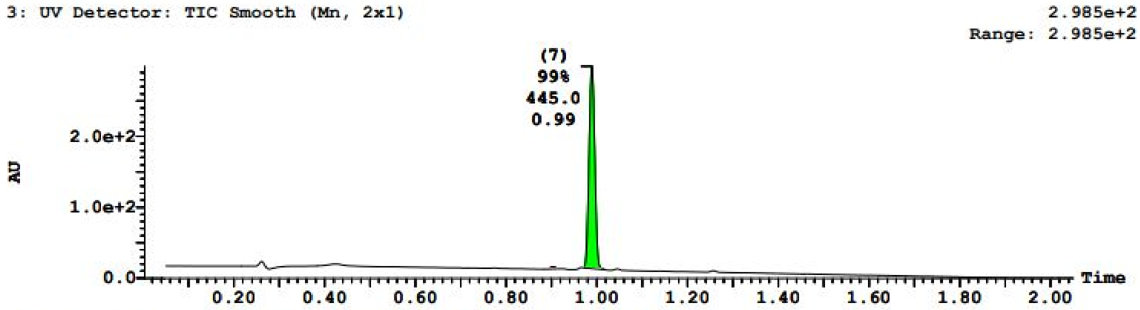

Peak Number	Compound	Time	AreaAbs	Area %Total	Mass Found
5		0.90	4e+004	1.00	
7	Found	0.99	4e+006	99.00	445.00

Compound 23

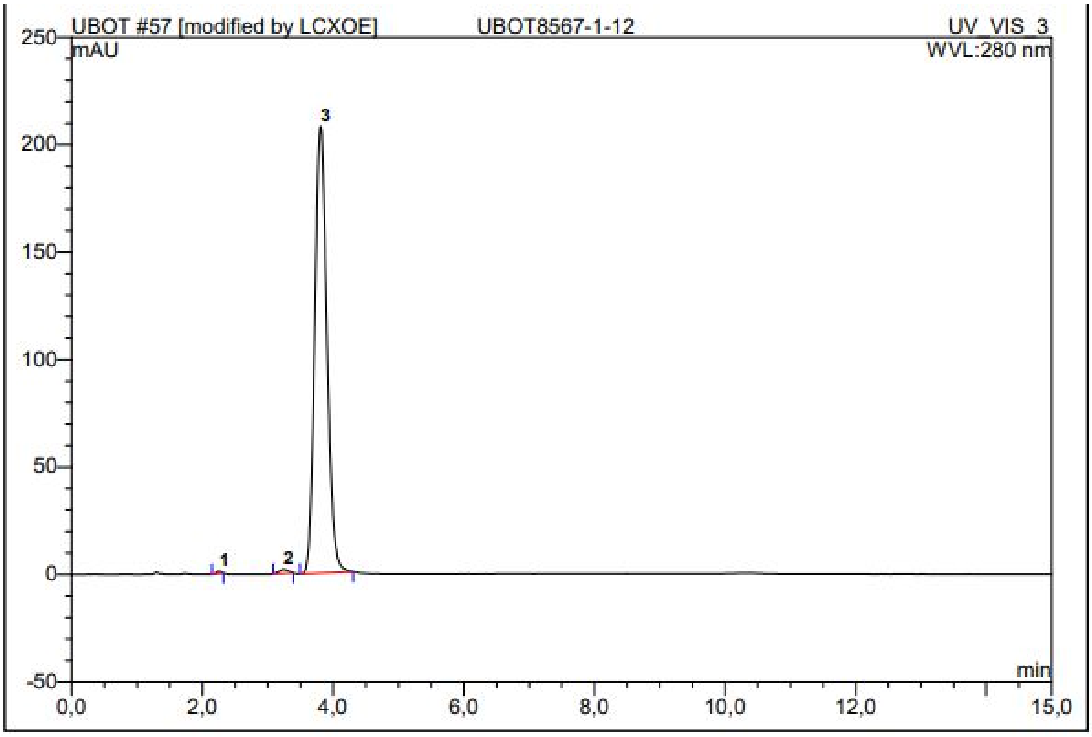

No.	Ret.Time min	Peak Name	Height mAU	Area mAU*min	Rel.Area %	Amount	Type
1	2,26	n.a.	1,085	0,098	0,22	n.a.	BMB*
2	3,24	n.a.	1,890	0,308	0,68	n.a.	BMB*
3	3,81	n.a.	207,920	44,604	99,10	n.a.	BMB*
Total:			210,895	45,010	100,00	0,000	

Compound 24

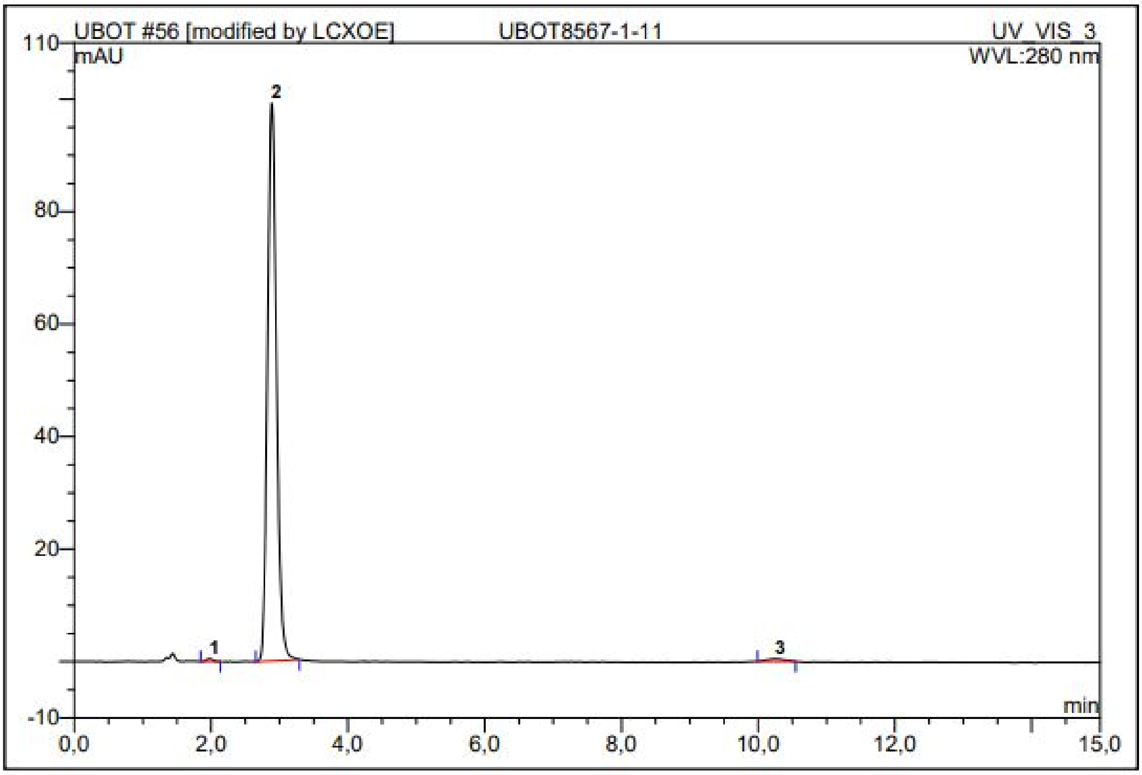

No.	Ret.Time min	Peak Name	Height mAU	Area mAU*min	Rel.Area %	Amount	Type
1	1,98	n.a.	0,461	0,051	0,33	n.a.	BMB*
2	2,88	n.a.	99,132	15,052	98,80	n.a.	BMB*
3	10,25	n.a.	0,437	0,132	0,86	n.a.	BMB*
Total:			100,030	15,234	100,00	0,000	

Compound 25

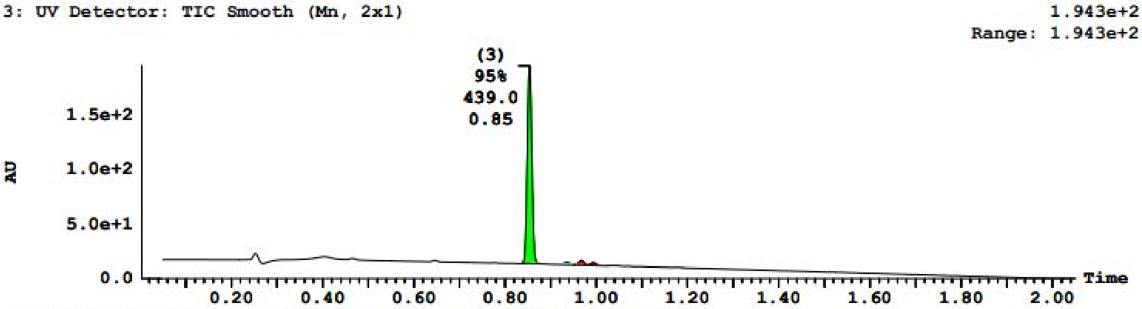

Peak Number	Compound	Time	AreaAbs	Area %Total	Mass Found
3	Found	0.85	2e+006	95.07	439.00
4	Found	0.94	3e+004	1.42	439.00
5		0.97	5e+004	2.25	
6		0.99	3e+004	1.26	

Compound 26

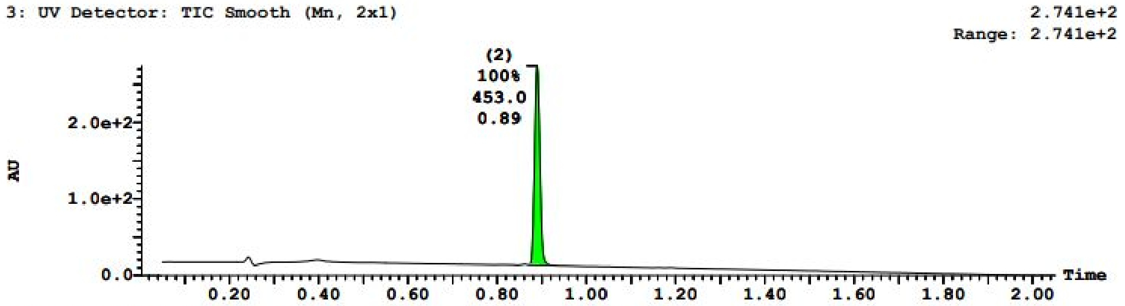

Peak Number	Compound	Time	AreaAbs	Area %Total	Mass Found
2	Found	0.89	4e+006	100.00	453.00

Compound 27

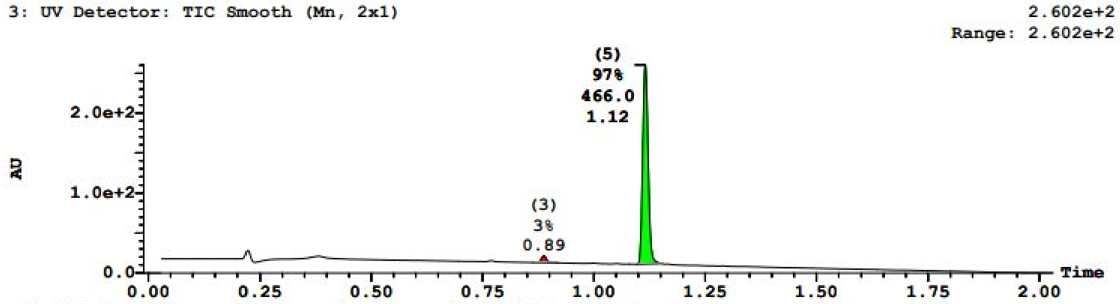

Peak Number	Compound	Time	AreaAbs	Area %Total	Mass Found
3		0.89	1e+005	3.00	
5	Found	1.12	3e+006	97.00	466.00

Compound 28

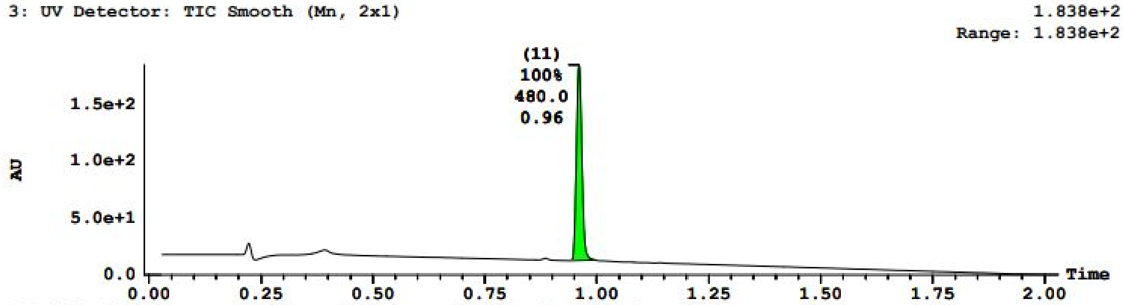

Peak Number	Compound	Time	AreaAbs	Area %Total	Mass Found
11	Found	0.96	2e+006	100.00	480.00

Compound 29

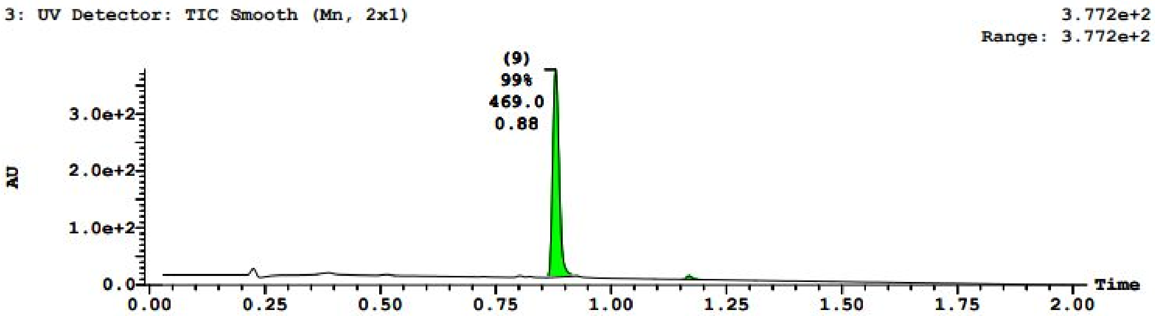

Peak Number	Compound	Time	AreaAbs	Area %Total	Mass Found
9	Found	0.88	6e+006	98.55	469.00
12	Found	1.17	9e+004	1.45	469.00

Compound 30

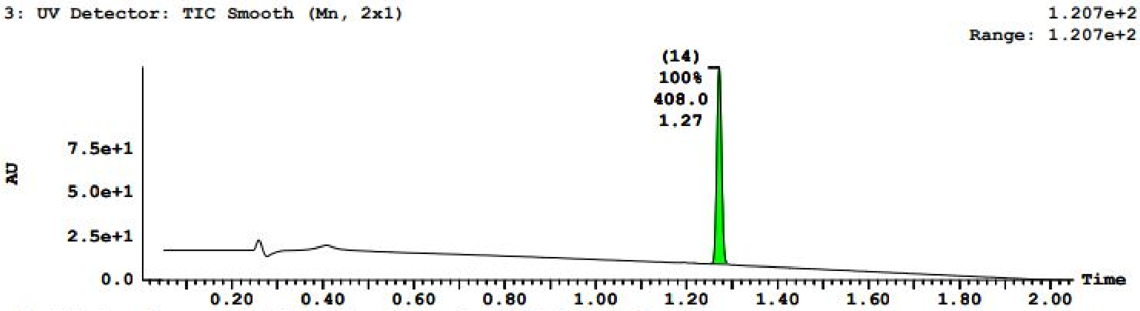

Peak Number	Compound	Time	AreaAbs	Area %Total	Mass Found
14	Found	1.27	1e+006	100.00	408.00

Compound 31

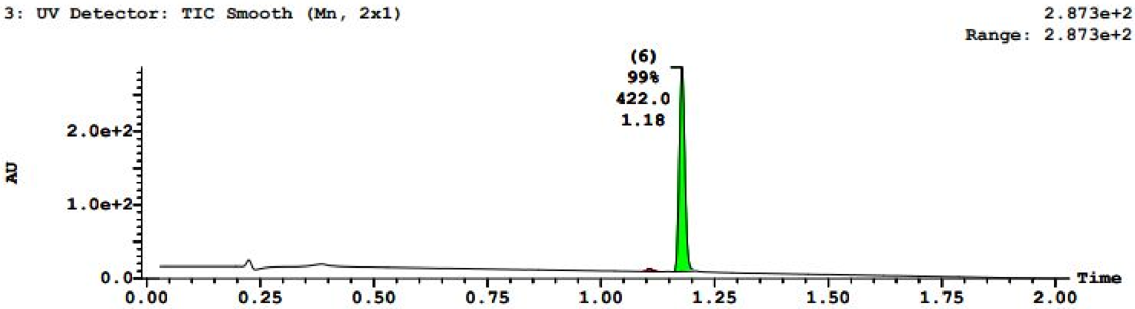

Peak Number	Compound	Time	AreaAbs	Area %Total	Mass Found
5		1.11	6e+004	1.43	
6	Found	1.18	4e+006	98.57	422.00

Compound 32

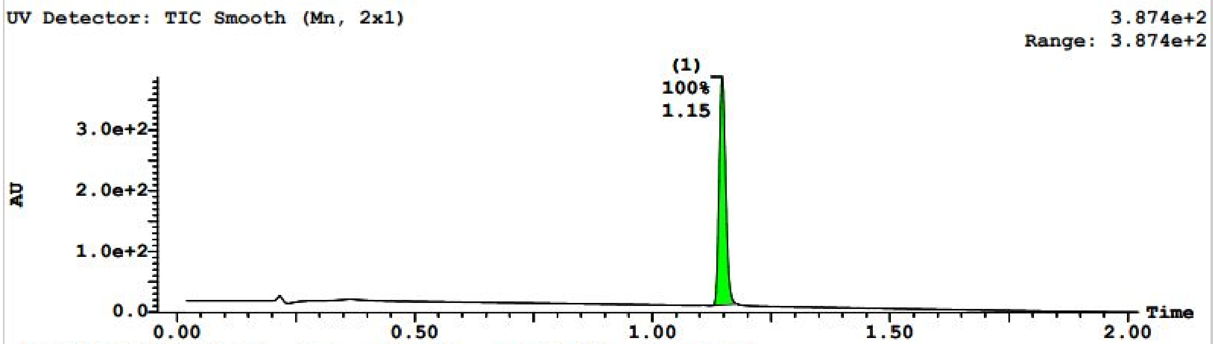

Peak Number	Compound	Time	AreaAbs	Area %Total	Mass Found
1	Found	1.15	6093591	100.00	478.18, 478.18

Compound 33

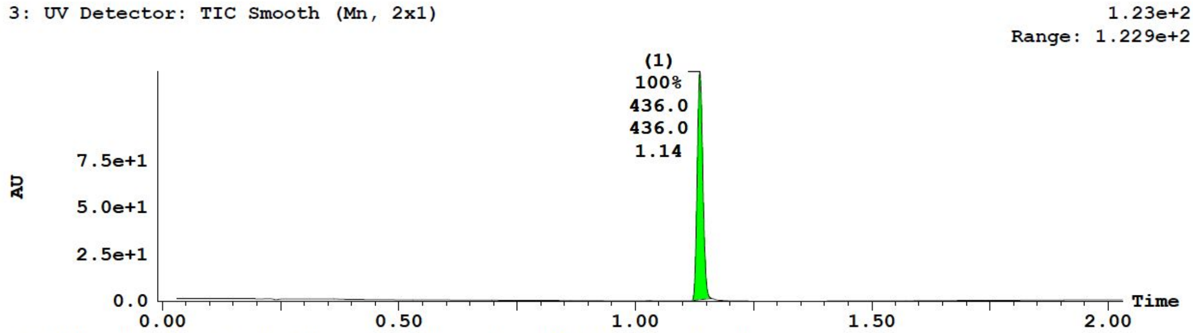

Peak Number	Compound	Time	AreaAbs	Area %Total	Mass Found
1	Found	1.14	2e+006	100.00	436.00, 436.00

Compound 34

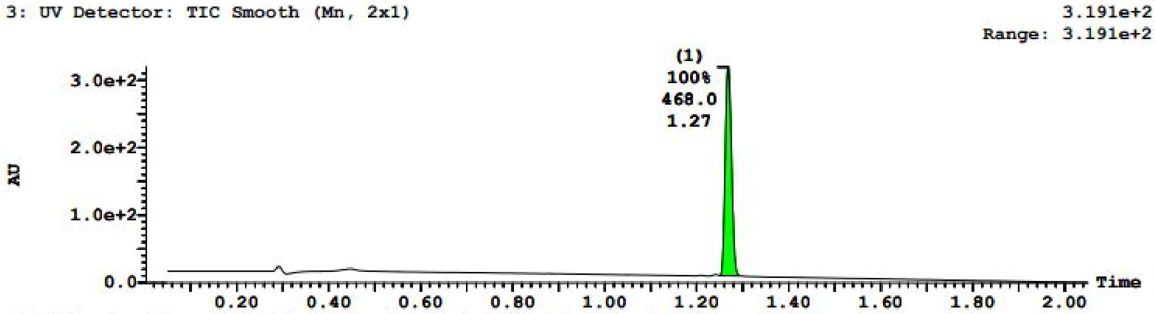

Peak Number	Compound	Time	AreaAbs	Area %Total	Mass Found
1	Found	1.27	5e+006	100.00	468.00

Compound 35

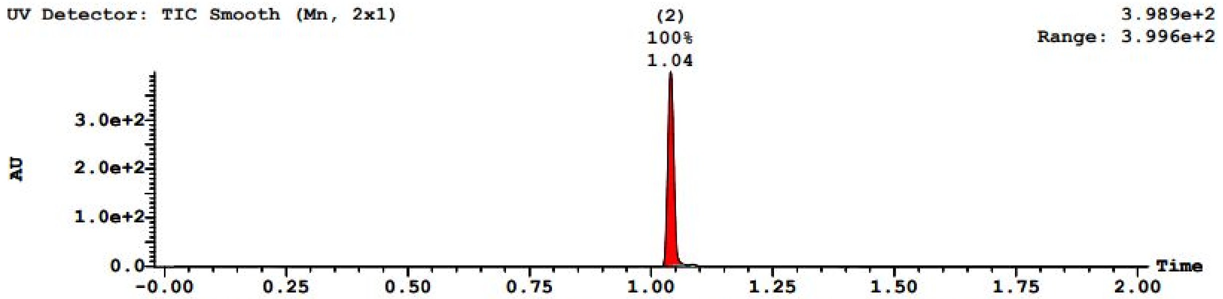

#	Spectrum	RT	Area %	Pos Neg UV	Mass Found
2	---	1.04	100.00	937, 467, 319	

Peak ID	Compound	Time	Mass Found
2		1.05	

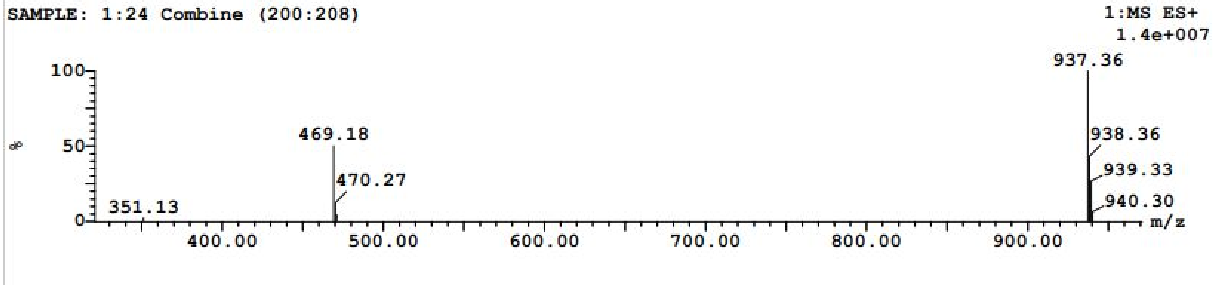

Compound 36

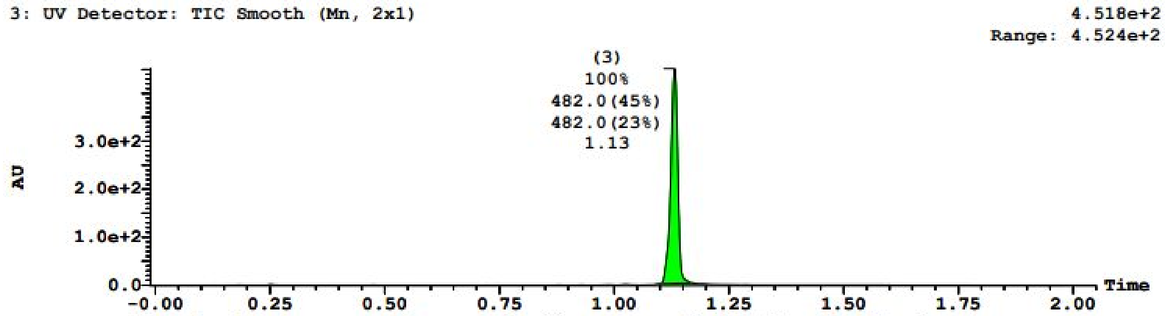

#	Spectrum	RT	Area %	Pos Neg UV	Mass Found
3	---	1.13	100.00	483, 481, 218	482.00, 482.00

Compound 37

Sample analyzed by NMR only:

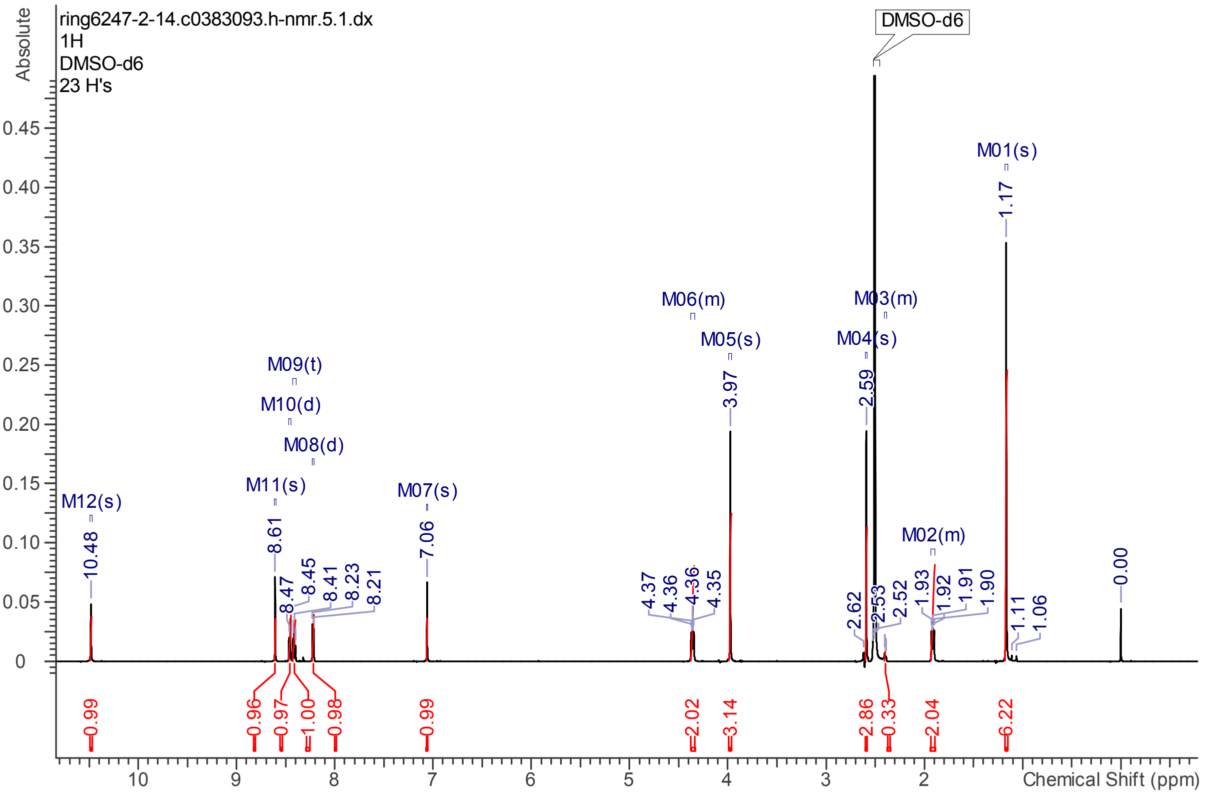

^{1}H NMR (DMSO-d$_6$, 600 MHz) δ 10.48 (s, 1H), 8.61 (s, 1H), 8.46 (d, 1H, *J*=7.6 Hz), 8.41 (t, 1H, *J*=7.6 Hz), 8.22 (d, 1H, *J*=7.6 Hz), 7.06 (s, 1H), 4.3-4.4 (m, 2H), 3.97 (s, 3H), 2.59 (s, 3H), 2.4-2.4 (m, 1H), 1.9-1.9 (m, 2H), 1.17 (s, 6H).

Compound 38

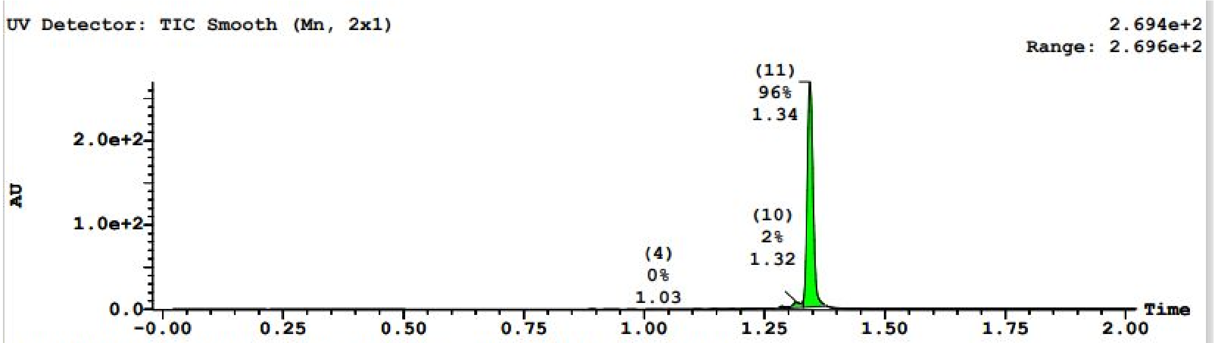

Peak Number	Compound	Time	AreaAbs	Area %Total	Mass Found
4		1.03	11600	0.29	
9	Found	1.29	34978	0.89	464.20, 464.20
10	Found	1.32	94605	2.40	464.20, 464.20
11	Found	1.34	3794020	96.41	464.20, 464.20

Compound 39

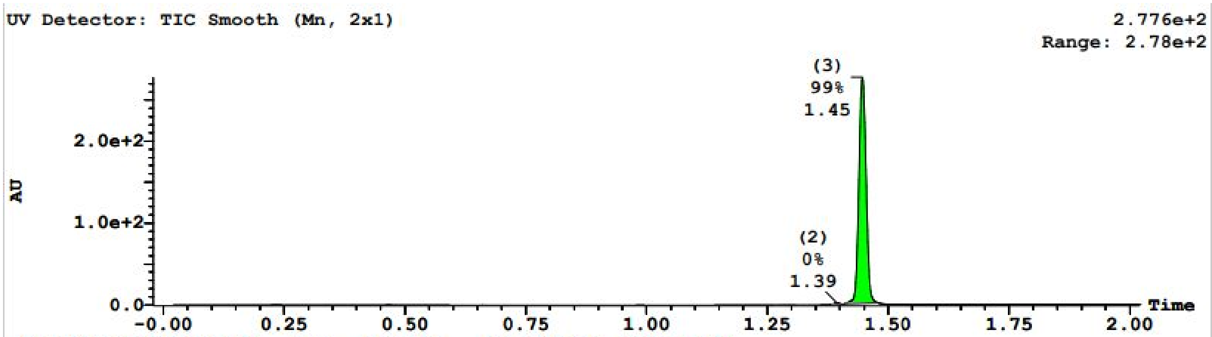

Peak Number	Compound	Time	AreaAbs	Area %Total	Mass Found
1		1.30	22026	0.47	
2		1.39	21924	0.47	
3	Found	1.45	4668442	99.07	478.22, 478.22

Compound 40

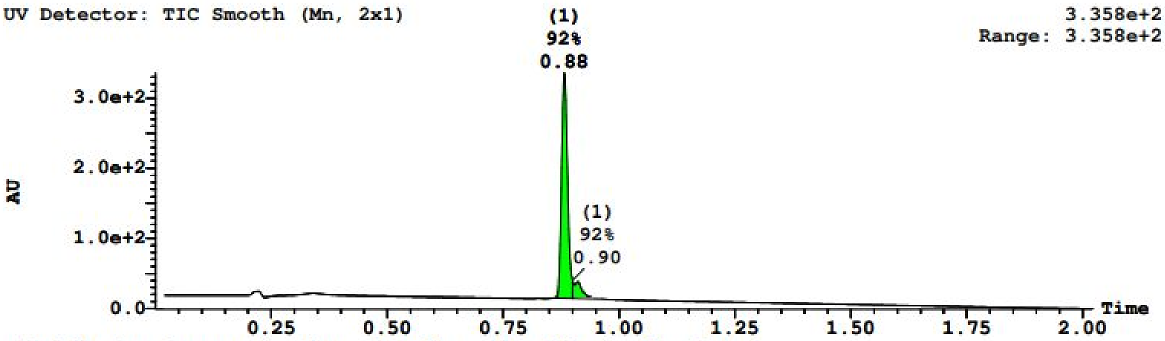

Peak Number	Compound	Time	AreaAbs	Area %Total	Mass Found
1	Found	0.88	4973829	92.13	432.15
2	Found	0.91	424951	7.87	432.15

Compound 41

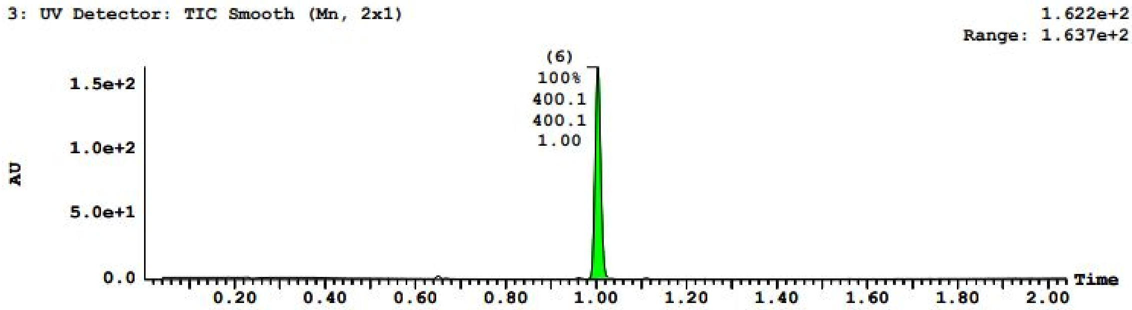

#	Spectrum	RT	Area %	Pos Neg UV	Mass Found
6	---	1.00	100.00	401, 399, 226	400.10, 400.10

Experimental section: physicochemical assays

Aqueous Solubility of Compound from DMSO solutions

Aqueous solubility at pH 6.5 was determined using an orientating HTS method.[13] Test compounds were applied as 1 mM DMSO solutions. After the addition of a buffer, pH 6.5, solutions were shaken for 24 h at rt. Undissolved material was removed by filtration. The compound dissolved in the filtrate was quantified using HPLC-MS/MS.

References

(1) Fabian, M. A.; Biggs, W. H., 3rd; Treiber, D. K.; Atteridge, C. E.; Azimioara, M. D.; Benedetti, M. G.; Carter, T. A.; Ciceri, P.; Edeen, P. T.; Floyd, M.; et al. A small molecule-kinase interaction map for clinical kinase inhibitors. *Nat Biotechnol* **2005**, *23*, 329-336.
(2) Kabsch, W. Integration, scaling, space-group assignment and post-refinement. *Acta Crystallographica Section D* **2010**, *66*, 133-144.
(3) Vagin, A.; Teplyakov, A. Molecular replacement with MOLREP. *Acta Crystallogr D Biol Crystallogr* **2010**, *66*, 22-25.
(4) Murshudov, G. N.; Skubak, P.; Lebedev, A. A.; Pannu, N. S.; Steiner, R. A.; Nicholls, R. A.; Winn, M. D.; Long, F.; Vagin, A. A. REFMAC5 for the refinement of macromolecular crystal structures. *Acta Crystallographica Section D* **2011**, *67*, 355-367.
(5) Emsley, P.; Lohkamp, B.; Scott, W. G.; Cowtan, K. Features and development of Coot. *Acta Crystallogr D Biol Crystallogr* **2010**, *66*, 486-501.
(6) Schüttelkopf, A. W.; van Aalten, D. M. PRODRG: a tool for high-throughput crystallography of protein-ligand complexes. *Acta Crystallogr D Biol Crystallogr* **2004**, *60*, 1355-1363.
(7) Friesner, R. A.; Banks, J. L.; Murphy, R. B.; Halgren, T. A.; Klicic, J. J.; Mainz, D. T.; Repasky, M. P.; Knoll, E. H.; Shelley, M.; Perry, J. K.; et al. Glide: a new approach for rapid, accurate docking and scoring. 1. Method and assessment of docking accuracy. *J Med Chem* **2004**, *47*, 1739-1749.
(8) Halgren, T. A.; Murphy, R. B.; Friesner, R. A.; Beard, H. S.; Frye, L. L.; Pollard, W. T.; Banks, J. L. Glide: a new approach for rapid, accurate docking and scoring. 2. Enrichment factors in database screening. *J Med Chem* **2004**, *47*, 1750–1759.
(9) Jones, G.; Willett, P.; Glen, R. C.; Leach, A. R.; Taylor, R. Development and validation of a genetic algorithm for flexible docking. *J Mol Biol* **1997**, *267*, 727–748.
(10) *BIOVIA Pipeline Pilot*; Dassault Systèmes: San Diego.
(11) Gasteiger, J.; Rudolph, C.; Sadowski, J. Automatic generation of 3D-atomic coordinates for organic molecules. *Tetrahedron Computer Methodology* **1990**, *3*, 537-547.
(12) *ADMET Predictor*; Simulations Plus, Inc: Lancaster, CA, 2017.
(13) Onofrey, T.; Kazan, G. Performance and Correlation of a 96-Well High Throughput Screening Method to Determine Aqueous Drug Solubility. In *MultiScreen® Solubility Filter Plate protocol note.*, Millipore Corporation: Billerica, MA USA, 2003.
